# Acknowledgment to Reviewers of *IJERPH* in 2020

**DOI:** 10.3390/ijerph18031259

**Published:** 2021-01-30

**Authors:** IJERPH Editorial Office

**Affiliations:** MDPI AG, St. Alban-Anlage 66, 4052 Basel, Switzerland

Peer review is the driving force of journal development, and reviewers are gatekeepers who ensure that *IJERPH* maintains its standards for the high quality of its published papers. Thanks to the cooperation of our reviewers, in 2020, the median time to first decision was 17 days and the median time to publication was 39 days. The editors would like to express their sincere gratitude to the following reviewers for their precious time and dedication, regardless of whether the papers were finally published:

Aadahl, MetteLingamdinne, Lakshmi PrasannaAadland, Katrine NyvollLinhardt, TinaAbad Robles, Manuel TomásLinks, PaulAbad-Segura, EmilioLinnenbringer, ErinAbakumov, EvgenyLins De-Azevedo-Vaz, SergioAbalasei, Aurelia BeatriceLinsen, LarsAbarca-Alvarez, Francisco JavierLintermann, AndreasAbarca-Sos, AlbertoLinz, AmandaAbate, GiuliaLinzalone, NunziaAbballe, AnnalisaLiobikienė, GenovaitėAbbas, AzharLionetti, VincenzoAbbas, FaisalLiotta, GiuseppeAbbas, Hasriwiani HaboLiou, Chih-lingAbbas, JaffarLiou, MichelleAbbas, TauqeerLipowicz, JérômeAbbott, Robert D.Lipowska, MałgorzataAbd El Hakim, YasminaLipowski, MariuszAbdallah, AliLippke, SoniaAbdallah, Mohamed F. Lippo, EnricoAbdelbasset, Walid KamalLipski, MariuszAbdelrady, AhmedLira, JenniferAbdelwhab, El-Sayed M.Lirola, María-JesúsAbdi, RezaLIS, AdrianaAbdin, EdimansyahLis, PiotrAbdul Rahman, Noor AishaLisa, AntonellaAbduljabbar, RusulLisek, JerzyAbe, SusumuLisetskii, FedorAbe, TakashiLisiński, PrzemysławAbe, TakumiLisowska, BarbaraAbed, Hassan H.Litofsky, Norman ScottAbedelmalek, SalmaLitschauer, BrigitteAbedi, TayebehLittle, JulianAbejón, RicardoLittle, SabineAbel, MarkLitvan, IreneAbeles, ShiraLitwic-Kaminska, KamilaAbell, NeilLiu, AijunAbellan-Aynes, OriolLiu, AilingAbend, MichaelLiu, BenmeiAberer, FelixLiu, BianAbia, Akebe Luther KingLiu, BinbinAboagye, EmmanuelLiu, BingAboelnga, Hassan TolbaLiu, BoAbós, ÁngelLiu, CaixiaAbou Rafee, Sameh A.Liu, CanAboul-Enein, BasilLiu, ChaoxingAbraczinskas, MichelleLiu, ChengqingAbraham, EyalLiu, Chen-YuAbrahão Nencioni, Ana LeonorLiu, Cheuk LunAbramyan, JohnLiu, Chia-ChiAbrantes, JoãoLiu, DanpingAbreu, Ana MariaLiu, DexinAbreu, IsabelLiu, DiAbreu, WilsonLiu, DuanyangAbril, Eulàlia PuigLiu, FilipeAbswoude, Femke VanLIU, GangAbugri, Daniel A.Liu, GeorgeAbuin, VanesaLiu, GuanQunAbuyassin, Bisher H.Liu, GuicaiAcampa, GiovannaLiu, HaojieAccinelli, ElvioLiu, HengruiAceytuno, María-TeresaLiu, HongyouAchal, VarenyamLiu, Hsiu-YuehAcharya, Bipin KumarLiu, HuAcharya, PayelLiu, Hua-MinAcharya, Tri DevLiu, Hui-MingAchterberg, Wilco P.Liu, JiaAchtziger, Anja KarinLiu, JianlinAckermann, SandraLiu, JianyeAckermann-Liebrich, UrsulaLiu, JiaweiAcosta, JulioLiu, JinAcosta, Lisiane Morelia WeideLiu, JingfangAcri, GiuseppeLiu, JingwenAcuna, EdgarLiu, JinlinÁdám, BalázsLiu, Jui-MingAdam, GeorgeLiu, KaiAdam, RaziaLiu, Li-FanAdam, VerrenderLiu, LihuaAdamcová, DanaLiu, LingboAdamczewska, KatarzynaLiu, LongAdamczuk Oliveira, GilsonLiu, LuoAdamczyk, JoannaLiu, MingAdamiak, CzesławLiu, MinhuiAdamiec, EwaLiu, NanaAdamowicz, MieczysławLiu, Pearl PeiAdan, AnaLiu, PeideAdarkwah-Yiadom, Charles ChristianLiu, PengAddison, CliftonLiu, PengfeiAdebo, OluwafemiLiu, Shing-HwaAdégbitè, Bayodé RoméoLiu, Sung-TaAdegboye, OyelolaLiu, SuningAdekoya, Timothy O.Liu, Sze Y.Adekunle, AdeshinaLiu, TaihongAdekunle, Timothy O.Liu, TammyAdeola, Abiodun MorakinyoLiu, TaoAdeyinka, Adewuyi AyodeleLiu, TiezhongAdhikari, RajanLiu, WanrongAdhikari, ShankarLiu, WeiAdi, Santoso Cornelia MelindaLiu, WeiboAdibi, SasanLiu, Wei-RongAdil, Syed FarooqLiu, WeishuAdina, Turcu-StiolicaLiu, WenlongAdini, BruriaLiu, WenpingAdisesh, AnilLiu, XiaodongAdivar, BurcuLiu, XiaoleiAdjepong, DennisLiu, XiaoliAdler, AstaLiu, XiaoqiangAdler, Gregory H.Liu, XiaoweiAdler, Peter H.Liu, XiaoxiAdliene, DianaLiu, XinAdnan, SarfarazLiu, XingpengAdolfsson, AnnsofieLiu, XinhuiAdom, TheodosiaLiu, XintaoAdonakis, GeorgiosLiu, XiuyunAdriani, AlessandroLiu, XiyongAdsuar Sala, José CarmeloLiu, YanAdsuar, José CarmeloLiu, Yan-AnAdu, PrinceLiu, YangAdu-Boateng, AfuaLiu, YaqunAdusei-Asante, KwadwoLiu, YiAe, RyusukeLiu, YingAeddula, Narothama ReddyLiu, YonggangAfanou, Anani K.Liu, YouchengAfenyo, MawuliLiu, YuAffonso, HelvioLiu, YunAfionis, StavrosLiu, Ze-huaAfonso Neves, JoséLiu, ZhentaoAfonso, Andrea Luísa FernandesLiu, ZhongweiAfonso, CláudiaLiu, ZhuomingAfrashtehfar, KelvinLiu, ZongweiAfroozeh, KarimLiutsko, LiudmilaAfshar-Mohajer, NimaLivanou, MariaAgakidis, CharalamposLivazovic, GoranAgarwal, Manyoo A.Lizarzaburu, EdmundoAgarwal, PoonamLizcano Casas, DavidAgaton, CasperLizio, GiuseppeAgbaedeng, Thomas A.Lizot, MauroAgbangla, NounagnonLlamas-Alonso, LuisAggarwal, GeetikaLlanes, CarlosAggarwal, MeghaLlaneza-Suarez, DavidAggarwal, ShrutiLlata ​​García, José RamónAggeli, ConstantinaLlauradó, ElisabetAghasian, ErfanLlena, CarmenAgnew-Francis, KylieLlinás, Julia GilAgnusdei, Giulio PaoloLlopis-González, AgustínAgo, Expédit EvaristeLlorca-Jaña, ManuelAgostinelli, SimoneLlorens Gumbau, SusanaAgostini, AlexandraLlorent, Vicente J.Agranovski, IgorLlorente Cantarero, Francisco JesusAgrawal, PriyankaLloret, AntonioAgrimi, GennaroLloret, DanielAgriopoulou, SofiaLlovet Rodriguez, CarmenAgrizzi, DilaLloyd, JoanneAguaded-Ramírez, EvaLo Feudo, TeresaAguado, TxetxuLo Giudice, GiuseppeAguayo, Beatriz BerriosLo Giudice, RobertoAguayo, Luis ValeroLo Kit Mei, JammieAgudo Romeo, María Del MarLo Pardo, DanteAguiar, MarcusLo Scalzo, RobertoAguilar, Miguel PicLo Storto, CorradoAguilar-González, AbielLo, Brian K.Aguilar-Mediavilla, EvaLo, Camilla Kin MingAguilar-Parra, José M.Lo, ChristopherAguilera Manrrique, GabrielLo, Kwong Fai AndrewAguirre, AlonsoLo, Li-WeiAgulheiro Santos, Ana CristinaLo, On-Yee AmyAgulló-Tomás, EstebanLo, Shyh-ChyiAgusa, TetsuroLo, Wei-ShuoAgustin-Panadero, RubenLoas, GwénoléAhamad, MazbahulLobato, Daniel Ferreira MoreiraAhen, FrederickLobbes, HervéAhern, HollyLober, AngelaAhluwalia, Indu B.Lobo, AntónioAhluwalia, MeenakshiLocatelli, VittorioAhmad, AakashLochbaum, MarcAhmad, FayyazLockey, AndrewAhmad, HafizLockie, DamienAhmad, MianLockie, RobertAhmad, MunirLococo, Peter M.Ahmad, RaufLocquet, MédéaAhmad, TanveerLodi, ErnestoAhmed, FaheemuddinLoebach, JanetAhmed, FawadLoellgen, HerbertAhmed, Mohamed Lemine Cheikh BrahimLoenneke, JeremyAhmed, MohiuddinLoewen, PeterAhmed, Sameera T.Loewy, ZviAhmedien, DiaaLoftus, StephenAhn, HosangLog, TorgrimAhn, Hye YoungLogan, Lauren H.Ahn, JooeunLogan-Sprenger, Heather M.Ahn, JunLoh, Ping YeapAhn, Ki OkLoh, VenursAhn, Young-jooLohela-Karlsson, MalinAhrens, Richard A.Loi, FedericaAhsan, Md. ArifulLoi, Samantha M.Aibar-Almazán, AgustínLoia, FrancescaAich, AnupamLoizzo, JamieAich, SatyabrataLöllgen, HerbertAidoo, AnthonyLombamo, Getahun ErsinoAiello, EmiliaLombard, Melissa A.Aiello, OrazioLombardi, LuciaAijaz, SaimaLombardi, MarcoAik, JoelLombardi, TeresaAikins, DeaneLombardi, TommasoAiroldi, ChiaraLombardo, GiorgioAisa, RosaLombardo, MauroAitsebaomo, A. PhilipLombart, CindyAittasalo, MinnaLomonaco, TommasoAjmal, TahminaLončar Brzak, BožanaAjmol, AliLondon, JonathanAkamani, KofiLondon, WendyAkbar, Poeti Nazura GulfiraLoney, TomAkerman, AshleyLong, AudreyAkers, TimothyLong, Chiau MingAkhmetzhanov, Andrei R.Long, ManhaiAkhtar, YasminLong, Michael A.Akhter, RifatLong, QingqiAkifusa, SumioLong, TaoAkkari, AbdeljalilLongo, AntonioAkkermans, RinieLongo, StefanoAkleman, DeryaLongstaffe, James G.Akmal, AdeelŁoniewski, IgorAkombi, Blessing J.Lonkwic, PawełAkombi-Inyang, BlessingLonsky, JakubAkpinar, AbdullahLoo, Xiu Ling EvelynAkram, MuhammadLoomans, MarcelAkram, UmairLoomis, DanaAkratos, Christos S.Loos, EugèneAksha, SanamŁopaciuk-Gonczaryk, BeataAktamkhanov, AkmalLopes Teixeira, GersonAkushevich, Igor V.Lopes, Alberto Jorge OliveiraAl Souki, KarimLopes, Catia MilenaAlabdullah, JihadLopes, HélderAlaimo, AlessandroLopes, Rui JorgeAlam, Md KausarLopes, TeresaAlam, Md KhairulLopes-Alonso, MoramayAlam, NeeloyLopez Carreno, RosanaAlam, SaminaLópez De Leon, Luis R.Alamo, TeodoroLopez De Rego, CruzAlarcão, VioletaLópez Entrambasaguas, Olga MariaAlavi, Seyed EbrahimLópez Martínez, Alicia EvaAlbaladejo-Blázquez, NataliaLopez Mora, ClaraAlbano, MarcoLopez Navas, Ana I.Albarrán, PedroLópez Sánchez, Guillermo FelipeAlbendín-García, LuisLopez, AntonellaAlbert, AlexLópez, DanielAlbert, JeffLópez, Daniel LópezAlbert, NienhausLopez, FrancescoAlbert, Ryan J.Lopez, JavierAlberta, LuccheseLópez, Jesús AdriánAlberti, SaverioLópez, Jose A.Albertin, GiovannaLopez, Jose JavierAlbino, Barraza VillarrealLópez, José LuisAlbornoz-Cabello, ManuelLopez, LucreziaAlbright, Michaeline B. N.López, MercedesAlbuquerque, AlexandraLopez, Michele AntonioAlcaide, Juan IgnacioLopez, NanetteAlcañiz, ManuelaLopez, OliviaAlcantara, Luiz Carlos JuniorLopez, StellaAlcántara-Ortigoza, Miguel AngelLopez, VelmaAlcaraz Carrillo De Albornoz, VicenteLópez-Alberca, Cristina RomeroAlcaraz, MiguelLópez-Berlanga, María del CarmenAlcaraz-Ibáñez, ManuelLópez-Borrull, AlexandreAlcudia Aguilar, AlejandroLópez-Cabrales, AlvaroAldieri, LuigiLópez-Camarillo, CésarAldous, ColleenLópez-Castro, LeticiaAldrich, Melissa B.López-Cortegano, EugenioAleffi, ChiaraLópez-Ejeda, NoemíAlegria-Moran, RaulLopez-Escamez, Jose A.Aleixo, Ana MartaLópez-Escribano, CarmenAlejo, AlíLópez-Fernandez, IvánAlejziak, WiesławLópez-Fernández, JorgeAleksandra, RogowskaLopez-Garcia, RicardoAleksandrovych, VeronikaLópez-García, SergioAlekseenko, AlexeyLópez-Gil, José FranciscoAleksić, AnaLópez-Gutiérrez, CarlosAleksova, AnetaLópez-Gutiérrez, Carlos JavierAlemany, SilviaLópez-Huertas, María RosaAlencar, Airlane PereiraLopez-Jornet, PiaAleo, GiuseppeLopez-Labrador, Francesc-XavierAletta, FrancescoLopez-Liria, RemediosAlexander Bizjak, DanielLópez-López, DanielAlexander, DavidLópez-López, LauraAlexander, KellyLópez-Martínez, AlbertoAlexander-Passe, NeilLópez-Martínez, Jorge O.Alexandre, FellousLópez-Medina, Isabel MaríaAlexandropoulou, IoannaLópez-Morales, VirgilioAlexeeff, StaceyLópez-Navarro, Miguel ÁngelAlfageme-García, PilarLópez-Núñez, CarlaAlfano, GaetanoLópez-Núñez, M. InmaculadaAlfano, MarcoLopez-Nuñez, RafaelAl-Faris, Nora A.López-Pascual, JoaquínAlfaro-Pérez, JorgeLópez-Pintor Muñoz, Rosa MaríaAlfieri, AndreinaLópez-Pintor, ElsaAlfonsi, ValentinaLópez-Sáez, Juan BoscoAlfonso Piña, William H.Lopez-Samanes, AlvaroAlfonso, Gago-CalderónLópez-Sánchez, YaizaAlfranca, OscarLopez-Santos, OswaldoÄlga, AndreasLópez-Toro, AlbertoAl-Ghabkari, AbdulhameedLopez-Villegas, AntonioAlgranti, EduardoLópez-Zafra, EstherAlhenc-Gélas, FrancoisLoppi, StefanoAl-Hilaly, YoussraLoppi, SteffanoAli, AimanLopreite, MilenaAli, AmanatLoprevite, SalvatoreAli, G. G. Md. NawazLorber, MatejaAli, GhaffarLord, RichardAli, Hayssam M.Lorenzetti, SilvioAli, Lamiaa M. A.Lorenzini, JasmineAli, SarvathLorenzo Calvo, JorgeAli, SharafatLorenzo, AlbertoAliaño González, María JoséLorenzo-Gómez, Maria-FernandaAlif, Sheikh MohammadLorenzo-López, LauraAl-Imam, AhmedLorenzoni, GiuliaAline, Silva-CostaLoreti, PierpaoloAlinejad-Rokny, HamidLőrincz, Ákos M.Alińska, AgnieszkaLorini, ChiaraAlipour, AzitaLorussi, FedericoAliu, ArmandoLorusso, FeliceAlizadeh, BabakLorusso, VitoAlizargar, JavadLoscalzo, YuraAljaaf, AhmedLoster, Jolanta ElżbietaAljaroudi, AliredaLosurdo, GiuseppeAl-Jawaldeh, AyoubLotko, AleksanderAl-Jumeily, DhiyaLottering, RomanoAlkaher, IrisLouie, LoboAlkan, MichaelLoupa, GlykeriaAlkhachroum, AyhamLoureiro, Isabel MariaAl-Khazraji, BaraaLoureiro, NunoAlkhubaizi, QootLourenço, CéliaAllegaert, KarelLourenço, MarioAllegra, EugeniaLous, JørgenAllegret, Jean-PierreLove, KeithAllem, Jon-PatrickLove, PennyAllen, Antiño R.Love, TerenceAllen, CharlesLovecchio, NicolaAllen, JoanneLovero, Kathryn LynnAllen, Norman S.Lovinsky-Desir, StephanieAllgood, Sarah J.Lovisolo, LisandroAllison, Paul J.Lovrecic, MercedesAlloh, FolashadeLovrić, NatašaAlmansa, JosuéLovro, ŠtefanAlmashhadani, ShiamaaLow, Banus Kam LeungAlmberg, Kirsten StaggsLow, David A.Almeida, FernandoLowden, ArneAlmeida, RuteLowe, MatthiasAlmeida, SaraLower, TonyAlmendra, RicardoLöytönen, MarkkuAlmendral, Violeta RuizLozano Blasco, RaquelAlmendra-Pegueros, RafaelLozano, DemetrioAlmendro-Candel, María BelénLozano, SusanaAlmendros, CarmenLozano-Baez, Sergio E.Almerich-Silla, José ManuelLozano-Díaz, AntoniaAlmerich-Torres, TeresaLozano-Paniagua, DavidAlmiron, NúriaLu, ChaoAlmond, HelenLu, Cheng-HsuanAlmoosawi, SuzanaLu, ChenshengAlmoshmosh, NadimLu, Chi-JieAlmståhl, AnnicaLu, ConnieAl-Muqdadi, Sameh W.Lu, HangzhouAl-Obaidi, KaramLu, Jeng-WeiAloisi, IrisLu, Jia-haiAlola, AndrewLu, JiahuiAlonso, CarolinaLu, JianzhongAlonso, FranciscoLu, Ming-WeiAlonso, MartaLu, NanAlonso, Miguel A.Lu, Tsung-ChienAlonso-Ferreira, VeronicaLu, XiaojunAlonso-Garcia, SantiagoLu, XiaoxiaoAlonso-Muñoz, LauraLu, YujiaoAlós-Simó, LiriosLu, YunAlpay Savasan, ZeynepLu, ZhenboAlperovitch-Najenson, DeborahLu, ZhipengAl-qaness, Mohammed A. A.Lubas, ArkadiuszAlrahaili, MusaadŁubianka, BeataAl-Rudainy, DhelalLubkowska, AnnaAlsaggaf, RotanaLubowiecki-Vikuk, AdrianAlsalloum, AtaaLuca, AndreiAl-Shammari, MarwanLuca, FalzoneAlshannaq, AhmadLuca, MariaAlsharairi, NaserLucaccioni, LauraAl-Shibaany, ZeyadLucarini, MassimoAlsop, RogerLucas, GuilhermeAlstete, Jeffrey W.Lucas, RobynAlston Taylor, Sharonda J.Lucas, StevenAlsufyani, NouraLucas, TimAltenburg, TeatskeLucay, Freddy A.Alteren, JohanneLucchese, AlessandraAltieri, FedericaLucci, TâniaAltman, MicahLucena-Anton, DavidAltmann, StefanLucero, JessAlto, Barry W.Lucero, JulieAlvarado, Jesús M. Luchini, ClaudioAlvarado-Lassman, AlejandroLucille, AlonsoAlvarado-Lorenzo, AlfonsoLucini, DanielaAlvarado-Lorenzo, MarioLückmann, Sara LenaAlvarez, GuzmánLucotti, SerenaÁlvarez, Joaquín F.Łuczyński, WłodzimierzAlvarez, OctavioLudeke, SörenÁlvarez-Albelo, CarmenLudlow, KristianaÁlvarez-Ayuso, EstherLudwig, OliverÁlvarez-García, DavidLudwikowski, BarbaraÁlvarez-García, JoséLudy, Mary-JonÁlvarez-Mercado, Ana IsabelLuedeling, EikeAlvarez-Mon, Miguel AngelLuesma, María JoséAlvarez-Narvaez, SonsirayLuevano-Contreras, ClaudiaAlvarez-Peregrina, CristinaLugg-Widger, FionaÁlvarez-Serrano, María AdelaidaLugo Ocando, JairoÁlvaro-Estramiana, José LuisLugo-Morin, Diosey RamonAlvero Cruz, Jose RamonLuh, Yir-HueihAlves Vas, Francisco JavierLühken, RenkeAlves, JoanaLui, Ka HeiAlves, Juliana AraújoLuig, MelissaAlves, NiltonLuigi Invernizzi, PietroAlves, SandraLuigi, CucciAlves, SheilaLuigi, LavorgnaAlves, SusanaLuino, FabioAlves, WellingtonLuis Ubago, JoseAlves-Guerreiro, JoseLuis, AnaAlvim-Pereira, FabianoLuís, ÂngeloAlwarea, AmerLuis, ElkinAlwerdt, JessieLuis-De Cos, IzaskunAly, KarimLuiz, Alfredo José BarretoAlyass, AkramLuján-Álvarez, ConcepcionAlzghoul, Manal M.Luján-Mora, SergioAmado Alonso, DianaLukáč, PavelAmado, MiguelLukács, AndreaAmado, SandraLukacz, Emily SpencerAmadora Pomar, CatalinaLukaszewska-Kuska, MagdalenaAmadori, FrancescaLukkahatai, NadaAmaechi, Chiemela VictorLukomska-Szymanska, MonikaAmalfitano, StefanoŁukowski, AdrianAman, M. JavadLukyanov, Pavel A.Amancio, DiegoLumbreras Lacarra, BlancaAmankwaah, AkuaLuna Maldonado, AurelioAmann, AtxuLuna, Aderval S.Amaradhi, RadhikaLund, Rolf LyneborgAmarakoon, UpamaliLund, SandraAmaral, Teodosio PerezLundberg, StefanAmarasena, NajithLundberg, Ulf L.Amarasiri, MohanLundeheim, NilsAmarelli, CristianoLundman, BeritAmăricăi, Elena ConstantaLundqvist, P.Ambati, AdityaLunghi, CarlottaAmbros, JiříLungu, Daniel AdrianAmbrosi, ElisaLunov, VitaliiAmbrósio, VitorLunsford-Avery, Jessica R.Ambroszkiewicz, JadwigaLunt, Neil T.Ambrozy, TadeuszLuo, BinAmedei, AmedeoLuo, HanqiAmendola, AlessandraLuo, HongliangAmerio, AndreaLuo, HuabinAmey, Foster K.Luo, JunAmezcua-Prieto, CarmenLuo, LiangAmialchuk, AliaksandrLuo, RenfuAmicarelli, VeraLuo, WentaiAmideo, Annunziata EspositoLuo, XiaoAmin, Nirav H.Luo, XiaohuaAmini, HereshLuo, Xiao-SanAmini, HosseinLuo, Yi (China)Aminoroayaie Yamini, OmidLuo, Yi (USA)Amitai, MayaLuo, YunjuanAmmar, AchrafLuo, ZhenAmmer, KurtLuong, HaiAmmitzboll, GunnLuongo, FabriziaAmnie, AsratLuongo, GiuseppeAmoah-Ramey, NanaLuparell, SusanAmoaku, Winfried M.Lupi, Saturnino MarcoAmodeo, Anna LisaLupo, Karen D.Amor Almedina, María IsabelLupp, GerdAmor González, Antonio ManuelLupu, LucianAmoriello, TizianaLuque Moreno, CarlosAmorim, GustavoLuque-Reca, OctavioAmorim-Costa, CéliaLuquin, Mª RosarioAmornyotin, SomchaiŁuszczek-Trojnar, EwaAmpollini, LucaŁuszczki, EdytaAmpon-Wireko, SabinaLuthra, PriyaAn, ByungryulLutkiewicz, KarolinaAn, ChunjiangLutomia, Anne NamatsiAn, Jeung HeeLutski, MiriamAn, MinjeongLuttenberger, Lidija RunkoAn, YuLutter, ChristophAna, AnaLutter, ErikaAnaby, DanaLützenkirchen, JohannesAnache, Jamil A. A.Luukkonen, TeroAnafi, PatriciaLuviano-Cruz, DavidAnand, JanetLuzardo-Ocampo, IvanAnand, KartikLuzzani, GloriaAnand, SwatiLv, HuixiongAnas, Andrea Roxanne J.Lv, JunAnastasi, EmanuelaLv, ShenghuaAnastasiou, EvgeniaLwin, Ko KoAnastasiu, LiviaLwin, KristenAnastassiadou, VassilikiLydahl, DorisAncel, JulienLydon-Staley, David M.Ancione, GiuseppaLynch, Caroline AnitaAndaluri, GangadharLynch, HeidiAnderberg, PeterLynch, KrystalAndereggen, LukasLynch, PaulAnderko, LauraLynge, ElsebethAndersen, David F.Lyon-Callo, VincentAndersen, VidarLyons, GrahamAnderson, AlexaLyons-Weiler, JamesAnderson, AnneLystad, Reidar P.Anderson, CraigŁyszczarz, BłażejAnderson, Darcy M.Lyu, QuanjunAnderson, David A.Lyu, TaoAnderson, Gary A.Lyubushin, AlexeyAnderson, JoelLyver, Maurice J.Anderson, KateLyytimäki, JariAnderson, PeterM. Dolores, GallegoAnderson, Philip O.M. Harandi, VahidAndersson Hammar, IsabelleM. Silva, FernandaAndersson, MagnusM. Weinstein, GeraldineAndina Díaz, ElenaM’Baye, BabacarAndjelkovic, MirjanaM’kacher, RadhiaAndo, TakafumiMa, ChangxiAndolina, Jeffrey R.Ma, Ching-To AlbertAndrade Cetto, AdolfoMA, DingAndrade, André Luiz MoneziMa, EnboAndrade, Jeanette M.Ma, GuanshengAndrade, MaríaMa, HongAndrade, MariliaMa, HongqinAndrade, Vanda MariaMa, HuiAndráš, PeterMa, JennyAndrasik, MicheleMa, JiaAndré, Leiliane CoelhoMa, JiangyaAndreini, Bianca PatriziaMa, Jie (China)Andreo, VeronicaMa, Jie (UK)Andreolli, MarcoMa, LaiAndreou, MariaMa, LiangsuoAndrés, José Manuel PalaoMa, LuAndrés, María T.Ma, MingguoAndresen, BjörnMa, PingAndrés-Villas, MontserratMa, SihuiAndretta, MassimoMa, WeiAndreucci, Maria BeatriceMa, XinAndreu-Sánchez, CeliaMa, XuanAndrews, AnyaMa, YongfengAndrews, NaomiMa, YuxiaAndriessen, KarlMa, Zheng FeeiAndrikopoulos, GeorgeMa, ZihuiAndriolo, JessicaMaalouf, WadihAndrius, PranskunasMäättä, SuviAndroniki (Nina), PapafilippakiMäät tä nen, Il ma riAndronikos, GeorgeMabasa, LawrenceAndrzej, NowickiMabhala, Mzwandile AndiAnelli, DeboraMabhaudhi, TafadzwanasheAnesi, AlexandreMáca, VojtěchAng, Eng TatMacalister, HeatherAngel, BárbaraMacaluso, FilippoAngeles Macambira, Simone GarciaAngelici, Maria CristinaMacaskill, AnnAngelillo, Italo FrancescoMacchia, Paolo Emidio MidioAngelini, FedericoMacchioni, ElenaAngelos, HatzakisMacChioni, PierluigiAngelova, Radostina A.Macdonald, BenAngeoletto, Fábio Henrique SoaresMacDonald, DouglasAngers, Jean-FrançoisMacdonald, LauraAngosto Sánchez, SalvadorMacdonald, Marey AllysonAnguah, Katherene O.-B.Mace, Robert E.Anguera, María TeresaMacedo, EloisaAnido Rifón, Luis E.Macedo, João CarlosAnifandis, GeorgeMacgregor, AishaAnikó, CsecseritsMacGregor, Barbara J.Anis, EmanMacGregor, CasimirAnishchenko, LesyaMacGregor, Lewis J.Anjum, AmnaMachado Reis, VictorAnkawi, BrettMachado Sanchez, HugoAnna Poulia, KalliopiMachado, Alexander MazAnnarapu, Gowtham K.Machado, AnaAnnesini, Maria CristinaMachado, CarolinaAnnibale, BrunoMachado, Christiano BittencourtAnnibalini, GiosuèMachado, JandysonAnnunziata, GiuseppeMachado, MariaAnsa, BenjaminMachado, Mariana V.Ansani, LuciaMache, StefanieAnsari, Mohammad AzamMachida, DaisukeAnsmann, LenaMachimbarrena, Juan M.Ansoleaga, ElisaMachová, KristýnaAnstead, Gregory M.Machoy, Monika ElżbietaAntal, OanaMaciá, Carolina GonzálvezAntczak, ElzbietaMacías Aranda, FernandoAntia, KhatiaMacías Seda, JuanaAntin, Jonathan FrankMacias, Rocio I. RodriguezAntinienė, DaliaMacias-Diaz, Jorge E.Antinozzi, CristinaMaciaszek, JanuszAntipova, VeronicaMaciejczak, MariuszAntoine-Jonville, SophieMaciejczyk, MarcinAntona, Carmen JiménezMaciejczyk, MateuszAntonacci, YuriMaciejczyk, PolinaAntonaci, FabioMaciejewski, GrzegorzAntoñanzas, FernandoMaciel, Carlos DiasAntonarakis, Gregory S.Macijauskienė, JūratėAntonelli, AlbertoMaciukiewicz, MalgorzataAntonelli, MicheleMacKay, DianaAntoniewska, AgataMackay, SallyAntonín Martín, MontserratMacken, LieveAntonio, López-VillegasMackenbach, Joreintje DingenaAntonio, Mapuana C. K.Mackenzie, RowanAntoniou, EvangeliaMackey, Thomas P.Antoniou, PanosMackiewicz, DorotaAntonkiewicz, JacekMackiewicz-Milewska, MagdalenaAntonogeorgos, GeorgeMackinnon, AllanAntonoglou, Georgios N.Mackowicz, JolantaAntonopoulou, ViviMaclaren, DavidAntonschmidt, HannesMacLean, Oscar A.Antón-Solanas, IsabelMaclellan, Reid A.Antosiewicz, JędrzejMacMillan, FreyaAnttila, MinnaMacNamara, MaureenAntunes, Celia M.Macori, GuerrinoAntunes, Francisco José NunesMacuda, MałgorzataAntunes, RaulMaculewicz, EwelinaAntúnez, AntonioMacVicar, SonyaAnuj, TiwariMączka, WandaAnunciação, Pedro FernandesMadalina, ArmieAnund, AnnaMadaline, TheresaAnvar, Amir M.Madan, PavanAnver, ShajahanMadarame, HaruhikoAnwary, Arif RezaMadariaga, AuroraAnyfantakis, Dimitrios I.Madau, Fabio A.Anyżewska, AnnaMaddalone, MarcelloAoki, MasayoMadden, Diana LynneAono, JunMadden, KennethAparecida Silveira, ErikaMaddipati, VeerannaAparicio, Maria RubioMaddock, JaneAparicio, SebastianMaddox, RaglanAparicio-Herguedas, José LuisMadej, DawidAparicio-Martinez, PilarMader, SebastianAparisi, DavidMadewell, CharlesApergis, NikolaosMadhurapantula, Rama SashankApers, LudwigMadill, CateApollo, MichalMadinabeitia, IkerApolzan, John W.Madra, AnnaApóstolo, JoãoMadrid, AlejandroApostolopoulos, NikosMadruga, MiguelAppell Coriolano, Hans-JoachimMadsen, Dag ØivindAppell, MichaelMadureira, TeresaAppelqvist, KaijaMaeda, KokiAppendini, ChristianMaeda, YuichiAppiah-Hagan, EmmnauelMaehre, HanneAppleton, KatherineMaekawa, RyoAppolloni, LetiziaMaekawa, ShinyaApter, AlanMaestrelli, PieroAquino-Martinez, RubenMaestú Unturbe, CeferinoAra, AndersonMaffettone, RobertaAracil-Marco, AdolfoMaffioli, Elisa M.Arafah, KarimMaftei, AlexandraAragón Vela, JerónimoMagafas, LykourgosAragon, JanniMagalhães Filho, Fernando J. C.Arai, ShojiMagalhães, BrunoArain, AubreyMagalhães, Fernando HenriqueAramendia, IñigoMagalhaes, Flavio De CastroAran, VeronicaMagalhaes, TerezaArana-Chavez, VictorMagallón Neri, ErnestoAranaz, AliciaMagan-Fernandez, AntonioAranda Balboa, María JesúsMagarati, MayaArango-Miranda, RaúlMagasi, SusanAranha, Ágata MarquesMagazzino, CosimoAranha, AnilMaggos, ThomasArasli, HuseyinMagiera, TadeuszArason, NeilMagini, RobertoArat, GizemMagiorkinis, EmmanouilAraújo, Allysson AllexMagit, AnthonyAraujo, LiaMagli, FrancescaAraújo, NoeliaMagliano, DiannaAraujo, RosaMaglione, MicheleAraújo, Thaíse GonçalvesMagliulo, GiuseppeArbabi, VahidMagnani, CorradoArbel, ReoutMagnano San Lio, RobertaArbo, IngeridMagnano, PaolaArcangeli, GiulioMagnavita, NicolaArce-Fernández, ConstantinoMagne, CyrilleArce-Ruiz, RosaMagnone, DanielArcher, DavidMagori, KrisztianArcher, NormMagriplis, EmmanuellaArchontakis, FragiskosMaguire-Jack, Kathryn L.Arcidiacono, CaterinaMahajan, HarshalArcila Calderón, CarlosMahajan, SachitArciniega, Alejandra BenitezMahalingaiah, ShruthiArcos González, PedroMahan, MichaelArcos Romero, Ana IsabelMahapatra, Parth SarathiArcurio, Lindsay R.Maharaj, SonillArcury, Thomas A.Mahdavipour, OmidArdalan, MaryamMaheu-Giroux, MathieuArdigò, Luca PaoloMähler, AnjaArdoy-Cuadros, JuanMahlert, BettinaArena, AlessandroMahmood, AsifArenas-Ramírez, BlancaMahmood, TaufiqueArensberg, Mary BethMahmoud, AhmedArévalo-Baeza, MartaMahmoud, Alaa El DinArgalasova, LubicaMahmoud, MedhatArgenziano, PasqualeMahmud, BasriArgilaga, AlbertMahmud, IqbalArgyropoulos, ChristosMahmud, Khan M.Arias Buría, Jose LuisMahmuda, Naila AlArias Ramos, NataliaMahoney, Sara E.Arias, Alexander GilMaibach, EdwardArias, Ana VictoriaMaiden, NeilArias, AndresMaier, DorinArias, Dulce MariaMaietta Latessa, PasqualinoArias-Santiago, SalvadorMaihami, RezaArici, CeciliaMailey, EmilyArif, Ali TalibMain, Luana C.Ariga, KatsuhikoMainar, AlfredoArima, SerenaMaindal, Helle TerkildsenArion, FelixMainero Rocca, LuciaAris, Izzuddin M.Mainka, AnnaArís, NuriaMaiorano, TizianaAristizábal Becerra, Luz AdrianaMair, JacquelineAristondo, OihanaMair, JudithAritio-Solana, RebecaMaisha, BuumaAriyoshi, WataruMaisonet, MildredArjamaa, OlliMaiuolo, JessicaArmah, FrederickMajaranta, PäiviArmah, Seth M.Majeed, BanArmand, AlbergelMajeed, HaroonArmaou, MariaMajek, PaulinaArmindo Melo, ArmindoMajewski, JanuszArmstrong, AndreaMajewski, SebastianArmstrong, HelenMajic, SamanthaArmstrong, PaulMajid, ShaheenArmstrong, StuartMajolino, DomenicoArmstrong, ThomasMajorin, FionaArmstrong-Mensah, ElizabethMajumdar, IndrajitArnarsson, ArsaellMajumder, AvisekArnaud, GouelleMajumder, SudipArnbjörnsson, EinarMak, Hugo Wai LeungArndt, Patrick G.Makarem, NourArnedo-Pena, AlbertoMakarewicz, CarrieArnold, Bruce BaerMakarova, IrinaAronsson, Carin AndrénMakarska-Bialokoz, MagdalenaArooj, FatimaMakena, Monish RamArora, AmandeepMaki, JamesArora, AmitMaki, KevinArora, HimanshuMaki, YohkoAroso, CarlosMakieła, ZbigniewArrabito, Giuseppe DomenicoMakker, HimenderArrandale, VictoriaMakore, Busisiwe Chikomborero NcubeArregui, MariaMakoś, PatrycjaArribas Galarraga, SilviaMakowiec-Dąbrowska, TeresaArribas Marín, Juan ManuelMakra, LászlóArrieta-Baez, DanielMakrakis, SergioArrogante, ÓscarMakris, IliasArroyo-Figueroa, GustavoMaksimov, SergeyArsoniadis, Gavriil G.Maksymiuk, GabrielaArteaga Herrera, OscarMakunga, Nokwanda P.Artese, AshleyMalaguarnera, MicheleArtiemjew, PiotrMalandrino, MeryArtime, MichaelMalara, JarosławArtola, AdrianaMalara, MarzenaArturo Vera Ponce De Leon, ArturoMalarvannan, GovindanArturo, Figueroa MontañoMalatzky, ChristinaArufe-Giráldez, VíctorMaldonado, AideAsa’ad, FarahMaldonado, Ana IsabelAsakura, TakashiMaldonado-Valderrama, JuliaAsare, MattMalek, LukaszAsbrand, JuliaMalekigorji, MaryamAschenbrenner, SteffenMaletskyi, ZakharAscheri, JoseMalewski, TadeuszAşchilean, IoanMalhotra, Davinder K.Aschonitis, Vassilis GeorgeMalhotra, JodieAsci, SerhatMali, MatildaAscoli Marchetti, AndreaMalicka, IwonaAseervatham, JayaMalik, Asif IqbalAsencios, Yvan J. O.Malik, KrzysztofAsere, Tsegaye GirmaMalik, MarekAsfaw, AbayMalík, PeterAsghar, ZahidMalinauskas, RomualdasAshbrook, Aaron R.Malinauskiene, VilijaAshley, DavidMalinen, MikkoAshley-Martin, JillianMalings, CarlAshmore, RussellMalinis, MaricarAshok, MonaMalinowska, Anna M.Ashour, HossamMalinowska-Cieślik, MartaAshour, Mohamed-BassemMalinowski, MateuszAshraf, Badar NadeemMalińska, KrystynaAsiama, KwabenaMalinzi, JosephAsian Clemente, Jose AntonioMall, ChristophAsif, MuhammadMallah, NarmeenAsif, Muhammad B.Mallakpour, ImanAsiki, GershimMallamaci, RosannaAsim, MuhammadMallela, Shamroop KumarAsimakopoulou, EleniMallen, EvanAsimina, StamatelopoulouMallet, Marc DanielAsk, Lina SchollinMallet, Marie L.Askew, DeborahMallett, CliffAslanidis, TheodorosMallett, JohnAsnaani, AnuMalli Mohan, Ganesh BabuAspinall, Peter J.Malli, FoteiniAspriello, Simone DomenicoMalliaropoylos, NikosAsquith, Christopher R. M.Mallineni, Sreekanth KumarAssandri, FaustoMallinson, Daniel J.Assari, ShervinMallinson-Howard, SarahAssefi, MohammadMallow, OleAssi, SulafMalmaeus, Jan MikaelAssirelli, AlbertoMalmqvist, EbbaAstasio Picado, ÁlvaroMaloberti, AlessandroAstill, GregoryMałodobra-Mazur, MałgorzataAstorino, ToddMaloney, Thomas N.Astrin, KennethMalta, MonicaAsumadu-Sarkodie, SamuelMaltais, MathieuAswi, AswiMaltman, ChrisAtabekova, AnastasiaMaltry, LauraAtapattu, SumuduMaltseva, ArinaAthinarayanan, ShaminieMaluccio, John A.Athyros, VasiliosMalvezzi, MatteoAtique, UsmanMalwal, Satish R.Atkins, Anthony S.Mamelund, Svenn-ErikAtkins, PennyMamen, AsgeirAtkins, RahshidaMamì, AntonellaAtkinson, Anthony C.Mamiit, Rusyan JillAtkinson, Christopher L.Mammadova-Bach, ElminaAtkinson, SamMamouni, KenzaAtkinson, StephanieMamudu, Hadii M.Atobe, MasakazuMan, AdrianAtroszko, Paweł AndrzejMan, Siu ShingAtsumi, ToshiyukiManai, FedericoAtsushi, ImaiManakil, JaneAttena, FrancescoMañas, AsierAtti, Anna RitaManca, StefaniaAttia, SamehMancardi, DanieleAttias, JosephMancinelli, ElisaAttique, MuhammadMancini, SimoneAttuquayefio, TukiMancl, Lloyd A.Attwell, KatieManda, SamuelAtukeren, ErdalMandal, AmritlalAtwood, CraigMandal, ChanchalAu, DoreenMandal, SubhamoyAuad, Lígia IsoniMandato, ClaudiaAuch, RogerMandel, SumitAudrain-Pontevia, Anne-FrancoiseMandelbaum, DavidAuerbach, SanfordMandemakers, Jornt J.August, Keith J.Mandić-Rajčević, StefanAugustine, LillyMandini, SimonaAugustine, Swinburne A. J.Mandolesi, SerenaAugustyniak, AdrianMandric, IgorAuler, AugustoManenti, AntonioAung, Meiji SoeManess, SarahAung, Myo NyeinManess, Sarah B.Aung, Wai PhyoManfredi, SylvainAurora, RajeevManfredini, RobertoAusín Benito, BertaMang, Stefania MirelaAustin, Erin EdwardMangano, AlessandroAutier, BriceMangano, CarloAu-Yeung, SheenaMangano, GiulioAvanzo, MicheleMangano, Valentina D.Avdonin, Pavel V.Mangla, AnkitAverbeck, Nicole B.Mangold, AlexanderAvila, PauloManheim, DavidÁvila-García, ManuelMani, DibaAvino, PasqualeMânica Da Cruz, Ivana BeatriceAvishai, GalManika, GeorgiaAvloniti, AlexandraMankowska-Wierzbicka, DorotaAvraam, JoanneMann, NathanAvram, AlexandruMannan, HaiderAvtanski, DimiterMannelli, GiudittaAvtar, RamMannetje, Andrea ’tAwan, UsamaMannheim, ViktoriaAwasthi, MayankaManni, AnnikaAweeka, Francesca T.Manni, RaffaeleAwolusi, Ibukun GabrielMännikkö, NikoAwosile, BabafelaManning, ChristieAwual, Md. RabiulManning, JimmieAwuzie, BankoleMannino, DavidAxe, Lisa B.Mannino, GiuseppeAxelrad, HilaMannion, CynthiaAxiotis, EvangelosMannu, AlbertoAyala, FranciscoMannucci, PierAyala-Zavala, J. FernandoManolopoulos, AnnaAyalew, Lisanework E.Manor, IrisAyán, CarlosManousakis, JessicaAyaz Arda, OzlemManousos, ManousakasAydede, Sema K.Mansell, TobyAyers, PhilMansfield, Julie A.Ayllón, EsterMansour, ChristopherAylward, LesaMansour, RamziAyonrinde, OyekoyaMånsson, AlfAyuso, Juan MielgoManta, FrancescoAyuso-Montero, RaúlManta, OtiliaAzab, WalidMantecchini, LucaAzadi, MehdiMantilla Herrera, Ana MariaAzamathulla, H. MohammadMantovani, AlbertoAzañedo, Carolina M.Mantzaris, Alexander V.Azara, AntonioMantziari, AnastasiaAzeez, AdeboyeMantzoukas, StefanosAzevedo Guedes, André LuisManuel Gonzalez-Chorda, VictorAzevedo, AndreiaManuel, Rodríguez-HuguetAzevedo, RuiManuti, AmeliaAzhar, Gulrez ShahManyk, TetianaAzimjon, SayidovManzanares, MariaAzis, KonstantinosManzano, Carlos A.Aziz, Abdul RashidManzano-Sánchez, DavidAzman, SametManzo, CiroAzmat, MuhammadManzo-Merino, JoaquinAznar, Juan PedroMao, FeiyueAznar, MónicaMao, Gen-XiangAzpilicueta, LeyreMao, KuoRayAzuma, TakashiMao, LiangAzzimonti, BarbaraMao, LiminB. N., Pavan KumarMao, MaoBabaei, AlirezaMao, XinhuaBabalola, Micky A.Mao, YanpingBabalola, SundayMao, YingBabatunde Alayande, AbayomiMaposa, DanielBabiarczyk, BeataMaqbool, RashidBabickova, JankaMaraffi, SabinaBabiuk, ShawnMaragou, Niki C.Babore, AlessandraMarais, GabrielBabraj, JohnMaran, Daniela AcquadroBabraj, John AndreeMaranesi, MonicaBabu, DineshMarat-Mendes, TeresaBacchini, DarioMaraver, FranciscoBacci, ChristianMarc, DanielBaccolini, ValentinaMarca-Francès, GuillemBacevičienė, MiglėMarcantonio, MatteoBach, Albert PagèsMarcantonio, RichardBachagha, NabilMarcella, Windmuller-CampioneBachem, RahelMarcello Falcone, PasqualeBäcker, Henrik C.March, StefanieBackes, ToddMarchand, William R.Backstrand, Jeffrey R.Marcheggiani, StefaniaBacqué, Marie-FrédériqueMarchena Rodríguez, AnaBadagliacca, GiuseppeMarchena, CarlosBadanta Romero, BárbaraMarchese, Silvia MariaBadau, AdelaMarchesi, AlessandraBadau, DanaMarchesi, IsabellaBaddour, NatalieMarchesini, GiulioBaden, DeniseMarchi, MaurizioBadlissi, FadiMarch-McDonald, JaneBado, IgorMarcos Pardo, Pablo JorgeBadry, Dorothy EleanorMarcos-Delgado, AlbaBadulescu, AlinaMarcos-Merino, José MaríaBădulescu, DanielMarcos-Pérez, DiegoBae, Jong-MyonMarcoulides, George A.Baek, Seung JoonMarcovici, GenoBaek, SeungryulMarcu, NicuBaena-Extremera, AntonioMarcu, SilviaBaer, ThomasMarcus, MichaelBaert, ThaisMardani, AbbasBaeyens, JanMarek, AgnieszkaBaeyens, Jean-PierreMarek, ErikaBaez, YolandaMarek, LukasBaeza Moyano, DavidMarek, MaciejBaffour, Kwaku KyeiMarella, ManjulaBagasra, AnisahMarena, CarloBagetta, GiacintoMargalef, MariaBaghdadi, Ziad D.Margaritelis, NikosBagheri, ZahraMargaritis, IrèneBagiu, Iulia-CristinaMargaritis, LukasBagley, Christopher AdamMargarucci, SabrinaBaglivo, CristinaMargherita, Emanuele GabrielBagordo, FrancescoMargiotta, AzzurraBagüés, AnaMargolis, LeeBahari-Chopiuk, NasrinMargoni, FrancescoBahelah, RaedMargues, AdilsonBahji, AneesMarhavilas, PanagiotisBahmani, SaharMaria Irene, BelliniBahn, Geon HoMaria Nuzzo, AnnaBahooToroody, AhmadMaría Rosaura, Leis TrabazoBahr, KatharinaMaria, Giner-SorianoBahri, MitraMaría, Soria-OliverBai, LibiaoMariani, Marco G.Bai, ShipingMariani, RaffaelloBai, XinwenMariano, Norzagaray CamposBai, YuMaric, FilipBai, YunliMarichalar Mendia, XabierBaiano, ChiaraMarín De Evsikova, CaralinaBaidwan, Navneet KaurMarín, MaríaBaier, DirkMarina, MichelBaigent, MichaelMarín-Díaz, VerónicaBaile Ayensa, José IgnacioMarinelli, AlesssandraBailey Bisson, JenniferMarinello, FrancescoBailey, CateMarinello, SamueleBailey, TessaMarine-Roig, EstelaBaillie, LesleyMaringer, Franz JosefBaindara, PiyushMarini, Carlo FerriBainschab, MarkusMarini, Herbert RyanBaiocco, SilviaMarini, MircaBaïz, NourMarini, PaolaBajaj, PuneetMarinkovic, DraganBajaj, RuchikaMarino, BrunoBajo Lorenzana, Victoria M.Marino, DavideBajpai, RamMarino, JosephBajusz, DávidMarino, MassimilianoBak, JuseonMarinov, MarinBaker, Jason R.Marín-Pagán, CristianBaker, KaylaMario, MiniatiBaker, RichardMario, PagnoniBaker, RoseMarion, Jason W.Baker, SandraMariounneau, VirveBaker, ShayneMarjoribanks, TimBakhshian, SaharMarker, SandraBakillah, AhmedMarkou, MarinosBakker, Mieke H.Markowicz-Piasecka, MagdalenaBakolis, IoannisMarkowska-Szczupak, AgataBaksi, AnanyaMarks, Emma J.Bakupog, ThomasMarks, Michael J.Bala, MałgorzataMarks-Bielska, RenataBalague, NataliaMarkus, ArjenBalakrishna, RamachandranMarkworth, James F.Balakrishnan, BijuMarletta, LuisaBalaraman, VelmuruganMarlicz, WojciechBalasubramanian, BalamuralikrishnanMarling, GarryBalawejder, MonikaMarmo, RossellaBalcells-Balcells, AnnaMarmot, AlexiBaldassarre, AntonioMarocolo, MoacirBaldassarre, MaurizioMaroney, Megan E.Bal-Domańska, BeataMarotta, GiuseppeBaldoni, EdoardoMarotti, JulianaBalducci, StefanoMarousek, JosefBalduzzi, GiacomoMarović, IvanBaldwin, Paula K.Marques Da Costa, Nuno Manuel SessaregoBalestra, ConstantinoMarques Telles, MonicaBaležentis, TomasMarques, AndreaBalin, AlicjaMarques, AntonioBalińska, AgataMarques, GoncaloBall, ChristopherMarques, Maria RitaBallani, FelixMarques, MarianaBallard, Allison J.Marques-Lopes, IvaBallarotto, GiuliaMarques-Sanchez, PilarBallco, PetjonMárquez, María Del Mar SimónBallester-Arnal, RafaelMarra, MaurizioBallestri, StefanoMarrano, NicolaBallmann, ChristopherMarranzano, MarinaBalogh, Esther A.Marrauld, LaurieBalogh, Jeremiás MátéMarrazzo, PasqualeBalsa-Barreiro, JoseMarriott, Bernadette P.Balsalobre-Lorente, DanielMarrone, OresteBalwicki, ŁukaszMarrone, RafaelleBalzano, TizianoMarroni, AlessandroBalzarro, MatteoMarrs, James A.Bambling, MatthewMars, MauriceBan, BhupalMarsch, AmandaBanach, ArturMarsden, JamieBanan, ZoyaMarshall, MalloryBandara, NishanthaMarshall, VirginiaBandaralage, Jayeni Chathurika Amarathunga HitiMarsico, PetraBando, HiroshiMarsillach, JuditBanerjee, AbhishekMarsillas, SaraBanerjee, Nilam SanjibMarston, LouiseBanerjee, SantanuMarszałek, HenrykBanerjee, SudipMarszalek, MartaBanfi, GiuseppeMarszalek-Grabska, MartaBang, SangsuMartaindale, HunterBang, SungsigMartchenko, MikhailBani Hani, AlaaMartell, KevinBankamp, BettinaMartelletti, PaoloBańkowska-Sobczak, Agnieszka EwaMartellucci, StefanoBanks, LoriMarti, ElisabetBanno, MasahiroMartín Aragón, María Del MarBansah, KennethMartin Cabrera, EduardoBansal, AmitaMartin Cervantes, Pedro A.Bansal, Geetha P.Martín Espinosa, Noelia MaríaBao, Jun-XiangMartín María, NataliaBao, XueMartín Payo, RubénBao, YiMartín San Agustín, RodrigoBapputty, ReenaMartín Valero, RocíoBaptista Rosas, Raúl CuauhtémocMartin, ClydeBaptista, João SantosMartin, DanielBaquero, AsierMartin, DavidBar, HaimMartin, EricBara, AnnaMartin, GinaBara, JeffreyMartin, Jeffrey S.Barabari, PoyanMartín, José María MartínBarabino, BenedettoMartin, LuciBarac, MajaMartín, Manuel Jesús CoboBarahona Mora, AzucenaMartin, NielsBarak, BoazMartin, PatrickBaran, JoannaMartin, PaulBaran, Mette L.Martín, RafaelBaranowska, BarbaraMartin, Richard H.Baranowski, ThomasMartín, SilviaBarassi, GiovanniMartin, Stefan AdrianBarata, JoãoMartín-Conty, José LuisBarata, PedroMartinelli, MariannaBaratta, RossellaMartinetti, AlbertoBarattucci, MassimilianoMartínez Chacón, Armando J.Barazzuol, LaraMartinez Espinosa, Juan CarlosBarb, Adrian S.Martinez Hernandez, EliasBarbara, Uliasz-MisiakMartínez Iglesias, OlaiaBarbato, ErsiliaMartínez Jimenez, Eva MaríaBarbato, GiuseppeMartínez López, Emilio JoséBarbayannis, NikosMartínez López, José IsraelBarbeau, WilliamMartínez López, LuisBarber, Sarah L.Martínez Ramón, Juan PedroBarberes, GabrielMartinez Ruiz, Maria AngelesBarberis, NadiaMartinez, Alexandre SoutoBarbic, FrancaMartinez, AntoniBarbier-Greenland, KatyMartinez, FelipeBarbieri, AntonioMartinez, HomeroBarbieri, Maria MaddalenaMartínez, IsabelBarbieri, MaurizioMartínez, Juan PedroBarbon Junior, SylvioMartínez, Luis CarlosBarbon, SilviaMartínez, Noemí SansóBarbosa Filho, Valder CordeiroMartínez, Silvia VarelaBarbosa, AlexandreMartinez, UrsulaBarcaccia, BarbaraMartinez, YolandaBarcala-Furelos, RobertoMartínez-Aires, María DoloresBarceló, Maria A.Martinez-Amezcua, PabloBarcena, CarmenMartínez-Bello, Daniel AdyroBarchitta, MartinaMartínez-Calvillo, SantiagoBarcik, JacekMartinez-Camblor, PabloBarclay, LesleyMartínez-Cañamero, MagdalenaBard, DelphineMartínez-Cañas, RicardoBardag-Gorce, FawziaMartínez-Córcoles, MarioBarden, Charles J.Martínez-Ferrer, BelénBarding, Gregory A.Martínez-Galiano, Juan MiguelBardy, FabriceMartínez-García, AlbaBarela, José AngeloMartínez-Gómez, MónicaBargellini, AnnalisaMartínez-González-Moro, IgnacioBargiel-Matusiewicz, KamillaMartínez-Guardado, IsmaelBari, AnasseMartinez-Guryn, KristinaBarik, SailenMartínez-Hackert, ErikBarilli, AmeliaMartínez-Hernández, FabiánBarkan, DanielMartínez-Isasi, SantiagoBarkauskienė, RasaMartinez-Jarreta, BegoñaBarker, Bridget M.Martínez-Linares, Jose ManuelBarker, EileenMartinez-Lopez, ErikaBarkham, TimothyMartínez-Martinez, JesúsBarmparis, Georgios D.Martínez-Medina, RamónBarnard, MarieMartinez-Merinero, PatriciaBarnas, EdytaMartínez-Moreno, AlfonsoBarnes, ChristopherMartinez-Moreno, CarlosBarnes, PhilipMartínez-Nova, AlfonsoBarnes-Josiah, DeboraMartinez-Pujalte, AntonioBarnett, JohnMartínez-Rocamora, AlejandroBarneva, RenetaMartínez-Rodríguez, AlejandroBarney, ChantelMartinez-Rojas, EnriquetaBarney, DavidMartínez-Ruiz, VirginiaBaron, Julianne L.Martínez-Sabater, AntonioBaron, Kelly G.Martínez-Sánchez, NoeliaBarona, Camilo OrdonezMartínez-Sanz, José MiguelBarone, Daniel A.Martinez-Vispo, CarmelaBarosova, HanaMartin-Fernandez, MarioBarquín, Roberto RuizMartín-Gómez, CésarBarr, Kelli L.Martinho, JoséBarr, PeterMartinho, Vítor João Pereira DominguesBarra, AnnaMartiniello, LauraBarrable, AlexiaMartín-Montalvo, AlejandroBarragán Escandón, AntonioMartino Alba, RicardoBarragán Martín, Ana BelénMartino, GabriellaBarragán-Sánchez, RaquelMartino, Giuseppe DiBarralon, PierreMartino, SabataBarranco-Chamorro, InmaculadaMartino, SimoneBarranco-Ruiz, YairaMartino, Tartaglia GianlucaBarreca, FrancescoMartinopoulos, GeorgiosBarreca, SalvatoreMartinotti, SimonaBarreiros, André NevesMartinou, KellyBarrense-DIas, YaraMartín-Rodríguez, Alberto J.Barreto Moreira Couto, Maria CarolinaMartín-Rodríguez, FranciscoBarreto, Alexandre De BarrosMartins Amaro, Ana Paula BetencourtBarrett, Anne E.Martins, Alberto M. B.Barrett, Sharon E.Martins, Anabela CorreiaBarria, PilarMartins, FatimaBarrigón, Maria LuisaMartins, Felipe José AidarBarrino, FedericoMartins, Fernando M. L.Barriocanal, CarlesMartins, JoãoBarrios-Fernández, SabinaMartins, LisaBarrios-Rodríguez, RocioMartins, Lisa Maria de OliveiraBarros, NelsonMartins, Luis R. M.Barros, PaulaMartins, Maria Do RosárioBarrow, JimMartins, Mariana RenovatoBarry, HelenMartins, MartaBartak, MiroslavMartins, RuiBartal, AlonMartins, VâniaBartel, SabineMartín-Salvador, AdelinaBartelmeß, TinaMartins-Junior, PauloBartha, FerencMartinsone, IneseBarthel, ErichMartin-Vide, JavierBartkowiak, GrażynaMarti-Pastor, MarcBartle-Haring, Suzanne E.Marto, Carlos MiguelBartoli, FrancescoMartorana, AlessandroBartoli, MattiaMartos Martínez, ÁfricaBarton, ChristianMartos-García, RaúlBarton, JackMartu, Maria AlexandraBartonova, AlenaMartynowicz, HelenaBartosiewicz, AnnaMartysiak -Żurowska, DorotaBartosik, KatarzynaMartzog, PhilippBartosz, GrzegorzMaruthamuthu, MuraliBartova, LucieMaruya, KoheiBartzas, GeorgiosMaruyama, TakuyaBaruffini, MorenoMaruyama, TesshoBarwicki, JanMarván, Maria LuisaBaryeh, KwakuMarvão Pereira, AlfredoBaryła-Matejczuk, MonikaMarzal-Felici, JavierBarzanò, GiovannaMarzano, GilbertoBar-Zeev, YaelMarzantowicz, ŁukaszBarzilay, JoshuaMarzec, MichałBarzyk, Timothy M.Marzec-Grządziel, AnnaBasanta, HaobijamMarzi, IsabelBasco, LeonardoMarzo-Campos, Juan CarlosBashford, CarolMasahiro, ShojiBasilico, NicolettaMasaki, TakayukiBasińska, MałgorzataMasanotti, GiuseppeBasnet, SijanMasarone, DanieleBassi, GiuliaMascarenhas, CarlaBassim, CarolMascarenhas, MayaBastas, AliMascherini, GabrieleBaster, ZbigniewMasciadri, AndreaBasteris, AngeloMasciari, ElioBastide, RémiMascitti, MarcoBastone, Alessandra De CarvalhoMasekameni, Daniel M.Bastos, VerónicaMaselli, FilippoBastrzyk, AnnaMashino, SonoeBasu Choudhary, AtinMashreky, SaidurBasu, ArpitaMásia, JoaquimBasu, JayasreeMaskeliunas, RytisBasu, MrittikaMaskell, DanielBasu, PratyushaMaslin, KateBasu, RupsaMasoero, MarcoBatai, KenMason, FredBatal, MalekMason, Howard J.Batchelor, PaulMason, JayBathrellou, EiriniMasoud, IsanejadBathula, Chandra SekharMasoudi, MahyarBatista, Miguel Luiz JuniorMaspero, CinziaBatista, PatríciaMasresha, BalchaBatitucci, GabrielaMassaglia, StefanoBatsila, MarianthiMassalska, MagdalenaBattaglia, FortunatoMassar, KarlijnBattaglia, MartinMassari, FrancescoBattaglia, YuriMassaro, AlessandroBattah, BasemMassaro, MarikaBattista, RebeccaMassaro, SebastianoBattistel, Postdoc AlbertoMassart, AlainBattisti, FabrizioMasselot, PierreBattles, HeatherMassey, William V.Bau Macià, JosepMassimi, AzzurraBauer, MiloslavMassuça, Luís MiguelBauer, StefanieMasterson-Algar, PatriciaBauer, StevenMastoraki, AikateriniBauer, Ursula E.Mastroeni, Marco FabioBaugh, Reginald F.Mastroleo, FeliceBaum, Chad M.Mastropietro, AlfonsoBaumann, AnjaMasud, Md. Abdul KaiumBaumann, NicoleMasuda, HisashiBaumbach, GünterMasutani, KosukeBaumber, AlexMat, Marielle LeBaumgarden, JosephMatalas, AntoniaBaumgardner, DarrelMatano, FabioBaumstark-Khan, ChristaMatarese, GiovanniBaur, CynthiaMataruna-Dos-Santos, Leonardo JoseBausys, RomualdasMateeva, NellyBauza, ValerieMatei, IrinaBavaresco Gambassi, BrunoMate-Munoz, Jose L.Bawazeer, Saleh A.Mateo Urdiales, AlbertoBaxter, Victoria K.Mateo, Daniel ColladoBay, Jacquie L.Mateo-Lázaro, JesúsBaylina, PilarMateos García, José TomasBayon, YvesMateos García, José TomásBayona, Jaime AndrésMateos, RaimundoBayrak, MucahidMateos, RaúlBazan-Krzywoszańska, AnnaMateus, CeuBazhanova, Elena D.Mateus, SóniaBazzaz, MohammadMathai, MatthewsBeach, MichaelMather, CareyBeall, AlyssaMathes, Alexander M.Beane Freeman, LauraMatheson, JesseBeard, LarryMathew, Ashish J.Beardsmore, CarolineMathew, SnehamolBearn, DavidMathew, SupriyaBeatriz Morgado-Gamero, WendyMathieson, StephanieBeberok, ArturMathijsen, AnetaBecerril, HilarioMathijssen, JolandaBecher, GeorgMathioudakis, Alexander G.Becher, HeikoMathonnat, JackyBechini, AngelaMatías Pompa, BorjaBechsgaard, ThorMatias, Ana RitaBeck, Alan M.Matias, ThiagoBeck, DavidMatijošius, JonasBeck, MatthiasMatikainen, PasiBecker, GenevieveMatin, AbdulBecker, Joana ProençaMatlala, Sogo FranceBecker, Leandro BussMatłosz, PiotrBecker, NorbertMatos Mascarenhas, DalbertBecker, Randy A.Matos Silva, FátimaBeckes, LaneMatos, ManuelBeckett, EmmaMatos, NelsonBeckman, LindaMatos, RuiBeckmann, VolkerMatosinhos, Cristiano ChristofaroBecoña, ElisardoMatowicka-Karna, JoannaBédard, AnnabelleMatranga, DomenicaBediou, BenoitMatre, DagfinnBedla, DawidMatsler, A. MarissaBednarova, LuciaMatsubara, KeiichiBedoya-Perales, Noelia S.Matsuda, TakashiBeekman, MarianMatsuda, YuheiBeer, MeinradMatsuda, YukikoBeeri, ItaiMatsui, KentaroBeesley, JonathanMatsuki, TaroBegdache, LinaMatsumoto, HiroshiBegin, DanielMatsumoto, YoshinoriBegley, AndreaMatsumura, KentaBegoray, DeborahMatsuoka, TeruyukiBegum, KohinoorMatsusaki, TakashiBehar, AlbertoMatsuyama, YusukeBehboodi, AhadMatsuzaki, JuntaroBehringer, ErikMatsuzaki, KentaroBehrooz, LeiliMattarocci, GianlucaBeijers, LianMattern, EleanorBeil, KurtMatteucci, FrancescoBeilby, JustinMatthes, JoergBeitelmal, Wesam H.Matthew, YalchBekteshi, SarandaMatthews, EvanBelaidi, Abdel AliMatthews, VeronicaBélanger, MélissaMatthewson, GillBelay, BrookMattijssen, Thomas J. M.Belbahri, LassaadMattingly, RhondaBelcher, BritiniMattioli, Anna VittoriaBelcher, John R.Mattisson, CeciliaBelfiore, PatriziaMattoscio, DomenicoBelingheri, MichaelMattsson, JanetBell, CarynMatud, María PilarBell, ColinMatud, PilarBell, DavidMatus, JustinBell, GriffinMatus, Patricia IsabelBell, RichardMatusik, PawelBell, SimonMatusik, PawełBellandi, TommasoMatys, JacekBellar, DavidMatzeu, GiusyBellasi, AntonioMatziou, VasilikiBellato, AlessioMauceri, ManuelaBelleville, GenevièveMauceri, RodolfoBellew, WilliamMauerer, GerlindeBelli, SimoneMaugeri, AndreaBellido-Vallejo, José CarlosMaugeri, Andrea GiuseppeBellinger, Nisha MukherjeeMaupin, JonathanBellini, MassimoMaupome, GerardoBellini, RoméoMaurer, LeonieBellisario, ValeriaMaurice, LaurenceBello, Nicholas T.Maurício Alves, Da Motta SobrinhoBelloni, MilenaMauriz, ElbaBellovary, Bryanne N.Mauro, FrancescoBellringer, MariaMaurya, ShailendraBelluzzi, ElisaMaviglia, MarcelloBelmin, JoëlMavragani, AmaryllisBelmonte, GenuarioMavroeidi, AlexandraBelmonte, Jesús LópezMavrogonatou, EleniBelmonte-Ureña, Luis J.Mavromatis, TheodorosBel-Oms, InmaculadaMawhinney, ThomasBeloukas, ApostolosMawson, AnthonyBeltowski, JerzyMawson, SusanBeltran-Velasco, Ana IsabelMaxwell, HazelBelvederesi, ChiaraMay, LawrenceBelviso, ClaudiaMay, MeghanBelzunegui-Eraso, AngelMaya, JesúsBem, AgnieszkaMayberry, JohnBemke-Świtilnik, MagdalenaMayberry, KatelynnBempelou, Eleftheria D.Maybery, DarrylBen Ali, MahaMayett Moreno, YesicaBen Mansour, JamalMayeuf-Louchart, AliciaBen-Ami, NoaMayfield, Anderson B.Benassi, FedericoMayhew, JerryBencardino, DanielaMaynar Mariño, MarcosBench, ShaneMayor, David L.Benden, MarkMayordomo, RaquelBender, Hans-UlrichMayr, AstridBender, KathrynMays, DarrenBender, NicoleMazadiego, Luis F.Bendick, MarcMazaheri, MandanaBenedek, CsillaMazda, YusukeBenedek, JózsefMazidi, MohsenBenedetti, IlariaMazilescu, Crisanta-AlinaBenedict, BarryMazoteras-Pardo, VictoriaBenesh, JulieMazur, ArthurBenevene, PaulaMazur, JoannaBenfaremo, DevisMazur, MartaBengalli, RossellaMazur-Bialy, AgnieszkaBéni, SzabolcsMazur-Pączka, AnnaBenítez Arciniega, Alejandra DonajíMazza, CristinaBenitez Porres, JavierMazzacane, SanteBenitez, DomingoMazzatenta, AndreaBenítez-Sillero, Juan De DiosMazzetti, GretaBenito, María J.Mazzilli, FernandoBenjamin, CourteneyMazzocchi, PaoloBenjamin, ElliotMazzoli, EmilianoBenjamin-Neelon, Sara E.Mazzone, MarianBenka-Coker, Megan L.Mazzoni, SabrinaBenkel, IngerMazzucato, MonicaBenlloch Osuna, MayteMazzulla, GabriellaBenlloch, MariaMbelambela, Etongola PapyBennardo, FrancescoMbulo, LazarousBennardo, LuigiMc Dowell, CillianBenner, JuliaMC Laughlin, JamesBennett, JulieMcAleer, MichaelBennett, MichaelMcAloney-Kocaman, KareenaBennett, Richard S.McAreavey, RuthBennetts, HelenMcArthur, DavidBenson, SarahMcAslan, DevonBensoussan, AlanMcBride, Devin W.Bentenuto, AriannaMcBride, KateBentzen, MarteMcCabe, EllenBenvenuto, MarcoMcCallum, ClaireBenz, KorbinianMcCann, LeoBenzies, KarenMcCarter, RobertBerardelli, IsabellaMcCarthy, ElaineBerardino, Santino Eugénio DiMcCarthy, HelenBerber, NemanjaMcCary, Lindsay M.Berbrayer, DavidMcCauley, LindaBerchialla, PaolaMcCawley, MichaelBercu, Ana-MariaMcClean, StuartBerei, Emese BeátaMcClement, SusanBerendsen, BjornMcCluskey, StuartBerenguer, CarmenMcColl, KarenBerens, Eva-MariaMcConatha, Jasmin TahmasebBeretta, EdoardoMcConkey, RoyBeretta-Piccoli, MatteoMcCoy, Sarah J. BreeseBerezin, AlexanderMcCraty, RollinBerezuk, André GeraldoMcCreary, AllieBerg, KristenMcCrillis, ElizabethBerg, MichaelMcCullagh, Marjorie C.Berg, Ole Kristian BronesMcCullogh, NicolaBergamaschi, EnricoMcCurdy, KarenBergantini, LauraMcDaniel, JohnBerg-Beckhoff, GabrieleMcDermott-Levy, RuthBergdahl, AndreasMcDiarmid, Melissa A.Bergefurt, LisanneMcDonald, Catherine C.Bergen, DorisMcdonald, TedBerger, FabianMcDougall, JulianBerger, IrisMcDowell, LizBerger, MassimoMcDowell, Susan M.Berger, NicolasMcElrone, MarissaBerger, RitaMcElroy, EoinBerger, StefanieMcElroy, MaryBergersen, AneMcentire, David A.Bergés Tiznado, Magdalena ElizabethMcEwan, TroyBerges, Bradford K.McFadden, SandraBergin, RebeccaMcFarland, DeborahBerglund, BrittaMcFarlane, Deborah R.Berglund, ErikMcGarity, SuzanneBergmann, LukeMcGeachie, MichaelBergmeier, HeidiMcGlashan, EliseBergner, ErynnMcGrail, MatthewBergold, SebastianMcGrath, Andrew P.Bergsten, Eva L.McGrath, RyanBergström, RobertMcGregor, Bethany L.Berhane, Hanna Y.McGregor, DuncanBerková, KateřinaMcGroddy, Megan E.Berkowsky, RonaldMcGuire, JeniferBerlanda, NicolaMcGuire, ShelleyBerman, TamarMcHill, Andrew W.Bernabé, Daniel GaleraMchiza, ZandileBernabe, EduardoMcInnes, JudyBernabéu, CarmeloMcIntosh, John L.Bernabeu, RodolfoMcJarrow, PaulBernabeu-Wittel, MáximoMcKay, Carly D.Bernal Escoto, Blanca EstelaMckay, KathrynBernal-Guerrero, AntonioMcKay, PatriciaBernard, BenoîtMcKenna, Peter EdwardBernardazzi, ClaudioMcKenna, Verna B.Bernardello, FabioMcKenzie, BriarBernardes, RafaelMcKenzie-Gopsill, AndrewBernardi, BrunoMcKeown, MickBernardi, SaraMcKinley, ErinBernardino, Raquel L.McKune, Sarah LindleyBernardo, Maria AlexandraMcLagan, DavidBernardo-Filho, MarioMclain, JeanBernardo-Sánchez, AntonioMcLaren, Margaret J.Bernhard, GermarMcLay, LaurieBernhard, ThuyMclean, AlistairBernhardt, RitaMcLean, Kristen E.Berniak-Woźny, JustynaMcLellan, BenBernstein, Eve R.McLeroy, KennethBernstein, JoshuaMcLoughlin, LarisaBerrigan, DavidMcLucas, BruceBerryman, CarolynMcLuskey, JohnBershteyn, AnnaMcMahon, GerardBertapelli, FabioMcMaughan, Darcy K.Bertasi, GiampietroMcMillan, DavidBertea, CinziaMcMillen, RobertBerteau, Jean-PhilippeMcmorrow, TaraBertelloni, Carlo AntonioMcNamara, JohnBertelloni, FabrizioMcNerney, MikeBerthelot, LaurelineMcOliver, CynthiaBerthold, AnneMcQueen, David V.Bertin, MelanieMcQuire, CherylBerton, FedericoMcsharry, CharlesBertoncini, Bruno VieiraMcSweeney, Matthew B.Bertram, HanneMcVey, David ScottBęś, AgnieszkaMcVicar, AndrewBeseler, CherylMcWhorter, AndreaBesnier, FlorentMcWilliam, RobinBessa, LucindaMdegela, MselengeBessa, TheolisMd-Yunus, Sham’AhBesse Rimba, AndiMeadows, AngelaBessems, KathelijneMeah, VictoriaBesser, GeroldMeanley, StevenBesser, LilahMearns, GaelBessergenev, Valentin G.Mearns, KathrynBessette, BarbaraMears, MeghannBessin, RicMeccariello, RosariaBest, CatherineMedas, DanielaBest, RussellMedellín-Castillo, NahumBetancourt, JoseMedici, GiacomoBeteck, Richard M.Medina Quero, JavierBetz, Heather HayesMedina Velázquez, Luis AlbertoBeug, MichaelMedina, F. XavierBeutell, Nicholas J.Medina-Ramirez, AdrianaBevilacqua, RobertaMedina-Ramírez, IlianaBex-Reimert, ViolaMedina-Vera, MyriamBezerra, BarbaraMedina-Vicent, MariaBezirtzoglou, EugeniaMediouni, SoniaBezner, JanetMedley, StuartBhandari, KrishnaMędrzycka, WiolettaBhandari, Manohar PrasadMedunić, GordanaBhandari, RamjeeMedupin, CeciliaBhardwaj, GouravMeduri, AlessandroBhargava, KritiMedvedev, Ilya NikolaevichBhaskar, SonuMedyńska-Juraszek, AgnieszkaBhat, AmrithaMeer, MargaritaBhat, OwaisMeerhoff, LaurentiusBhat, Sartaj AhmadMeerhoff, RensBhat, SushanthMeeroff, DanielBhatia, NatashaMeerson, AriBhattacharjee, ArindamMegna, MatteoBhattacharya, AnannyaMehan, SushantBhattacharya, AnasuaMehbub, Mohammad FerdousBhattacharya, SomanonMehdi, TahsinBhattacharyya, TapanMehdipanah, RoshanakBhattarai, ChiranjiviMehlhorn, HeinzBhattarai, KeshavMehrotra, PrakharBhattarai, KrishnaMehta, Ambereen KurwaBhatti, DanishMehta, NoshirBhattu, DeepikaMehta, SunaliBhave, DevayaniMei, YangBhawal, Ujjal KumarMeier-Stephenson, VanessaBhayat, AhmedMeiguins, BianchiBhochhibhoya, AmirMeil, William M.Bhowmik, DebsindhuMeinelt, ThomasBhuiyan, AzadMeitner, MichaelBhurosy, TrishneeMeixner, OliverBi, ChaoMejia Caceres, Maria AngelicaBi, YonghongMejzlik, JanBiagi, MarcoMeka, PenchalaBiałek-Jaworska, AnnaMekhemar, MohamedBiałowiec, AndrzejMekuchi, MiyukiBiałowolski, PiotrMelão, NunoBian, EmilyMelé, Pere MercadéBiancari, FaustoMeléndez Moral, Juan C.Biancarosa, IreneMelendez, RitaBianchi, Anna MariaMelfos, VasiliosBianchi, FabrizioMelgaard, DorteBianchi, JonasMelguizo-Rodriguez, LuciaBianchini, ElisabettaMeli, VijaykumarBianco, AngelicaMeliker, JaymieBianco, DavideMelini, FrancescaBianco, RosellaMelisa, StevanovicBias, ThomasMella, JaimeBiasini, Fred J.Mello, Johanna De AlmeidaBibra, MohitMellor, DuaneBickerdike, AndreaMelly, LudovicBickford, Marion E.Melo Villar, LiviaBiddle, Stuart J. H.Melo, PauloBidmon, SonjaMelo, PedroBidoli, EttoreMeloni, CarloBiegala, MichalMeloni, FernandoBiel, WiolettaMeloni, ItaloBielawska-Batorowicz, EleonoraMelrose, JamesBieleke, MaikMemon, ImranBieleninik, ŁucjaMemon, TosifaBielinis, ErnestMenahem, SolomonBielski, StanisławMenal, SusanaBień, AgnieszkaMénard, HervéBieniek-Tobasco, AshleyMena-Tudela, DesiréeBienvenido-Huertas, DavidMenchini Fabris, Giovanni BattistaBigelow, SethMendall, MichaelBigliardi, BarbaraMendenhall, AmyBikov, AndrásMendes Lipinski, JussaraBilan, YuriyMendes, AdrianoBilancini, EnnioMendes, Fernando José Figueiredo Agostinho D’AbreuBilbao, NaiaraMendes, Larissa LouresBillat, VeroniqueMendes, LuisBilliard, MichelMendes, PedroBilliot, ShanondoraMendes, RafaelBilski, JanMendes, SusanaBin, ShengMéndez Bolaina, EnriqueBin, Yu SunMendez Campos, BarbaraBinda, GilbertoMéndez Mateo, InmaculadaBindels, JacquesMendez, FranciscoBinder, AliceMéndez, Loyda B.Bingham, EllaMendez, Martin OswaldoBini, ClaudioMendez, RosaBinti Ramli, Nur SuhailiMéndez-Martínez, CarlosBiolatti, MatteoMéndez-Ulrich, Jorge LuisBiondi, BeatriceMendo, AntónioBirch, PhilMendogni, PaoloBirchenall, Katherine A.Mendo-Lázaro, SantiagoBirjandi, Anahid AhmadiMendonca-Torres, Maria CeciliaBirkás, MártaMendoza, Daniel L.Birmingham, C. LairdMendoza, Rose Marie O.Bíró, ÉvaMendoza-Cano, OliverBirt, LindaMendoza-Nuñez, Victor ManuelBiscarini, FilippoMendy, Angèle FloraBischoff, KarynMeneghini, Anna MariaBisri, Mizan Bustanul FuadyMeneguzzi, AlvaroBister, StefanMeneguzzo, FrancescoBiswas, ArijitMeneguzzo, PaoloBiswas, AviroopMenendez, Hector D.Biswas, SaptashatiMenendez, MiguelBis-Wencel, HannaMenescardi, CristinaBith-Melander, PollieMenezes, José C. J. M. D. S.Biudes, Marcelo S.Meng, AnnetteBiudes, Marcelo SacardiMeng, FanxinBiviá-Roig, GemmaMeng, FanyuBiziuk, Marek K.Meng, MengBizzoca, DavideMeng, QingchunBjørge, HeidiMeng, ShujuanBjörklund, ChristinaMeng, YuBlack, GeoffreyMeng, Yuan XiangBlack, Iain R.Menin, DamianoBlackburn, JoannaMenna, Lucia FrancescaBlackman, RossMennis, JeremyBlackshear, Tara B.Mento, CarmelaBlackstone, SarahMenvielle, LoickBlades, MarkMeoni, GabrieleBláha, LadislavMercader, IsabelBlaine, TomMercado, Jesús M.Blaise, Nguendo YongsiMercer, AndrewBlake, HollyMercer, John A.Blake, MarianneMerchan, Carlos IglesiasBlanck-Lubarsch, MoritzMerchán-Baeza, Jose AntonioBlanco Blanco, JoanMercincavage, MelissaBlanco, ÁngelesMercury, Santo RaffaeleBlanco, JuanMeregaglia, MichelaBlankinship, Lisa AnnMeregildo Rodriguez, Edinson DanteBlanton, CynthiaMergelsberg, EnriqueBlanton, LucasMergoni, GiovanniBlasco, DaniMerico, EvaBlasco-Lafarga, CristinaMerimanov, SerikBlaser, ArthurMerino, Ismael FuenteBlaskova, MartinaMerino, Jose JoaquinBłaszczak, UrszulaMerino-Andres, JavierBłaszczyk, EwaMerkiel-Pawłowska, SylwiaBłaszczyk-Bębenek, EwaMerlo, Emanuele MariaBłaszczyszyn, MonikaMerlo, GiaBlaya Haro, FernandoMermier, Christine M.Błażewicz, AnnaMero, AnttiBlazy, RafałMerollini, KatharinaBlecharz, JanMeroni, GabrielleBleizgys, AndriusMerrell, Laura K.Blesius, LeonhardMerritt, RowenaBlewitt, ClaireMerry, MelissaBlicharska, ElizaMertens, ChristianBlicharski, TomaszMesa, FranciscoBligh, BrettMesch, GustavoBliss, KadiMeschino, Diane De CampsBloemers, FrankMesenge, ChristianBloemers, JosMesimeri, MariaBlomme, EricMesinovic, JakubBlount, Robert J.Mesplède, ThibaultBloxham, SaulMesquita, JoãoBlumberg, Fran C.Messa, PiergiorgioBlunn, GordonMessaritakis, IppokratisBlunt, WarrenMesseri, AlessandroBoanca, PaunitaMessersmith, AmyBoansi, DavidMessina, GiovanniBoardman, JedMessina, JaneyBoateng, DanielMessineo, LudovicoBoateng, Godfred O.Messini, Christina I.Boaz, MonaMestanza-Ramón, CarlosBobkina, JelenaMestres, ConxitaBobowski, NualaMeszaros, MartinaBocarsly, MiriamMetcalf, OliviaBocchi, BarbaraMetcalf, PatriciaBocchieri, SalvatoreMeteran, HowramanBocchino, AnnaMetere, AlessioBoccia, FlavioMetovic, JasnaBoccio, Dana E.Mettifogo, MariangelaBocharova, IrinaMetwally, MayadaBochicchio, FrancescoMetzinger, LaurentBochniarz, MariolaMeulenberg, ElineBocian, SzymonMeurice, FrancoisBock, Joel R.Meyer, Alexa LeonieBockerman, PetriMeyer, AndrewBöckerman, Petri RikhardMeyer, João F. C. A.Bodendorfer, Blake M.Meyer, Martin Joh.Bodin, JohannaMeyer, SebastianBodomo, AdamsMeyer, StephenBoeckmann, MelanieMeyer, Thomas J.Boekhout, Janet M.Meylan, NicolasBoelens, LuukMeyn, LeslieBoente, CarlosMeza-Figueroa, DianaBoerchi, DiegoMezzomo Collares, FabrícioBoffi, MarcoMi, HongBogataj, DavidMiah, Mohammad IslamBogdan, MariaMialhe, Fábio LuizBogdanovica, IlzeMiani, AlessandroBogdański, PawełMiao, HongyuBoger, HeatherMiao, QingfengBoggero, IanMiao, RentaoBoggiano, Victoria L.Micera, RobertoBogueva, DianaMicha, DimitraBoguszewski, DariuszMichael, BryaneBöhmelt, TobiasMichael, JamesBohorquez, RocíoMichaels, NatalieBohrerova, ZuzanaMichał, PreisnerBoisvert, MichelleMichalczyk, MałgorzataBojnec, ŠtefanMichalik-Śnieżek, MalwinaBojorquez, IetzaMichaloski, Ariel OrleiBojsen-Møller, EmilMichalowska, JoannaBok, EwaMichalska-Dudek, IzabelaBolcha, PeterMichalski, GrzegorzBoldura, Oana-MariaMichel, AndreaBolevich, SergeyMichel, Jean-PierreBoling, Warren W.Michel, Marina EvrardBolis, IvanMichel, OlafBollig, GeorgMichele, CompagnoBollinger, LanceMicheletti, ChristianBolognesi, Salvatore FalangaMichelon, WilliamBolognini, DanieleMichikawa, TakehiroBoman-Davis, MarieMichishita, RyomaBombelli, GiovanniMichnik, Robert A.Bombieri, CristinaMiciuła, IreneuszBonadies, NicolasMickael, Michel-EdwarBonadonna, LuciaMickiewicz, WitoldBonan, Paulo Rogério FerretiMiçooğulları, Bülent OkanBonar, EmiliaMicucci, AlfonsoBonasia, RosannaMiddleton, Beth RoseBonati, MaurizioMiddleton, RebekkahBonato, MatteoMiddleton, Valerie A.Bonato, MaudMidtgaard, JulieBonaventura, AldoMieg, Harald A.Bond, MelissaMielgo-Ayuso, JuanBondoc, IonelMierek-Adamska, AgnieszkaBonduà, StefanoMierzwiak, RafalBone, MelissaMierzyński, RadzisławBonetta, SaraMietelski, Jerzy WojciechBong, Way KiatMiething, AlexanderBongaerts, BrendaMiglietta, NicolaBonham, JimMigliorati, MarcoBoniel-Nissim, MeyranMigliore, EnricaBonilla González, EdmundoMigliori, Carmela AnnaBonilla, CarolinaMignano, AntoninoBonilla, Diego A.Miguel, CristinaBonmati, AnnaMiguel, SilviaBonnet, EmmanuelMíguez, Maria del CarmenBono Cabré, RoserMihai, Florin-ConstantinBono, FilippaMihaicuta, StefanBono, RobertoMihaiescu, Tania CeciliaBonsaksen, ToreMijailovich, SrbaBonsu, Nana OseiMijiritsky, EitanBonuck, KarenMijiritsky, OriBonvicini, CristianMika, AnnaBoogaard, PeterMikalef, PatrickBooken, NinaMikayilov, Jeyhun I.Booker, JamesMikhailov, Theresa A.Boolani, AliMikhailova, ElenaBoon, ChengMikheev, Vladimir B.Boone, Edward L.Mikkelsen, EvaBoonstra, AlbertMikkola, TuijaBoortz-Marx, Richard LeviMiklós, MartinaBopape, Mary-Jane MorongwaMikolajczyk, EdytaBorame Lee, DickensMikšić, ŠteficaBoratyńska, KatarzynaMikuś, PawełBorbély, YvesMikušová, MarieBorecki, MichalMilán-García, JuanBorel-Hänni, FrançoisMilanowski, LukaszBorella, PaolaMilavić, BorisBores García, DanielMilazzo, MarioBorge, RafaelMilbrandt, EricBorgers, AloysMild, Kjell HanssonBorges De Sousa Magalhães, Bruno MiguelMiles, Toni P.Borges, AfricaMileti, IlariaBorges, AlissonMiletta, MariaBorges, Ana IsabelMilham, PaulBorges, Jose G.Milia, EgleBorges, RicardoMilić, JakovBorges, Tatiana LongoMilichovský, FrantišekBorghi, FrancescaMilionis, AthanasiosBorgonovi, ElioMilis, KevinBorisenkov, Mikhail F.Milkovich, ElizabethBorkowska, Aneta R.Milleman, Jeffery L.Borkowski, PrzemyslawMillen, AlettaBorm, PaulMiller Jones, JulieBorn, BrandenMiller, Anthony B.Born, ChristopherMiller, ElżbietaBorn, PatriciaMiller, EmmaBornioli, AnnaMiller, Gary W.Boronat, MontserratMiller, GloriaBoroń-Kaczmarska, AnnaMiller, Jillian VinallBoros, LajosMiller, Kathleen K.Borovac, Josip A.Miller, LaurieBorowik, AgataMiller, LisaBorowska-Stefańska, MartaMiller, SarahBorowski, PiotrMiller, Virginia M.Borraccino, AlbertoMiller-Lewis, LaurenBorrego, ÁngelMillham, RichardBorri, DinoMilligan, GemmaBorriello, GiorgiaMillion, A. J.Borro, LucaMillman, Zachary BenBorro, MarinaMills, HayleyBorruso, GiuseppeMilne, Barry JohnBortey-Sam, NestaMilner, JamesBortkiewicz, AlicjaMilner, Trudie F.Bortolotto, TissianaMilosavljević, IvanBorucka, AnnaMilosevic, DraganBorychowski, MichałMilton, MarionBorymski, SławomirMims, DebraBorys, MichałMin, LiuBorzabadi-Farahani, AliMina, RobertoBorzì, FrancescaMinakata, YoshiakiBorzikowsky, ChristophMinami, YoichiBos, ElskeMinarchick, ValerieBosch-Capblanch, XavierMinard, Charles G.Boschetti, TizianoMinassians, Henrik P.Boschman, Julitta S.Minaya, DulceBosco, AlessandroMincarone, PierpaoloBose, ArneshMincione, AntonioBose, SrimoyeeMîndrescu, VeronicaBose, TanimaMinea, IonutBosia, DanielaMinghelli, BeatrizBoskey, Elizabeth R.Mingorance-Estrada, Ángel CustodioBossew, PeterMinian, NadiaBossi Fedrigotti, ValérieMininni, Alba N.Bosso, LucianoMinnaar, PhillipBossu, MaurizioMinotti, BrunoBostic, JeffreyMinova, ZuzanaBoswell, NikkiMintzes, BarbaraBotelho, JoãoMiotla, PawelBotelho, Raquel Braz AssuncaoMiousse, IsabelleBotes, LuciusMiovsky, MichalBottenberg, PeterMir, DebbyBotti, LuciaMir, Tahir MaqboolBottoms, LindsayMira, José JoaquínBotturi, AndreaMirabelli, MariaBotubol-Ares, José ManuelMiraglia, MariellaBouaïcha, NoureddineMiraglia, SimoneBouaziz, WalidMiralles, Conxita MestresBoubchir, LarbiMiralles, FrancescBoubert, LauraMiralles, Jose LuisBoucher, JulieMiran, Seyed M.Boudjema, SophiaMiranda Barbosa, EdesioBoufounou, ParaskeviMiranda Duro, María Del CarmenBoukerrou, LakhdarMiranda Paez, JesúsBoukhibar, Lamia MestekMiranda, JonatanBoulahouache, ChaoukiMiranda, Jose M.Boulanger, Saveria Olga MurielleMiranda-Ackerman, Marco A.Bouloumpasis, IoannisMiranda-Sanchez, FabiolaBoulton, ChrisMiranda-silva, DanielaBoulton, DanielMiras-Avalos, Jose ManuelBoulton, ElisabethMirete, LucíaBouma, GaryMirey, GladysBoumadan Hamed, MoussaMirghorbani, SaeidehBoumghar, YacineMirlohi, SusanBourgeois, DenisMiró, ConradoBourgeois, Frank A.Miron, Isidro JuanBourikas, LeonidasMironeasa, SilviaBourliva, AnnaMirsch, JohannaBourne, AllisonMirza, MaryamBoursier, ValentinaMisaki, KentaroBoussios, StergiosMiscioscia, MarinaBoutaayamou, MohamedMishchuk, HalynaBouvier, Anne-MarieMishina, MasahiroBouwmeester, OnnoMishra, Amrit KumarBouza-Rodríguez, José BenitoMishra, Asit K.Bouzigon, RomainMishra, BijeshBovell, LiselleMishra, Geetesh K.Bover, JordiMishra, Jay S.Bowdle, AndrewMishra, JyotiBowen, DeborahMishra, ManishBowen, JeffMishra, MeerambikaBowker, KatherineMishra, MuditBowling, Heather L.Mishra, PriyankaBowman-Bowen, Lisa C.Mishu, MasumaBowmer, Kathleen H.Misiak-Kwit, SandraBoyages, JohnMiskiewicz, AndrzejBoyd, BillMiskowiec, PawełBoyd, JacquelineMiskulin, IvanBoyd, RobertMissoni, EduardoBoykan, RachelMist, Scott D.Boyland, EmmaMistiaen, WilhelmBoyoung, HeoMisyak, SarahBozic, JoskoMiszczyk, JustynaBozkurt, SelimMiszczyńska, Katarzyna M.Bozlaker, AyşeMitanchez, DelphineBozzato, PaoloMitáš, JosefBozzo, GiancarloMitchell, ConstanceBraatz, FrankMitchell, FeliciaBracci, EnricoMitchell, John S.Bracci, MassimoMitchell, Shamika AnnBracco, FabrizioMitra, Amal K.Brach, CindyMitre, NaimBrackbill, Robert M.Mitrou, FrancisBraczkowski, RyszardMitsakou, ChristinaBradley, EleanorMitsea, Anastasia G.Bradley, Robert S.Mitsiakos, GeorgiosBradshaw, BenMittal, KarunaBrådvik, LouiseMittapelly, PriyankaBrady, Benjamin R.Mitten, DeniseBraga, Ana CristinaMiura, HirokoBragada, Jose A.Miura, TakayukiBragazza, LucaMiwa, Julie M.Bragazzi, Nicola LuigiMiyachi, RyoBrager, AllisonMiyagawa, MuneyukiBragg, MarieMiyahara, MotohideBragg, RobertMiyama, TakeshiBragonzoni, LauraMiyamoto, IkuyaBrągoszewska, EwaMiyamoto, YukiBrahmajothi, Mulugu V.Miyata, SeikoBrai, AnnalauraMiyazaki, JunBrainer, AmyMiyazaki, TetsuroBrajša-Žganec, AndrejaMiyoshi, Shin-ichiBraks, MarietaMizgier, MałgorzataBrall, CarolineMizrahi, ShlomoBramanti, EmiliaMizuguchi, MarikoBranca, Jacopo Junio ValerioMizuno, AkikoBranco, Manuel CasteloMizuno, KohBranco, PedroMizuta, KentaroBranco, VascoMizutani, ShinsukeBrand, SergeMkandawire-Valhmu, LucyBrandao, BrunaMladenova, IrenaBrandes, MirkoMlynarczyk, DariuszBrandhagen, MartinMłynarczykowska, AnnaBrandon, Jennifer A.Mlynarska, AgnieszkaBrandon, ThomasMłyński, DariuszBrands, EdwinMłyński, RafałBrandt, EricMo, ChenglinBrandt, Eric B.Mo, FeiBrandt, KirstenMoaddab, MahsaBranley-Bell, DawnMoberg, LouiseBrann, MariaMoccia, MarcelloBranthwaite, HelenMoccozet, LaurentBrasil, Gutemberg HespanhaMochimaru, MasaakiBrassetti, AldoMocny-Pachońska, KatarzynaBratland-Sanda, SolfridMóczár, CsabaBratosin, StefanModa, Haruna MusaBratt, AlisonModenese, AlbertoBratteteig, ToneModerau, NinaBråtveit, MagneModjadji, PerpetuaBrault, MarieModoni, GianfrancoBrauner, EdoardoMoè, AngelicaBraun-Lewensohn, OrnaMoertl, SimoneBraverman, EricMoggi, SaraBravo Olivas, Myrna LeticiaMogilski, Justin KyleBravo, MercedesMognetti, BarbaraBravo-Aguilar, MaríaMogut, DamirBrawner, Clinton A.Mohamed, MostafaBray, IssyMohamed, Salah A.Bray, James W.Mohan, DeviBrazier, JonMohebbi, AbolfazlBrazo-Sayavera, JavierMohebbi, AminBrčić Karačonji, IrenaMohelska, HanaBrecht, MarcusMohieldin, Ashraf M.Bredin, Shannon S. D.Mohiuddin, MuhammadBreen, MichaelMöhner, MatthiasBreeze, RuthMohr, David C.Bregenzer, AnitaMohrlok, UlfBrehm, WalterMoir, GavinBremberg, SvenMoir, Gavin L.Brendel, Alfred BenediktMojiri, AminBrennan Grieve, Victoria LunaMojsa-Kaja, JustynaBrennan, LindaMojtahedi, ZahraBrennan, PatriciaMojumdar, KamalikaBresciani, SabrinaMokgatle, Mathildah MpataBresin, KonradMokhlespour Esfahani, Mohammad ImanBressot, ChristopheMoktefi, AnissaBreton, EricMokwena, KebogileBretzke, JamesMoldan, BedřichBreuker, AnjaMoldogazieva, Nurbubu T.Brewer, Britton W.Moldowan, John MichaelBrian, MichaelMolenbroek, JohanBricard, DamienMolero Jurado, María Del MarBriciu, Andrei-EmilMolero, PatricioBricknell, MartinMolero, Pilar PuertasBridge, CatherineMolgat-Seon, YannickBriedis, VitalisMolin, FredrikBriggs, BrandonMolina Fernández, Antonio JesúsBright, Candace ForbesMolina Leyva, AlejandroBrígido, ClarisseMolina Moreno, ValentínBrilman, WimMolina Torres, GuadalupeBrimhall, Kim C.Molina, FranciscoBrindisi, MatteoMolina-González, María DoloresBrindley, PaulMolina-Hidalgo, CristinaBringas, Maria LuisaMolina-Luque, RafaelBrinkmann, ChristianMolina-Mula, JesúsBrinthaupt, Thomas M.Molinaro, SabrinaBrion, JohnMolina-Sánchez, HoracioBriones-Vozmediano, EricaMolina-Torres, GuadalupeBrisbois, BenMoline, JacquelineBrito Beck Da Silva, KarineMoliner-Urdiales, DiegoBrito, LeandroMolino, MonicaBrito, PauloMolitor, JohnBrito, PedroMollalo, AbolfazlBritt, EileenMöller, HolgerBritton, UnaMöller, SörenBritt-Rankin, JoMollica, CristinaBrkic, Faris F.Molnar, JosephBrković Dodig, MartaMolter, AnnaBroach, JosephMoltzan, Catherine J.Broadhurst, C. LeighMomma, HarukiBroady, TimothyMomsen, Janet HenshallBrocchi, Beatriz ServilhaMona, MahmoudBrocco, EnricoMonaco, Maria Grazia LourdesBrochu, PaulaMonaghan, ThomasBrodehl, AndreasMonahan, KathleenBrodziak, AnetaMonahan, Kevin J.Broessner, GregorMonahan, KyleBrogi, SimoneMonaro, MerylinBrognara, LorenzoMoncayo, RoyBroitman, DaniMondal, PritishBrombach, ChristineMondal, SudipBroniewicz, ElzbietaMondal, Utpal KumarBronikowska, MalgorztaMondéjar-Jiménez, JoséBronikowski, MichałMonfort-Pañego, ManuelBrønnum-Hansen, HenrikMongkuo, Maurice Y.Bronzaft, ArlineMónico, Lisete Dos Santos MendesBrooke, JoanneMon-López, DanielBrooker, KatieMonroy, FernandoBrookes, Sharon M.Mons, UteBrooks, Alyssa T.Monsarrat, PaulBrooks, Audrey J.Monsuez, Jean-JacquesBrose, LeonieMont, DanielBrotto, MarcoMontag, Annika C.Brough, LouiseMontag, ChristianBrown Mahoney, ChristineMontagna, Maria TeresaBrown, AliceMontalvo, Fermín TorranoBrown, Bryan R.Montaña Blasco, MireiaBrown, CaitlinMontana, JessicaBrown, Candace S.Montanari, MarcoBrown, Carol E.Montanes, AntonioBrown, Cary A.Montano, SteffanoBrown, ChrisMontazeri, HamidBrown, ClareMonteagudo Chiner, PabloBrown, DennisMonteagudo, AndreaBrown, FreddyMonteagudo, Noemí CuarteroBrown, JamesMonteil, ChristelleBrown, JoeMonteiro, AnaBrown, JulieMonteiro, Carlos EduardoBrown, Kathleen C.Monteiro, CristinaBrown, Katie N.Monteiro, DiogoBrown, LauraMonteiro, LuísBrown, LaVerne L.Monteiro, MarianaBrown, LeanneMonteiro, Samuel José FonsecaBrown, MarkMonteiro-Soares, MatildeBrown, PhilMontemezzi, PietroBrown, RobMontemurro, NicolaBrown, Ronald B.Montero-Navarro, AntonioBrown, Scott C.Montes, HectorBrown, SuzanaMontes-Botella, Jose-LuisBrown, VictoriaMontgomery, MaggieBrowne, ChrisMontgomery, MichelleBrowne, JenniferMontiel, José MaríaBrowne, MatthewMontlaur, Adeline DeBrownlee, IainMontllor, JoanBrownlee, Iain A.Monton, EnriqueBrözel, VolkerMontoro, Miriam AgredaBrożyna, JacekMontoya Alonso, Jose AlbertoBrucker, NatáliaMontoya-Castilla, InmaculadaBrucoli, MatteoMontserrat Codorniu, JuliaBrueger, Andreas X.Montusiewicz, JerzyBruggmann, PhilipMonyeki, Kotsedi DanielBrugliera, LuigiaMoody, KyleBruinsma, GuidoMoon, IgiBruland, DirkMoon, Kyong WhanBrumitt, JasonMoon, Kyoung-SikBrundage, Cord M.Moon, SohyunBrunner, ElizabethMoonesinghe, RamalBruno, AntonioMoore, Amy M.Bruschini, LucaMoore, Brianna F.Brustio, Paolo RiccardoMoore, ChesterBruun-Pedersen, Jon RamMoore, JoeBruzzese, DarioMoore, KarenBruzzese, FrancescaMoore, Kevin C.Bruzzone, SilviaMoore, LibbyBryant, BartMoore, RolandBryant, ChristopherMoore, Ronald B.Bryant, KimberleyMoore, SimonBryl, KarolinaMoore, SylviaBryla, PawelMoore, T. J.Brytek-Matera, AnnaMoored, KyleBrzezińska, DorotaMoore-Nall, AnitaBrzeziński, MichałMora Ríos, EliaBrzozowski, JanMora, Miguel Angel MoralesBubnys, RemigijusMora, RodrigoBubu, OmonighoMorabito, MarcoBucchi, LauroMoraes, Renato De OliveiraBucci, MarcoMorais, Jorge E.Bucci, Maria PiaMorais, NunoBuccieri, KristyMorais, SamanthaBuch, EricMoraleda Sepúlveda, EstherBuchaim, Rogério LeoneMoraleda, ÁlvaroBuchanan, RobertMorales Conde, María JesúsBuchanan, SusanMorales Contreras, Manuel F.Buchanich, Jeanine M.Morales Suárez-Varela, María M.Buchet, ReneMorales, DiegoBuchmann, AnneMorales-González, José AntonioBuchowicz, AndrzejMorales-Morales, DavidBuchta, William G.Morales-Polo, CarlosBuck, ChristophMorales-Torres, AdrianBuckingham, LelaMorales-Vidal, MartaBuckley, JeffreyMoral-García, José EnriqueBuckley, JohnMoralli, DanielaBuckner, StefanieMora-Montes, HectorBucolo, ClaudioMoran, KevinBuczak-Stec, Elzbieta W.Morán, Mª LucíaBuda, Valentina OanaMoraveji, NeemaBuddaseth, SalmaMörchen, ManfredBudeanu, AdrianaMore, ElizabethBudimirovic, DejanMorea, DonatoBudnik-Przybylska, DagmaraMorean, MeghanBudryn, GrażynaMorehouse, RichardBudryte, DovileMoreira Da Costa, LuisBudzier, HelmutMoreira, CristianaBudzyń, MagdalenaMoreira, José Claudio FonsecaBudzynska, SylwiaMoreira, Mário W. L.Budzyński, MarcinMoreira, Mário Wedney De LimaBuelga, SofíaMorel, GaëlBueno Antequera, JavierMorenilla Jimeno-Morenilla, AntonioBueno-Silva, BrunoMoreno Manso, Juan ManuelBuesa-Estelléz, AlmudenaMoreno Molina, DavidBuff, RachelMoreno Villares, José ManuelBugajski, PiotrMoreno, DaniBuguña, DavidMoreno, FranciscoBui, DinhMoreno, Luis A.Buiarelli, FrancescaMoreno, OswaldoBuisseret, FabienMoreno, RománBujakowski, FilipMoreno, SaraBujan, JuliaMoreno-Andrés, JavierBujnowska-Fedak, Maria MagdalenaMoreno-Asso, AlbaBujnowski, AdamMoreno-Maldonado, ConcepciónBüke, TayfunMoreno-Marcos, Pedro ManuelBukhari, ZiaMoreno-Perez, VictorBukkuri, AnuraagMoreno-Rangel, AlejandroBuková, AlenaMoreno-Rodriguez, RicardoBuksnyte–Marmiene, LoretaMorente Oria, HonoratoBulai, Iulia MartinaMorente, Maria Ángeles PérezBulathwatta, AsankaMorente-Oria, HonoratoBulati, MatteoMorera, MariaBulbena, AntonioMoreso, FrancescBulejko, PavelMoret, CarmenBulfamante, Antonio MarioMoret-Tatay, CarmenBulińska-Stangrecka, HelenaMoretti, AntimoBulkeley, KimMoretti, FrancescaBuller, Ian DavidMoretti, SamueleBulli, PeterMoretto, AngeloBulotaitė, LaimaMorga, RafałBumbach, Michael D.Morgado, MarianaBunc, VaclavMorgan, AlexanderBunds, KyleMorgan, HaniBundy, AnitaMorgan, HaydnBungau, ConstantinMorgan, IanBungau, SimonaMorgan, JenniferBungum, TimothyMorgan, JillBuoite Stella, AlexMorgan, KellyBuonaguro, Elisabetta FilomenaMorgan, MarshaBuono, PasqualinaMorgan, PaulBuonomo, IlariaMorgan, Robert F.Buralli, Rafael J.Morgan, T. LeeBurattin, AndreaMorgante, GiuseppeBurdett, HowardMori, TaikiBurduhos, Bogdan-GabrielMoriarty, TerenceBurduk, PawełMorin, MathieuBureika, GintautasMorineau, ThierryBurger, Divan AristoMorishita, ShinichiroBurger, HuibertMorishita, YoshiyukiBurghardt, KyleMorita, ManabuBurgin, RachaelMorita, NoriteruBurgio, AlessandroMoriuchi, HiroyukiBurgos, HéctorMoriya, ManabuBurgos-Garcia, AntonioMoriyama, HideakiBurgos-Ramos, EmmaMoriyama, NobuakiBurgos-Robles, AnthonyMoriyama, YokoBurian, EgonMork, Knut AntonBurk, Christian L.Moroe, NomfundoBurkauskas, JuliusMoroń, MarcinBurkhart, Craig G.Morouço, PedroBurki, UmarMorozov, IgorBurkina, ViktoriiaMorozova, Vera V.Bürklein, SebastianMorpurgo, AndreaBurkowska-But, AleksandraMorrás, MaríaBurmester, VictoriaMorris, AbigailBurnett, HarveyMorris, DavidBurnley, StephenMorris, HeatherBurns, KatherineMorris, Meg E.Burns, LawrenceMorris, Robert D.Burns, Penny L.Morris, SheldonBurns, PippaMorris-Eyton, HeatherBurns, RyanMorrissey, HanaBurr, BrandonMorrissey, KarynBurr, WesleyMorriss-Roberts, ChristopherBurridge, TerryMorrone, AldoBurris, HeatherMorse, SiobhanBurris, Heather H.Morseth, BenteBurrmann, UlrikeMorseth, Marianne SandsmarkBurrola-Aguilar, CristinaMortazavi, RoyaBursac, ZoranMorton Ninomiya, Melody E.Burstein, RoyMoruzzi, Rodrigo BragaBurszta-Adamiak, EwaMorys, FilipBurtey, StéphaneMosavi, AmirBurton, Elvin ThomaseoMosby, TerezieBurton, Richard F.Mosca, Erica IsaBurton, RobynMoscatelli, SilviaBurzynska, MonikaMoser, Christine M.Bus, AgnieszkaMosgöller, WilhelmBus, James S.Moshammer, HannsBusardó, Francesco P.Mosier, BrianBusby, ChrisMoskalewicz, JacekBusch, AlbertMoslehpour, MassoudBusch, Sidsel StærmoseMosler, SaruhanBuschmann, AlejandroMoss, KarenBuschmann, JürgenMoss, MarkBuse, ChrisMostafavi Yazdi, Seyed JamaleddinBuse, Helen Y.Moszak, MałgorzataBuselli, RodolfoMotalebi Damuchali, AliBush, NickiMotamed, CyrusBusico, GianluigiMota-Rolim, Sergio A.Busquets, Maria AntòniaMotaze, VillyenBusse, HeideMotegi, AtsushiBustaffa, ElisaMotoie, GabrielaBustillo-Lecompte, CiroMotoyama, YasuyukiBustos-Martínez, JaimeMotra, HemBustos-Terrones, YanethMotrico, EmmaBuszko, KatarzynaMotta, Chiara MariaButkauskas, DaliusMotta, Fernanda LopesButler, JohnMottaghipisheh, JavedButler, MichelleMottet, DenisButler, SandraMotti Ader, Lilian GenaroButler, TamaraMotwani, MonaButler-Barnes, Sheretta T.Mou, XichenButlewski, MarcinMoudatsou, MariaButnariu, MonicaMouhyi, JaafarButterick, TammyMoulds, KylieBuvanaswari, P.Mouloud, KeniouaBuxton, SamuelMoulton, John KevinBuysschaert, JoostMourad, AhmadBuzzachera, Cosme FranklimMourad, KhaldoonBuzzi, MarinaMourão, Carlos FernandoBycura, DierdraMourão, Paulo ReisBydłosz, JarosławMourgues, CatalinaByeon, HaewonMousavizadeh, LeilaByeon, SanghoonMoutelíková, RomanaByrd, BrianMovsisyan, AniByrd, Karen S.Mowe, MaxineByrne, JenniferMoya Higueras, JorgeByun, Hi-RyongMoyano Muñoz, María De Las NievesByun, Soo-HwanMoyano, NievesBywater-Reyes, SharonMoyce, SallyC. Neves, JoãoMoyer, RachaelCaamaño Isorna, FranciscoMoysi, Joaquín SanchísCaballero, María J. ArévaloMozaffari, MahmoodCaballero, PabloMozas-Moreno, JuanCaballero-Mora, Maria AngelesMozumder, MohammadCabeen, MatthewMrazek, Alissa J.Cabello-Alvarado, Christian J.Mrowa-Hopkins, ColetteCabero, JulioMrówczyńska, MariaCabeza, Ricardo A.Mrowiec, BozenaCabeza, RuthMrugalska, BeataCabicieri Profice, ChristianaMu, HairongCable, NorikoMucci, NicolaCabral Pinto, MarinaMucciardi, MassimoCabral, Howard J.Muccio, RosyCabral-Pinto, Marina M. S.Mucha, WalterCabrera, Mildred Vanessa LopezMuchacka-Cymerman, AgnieszkaCabrera-Barona, PabloMuchova, ZlaticaCabrera-Chávez, FranciscoMucko, PrzemekCabrera-Munoz, NestorMudde, AartCaccavale, MauroMueller, JochenCaccianiga, GianluigiMueller, TorstenCacciottolo, MafaldaMuennig, PeterCáceres Jensen, LizethlyMuffato, VeronicaCáceres Reche, PilarMugabi, Ivan K.Cachada, Anabela Ferreira De OliveiraMugera, Amin W.Cadilla, CarmenMuggeo, Vito M. R.Caduff-Janosa, PiaMugica, VioletaCaffaro, FedericaMugueta-Aguinaga, IranzuCaffò, AlessandroMuhammad, NaoshadCafolla, DanieleMühlbacher, Axel C.Cagetti, Maria GraziaMühle, ChristianeCaggiano, GiuseppinaMuhle, PaulCagin, UmutMuhonen, TuijaCahan, RivkaMukherjea, ArnabCahill, Shaina P. A.Mukherjee, AmitavaCahir, CaitrionaMukherjee, DwaipayanCai, DeminMukherjee, KusumikaCai, HaiyuanMukherjee, RajCai, JialiangMukherjee, SoumyadeepCai, KuiMukherjee, SudipCai, KunzhengMukhopadhya, AnindyaCai, MengfanMukumbang, FerdinandCai, ShirongMulasso, AnnaCai, YafeiMullaugh, KatherineCai, YingyunMüller, FrankCai, Zong-YanMüller, Jochen A.Caire Juvera, GracielaMüller, JohannesCairncross, CarolynMüller, Kai W.Cairoli, Jean LouisMüller, LouiseCairós-Ventura, Luis MiguelMuller, MarcCake, MartinMüller, MartinCała, MarekMüller, NotgerCalabria, DonatoMüller, PatrickCalabria, ElisaMüller, StephanCalabuig-Moreno, FerranMuller, SylvianeCalamanti, ChiaraMűllerová, MonikaCalasans-Maia, Jose De AlbuquerqueMullie, PatrickCalati, RaffaellaMulligan, Megan K.Calbet, Jose A.Muloi, DishonCalcabrini, CinziaMulyasari, FarahCalcagnì, AntonioMunawir, MunawirCalcagno, GiuseppeMundi, Manpreet S.Calcaterra, ValeriaMuniyan, SakthivelCaldas, Lucio AyresMuniz Pumares, DanielCaldeiro Pedreira, Mari CarmenMuñiz, JoséCalder, AmandaMuniz, JuanCalder, RobMuñoz González, Juan ManuelCalderon, AntonioMuñoz Marín, DiegoCalderon-Garciduenas, LilianMuñoz, Cynthia E.Calderon-Monge, EstherMuñoz, Guiomar NocitoCaldwell, JacobMuñoz-Fernández, NoeliaCaleiro, AntónioMuñoz-García, AntonioCaliff, Christopher B.Muñoz-López, FranciscoCalin, Adrian CantemirMuñoz-Saavedra, LuisCalina, DanielaMunro, AlisonCałka, BeataMunro, AllanCalla, JaesonMunshi, SoumyabrataCallado, AndreMuntimadugu, EameemaCallander, Denton JamesMuntyan, Maria S.Calle, Mariana CeciliaMunuera, PedroCallea, AntoninoMura, GiuliaCallegari, BiancaMurab, SumitCallegari, CamillaMurakami, KeikoCalleja-Gonzalez, JulioMurakami, KeisukeCalle-Pascual, Alfonso LuisMuramoto, JojiCalmet, MichelMurante, Anna MariaCalò, Carla MariaMurase, DaikiCaltabiano, RosarioMurato, MiguelCaluk, NermaMurawska-Ciałowicz, EugeniaCalvo Arenillas, José IgnacioMurdoch, David R.Calvo Herrero, Luis MiguelMuresan, Iulia CristinaCalvo Lerma, JoaquimMuresan, VladCalvo Lobo, CésarMurff, Sharon H.Calvo, AngelaMurfree, LaurenCalvo, Maria Icíar ArteagoitiaMurgante, BeniaminoCalvo, NuriaMuriana, CinziaCalvo-Lluch, ÁfricaMuriel-Sánchez, Juan ManuelCalvo-Lobo, CésarMurillo Tovar, Mario AlfonsoCalvo-Sotomayor, IñigoMurino, TeresaCalzada-Mendoza, Claudia C.Murmura, FedericaCamacho Chab, Juan CarlosMurnen, Sarah K.Camacho Miñano, María JoséMuro Jr., AldoCamacho, David ParraMuro, AldoCamacho-Cardeñosa, AlbaMuro, ShigeoCamacho-Conde, Jose AntonioMurotani, KentaCama-Pinto, AlejandroMurphy, Caroline S.Camarero-Figuerola, MartaMurphy, EleanorCambray, SerafíMurphy, JillCambron, ChristopherMurphy, MaureenCamden, ChantalMurphy, RaeganCamden, Matthew C.Murra, DanieleCame, HeatherMurray, AndrewCamerin, FedericoMurray, EstherCamerino, DonatellaMurray, MeganCamerino, OleguerMurray, Peter E.Cameron, DanielMurray, RiannaCamiciottoli, GiannaMurray, Steven RossCamila, Hochman-MendezMurrell, Audrey J.Camina Martin, AliciaMurrell, DedeeCamkin, JeffMurrell, George A. C.Camorrino, AntonioMuruganandam, GopinathCampa, FrancescoMuruganandan, ShanmugamCampagna, SaraMusazzi, Umberto M.Campana, Luca GiovanniMuscat, DanielleCampanini, AnnamariaMuscat, Joshua E.Campbell Jenkins, Brenda W.Musetti, AlessandroCampbell, GraceMushtaq, NasirCampbell, JackieMuskin, Philip R.Campbell, JulieMusolino, DarioCampbell, KatarzynaMusonda, InnocentCampbell, MorganMußbacher, MarionCampbell, PatMusselwhite, CharlesCampbell, PaulMussey, Joanna L.Campbell, RochelleMusso, NataleCampeotto, FlorenceMustacchi, GiorgioCampioni, IlariaMustachio, LisaCampisciano, GiusiMustafa, AhmedCampisi, GiuseppinaMustafa, GhulamCampo, GiuseppeMustapha, AdetounCampo, LauraMuszyński, SiemowitCampos Souza, Paulo VitorMuthu Karuppan, Mohan KumarCampos, Alex Fabiano CortezMuthugala, M. A. Viraj J.Campos, Esther RocaMuthumalage, ThivankaCampos, FelipeMuthuraju, SanguCampos, Maria Jorge GeraldesMutoro, Antonina N.Campos-Sánchez, Francisco SergioMutrie, NanetteCampus, GuglielmoMutshinda, CrispinCamu, WilliamMuttin, FrédéricCamus, MelindaMutu, Margaret ShirleyCañadas-De La Fuente, Guillermo A.Myers, AnnaCañas, ElizabethMyers, CandiceCanavese, FedericoMyers, LauraCancela Carral, José MªMyers, SamuelCancelliere, PiergiacomoMyers, VickiCancello, RaffaellaMylonas, IoannisCancelo, Maite TeresaMyneni, AjayCanciani, ElenaMyslinski, NorbertCandiani, SimonaMysliwiec, TamiCândido Carvalho, KátiaMyszkowska-Ryciak, JoannaCandido, RenatoMytych, JenniferCanedo, Edna DiasNa, Hyeong SukCaneiras, CátiaNa, MuziCanha, NunoNa, SanggyunCaniato, MarcoNaald, BrianCannistraro, GiuseppeNabaweesi, Rosemary K.Cannito, MaddalenaNabors, Laura A.Cannizzaro, EmanueleNacif Antunes, MicheleCannon, ClareNadais, HelenaCano Lozano, María Del CarmenNadal, MiquelCanosa, StefanoNadeem, MuhammadCantero-Garlito, Pablo A.Nader, MartínCantisani, GiuseppeNaderi, NassimCantista, PedroNaderpour, MohsenCanto, JesúsNadhim, Evan A.Cantó-Navés, OriolNadorff, Danielle K.Canton-Cortes, DavidNaessens, James M.Cantor, TarperiNaezer, MarijkeCantor-Cutiva, Lady CatherineNafees, LubnaCantrell, JenniferNagai, NorihiroCanziani, RobertoNagao, HideyukiCao, Gui-yingNagaraj, ViniCao, JianjunNagarajan, PrabakaranCao, MengqiuNagare, RohanCao, QingguiNagasaka, KazunoriCao, ShunshengNagasawa, KeisukeCao, WangnanNagashima, KengoCao, XuebingNagelkerke, NicoCao, YangNagington, MauriceCao, YingtingNagisetty, RajaCao, ZhidongNagórska, MalgorzataCao, ZhiguoNagórska, MałgorzataCao, ZhixingNaguib, MahmoudCaparrós-González, Rafael ArcángelNaguib, TarekCapasso, AnnaNagwekar, JanhaviCapasso, LorenzoNagy, Andrea SzabóČapek, JanNagy, KatalinCapela E Silva, FernandoNagy, LianaCapelli, CarloNagy, Noémi M.Capitman, John AmsonNagy-Pércsi, KingaCapobianco, EnricoNahvi, AliCapobianco, GiampieroNaicker, NishaCapocasale, GiorgiaNaidenko, Olga V.Capone, VincenzaNaik, Akshata R.Caponnetto, PasqualeNaik, Vanaja KrishnaCaporusso, NicholasNair, ShakuCapote, ReneNajafi, EsmaeilCapozzi, VittorioNaji, Hassan S.Capparè, PaoloNajjar, MohammadCappella, Joseph N.Nakahara, HirokiCappello, TizianaNakai, KunihikoCapper, DanielNakai, MichikazuCappetta, MonicaNakajima, KeiCapuano, AngelaNakamoto, TetsuoCapurso, CristianoNakamura, FabioCaraballo, IsraelNakamura, GilbertoCarabella, CristianoNakamura, MasanaoCarabelli, GiuliaNakamura, MasashiCaramelo, FranciscoNakamura, MasatoshiCarandang, Rogie RoyceNakamura, MiekoCarapella, LauraNakamura, YoshiyukiCaravaca, Luis AndreuNakayama, NaomiCarballeira, EduNakayama, ShojiCarballeira, EduardoNakazawa, MinatoCarbone, MicheleNakonieczny, DamianCarboni, LuciaNalbandian, Harutiun M.Carcedo González, Rodrigo J.Nalbone, DavidCarcelén-Fraile, María Del CarmenNaldi, MaurizioCardaci, AlessioNalecz, HannaCárdenas Vélez, DavidNalepa, IrenaCárdenas, CésarNam, Ok HyungCardona, DianaNam, TaewooCardona, FernandoNam, YunyoungCardona, José RamónNamani, TrishoolCardoso, Andreia SaavedraNamatovu, FredinahCardoso, JorgeNamiot, DmitryCardoso, LuciliaNamisaki, TadashiCardoso, Sílvia AlmeidaNanayakkara, ShanikaCarestia, MariachiaraNanjappa, DeepakCarey, MelissaNano, AdelaCarey, RachelNante, NicolaCarey, ReneeNaokazu, HiroseCaridade, Sónia Maria MartinsNaoko, YamamotoCariello, Annahir N.Napal, MaríaCariello, MaricaNapoletano, ChiaraCariñanos, PalomaNapoli, ChristianCarini, CristianNapolioni, ValerioCarlan, SteveNapolitano, FrancescoCarle, FlaviaNapolitano, MariasantaCarleton, NicholasNappier, Sharon P.Carli, RaffaeleNappo, NunziaCarlin, AngelaNaranjo, ArleneCarlin, DanielleNaranjo, Jose EugenioCarlin, EmmaNaranjo-Hernández, DavidCarlin, EricNarasimhulu, Chandrakala AlugantiCarlisle, KarenNarayan, PrakashCarll, AlexNarayan, SeemaCarlos-Alberola, María Del MarNarayanan, BarathCarlos-Vivas, JorgeNarayanasamy, SureshCarlotto, SilviaNarciso, Joan OñateCarlsen, Hanne KrageNardella, ElizabethCarlson, BronwynNardini, AndreaCarlson, RossNarduzzi, LucaCarlsson, JessicaNarkhede, MayurCarlström, EricNarloch, JerzyCarmassi, ClaudiaNaro, AntoninoCarmen Ortega-Navas, Maria DelNaruishi, KojiCarmen Pichardo Martínez, MaríaNaruse, TakashiCarmignani, MarcoNarushima, MiyaCarmo, IsabelNarzisi, AntonioCarmo, João PauloNascimentoCarmona-Torres, Juan ManuelNascimento, YuriCarnagarin, RevathyNash, CarolCarnelley, KatherineNash, PoppyCarnero, Samuel FernándezNash, SallyCarnevale Miino, MarcoNasiadek, MarzennaCarney, A SimonNaskar, ShovanCarney, GregNasralla, Moustafa M.Carney, TerryNassour, AbdallahCarniel, EmanueleNassur, Ali-MohamedCarnis, LaurentNatale, AldaCarodenuto, SophiaNatale, FrancaCaron, RosemaryNatale, VincenzoCaroppo, AndreaNatalie, SuffCarosi, PaoloNatarajan, NithyaCarotenuto, MaurizioNatarajan, SivaramanCarpenter, Adam T.Nath, ArijitCarpenter, DavidNathan, KalpanaCarpentier, YvainNathan, MariaCarpentieri, AndreaNathans, LauraCarper, MichaelNaufel, MariaCarpinelli, LunaNaughton, ShaanCarpio, Antonio JoséNaugler, ChristopherCarr, LauraNaumann, ElkeCarr, Loneke BlackmanNaushad, Mu.Carrasco Polaino, RafaelNavajas, JoaquínCarrasco, AlejandroNavajas-Romero, VirginiaCarrasco, LuisNavalta, JamesCarrasco-Poyatos, MaríaNavarre-Jackson, LayanaCarreiro-Martins, PedroNavarrete-Munoz, Eva MariaCarrera Quintanar, LucreciaNavarro Ferreira, AnaCarrera, IvánNavarro Flores, EmmanuelCarrera, JenniferNavarro Pérez, José JavierCarreras, HebeNavarro, CasildaCarrier, PaulNavarro, Francisco J.Carrieri, MariellaNavarro, MaríaCarriker, ColinNavarro, RaulCarrilho, EuniceNavarro-Flores, EmmanuelCarrillo, GennyNavarro-Haro, María VicentaCarrillo, IreneNavarro-Pérez, Carmen F.Carrillo-Álvarez, ElenaNavarro-Tito, NapoleónCarrillo-Castrillo, Jesús A.Navas Macho, PatriciaCarrington, Jane MNavas Sanz, DanielCarroll, Dana MowlsNavidi, UteCarroll, Harriet A.Nawarathna, Thiloththama Hiranya KumariCarroll, Kathryn A.Nawaz, Adil MalikCarroll, SuzanneNayanatara, Arun KumarCarrouel, FlorenceNaydenov, StefanCarson, FraserNaylor, MichaelCarswell, AndrewNaylor, Patti-JeanCarter, SophieNazare, BarbaraCarteri, Randhall Bruce KreismannNazari-Sharabian, MohammadCartmill, Andrew D.Nazarko, ŁukaszCarucel Cara, Mª JesúsNcube, SomandlaCaruggi De Faria, LucianoNdayambaje, BenjaminCarús, LuisNeagoe, OctavianCaruso, GianlucaNeagu, OlimpiaCaruso, GiuliaNeale, ChrisCaruso, SalvatoreNeale, RachelCarvajal, MaríaNebe, StephanCarvajal, Thaddeus M.Nechita, FlorinCarvalho, FabioNęcka, EdwardCarvalho, FilipeNęcka, KrzysztofCarvalho, HumbertoNedeljković, MilicaCarvalho, María JoséNedjai, RachidCarver, AlisonNeeb, AntjeCasado, AlfonsoNeelakantan, PrasannaCasado, NataliaNeffe-Skocińska, KatarzynaCasado-Hernández, IsraelNegri, AttàCasado-Vara, RobertoNegrini, RobertCasado-Verdejo, InésNegy, CharlesCasais, BeatrizNehemiah, ArielCasakin, HernanNehr, SaschaCasalino, GabriellaNeill, AaronCasanova Navarro, MarianoNeiva, Henrique P.Casanovas, CarmenNejman, AlicjaCasares, MiguelNelson, BakeyahCasartelli, LucaNelson, Dorothy A.Casas, Rosa M.Nelson, Fred R. T.Case, BruceNemat, BabakCasella, GiovanniNemec, JurajCasey, JenniferNemer, MaysaaCasey, JoanNemery, BenoitCasey, Joanna GordonNemeș, Roxana MariaCasey, PatriciaNémeth, LászlóCasperson, Shanon L.Nemeth, Lynne S.Cassady, Steven J.Nena, EvangeliaCassarino, MaricaNenna, AntonioCassetta, MicheleNeocleous, GregoryCassidy, SophieNepal, SmritiCassilhas, Ricardo CardosoNepomuceno, Erivelton GeraldoCastagna, JessicaNeri, AlessandraCastaldi, SilvanaNeri, CaterinaCastaldo, AttilioNerini, AmandaCastaman, GiancarloNesbit, RachelCastañeda, ArkaitzNesbitt, DanielleCastañeda, ErnestoNess, OttarCastañeda-Chávez, María Del RefugioNess, ValerieCastañeda-Miranda, AlejandroNestares Pleguezuelo, Maria TeresaCastanho, Rui AlexandreNesterkina, MariiaCastejón, Juan LuísNesteruk, IgorCastel, AlanNesticò, AntonioCastellani, John W.Nestler, UlfCastellano Paulis, JulenNeter, EfratCastellano, JulenNeto, VictorCastellanos, Miguel ÁngelNetto, KevinCastelletti, NoemiNettore, Immacolata CristinaCastelli, DarlaNeubauer, DeaneCastelli, LuciaNeubauer, EvaCastelli, VanessaNeuberger, ManfredCastellví, JordiNeufeld, Hannah TaitCastiglione, ArcangeloNeuhuber, Winfried L.Castilla, DianaNeuman, YairCastillo Carrion, MercedesNeumann, EwaldCastillo, Carlos A.Neumann, TimCastillo, DanielNeuner, BrunoCastillo, IsabelNeupane, SubasCastillo-Rodríguez, AlfonsoNeves, AngelaCastillo-Villar, FernandoNeves, JoãoCastonguay, JessicaNeves, RuiCastrejón-Pérez, Roberto CarlosNevi, LorenzoCastren, MaijaNevidomskaya, Dina G.Castro Alves, RicardoNewaz, MohammadCastro David, Luiz HenriqueNewbury, JoanneCastro Gracia, Adoración CarmenNewby, DanielleCastro Quezada, ItandehuiNewhouse, IanCastro Ramirez, CandidaNewins, AmieCastro, Flávio Antônio De SouzaNewman Young, BarbaraCastro, HenriqueNewman, EricaCastro, Juan C.Newman, John F.Castro, Luis A.Newman, Steven E.Castro, Maria AntónioNewman, Winifred E.Castro, PaulaNewsome, CheyenneCastro, Paulo CesarNewson, LisaCastro, TelmaNewton, MariaCastro-Colin, MiguelNewton, ValerieCastro-Méndez, AuroraNexo, EbbaCastro-Sanchez, ManuelNg, AlexCastro-Sepulveda, MauricioNg, AshleyCastuera, Ruth JiménezNg, Ding-QuanCasu, GavinoNg, EricCasu, GiuliaNg, Isabella F. S.Casucci, CristianoNg, JackyCasula, MilenaNg, JohanCasuso-Holgado, Maria JesusNg, Ka ShingCaswell, BessNg, Kheng SiangCatalán, Ángel AbósNg, KwokCatalano Iniesta, LeonardoNg, NormanCatalano, AntoninoNg, Qin XiangCataldi, StefaniaNg, Shu Kay AngusCatania, GianlucaNg, Ting KinCatapano, MichaelNg’ombe, John NedsonCatchlove, SarahNghiem, SonCatela, DavidNgin, ChanrithCates, DianaNgonghala, CalistusCatherine, HayesNgoubene-Atioky, Arlette J.Catlow, SarahNgoy, KikomboCatros, SylvainNguyen, Anh (Bao)Cattuzzo, Maria TeresaNguyen, Duc-HiepCatunda, CarolinaNguyen, Joe TruongCaudle, MichaelNguyen, KienCauli, OmarNguyen, Ly Sy PhuCausby, RyanNguyen, MinhCava, María-JesúsNguyen, Minh HoangCavacas, Maria AlziraNguyen, NhungCavagnetto, DavideNguyen, QuynhCavalcante Neto, Jorge LopesNguyen, Thu T.Cavalcanti, Yuri WanderleyNguyen, TranCavaleiro Rufo, JoãoNguyen, TrungCavaleri, DanieleNguyen, Van KhanhCavallaro, LucaNguyen-Truong, DawnCavalli, LoredanaNgwube, AlexanderCavallo, AuroraNi Lochlainn, MaryCavallo, EugenioNí Shé, ÉidínCavallo, FedericaNi, JunCavanaugh, DanielNi, JunjunCavas-Martínez, FranciscoNiavis, SpyrosCave, BenNibali, LuigiCave, MarkNiccolai, AlbertoCavia Naya, VictoriaNicholas, AngelaCavicchi, CaterinaNicholas, OwenCaviglia, CaterinaNicholson, John W. Caviglioli, GabrieleNicholson, LauraCavoretto, PaoloNickelsen, Niels Christian MossfeldtCawood, SallyNicklas, JacindaCayón De Las Cuevas, JoaquínNicklin, Laura LouiseCazcarro, IgnacioNicodemo, GianfrancoCazzolla, Angela PiaNicolaides, StevenCè, EmilianoNicolaou, ConstantinosCeballos-Laita, LuisNicolas-Silvente, Ana IsabelCebrià I Iranzo, Maria Dels ÀngelsNicolau, AnaCebrino Cruz, JesúsNicolau, CristinaCebula, JanNicolau, Juan LuisCeccato, VaniaNicole, GrünCeccatto, Vânia MarilandeNicolescu, Cristina MihaelaCeccherini, Maria TeresaNicolò, GiuseppeCecchi, FrancescaNiculita-Hirzel, HélèneCecchini, ArnaldoNie, DayongCecconet, DanieleNie, FanhaoCecilia, Juan AntonioNie, JinleiCeciliani, AndreaNie, LimingCedano, Karla G.Nie, PengCedeño, David L.Nie, ZichaoCedó, LídiaNiebel, DennisCedro, AnnaNieć, JakubCedrone, FabrizioNiedbała, GniewkoCefalo, RaffaelaNiedermeier, MartinCegiełka, AnetaNiederseer, DavidCegielska-Radziejewska, RenataNiedziółka, PawełCeicyte, JolitaNiedźwiedzka-Rystwej, PaulinaCejudo, AntonioNielen, MarkCelakovska, JarmilaNielsen, KariCelaya-Padilla, José M.Nielsen, MichaelCeleiro, Iván De RosendeNielsen, PetterCeleste, Roger KellerNiemi, MariaCelicourt, PaulNieminen, PenttiCelińska-Janowicz, DorotaNiemitz, Jeffrey W.Cella, Silvano GabrieleNienaber-Rousseau, CornelieCellini, NicolaNierostek, LechCenik, BasarNiessner, ClaudiaCeptureanu, Sebastian IonNieto Fontarigo, Juan JoseCerankowski, K. J.Nieuwenhuijsen, KarenCerchione, RobertoNievas Soriano, Bruno JoséCerda, ArtemiNieves, KourtneyCerda, BernardinoNiewiadomska, EwaCerejeira, JoaquimNifli, Artemissia-PhoebeCeresia, FrancescoNigam, Jeannie A. S.Čereška, AudriusNigg, Claudio R.Cerino, StefaniaNighbor, Tyler D.Cermakova, PavlaNigra, Anne E.Cerna, MiloslavaNijdam, Niels AlexanderCernasev, AlinaNijim, MaisCerniglia, LucaNiklasson, JohanCernuda, José A.Nikodem, MaciejCeron Breton, Julia GriseldaNikolaeva, NadezdaCerri, CesareNikolaidis, Pantelis T.Cerruto, EmanueleNikolaos, KoutlianosCerutis, Delie RoselynNikolaou, Charoula KonstantiaCervera-Garvi, PabloNikolaou, DimitriosCervera-Gasch, ÁguedaNikolaou, EleniCervino, GabrieleNikolett, KállaiCerwén, GunnarNikolic, DejanCeulemans, MichaelNikolić, MilicaCeyhan, SercanNikolopoulos, DimitriosCezar-Vaz, Marta ReginaNikolopoulou, KleopatraCha, Seung-EunNikolova, NataliaChaber, RadosławNikulchev, EvgenyChaberek, GrazynaNilsen, Lill Tove N.Chabrillat, SimonNilson, Eduardo A. F.Chacartegu, ConsueloNilsson, KjellChacin-Suarez, AudryNilsson, StefanChacón Cuberos, RamónNiñerola, AngelsChacón-Borrego, FátimaNiño-Ruiz, Elias DavidChaddad, AhmadNiort, JannickChadwick, PhilipNirmalkar, KhemlalChadwick, RuthNishi, DaisukeChae, SoosangNishi, MayumiChai, HongyanNishi, NobuoChai, LiNishida, KeiChai, YanweiNishide, ShinyaChakraborty, AmlanNishijo, MunekoChakraborty, ShamikNishikawa, YuichiChakraborty, SudipNishimura, TomokoChalah, Moussa AntoineNishimura, TsutomuChalkley, AnnaNishio, HisahideChalmin-Pui, Lauriane SuyinNishioka, ShintaChaloupková, HelenaNissan, JosephChałupnik, StanisławNisse, CatherineChamarro, AndresNissen Bjørnsen, HanneChambers, MaryNitecka-Buchta, AleksandraChami, GoyletteNitzan, DoritChampion, KatrinaNitzkin, JoelChamut, SteffanyNiu, LeiChan, Alan H. S.Niu, ShifengChan, Benny Kwok KanNiu, YuanChan, CatherineNivedita, NiveditaChan, Chien-LungNivolianitou, ZoeChan, ClementNixon, Daniel W.Chan, DanielNjabo, Kevin YanaChan, DerwinNjoku, AnuliChan, Ho YinNjororai, FletcherChan, Hsun-YuNjororai, Wycliffe W. SimiyuChan, JohnnyNkenke, EmekaChan, Ka HungNkogo, Joselyne ChenaneChan, Ko-LingNkosi, VusumuziChan, Kuei-HuiNlenanya, InyaChan, Ngan YinNnaji, ChukwumaChan, Ping-LungNobile, StefanoChan, PuthearathNoble, FionaChan, SteveNoble, HelenChan, WayneNoblin, AliceChan, Ying TungNobmann, Elizabeth D.Chan, Yong-JietNocera, SilvioChand, Hitendra SinghNodari, ChristineChand, Mohan BahadurNoé, EnriqueChanda, AnindyaNofal, SalahChander, HarishNogami, MakotoChandler, Joshua D.Nogueira, Oscar RodriguezChandra Putra, HandiNoh, SoowoongChandra, Boggarapu PraphullaNohr, DonatusChandra, PrakashNojiri, ShukoChandran, ArunaNolan, ClodaghChandrasekaran, AkshayaNolan, MaryChandrasekaran, MurugesanNolasco, AndreuChandrasekaran, Srinivas NiranjNold, JulianChaney, Beth H.Nolen-Doerr, EricChang, AngelaNoll, MatiasChang, Chee-JanNoll-Hussong, MichaelChang, Chen-KangNolwenn, Le MeurChang, Chia-chiaNomngongo, Philiswa NosizoChang, Chih-ChingNomura, KyokoChang, Ching-ChihNonaka, DaisukeChang, Ching-HsunNonami, AtsushiChang, Ching-WenNoone, ChrisChang, Chung-TeNora-Krukle, ZaigaChang, Chun-YenNorbedo, StefaniaChang, Geng-RueiNordbø, Emma Charlott AnderssonChang, HyunNoren, HasmunChang, IkyoungNorful, Allison A.Chang, Jer-HaoNoriega, CristinaChang, Jia-FengNorman, TrevorChang, JianxiaNorman-Burgdolf, HeatherChang, JongwhaNorris, LukeChang, Ke-VinNorström, FredrikChang, Liang-ChihNorte Navarro, Aurora IsabelChang, Nai-JenNorthcross, AmandaChang, PingNorval, MaryChang, Po-JuNosa, Vili HapakiChang, Po-YaNoskov, AlexeyChang, Sei-JinNosrat, SanazChang, Sheng-HsiungNoszczyńska, MagdalenaChang, Shin-TsuNota, AlessandroChang, Ta-YuanNoto, GuidoChang, Wei-JenNotton, GillesChang, Won WonNounagnon Frutueux, AgbanglaChang, XuhongNourazari, SaraChang, Ya-JuNoushi, NioushahChang, Yi-KuoNovačko, LukaChang, Yuan ShiunNovak Kujundžić, RenataChang, Yu-ChanNovak, NicoleChang, Yu-ChaoNovak, PriscillaChang, Yun-HsuanNovak-Zezula, SonjaChang, YunjeongNovelli, GiuseppeChang, Yun-KeNovello, GiorgiaChangfoot, NadineNovick, Richard P.Chantal, VerdonNovikova, IrinaChao, Chia-TerNovío, SilviaChao, Chien-ChungNovita, ShallyChao, Chien-MingNovo, AndréChao, Ren-FangNovo, LuísChao, Stessa Tzu-YuanNovotná, MarkétaChao, YingyuNovo-Veleiro, IgnacioChaparro, Teresa SanchezNowacka-Jechalke, NataliaChaplin, John EricNowaczewska, MagdalenaChapman, CathNowak, AnnaChapman, Christopher L.Nowak, GlenChapman, DaleNowak, JacekChapman, JeremyNowak, Michal S.Chapman, KatyNowak, PawelCharach, GideonNowak, Paweł F.Charalampopoulos, LoannisNowak, Paweł FryderykCharao, Mariele FeifferNowak-Chmura, MagdalenaCharizopoulos, NikosNowakiewicz, AnetaCharkiewicz, Angelika EdytaNowakowska, AnnaCharles Ryan, JohnNowakowska-Lipiec, KatarzynaCharles, LuendaNowicki, GrzegorzCharleston, MichaelNowicki, MichałCharlot, KeyneNozohouri, SaeidehCharlotte, Wendelboe-NelsonNubani, LindaCharman, William NeilNübling, MatthiasCharmas, MalgorzataNübling, RüdigerChasin, MarshallNucci, LuciaChastin, SebastienNucci, Luciana B.Château, Pierre-AlexandreNucera, EleonoraChatterjee, IshitaNucera, RiccardoChatterjee, SatabdiNugent, AnneChatterjee, SubhasriNukpezah, JuliusChattopadhyay, SurajitNunbogu, Abraham MarshallChatwood, SusanNunes, Everson A.Chatzifotiou, SevasteNunes, LeonelChatzitheodoridis, FotiosNunes, Leonel Jorge RibeiroChau, Alex M. H.Nunes, SaraChau, KénoraNunes, TeresaChau, Kwok-wingNúñez Enríquez, OscarChaudhari, SarikaNúñez Pérez, José CarlosChaudhary, Dhiraj KumarNuñez, AngelChaudhri, VirendraNuñez, PaulaChauhan, Madhulata SinghNúñez, Remigio ParadeloChauhan, MunishNúñez-Rocha, Georgina MayelaChauhan, NeerajNurchi, Valeria MChaurasia, Akhilesh KumarNurunnabi, MohammadChaushu, GavrielNurwulan, Nurul RetnoChaussé, PierreNusca, AnnunziataChavan, PrachiNuss, Henry J.Chavarria, EnmanuelNutile, SamuelChaves, Luis FernandoNuviala-Nuviala, AlbertoChaves, PaulaNuzzo, James L.Chavez, Adela OlivaNyangweso, MaryChavez, FranciscoNych, AlicjaChavez-Santoscoy, Rocio AlejandraNycz, Jacek E.Chávez-Tapia, Norberto C.Nyein Aung, MyoChawla, AseemNyende, HawaChe, XiaoyuNyholm, MariaChe, ZhengpingNyirenda, GibsonCheba, KatarzynaNylander, KarinChebli, YoussefNyman, DagChecchi, VittorioO Connor, DeirdreCheema, Muhammad A.O’ Connor, DominicCheemarla, Nagarjuna ReddyO’Brien, AprilChehri, AbdellahO’Brien, JaneChehroudi, BabakO’Brien, KerryChell, KathleenO’Brien, NiamhChellan, NireshniO’Brien, PaulaChelli, Francesco MariaO’Connor, Elizabeth A.Chelsea, SpencerO’Connor, KarlChen, AiminO’Donnell, RachelChen, BoO’Donovan, KristinChen, ChaonaO’Dwyer, JeanChen, ChaoyangO’Dwyer, SiobhanChen, ChenyuanO’Grady, MathewChen, Chia-chengO’Hagan, JohnChen, ChiachungO’Hare, AmindaChen, Chia-YuO’Hern, SteveChen, Chieh-fuO’Leary, SimonChen, Chien-ChangO’Loughlin, FiachraChen, Chien-LiangO’Neal, HollisChen, Chih ChungO’Neal, SuzanneChen, Chi-NienO’Reilly, MaryChen, Chiung YaoO’Shea, AmyChen, ChongO’Sullivan, Amy K.Chen, DanhongO’Sullivan, KimberleyChen, DechanO’Sullivan, Mary M.Chen, FeiyuOakes, JaneChen, Feng (China)Oakey, ZackeryChen, Feng (UK)Oancea, S. CristinaChen, GongOats, ReneeChen, Han-ShenOba, ShinoChen, HongshengObafemi, OlukoyaChen, HuiweiObara-Michlewska, MartaChen, Hung-YuObeng-Gyasi, EmmanuelChen, James K. C.Ober, JózefChen, James MingOberbarnscheidt, ThersillaChen, Jau-erOberman, TinChen, Jen-TsungOberst, UrsulaChen, JiangangObeso, AnaChen, JingObih, PatienceChen, JingfengOblad, TimothyChen, Jin-ShuenObolski, UriChen, Jui-ShengObrenovic, BojanChen, JulianaObrero Gaitán, EstebanChen, JunfeiObruca, StanislavChen, Kao-ChinObuchi, ChieChen, KatherineOchia, RuthChen, KejieOchiai, HirokoChen, Kow-TongOchiai, RyujiChen, Kuan-FuOchnik, DominikaChen, Kuo-HuOchoa, Alexander AchalandabasoChen, Kuo-ShingOchoa, JulioChen, LikwangOchoa-Brust, AlbertoChen, LiliOchoa-Leyva, AdrianChen, Li-ShengOchoa-Zezzatti, AlbertoChen, LiwenOchsner, MichaelChen, Mei-LanOczkowski, MichałChen, Mei-YenOda, YoichiroChen, MengOdeh, MajedChen, MindongOdei, JamesChen, MingOdemer, RichardChen, MinjianOdinea Maria, Amorim BatistaChen, NanOdland, JonChen, NingÖdman, AnnaChen, Pei-ShihOdo, NnaemekaChen, PengyuOdom-Forren, JanChen, Ping-KuoOdunitan-Wayas, FeyiChen, QihuiOe, MisariChen, RuolingOechsle, KarinChen, Ru-SiOehring, DanielaChen, ShanquanOfei-Manu, PaulChen, ShaohuaOffiong, Nnanake-AbasiChen, Shih-ChiehOffringa, Ite A.Chen, Shih-KuoOftedal, ElinChen, SiluOftedal, Elin MeretheChen, SimingOftedal, StinaChen, Si-TongOgasawara, IsseiChen, Song ChaoOgbe, EmmanuelChen, SuiyaoOgeil, RowanChen, Szu-ChiaOgen, YaronChen, TianhuaOgletree, ScottChen, TingguiOgneva-Himmelberger, YelenaChen, TingtingOgonowska-Slodownik, AnnaChen, Tsung-PoOgórek, RafałChen, Tzu-LingOgra, AurobindoChen, Vincent C. W.Ogrodowczyk, AnnaChen, Wan-JiunOgryzek, MarekChen, WeiOgunjirin, AdebowaleChen, Wei TongOh, ByeongsangChen, WeihongOh, InhaChen, WeiliangOh, Je HyeokChen, Wei-YuOh, Ju-HyeonChen, WenhaoOh, SeieunChen, XiOh, Yong HwaChen, XiaoyanOh, YounjaeChen, XinOhaja, MagdalenaChen, XingchengOhba, SeigoChen, XingjuanOhira, AkihiroChen, XuOhki, MasafumiChen, XueweiOhlendorf, DanielaChen, YaoningOhlmeyer, MartinChen, YewangOhno, JunChen, Yi-ChunOhsawa, YukioChen, Yih-SharngOhta, MitsuakiChen, Yi-JenOhta, RyuichiChen, Yi-LangOhta, TakahisaChen, Yi-LungOjeda-Benitez, SaraChen, YimingOjha, ChandrakantaChen, Yi-TuiOjima, KoichiChen, Yi-WenOjio, YasutakaChen, Yu-ChunOjo, MercyChen, Yueh-ChihOjo, PeterChen, Yulin (China)Ojong, NathanaelChen, Yulin (Taiwan)Okada, HiroshiChen, Yung-ShengOkada, SeigoChen, Yu-ShanOkaiyeto, KunleChen, YutingOkajima, HideakiChen, Yu-TingOkamoto, MotokiChen, ZhaoOkamura, KazukoChen, ZhongbingOkayama, MasanobuChen, ZhongzhiOkino, Cintia HiromiChen, ZhuoOkoro, ChiomaCheng, Chin-MinOkoro, Olihe N.Cheng, Chun-AnOkrzymowska, PaulinaCheng, Fu-JenOksanen, AtteCheng, HaoOkubo, YoshiroCheng, Hong ShengOkuda, MasayukiCheng, HongguangOkulicz-Kozaryn, KatarzynaCheng, HuanyuOkumura, JiroCheng, Hung-ChiOkunade, Adewole L.Cheng, Juei-TangOkuno, KentaroCheng, Jung-LangOkut, HayrettinCheng, Kai-WenOlabe Sánchez, Pablo JavierCheng, Kuang-HungOlafsdottir, AnnarCheng, QuOlague, José TrinidadCheng, QunkangOlah, BranislavCheng, ShibinOlcina, GuillermoCheng, ShixiongOlejniczak, DominikCheng, Su-FenOleniacz, RobertCheng, Teng YuanOleńska, EwaCheng, Tsang-HsiangOleschko Lutkova, KlavdiaCheng, WeimingOleskow-szlapka, JoannaCheng, WeiyingOleson, James C.Cheng, Ya-YunOliart-Ros, Rosa MaríaCheng, Yu-HsiangOligbu, GodwinCheong, Kang HaoOlišarová, VěraCheong, TaesuOliva Lozano, José MaríaChepeha, JudyOliva Pacual-Vaca, AngelCherchi, MarcelloOliva, ImmacolataCherlin, SvetlanaOliva, JoséCherng, Chen-HwanOliván-Gonzalvo, GonzaloChernyak, SergeiOliva-Pascual-Vaca, JesúsCherrie, JohnOlivares, FernandoCherry, Mark J.Olivares-Olivares, Pablo JoséCherukupalli, RajeevOlive, XavierCheruzel, LionelOliveira Henriques, CarlaCheshmehzangi, AliOliveira Júnior, José Max BarbosaChetty, LaranOliveira, AmílcarCheu, HoiOliveira, AnaCheung, AmandaOliveira, AndréCheung, JasmineOliveira, CidaliaCheung, Lewis Ting OnOliveira, Cláudia SirleneCheung, Man KitOliveira, José Egídio BarbosaCheung, Peggy Pui YeeOliveira, LucianaCheung, Rebecca Y. M.Oliveira, MartaCheungpasitporn, WisitOliveira, Paula A.Chevret, SylvieOliveira, Ricardo BrandãoChew, Song FohOliveira, RúbenChi, Ching-ChiOliveira, Rui S.Chi, GeraldOliveira, Sonia M.Chi, Wen-ChouOliveira, TiagoChianese, ElenaOliveira-Filho, Aldemir B.Chiang, Chien-HsiehOliveira-Filho, José P.Chiang, Long Y.Oliver, AmparoChiang, Meng-TsanOliver, DavidChiang, Yung-HsiaoOliver, ShünéChiappedi, MatteoOlivera, LeningChiaramello, EmmaOliveto, GiuseppeChiarelli, Fabio MatosOlivieri, Juan GonzaloChiarotti, FlaviaOlkiewicz, MarcinChiba, RieOller Jr., John W.Chiciudean, GabrielaOlmedilla, AurelioChiefari, EusebioOlmedillas, HugoChien, Chun-RuOlmedo, PabloChien, Lung-changOlmos Gómez, María Del CarmenChien, Ming-HsienOlmos, Alex A.Chiesa, MariaOlolade, OlusolaChiesa, ScottOlolade, OlusolaoChiew Hong, NgOlomu, IsokenChigbu, De Gaulle I.Olorunsaiye, Comfort Z.Chih, HuijunOloso, Nurudeen OlalekanChikafu, HerbertOlsen, JonathanChikte, UsufOlsen, KirkChild, StephanieOlson, KristineChildress, Marcia D.Olson, MichaelChileshe, NicholasOlson, Michael W.Chilibeck, PhilOlsson, LennartChillón Garzon, PalmaOlsson, M. CharlotteChim, Hui QingOlszańska, Anna MariaChin, Kok YongOlszewska, AnetaChin, TachiaOlszewska-Guizzo, Agnieszka AnnaChinchilla-Rodríguez, ZaidaOltra, ElisaChinnappan, MahendranOlushola, JoyceChinnasamy, AlagesanOluwole, OluwafemiChiodo, EmilioOmar, Syed HarisChiou, Tai-YingOmatsu, TsutomuChiou, Wen-KoOmboni, StefanoChipps, JenniferOmiya, TomokoChirico, FrancescoOmur-Ozbek, PinarChirumbolo, SalvatoreOnagbiye, SundayChirwa, Evans M. NkhalambayausiOncioiu, IonicaChirwa, Sanika S.Ondruš, DaliborChitale, ShalakaOnete, MarilenaChitimia-Dobler, LidiaOneto, LucaChitraju, ChandramohanOnetti, WanesaChiu, Ching-JuOng, Chong YauChiu, Chuang-YuanOng, Ken Ing CherngChiu, Hsiu-ChingOngena, YfkeChiu, Hua-ShengOniga, Smaranda DafinaChiu, Ping-FingOnik, GrzegorzChiu, Po-ChihOnisor, FlorinChiu, Po-ShengOnnis, Leigh-AnnChiu, Sheng-YiOno, YukoChiu, WeishengOnoda, HiroshiChiu, Yi-HanOnofrei, Roxana RamonaChiu, Yong-HuOnyango, ElizabethChiva-Bartoll, ÓscarOoi, KennethChlubek, DariuszOosthuizen, JaquesChmielewska, DariaOperacz, AgnieszkaChmielewska, Marta EwaOpioła, WojciechChmielinski, PawelOpoka, WlodzimierzChmielowiec, KrzysztofOpoku-Acheampong, AudreyChmura, PawełOppi, ChiaraCho, Do YeonOpstad, LeivCho, HaeryunOpydo-Szymaczek, JustynaCho, HuidaeOr, Pui LaiCho, Hyun-JaeOramas-Royo, SandraCho, JamesOrand, AbbasCho, Jeong-HyungOrandi, SanazCho, JoonmoOrchard, LisaCho, Kang SuOren, DanCho, Kyung-HwanOrgambídez-Ramos, AlejandroCho, Sang-ChoonOrian, LauraCho, SeongkwanOriente, FrancescoCho, Seung-HyunOris, MichelCho, YounghyunOriyama, SanaeChoa, Fow-SenOrlacchio, ArturoChoate, Peter W.Ormsbee, FloydChocano-Bedoya, PatriciaOrnelas, YoshiraChodakowska, EwaOrnelas-Soto, Nancy EdithChoi, DonghoOrnelas-Van Horne, YoshiraChoi, Edmond Pui HangOrner, KevinChoi, GiehaeØrngreen, Mette CathrineChoi, HongjoOropesa Ruiz, Nieves FátimaChoi, HyoOropeza-Perez, IvanChoi, Hyung JinOrsini, GiovannaChoi, Jane RuOrsolini, LauraChoi, Jeong-DongOrtega Barón, JessicaChoi, Jin YoungOrtega, EnriqueChoi, JinkyungOrtega, JohnChoi, Jun H.Ortega, José MarcosChoi, MineeOrtega, María-isabelChoi, MyeongjinOrtega-Campos, ElenaChoi, Pak-SingOrtega-Galán, Ángela MaríaChoi, Peggy H. N.Ortega-Gaspar, MartaChoi, SangchunOrtega-Sánchez, DelfínChoi, Seong-SooOrtega-Torres, EnricChoi, Siu-MingOrtiz, Andrea MonicaChoi, Suk BongOrtiz, AndricChoi, Sung-HwanOrtiz, María CristinaChoi, SungyongOrtíz-Jiménez, LuisChoi, Won HyungOrtmeyer, Heidi K.Choi, Won SukOrtolano, SaidaChoi, Won-JunOrttung, Robert W.Choi, Yoon JeongOrui, MasatsuguChoi, YoungnaOrusa, RiccardoChoi, Youn-HeeOrvis, Kathryn S.Chokkanathan, SrinivasanOryani, BaharehCholewa, JarosławOrzolek, Michael D.Chomyszyn-Gajewska, MariaOsborne, John O.Chong, Kai LeongOsganian, Stavroula K.Chong, Ngee SingOsgood, Jeffrey M.Chong, Poh HengOsier, NicoChoo, Pei LingOskarbski, JacekChoonara, ImtiOsmani, Ahmad ReshadChopik, William J.Osorio, CarlosChotta, Nikolas A. S.Osório, HugoChou, Che-YiOsowiecka, KarolinaChou, Pang-YunOspina Betancurt, JonathanChou, RogerOster, CandiceChou, Tzu-ChiehØsthus, StåleChou, Yu-ChingOstiguy, NancyChoudhuri, AvikOstini, RemoChover-Sierra, ElenaÖstlund, BrittChow Peggy, Pik KeiOstoa-Saloma, PedroChow, Amanda FroehlichOstroumova, EugeniaChow, Bik ChuOstrowska, BożenaChow, DannyOstrowska, LucynaChow, DavidOstróżka-Cieślik, AnetaChow, Simon Kwoon-HoOsuna, EduardoChow, Winnie S.Oswald, JohnChowdhury, FarhanOszako, TomaszChowdhury, SourangsuOta, AtsuhikoChrcanovic, BrunoOtaki, MasahiroChrisinger, BenjaminOtamendi, Francisco JavierChrisman, MatthewOtanez, MartyChristensen, JonasOtani, HidenoriChristenson-Diver, ElizabethOtani, ShinjiChristian, Colmore S.Otazo Sánchez, Elena MaríaChristiana, RichardOteri, GiacomoChristides, TatianaOtero, CarolinaChristie, NicolaOtero, Patricia OteroChristoph, MaryOtero-Agra, MartínChristopher, LambOtero-López, José ManuelChrobak, ElwiraOtero-Saborido, Fernando ManuelChromy, DavidOtis, Melanie D.Chroust, Alyson J.Otoum, SafaChruściel, PawełOtremba, ZbigniewChrysanthakopoulos, Nikolaos A.Otsuki, TakemiChrysikou, EvangeliaOtt, ThomasChrzan-Dętkoś, MagdalenaOttarsdottir, UnnurChtourou, HamdiOtto, PhilippChu, Chun HungOu, Chun-QuanChu, Daniel K. W.Ou, DayiChu, Dinh ToiOu, JudyChu, LanlanOuaret, RachidChu, RayOuimet, FrédéricChua, Koon Jiew EriOuko, JohnChua, Tze-ErnOuyang, ChunpingChua, WinnieOuyang, Xiao-kunChuang, DengminOuzouni, ChristinaChuang, Hai-HuaOvalle, María AntoniaChuang, Hung-YiOvaska, TomiChuang, Kai-JenOviedo-Caro, Miguel AngelChuang, Shu-ChunOwada, KeiChuang, Ting-WuOwari, YutakaChuang, Yao LiOwen, LaraChubko, NadezhdaOwen, RobertChui, Kwok TaiOwili, Patrick OpiyoChukhrova, NataliyaOxford, SamChulvi-Medrano, IvánOxford, SamuelChun, Jong TaiOyanadel, CristiánChung, Cheng-ShiuOyelade, TopeChung, Dau-ChuanOzamiz-Etxebarria, NaiaraChung, Esther K.Ozbil, AyseChung, Ho JinOze, IsaoChung, Hsin-FangOzga, AndrewChung, JibumOżga, WojciechChung, Kyeong WooOzguven, Eren ErmanChung, Kyung-YongOzodiegwu, IfeomaChung, Ming YanOzone, SachikoChung, Rebecca H.Pääkkönen, RaunoChung, Sae WonPaal, PeterChung, Shan-ShanPaas, FredChung, Yang WoonPabayo, RomanChung, Yat-NorkPablo-Martí, FedericoChung, Yong SukPablos, AnaChung, YouchungPac, AgnieszkaChung-Hsiung, HuangPacana, AndrzejChurch, DavidPacchi, CarolinaChurro, CatarinaPace, AnnalisaChutiyami, MuhammadPach, GrzegorzChwałczyńska, AgnieszkaPacheco, Susan E.Chye, YannPaches, MariaChylińska, JoannaPaci, ChrisChyrchel, MichałPaciência, InêsChytrý, VlastimilPacifici, AndreaCiaburro, GiuseppePacifico, AntonioCialdea, DonatellaPacifico, Francesco M.Ciaramitaro, VivianPacitto, AntonioCiarcia, RobertoPacoal, PatríciaCiarleglio, AdamPacurari, MaricicaCiarreta, AitorPączka, GrzegorzCiazela, JakubPadilla, EmilioCibella, FabioPadilla, Heather M.Ciccarelli, MariaPadilla-Eguiluz, Norma G.Ciccarese, GiuliaPadilla-Rivera, AlejandroCicchella, AntonioPadovese, ValeskaCicciù, MarcoPadua, LuisCicco, SebastianoPaduano, StefaniaCiccone, MarcoPadula, Antonio DomingosCiccone, Marco MatteoPadula, WilliamCicero, ArrigoPadulo, JohnnyCichero, ElenaPaek, DomyungCichowska-Kopczyńska, IwonaPaes, Marciano VianaCiciolla, LuciaPagan, RicardoCiciurkaite, GabrielePaganini, MatteoCicolin, AlessandroPagano, FrancescaCid, LuisPagano, StefanoCiekot-Sołtysiak, MonikaPaganoni, Anna MariaCienfuegos-Suárez, PabloPaggiaro, PierluigiCieplak, TomaszPagliardini, LucaCiesielska, AnnaPaglione, LorenzoCieśla, AgnieszkaPagnini, FrancescoCieślik, BłażejPahwa, RomaCieślińska, AnnaPai, Chih-WeiCieślińska, BarbaraPai, Fan-YunCifuentes, Carlos A.Paindavoine, MichelCigrovski, VjekoslavPaineiras-Domingos, Laisa LianeCihacek, LarryPainho, MarcoCihon, TraciPainschab, MatthewCilia, GiovanniPaixão Cansado, Isabel PestanaCimadoro, GiuseppePaixão, Maria MargaridaCimolin, VeronicaPaixão, SusanaCingolani, MarianoPajardi, DanielaCingranelli, DavidPajares, Encarnación MoralCinzia, AlbanesiPak, Tae-YoungCioffi, IolandaPakdel, EsfandiarCioffi, MarceloPakhale, SmitaCiommi, Maria TeresaPakpour, SepidehCirer-Sastre, RafelPakseresht, AshkanCirillo, MarcoPal Choudhuri, ShreoshiCirovic, GoranPal, DebjaniCisek, MarcinPalacios, Eider GoñiCisilino, FedericaPaladino, SimonaCiski, MateuszPalakurthi, BhavanaCissoko, MadyPalamarchuk, Roman S.Cisterna, PedroPalamuleni, LobinaCiszewski, Wojciech MichałPalani, HariCiszkiewicz, AdamPalaskas, TheodosiosCitelli, MartaPałasz, ArturCivit-Balcells, AntonPalatnik, Ruslana RachelČižiūnienė, KristinaPalazón-Bru, AntonioClaborn, DavidPalazzolo, Dominic L.Claes, JommePalella, Boris IgorClair, AmyPalermi, StefanoClairmont, LindseyPalestini, PaolaClanchy, FelixPaletto, AlessandroClancy, JoyPaliokas, IoannisClapp, RichardPalioura, SotiriaClapton, GaryPalladini, GiuseppinaClari, MarcoPalladino, RaffaeleClark, CharlottePallag, AnnamáriaClark, KimPallarés, Jordi TousClark, MalissaPallmann, PhilipClark, NancyPallotti, FrancescoClark, SeanPalma Sircili, MarceloClark, SuzannePalma, AlexandreClark, Terryann CoraliePalma, MonicaClark, VanessaPalma, PauloClarke, Anthea C.Palma, Paulo JorgeClarke, George R. G.Palmer, CliveClarke, LindaPalmer, DebraClarke, NeilPalmero, FranciscoClarkson, LarissaPalmier, Andrea ClementeClaros, EdithPalmieri, AriannaClas-Håkan, NygårdPalmisani, JolandaClaudia, Delgadillo PugaPalombi, LauraClaudino, JoãoPalomino-Moral, Pedro ÁngelClavenna, AntonioPalomo López, PatriciaClay, LucasPalomo-Toucedo, Inmaculada C.Clayton, Zachary S.Palos-Sánchez, Pedro R.Cleare, SeonaidPalotas, AndrasCleary, PatriciaPalou Sampol, PereClement, JanPalou, PereClemente, Filipe ManuelPalumbo, RoccoClemente, MatteoPamphlett, RogerClemente-Suárez, Vicente JavierPan, FeiClemes, StacyPan, HaozhiClennin, Morgan N.Pan, HongyingCliff, DylanPan, JiazhuClifton, PeterPan, LinlinClimstein, MikePan, MiClouthier, Allison L.Pan, MingfeiCloutier, DenisePan, XiCluff, LauriePan, XiaoyueCluley, VictoriaPan, XuejunClutterbuck, RyanPan, YijunCoates, RosePan, YunCoban, OksanaPan, ZhihuaCobb, SharonPanagiotopoulos, PanagiotisCobo, María José GutiérrezPanagopoulos, ThomasCobo-Cuenca, AnaPanagopoulos, YiannisCobos-Puc, LuisPanait, MirelaCobos-Sanchiz, DavidPanaite, VanessaCocca, ArmandoPanarello, DemetrioCocco, PierluigiPancotto, FrancescaCochrane, JodiePanda, BibhuduttaCoco, MarinellaPanda, ManojCocoradă, ElenaPandey, ManishCocuzza, SalvatorePandey, PramodCodella, RobertoPandey, SumaliCodina, NuriaPandhi, AbhiCoe, Christopher L.Panek-Shirley, Leah M.Coelho Silva, MateusPaneque, ManuelCoelho, AnaPang, LeiCoelho, Antonio Victor CamposPang, PatrickCoelho, J. Luis BentoPang, ShintaroCoelho-Silva, JuanPang, YuanjieCoffman, Cynthia JanPangestu, MulyotoCoggon, DavidPani, DaniloCohen, AdamPani, Shantanu KumarCohen, GuyPaniagua, ÁngelCohen, Harold C.Paniagua, Francisco JavierCohen, Michael B.Panico, AlessandraCohen, OdeyaPanico, AntonioCohen, RamiPannek, JürgenCohen, Rubin I.Pano-Rodriguez, AlvaroCoheña-Jimenez, ManuelPanoutsakopoulos, VassiliosCoiera, EnricoPant, Puspa RajCoimbra, SusanaPantanella, FabrizioCoker, DavidPantelidou, MariaCokley, JohnPantelis, ZebekakisColasante, AnnaritaPantha, SandeshColavita, GiampaoloPantusa, DanielaColder Carras, MichellePantyley, ViktoriyaColdwell, DavidPanzarini, ElisaCole, CaseyPao, MarylandCole, ChristianPaoletta, MarcoColella, GiuseppePaoletti, Anna MariaColeman, Nichola J.Paoli, SoniaColenbrander, AugustPaolini, ValerioColi, PierluigiPaolotti, DanielaColim, AnaPapachristos, Ioannis V.Colinet, GillesPapadakaki, MariaCollado-Mateo, DanielPapadaki, DimitraCollart-Dutilleul, Pierre-YvesPapadaki, EugeniaColleluori, GeorgiaPapadakis, ManosCollese, TatianaPapadakis, StamatiosCollini, FedericaPapadakis, ZachariasCollins, ChristopherPapademas, PhotisCollins, MatthewPapadimitriou, Dimitrios T.Collins, SamuelPapadimitriou, KaterinaCollins-Emerson, JuliePapadimitriou, KonstantinosCollison, LydiaPapadopoulos, TheofilosCollister, LaurenPapadopoulou, SousanaCollivignarelli, Maria CristinaPapagiannis, DimitriosColman, RickiPapaioannou, EmmanouilColombo, MarcoPapaix, ClaireColomer Feliu, JordiPapalia, RoccoColonna, PasqualePapamitsou, TheodoraColosimo, AlfredoPapanastasiou, AnastasiosColpaert, AlfredPapandreou, MariaColquhoun, RyanPapanikolaou, YanniColquitt, GavinPaparas, DimitriosColston, DavidPaparusso, AngelaComacchio, CarlaPapathanakos, GeorgiosComan, EmilPapathanasopoulou, VasileiaComas, MariaPapathanassis, AlexisComas-Garcia, AndreuPapavasileiou, GeorgiosCombroux, IsabellePapazafiropoulou, AthanasiaCometti, CarolePapazoglou, KonstantinosComi, CristoforoPapet, IsabelleComino, PennyPapi, PieroComizzoli, PierrePapoutsi, CharaCommodore, SarahPapoutsi, GeorgiaCompton, StevenPapović, TamaraComunello, FrancescaPappalardo, VivianaConca, EleonoraPappas, Richard StevenConceição, NatérciaPapp-Wallace, Krisztina M.Conde Cid, ManuelPaquette, Marie-ClaudeConde, BebianaParagliola, Rosa MariaCondello, GiancarloParajuli, Daya RamCondemi, VincenzoParajuli, Rishi RamConde-Sala, Josep LluísPáramo, María FernandaCondessa, Isabel CabritaParanhos, Luiz RenatoCondo, RobertaParasidis, EfthimiosCone, JamesParaskevas, George P.Conejero, AngelaParaskevopoulou, AngelikiConesa, Juan A.Parchomiuk, MonikaConesa-Zamora, PabloPardío-Sedas, Violeta T.Confalonieri, MarcoPardos Mainer, ElenaConforti, MassimoParedes-Madrid, LeonelCong, LiuParent, André-AnneConklin, DanielParent-Lamarche, AnnickConley, ClaireParfrey, HelenConnaughton, Daniel P.Pari, LuigConnelly, LukeParis, HunterConner, DavidParis, JoelConners, Gregory P.Paris, MarioConnolly, AlisonParis-Alemany, AlbaConnor, Helene DianaParisey, NicolasConroy, DominicParisi, AlessandroConserva, EnricoParisi, AlfioConson, MassimilianoPark, AelyConstance, DouglasPark, ByoungjinConstantine, ParinosPark, Chae YeonConstantini, KerenPark, Chan RyulConstantino, Rose E.Park, Chang-EunConstantinou, Costas S.Park, Chan-SikContag, StephenPark, Cheong K.Contalbrigo, LauraPark, ChloeContaldo, MariaPark, DanielConte, FerruccioPark, Du-JinContestabile, PasqualePark, Edwards A.Conti, CristianaPark, Eun-YoungConti, DanielaPark, Hee-SooConti, SaraPark, Hun-YoungContiero, PaoloPark, Hyun JunContini, DanielePark, Jae BumContreras-Cornejo, Hexon AngelPark, Jeong-YeolConversi, DanielePark, JihongConversi, DavidPark, JusikConvertino, MatteoPark, JuyounConvissar, Robert A.Park, KeunhyunCook, Alan D.Park, KihongCook, MargaretPark, Kwang O.Cook, MatthewPark, KyungBaeCook, SarahPark, MoojongCools, WouterPark, Sang KilCooper, BudPark, Shin JunCooper, DavidPark, Sin-AeCooper, RobinPark, SohyunCooper-Knock, JohnathanPark, SojungCopat, ChiaraPark, SongyoungCope, MichaelPark, Sook-HyunCoppedè, FabioPark, SookukCoppeta, LucaPark, So-YounCoppo, LuciaPark, Sungkyu ShaunCoppola, AngelaPark, Yoo KyoungCoppola, GabriellePark, Youn ShikCoq-Huelva, DanielPark, YuriCoratella, GiuseppeParker, AlisonCorazza, IlariaParker, Elizabeth A.Corazza, Maria VittoriaParker, HelenCorbacho, IsaacParker, JamesCorbett, StephenParker, KateCordani, MarcoParker, Lucy AnneCordaro, MassimoParker, MeganCordeiro, Erick M. G.Parks, ChristineCordeiro, JoãoParks, JasonCordente Rodríguez, MariaParks, RuthCordi, MarenParl, FritzCordier, WernerParlapani, Foteini F.Córdoba Fernández, AntonioParlesak, AlexandrCórdoba, OctaviParmenter, TrevorCordoza, MakaylaParnell, JillCori, LilianaParodi, EmiliaCoria, Luis N.Parolini, MarcoCorica, FrancescoParpala, AnnaCorlateanu, AlexandruParr, Jeffrey J.Corlier, JulianaParra Boronat, LorenaCorlin, LauraParra Camacho, DavidCormio, LuigiParra, DenisseCornejo, JoseParra-Anguita, LauraCorominas Roso, MargaritaParra-Rizo, María AntoniaCoronel, AnibalParra-Saldívar, RobertoCoros, Monica MariaParrello, SantaCorrado, GiovanniParreño-Castellano, Juan ManuelCorrado, RindoneParrettini, SaraCorral, NadiaParrinello, GiampieroCorrales-Serrano, MarioParrino, FrancescoCorrea Marques, RejaneParrish II, Richard H.Corrêa, Camila RenataParrish, RichardCorrêa, Elizabeth NappiParry, DaveCorrea, JohnParry-Strong, AmberCorrea, Maria ElviraParsa, Ali DavodCorrea-Burrows, PaulinaParsaeimehr, AliCorreale, LucaParsanathan, RajeshCorrea-Rodríguez, MariaParsons, JasonCorregidor-Sanchez, Ana IsabelParsons, MatthewCorreia, Pedro Miguel Alves RibeiroParsons-Smith, Renée L.Corridore, DenisePartalidou, MariaCorrigan, Oonagh PatriciaPartchev, IvailoCorsalini, MassimoPartington, HazelCorsi, AlessandroParv, LuminitaCorso, GiovanniParvate, AmarCorten, LieselotteParvez, MohammadCortés, Rocío JiménezPasanen, PerttiCortés, UlisesPasanisi, PatriziaCortese, AntonioPascale, ScherommCortese, ClaudioPascarella, JosephCortes-Reyes, MarinaPaschali, MyrellaCortés-Rodríguez, Alda ElenaPaschalis, VassilisCortignani, RaffaelePáscoa-Pinheiro, JoãoCortis, CristinaPasek, MałgorzataCorvalan, CarlosPasek, MarcinCosola, SaverioPasini, MargheritaCosta De Camargo, AdrianoPásková, MartinaCosta Freitas, Maria De BelémPasławski, JerzyCosta, Ana Luísa MoreiraPasman, Hans J.Costa, Emilia MartinsPasquale, Jorgelina DiCosta, JoanaPasqualetti, PatrizioCosta, JoaoPasqualoto, KerlyCosta, LaraPassafaro, PaolaCosta, Mário JorgePassanisi, AlessiaCosta, SebastianoPassarelli, Pier CarmineCosta, StefaniaPasserini, GiorgioCostabile, AngelaPassos, LígiaCostabile, FrancescaPassos, MarisaCosta-Black, Katia MPasten, DenisseCosta-Climent, RicardoPasternak, GerardCosta-Da-Silva, André LuisPastor Fernández, AndrésCostan, Victor-VladPastor Seller, EnriqueCostantini, AriannaPastor Vicedo, Juan CarlosCostantini, ElisabettaPastor, DiegoCostantini, EricaPastor, RosarioCostantino, ClaudioPastor-Bravo, María Del MarCostanza, AlessandraPastore, IdaCostanza, FrancescaPastori, DanieleCostanzo, SimonaPastorino, PaoloCosta-Pinto, Ana RitaPastuszka, AgnieszkaCosta-Sánchez, CarmenPaszkiel, SzczepanCosta-Tutusaus, LluísPaszkowski, TomaszCosteira, CristinaPatarata, LuísCostella, Marcelo FabianoPatarrão, RitaCostley, AlexPatchay, SandhiranCostola, MichelePatel, AlokCota, IulianaPatel, Barkha P.Cota, LuísPatel, DevenCôté, RochellePatel, Dilip R.Cotfas, Liviu-AdrianPatel, JenilCotter, JoshuaPatel, KushalCottineau, ClementinePatel, Mahomed S.Couch, James R.Patel, MoosaCoughlan, ChristinaPatel, MrudulaCoulson, SamanthaPatel, PalakCoulter, JohnPatel, RiyaCounil, Francois-PierrePatel, UrvishCounsell, AlyssaPatel, Vibhuti N.Court, Karem A.Patelarou, AthinaCourtney, JimikayePatelarou, EvridikiCousins, RosannaPatella, VincenzoCoussens, ScottPatelli, EdoardoCouth, SamuelPateman, Rachel MaryCoutinho, ArturPaternoster, GianlucaCoutinho, Henrique Douglas MeloPat-Espadas, Aurora M.Coutinho, Maria FranciscaPathak, AshishCouto, Arnaldo CézarPathak, DhrubaCouto, CristinaPathirana, Ruvini U.Coutts, Rosanne A.Pathirana, SumithCouvin, DavidPatil, AshokCovelli, StefanoPatil, UjwalkumarCoventry, PeterPatil, VeerupaxagoudaCowles, Elizabeth A.Patini, RomeoCox, Christopher R.Patino, LuisCox, Jennie D.Patiño, María José MartínezCox, RosiePatiris, DionisisCrabbe, HelenPatlakas, PlatonCrabbe, JamesPatman, ShaneCraemer, ThomasPatnaik, JenniferCraig, Bruce W.Patnaik, SouravCraig, MorriePatowary, AshokCrane, MelaniePatriarca, RiccardoCranganu, ConstantinPatricia, Concheiro-MoscosoCraveiro, DanielaPatrick, MatthewCraveiro, IsabelPatrick, Robert (Canada)Crawford, LindseyPatrick, Robert (USA)Crawford, Stephanie Y.Patrizi, BarbaraCrean, Angela J.Patrizi, PatriziaCreasey, Jean L.Patrizia, OlivaCrecente, FernandoPatro-Małysza, JolantaCreedon, Sile A.Patrone Cotrim, TeresaCreel, EileenPatsiaouras, GeorgiosCreely, EdwinPatten, Scott B.Creese, JenniferPatterson, CarsonCremasco, Margherita MichelettiPatti, SebastianoCrenna, FrancescoPattison, TonyCrescimbene, LaraPattusamy, MuruganCrescimbene, MassimoPatyra, EwelinaCrespo, NunoPaudel, BasantaCrespo-Escobar, PaulaPaudel, Keshav RajCrespo-Lopez, Maria ElenaPaudel, MohanCrevecoeur, SophiePauk, JolantaCriado-Alvarez, Juan JoséPaul, AlokCrisan, BogdanPaul, AnuCrisol-Moya, EmilioPaul, ChristopherCrisp, GeoffreyPaul, JohnCristache, Corina MarilenaPaula, Anabela BaptistaCristiani, PierangelaPaulik, EditCristina Rada, ElenaPauliquevis, TheotonioCristino, SandraPaulo Mirasol, EstherCristofaro, Maria GiuliaPaulo, Rui MiguelCrizer, David M.Paulson, SallyCrock, MaryPaunović, IvanCrocker, George H.Pauri, FlaviaCrofts, CatherinePautz, AndreaCroghan, IvanaPavela, RomanCrohn, Helen M.Pavilonis, BrianCrompton, DavidPavione, EnricaCronk, RyanPavli, FoteiniCronkite, Ruth C.Pavlickova, KatarinaCrook, BrianPavlidis, GeorgeCrooks, GeorgePavlik, EdwardCropp, CherylPavlov, DanailCrosbie, EricPavlovskis, MiroslavasCross, Suzanne L.Pavone, PieroCrouch, AlanPawar, AmbarishCrowe, KellyPawar, Shrikant D.Crowley-McHattan, ZacharyPaweł, TomaszewskiCrozier, Alyson J.Pawlaczyk-Kamieńska, TamaraCruceru, Anca FranciscaPawlaczyk-Łuszczyńska, MałgorzataCruickshank, Margaret EleanorPawlby, SusanCruise, EdiePawlewicz, KatarzynaCruvinel, Vanessa Resende NogueiraPawlowska, BeataCruz Campas, Martín EusebioPawlowska, ElzbietaCruz Diaz, DavidPawlowski, LucjanCruz, Carlos OliveiraPayá Castiblanque, RaúlCruz, Maria LeticiaPayan-Carreira, RitaCruz, PedroPayne, PhillipCruzado Rodríguez, Juan AntonioPayne-Sturges, Devon C.Cruz-Morato, Marco AntonioPaz Montelongo, SorayaCruz-Quintana, FranciscoPaz, IgorCsordás, AnitaPazdro, KseniaCsortan, GeorgiaPearce, Dora ClaireCuadra, GiancarloPearce, John A.Cuadrado-Cenzual, Maria AngelesPearl, DrewCuberos, Ramón ChacónPearman, Timothy P.Cucinotta, FilippoPearson, Claire F.Cueli, MarisolPearson, NatalieCuenca-Martínez, FerranPeasgood, TessaCuesta, DanielPeaucelle, MarcCueto Ancela, José LuisPech, MartinCuevas-Covarrubias, SergioPeck, Richard M.Cugurullo, FedericoPeckham-Gregory, Erin C.Cui, LinlinPeckhaus, AndreasCui, WeiweiPecor, KeithCui, WenyuanPecoraro, AngelaCui, XiaohuiPecyna, PaulinaCui, YanxiangPęczkowski, GrzegorzCui, ZhouqiPedemonte-Sarrias, EduardCuller, CorinnaPeden, AmyCullerton, KatherinePeden, Margaret M.Culpan, IanPedersen, CathrineCumbo, FabioPedersen, Daphne E.Cummins, IanPedraza Chaverri, JoséCunanan, AaronPedregal Mateos, BelénCunha Rudge, Marilza VieiraPedreño Rojas, Manuel AlejandroCunha, DiogoPedreño, Jose NavarroCunitz, KatrinPedrero Pérez, E. J.Cunliffe, DanielPedrero-Esteban, Luis MiguelCunningham, Christopher W.Pedruzzi, RebeccaCunningham, George B.Pedullà, LudovicoCuong, Do QuocPeer, SamuelCupani, MarcosPeerenboom, Peter BobCurado, AntónioPeers, CameronCurenton, StephaniePegado, ElsaCuric, GoranPegalajar, Mª CarmenĆurković, MarioPehlken, AlexandraCurl, AngelaPei, QingCurra, EdoardoPei, YonggangCurrie, CharlottePeidró, Elias CasalsCurrier, G. FränsPeira, GiovanniCurry, DionPeiris, CaseyCurtin, KimberleyPeiró, Ana M.Curtis, Jacqueline W.Peiró, SalvadorCurtis, MarionPeitsidis, PanagiotisCurto, AdrianPeixe, Tiago SeveroCurto, AdriánPejin, BorisCushing, Sharon L.Peláez-Ballestas, IngrisCuthbertson, JosephPeláez-Fernández, María AngelesCuthill, FionaPeláez-Moreno, CarmenCuypers, BartPelegrín, AntoniaCvetković, VladimirPeletz, RachelCvorovic, JelenaPelfrêne, AurélieĆwieląg-Piasecka, IrminaPeli, MarcoĆwirlej-Sozańska, AgnieszkaPelicano, Christian MarkCwynar, AndrzejPelicioni, Paulo Henrique SilvaCybulski, MateuszPelidou, Sygkliti-HenriettaCynarski, Wojciech J.Pelissari, Allan LibanioCyplik, PawełPellegrina, HeitorCyril, FouilletPellegrini, ElisaCzapla, MichalPellegrini, GaiaCzaplicki, LaurenPellegrini, PaolaCzarniecka-Skubina, EwaPellegrini-Giampietro, Domenico EdoardoCzarnigowska, AgataPellegrino, GerardoCzarnocki, Krzysztof J.Pellegrino, RobertaCzech, BożenaPellesi, LanfrancoCzech, KatarzynaPelletier, ChelseaCzechowski, Piotr OskarPelletier, GuillaumeCzenczek-Lewandowska, EwelinaPellicer, TeresaCzerwińska, AgnieszkaPellitteri, JohnCzlapka-Matyasik, MagdalenaPeluso, IlariaCzuczwar, MirosławPeluso, Marco E. M.Czwikla, GesaPeña, CarlosCzyż, KatarzynaPeñacoba Puente, CeciliaCzyżewska, MartaPeña-García, AntonioCzyżowska, DorotaPeña-González, IvánD’Accardi, EsterPeñarroja-Cabañero, VicenteD’Acci, LucaPeña-Sánchez, Antonio RafaelD’Accolti, MariaPence, Alicia R.D’Addazio, GianmariaPence, BrandtD’Agostino, DonatoPenelo, EvaD’Alessio, Patrizia A.Peng, DuoD’Alonzo, MarcoPeng, HaoD’Alterio, Maurizio NicolaPeng, JianfengD’Amario, MaurizioPeng, Michael Yao-PingD’Ambrosio, Francesca RomanaPeng, WeiD’Amico, CesarePeng, YangD’anca, MariannaPeng, Ying-JieD’Angelo, StefaniaPenlington, ChrisD’Anna, CarmenPenmetsa, Gopi K.D’Apuzzo, FabriziaPennington, CatherineD’Ascenzi, CarloPenno, MeganD’Ascenzo, FabrizioPentakota, Sri RamD’Auria, AnnaPentaris, PanagiotisD’Costa, VanessaPenteado, CarmenluciaD’Cunha, Nathan M.Penttinen, Olli-PekkaD’Ettorre, GabrielePenz, ErikaD’Onofrio, LucaPepe Razzolini, Maria TerezaD’onofrio, RosalbaPépin, MichelD’Souza, Randall FelixPepper, JessicaD’Souza, RoshanPeprah, Emmanuel K.D’Souza, Russell F.Peral Gómez, PaulaD’Ulizia, AriannaPerales Jarillo, MikelD’Ursi, Anna MariaPerales, AlfredoD’Urso, GiulioPeral-Suárez, ÁfricaDa Broi, UgoPerano, MirkoDa Cruz Perez, Danyel EliasPerbellini, LuigiDa Cunha De Sá-Caputo, DanúbiaPercino, JudithDa Cunha, Mário Antônio AlvesPercival, TriciaDa Fonseca, Maria De Jesus MendesPerdana, Aji PutraDa Ponte, GuidaPereira Costa, VitorDa Pozzo, StefanoPereira De Figueiredo, InêsDa Rosa, Wellington Luiz De OliveiraPereira, AlexandraDa Silva Batista, Marco AlexandrePereira, AnaDa Silva Castanha, Priscila M.Pereira, Ana De Fátima Da CostaDa Silva Grigoletto, Marzo EdirPereira, Ana LuisaDa Silva Guerra, Danielle ReginaPereira, CatarinaDa Silva Rotta, Luiz HenriquePereira, DoraDa Silva Souza, Ivan DomicioPereira, Elsa Cristina SacramentoDa Silva, A. MoreiraPereira, Jorge AlbertoDa Silva, Denize FranciscaPereira, ManuelDa Silva, IvanPereira, Maria De LourdesDa Silva, Juarez BentoPereira, PauloDa Silva, JulimarPereira, SaraDa Silva, Luis Cláudio NascimentoPereira-da-Silva, LuísDa Silva, Maria De Fátima PereiraPereira-Silva, Erico FernandoDa Silva, Paula F.Perelli, ChiaraDa Silveira, Ismael HenriquePerenc, LidiaDa Veiga, Claudimar PereiraPerepa, PrithviDabbous, SaadPeres, S. CamilleDąbrowska, JolantaPeressotti, FrancescaDabrowska, KatarzynaPereyra-Zamora, PamelaDa̧browska-Galas, MagdalenaPérez Ballesta, PascualDąbrowski, KarolPérez Corrales, JorgeDąbrowski, MikołajPérez- De La Cruz, SagrarioDaca, AgnieszkaPérez Marfil, Maria NievesDacheux, Jean-LouisPérez Navío, EufrasioDaCosta, BoaventuraPérez, Eduardo CasilariDada, ShakilaPérez, Felipe OjedaDadelo, StanislavasPerez, FranciscoDadi, TallentPerez, GabrielDaďová, KláraPérez, JorgeDadzie, JohnPerez, LeviDahlqvist, HelénePerez, Maria Coral FalcoDahm, MathewPerez, PatricioDahmen, BrigittePerez, Victor W.Dai, BoyiPerez-Acebo, HeribertoDai, QiliPérez-Alenda, SofíaDai, ZhijunPérez-Alonso, JoséDaim, Tugrul U.Perez-Burriel, MarcDaiwen, KangPérez-Cutillas, PedroDalamitros, AthanasiosPérez-Donoso, Jose ManuelDalby, SimonPérez-Escoda, AnaDalefield, RosalindPérez-Ferreirós, AlexandraDalessandri, DomenicoPérez-Fuentes, María Del CarmenDaley, DorothyPérez-Gómez, JorgeDalkiz, MehmetPérez-González, CarlosDalla Villa, PaoloPérez-Guerrero, Edsaul EmilioDallari, FiorellaPérez-Heredia, MercedesDal-Pizzol, Tatiane Da SilvaPerez-Hernandez, ConcepciónDalton, HazelPérez-Herrero, María A.Dalvie, Mohamed AqielPerez-Heydrich, CarolinaDaly, AlisonPérez-Jover, VirtudesDama, MuraliPérez-López, JulioDamaševičius, RobertasPérez-Marín, MariánDamialis, AthanasiosPérez-Martín, EnriqueDamiani, DanielaPérez-Mira, VenturaDamiani, GianfrancoPérez-Núñez, RicardoDamma, DevaiahPérez-Ordás, RaquelDamodaran, SureshPérez-Padilla, JavierDamodaran, TirupapuliyurPérez-Rodrigo, CarmenDamos, Petros T.Perez-Rodriguez, FernandoDamulevičienė, GytėPérez-Ros, PilarDanacova, MichaelaPérez-Rubio, GloriaDancer, StephaniePerez-Sayans, MarioDanchin, MargiePérez-Surio, Alberto FrutosDandekar, AdityaPerez-Tejero, JavierDanese, GiuseppePérez-Villarreal, Héctor HugoDaneshkhah, AlirezaPérez-Zepeda, Mario UlisesDanesi, FrancescaPergadia, MicheleDang, ChongyuPeri, LuriDang, ShaonongPeriasamy, RameshDang, Thanh DucPerić, DušanDang, ZhiPericot-Valverde, IreneDaniel, Da SilvaPerikos, IsidorosDanielewicz, AnnaPerimal-Lewis, LuaDaniels, CharlesPerin, CeciliaDaniels, JenniferPeri-Rotem, NitzanDaniels, TomPeris, LolaDanila, EdvardasPerisetti, AbhilashDanilovich, Margaret K.Peristeri, EleniDanjou, AuréliePerkins, David A.Danks, MarcellaPerkins, GregDantas, CarinaPerko, TanjaDantas, Mario A. R.Perła-Kaján, JoannaDantas, Roberto OliveiraPerlini, CinziaDante, AngeloPerlman, DanielDanvers, Alexander F.Perna, AngelicaDanzi, EnricoPerna, SimoneDaoust, Jean-FrancoisPerouansky, MishaDaovisan, HanvedesPerpetuini, DavidDar, Mohd SaleemPerra, OliverDar, Wasim A.Perraudin, ClémenceDarbro, JonathanPerrey, StéphaneDarchia, NatoPerri, Mark J.Dardé, Marie-LaurePerricone, CarloDario, BrunettiPerron, HervéDariš, BarbaraPerrot, TaraDarling, KatherinePerrotta, Andrew ScottDarlington, EmilyPerrotti, VittoriaDarnell, RegnaPerry, CynthiaDaronkola, Hassan KalantariPerry, Kevin D.Darques, RégisPers, Yves-MarieDarr, DietrichPersson, IngmarDarssan, DarsyPersson, MargaretaDarvell, Brian W.Perugini, EleonoraDarville, AudreyPérusse, MichelDarvizeh, AtanazPes, Giovanni MarioDaryanani, SunilPescara-Kovach, LisaDas Mercês, Magno ConceiçãoPesimena, GabrieleDas, Dillip KumarPesola, FrancescaDas, RajdeepPessi, TanjaDas, RinaPessoa, Luis Gustavo AmorimDas, SauravPestana, José V.Dasgupta, DebanjanPester, AndreasDasgupta, SatarupaPeszek, ŁukaszDasgupta, ShouroPetach, HelenDash, SarahPeter, Brad G.Dashper, KatherinePeter, RichardDassenakis, Manos J.Peters, FlorisDastsooz, HassanPeters, John C.Datta, RahulPeters, MicahDatta, SujayPeters, PaulDaugherty, JillPeters, RuthDaumann, FrankPeters, SusanDaumiller, MartinPetersen, ElijahDaus, CatherinePeterson, GregoryDautovitch, NataliePeterson, Joel C.Dautzenberg, BertrandPeterson, Jordan CarrDaveluy, StevenPeterson, LisaDavey, JanetPeterson-Hiller, AmieDavidovich, EstiPetersson, JakobDavidow, AmyPethick, JamesDavidy, AlonPethick, JamieDavies, Anita A.Petisco-Rodríguez, CristinaDavies, BenjaminPetito, AnnamariaDavies, Jacqueline M.Petitti, DianeDavies, KatePetkari, EleniDavies, SimonPetkevičienė, JaninaDavis, A. SallyPetracchini, FrancescoDavis, JustinPetracci, BarbaraDavis, KathleenPetrakovska, OlgaDavis, MarionPetrarca, Mateus HenriqueDavis, MaryPetráš, MarekDavis, Mellar P.Petrashova, DinaDavis, Sally M.Petrauskiene, SandraDavlyatov, GanisherPetreaca, Ruben C.Dawes, JonathanPetrella, AndreaDe Abreu, Luiz CarlosPetrelli, AlessioDe Alcantara Camejo, FlavioPetretto, Donatella RitaDe Alcaraz, Antonio GarciaPetrič, GregorDe Allegri, ManuelaPetrica, JoãoDe Almeida Bicudo, Álvaro JoséPetriglieri, Jasmine RitaDe Almeida Honório, Samuel AlexandrePetrisor, Alexandru-IonutDe Amicis, RamonaPétritis, DimitriDe Andrade, MarizaPetriz, Bernardo A.De Andrés, Ana LópezPetrocchi, SerenaDe Angelis, MarcoPetroni, SergioDe Angelis, PaoloPetrosino, MariaDe Angelis, SaraPetrov, Alexey M.De Araújo Guerra, Paulo HenriquePetrov, Megan E.De Araujo, Ludgleydson Ludgleydson FernandesPetrov, Vladimir G.De Araújo, Tânia MariaPetrović, BojanDe Arróyabe, PabloPetrulionis, MariusDe Barros, Selma GouvêaPetrunoff, NicholasDe Bell, SiânPettersen, Kjell SverreDe Berardis, DomenicoPetzold, GuillermoDe Bernardi Schneider, AdrianoPeyré-Tartaruga, Leonardo AlexandreDe Bernardo, MaddalenaPeyton, David H.De Blas, Clara SimonPezhummoottil Vasudevan, AsharaniDe Briyne, NancyPezzuto, AldoDe Bruyckere, PedroPfadenhauer, LisaDe Carvalho, Diana E.Pfaff, HolgerDe Carvalho, Kênia Mara BaiocchiPfahlberg, AnnetteDe Carvalho, Luis Manuel MadeiraPfeifer, Jeffrey E.De Carvalho, Renata MedeirosPfeifer, KlausDe Carvalho, Rodrigo M.Pfuhl, GeritDe Castro, AlexandrePhan, LinhDe Castro, Bruno AlbuquerquePhan, TungDe Castro, GabrielaPhanthuwongpakdee, NuttavikhomDe Castro, Ricardo DiasPhibbs, Suzanne R.De Castro, Shamyr SulyvanPhilalithis, AnastasDe Cauwer, HaraldPhilibert, DanielleDe Cos Guerra, OlgaPhillipou, AndreaDe Craemer, MariekePhillips, J. CraigDe Cuyper, ChristaPhillips, KevinDe Decker, PascalPhillips, LorraineDe Deurwaerdere, PhilippePhilp, FraserDe Diego Cordero, RocíoPhipps, RobynDe Donno, Maria AntonellaPhiri, JosphatDe Figueiredo Neves, Gustavo ZenPhonchi, NametsoDe Figueiredo, Ciro José JardimPhulera, SwastikDe Figueiredo, José Antonio PoliPiacenti, IlariaDe Filippo, DanielaPiacentini, Maria FrancescaDe Francesco, ErnestinaPiana, AndreaDe Francesco, Maria CarlaPiancino, MariaDe Francisco, SeylaPiantini, SimoneDe Freitas, Hanniel Ferreira SarmentoPiasecka, AgnieszkaDe Geest, BartPiasecki, JessicaDe Gennaro, LuigiPiasecki, MichałDe Giuseppe, ToniaPiątkowska, MonikaDe Graaf, Barend H. J.Pic Aguilar, MiguelDe Groene, GerdaPiccoli, ValentinaDe Groot, EricPícha, KamilDe Haro, María Reina GranadosPichon, LionelDe Jesus, KarlaPichon, MaximeDe Jesus, Saúl NevesPichugin, YuriyDe Juanas, AngelPickart, LorenDe Kock, CarmenPicoloto, Rochele SogariDe Korne, DirkPicon, MoisesDe Kroon, Marlou L. A.Pié, LaiaDe La Cruz Campos, Juan CarlosPiebenga, WillemDe La Cruz Sánchez, ErnestoPiechowiak, TomaszDe La Cruz, Milagros HidalgoPięciak, TomaszDe La Franier, BrianPiemontese, LucaDe La Fuente, JesúsPienkowski, KrzysztofDe La Fuente-Robles, Yolanda MaríaPiepenbrink, KurtDe La Fuente-Solana, Emilia I.Pieper, BarbaraDe La Iglesia, BeatrizPieper, ClaudiaDe La Paz, Manuel PosadaPierannunzio, DanielaDe La Piedad Garcia, XochitlPierantoni, Giovana MariaDe La Poza Plaza, ElenaPierce, Alex M.De La Rosa, Antonio LuquePiercy, HilaryDe La Rosa, José MaríaPierucci, PaolaDe La Torre, Arturo HardissonPiesowicz, ElżbietaDe La Torre-Montero, Julio C.Pietilä, PekkaDe La Torre-Torres, OscarPietkiewicz, MichałDe La Vega, RocioPietkiewicz, PawełDe Laat, Jan A. P. M.Pietraszewski, PrzemysławDe Las Heras Rosas, CarlosPietrobelli, AngeloDe Las Heras-Pedrosa, CarlosPietrogrande, Maria ChiaraDe Las Mercedes Novo-Muñoz, MariaPietrzak, OliwiaDe Leeuw, PeterPietrzak-Zawadka, JoannaDe Leo, GianlucaPietschnig, JakobDe Leo, VincenzoPigatto, Paolo D.De Ligt, KellyPigłowski, MarcinDe Lille, Lydia CoudroyPignataro, GiuseppeDe Luca, PaolaPignatti, PatriziaDe Marchi, TommasoPihkala, PanuDe Marcos Ruiz, SusanaPihlainen, KaisaDe Martelaer, KristinePihl-Thingvad, JesperDe Masi, LuigiPihut, MałgorzataDe Matteis, AlessandraPikala, MałgorzataDe Mauroy, Jean ClaudePike, IanDe Mello-Sampayo, FelipaPikhart, HynekDe Melo, Rosa Cândida Carvalho PereiraPikhart, MarcelDe Melo, Silas NogueiraPiknova, BarboraDe Melo-Tavares, Claudia MaraPikora, TerriDe Mendonça, Dina I. M. D.Pilař, LadislavDe Mesnard, LouisPileggi, ClaudiaDe Miguel, SilviaPilewska-Kozak, Anna BogusławaDe Miranda, João Antônio LealPilgrim, Erik M.De Molon, Rafael ScafPill, ShaneDe Morais, Marcos Vinicius BuenoPilla, RaffaeleDe Moura, Ana PintoPillai, PriyaDe Müllenheim, Pierre-YvesPillay, ManikamDe Oliva, Samara UrbanPillay, Nalini SooknananDe Oliveira, Aline MacedoPilli, NageswaraDe Oliveira, Daniel VicentiniPilogallo, AngelaDe Oliveira, Gustavo VieiraPilon, Bonnie A.De Oliveira, Leise KelliPilotto, José HenriqueDe Oliveira, Tássio BritoPiloyan, ArtakDe Oña, JuanPiltan, FarzinDe Pablo Valenciano, JaimePimenoff, Ville N.De Palma, GiuseppePimentel, GustavoDe Pascale, FrancescoPimentel, PedroDe Paula, Janice SimpsonPina Gadea, CarmeloDe Paz Fernández, Jose AntonioPineda-Espejel, Heriberto AntonioDe Perio, MariePinedo-Rivilla, CristinaDe Roock, SophiePiñeiro Aguín, IsabelDe Rosa, DanielePiñero, ElenaDe Rosa, EnricaPing Yu, ChangDe Rosa, MarcelloPingani, LucaDe Rosis, SabinaPingitore, NicholasDe Ruiter, Jan JaapPinheiro, PlácidoDe Ruiter, Maurits H. T.Pinho, Maria Gabriela M.De Runz, CyrilPinho, Ricardo A.De Ruyter, BorisPinilla-Dominguez, JaimeDe Saá Guerra, YvesPink, AimeeDe San Antonio-Gómez, CarlosPinkas, AdiDe Sanctis, Juan BautistaPinkas, JarosławDe Santis, FulvioPinna, FrancescoDe Santos Berbel, CésarPino, MaribelDe Sario, ManuelaPino-Ortega, JoséDe Schryver, AntoonPinto, AgneseDe Silva, AmarasiriPinto, EleonoraDe Silva, NicolePinto, ElisabeteDe Silva, ShamaliPinto, ManuelDe Simone, ClaudioPinto, Marina Marques Da Silva CabralDe Simone, FrancescoPinto, Rogério M.De Simone, Salvatore GiovanniPinto, TeresaDe Soet, Johnannes JacobPinto, UelintonDe Sousa Leite, TiagoPintor, ArianaDe Sousa, Álvaro Francisco LopesPiotrowicz, KatarzynaDe Sousa, Bruno MacedoPiotrowska, KatarzynaDe Souza Batista, Victor EduardoPiotrowski, AndrzejDe Souza Lalic, SusanaPiovesan, LucaDe Souza Loureiro, ElisângelaPiper, BrianDe Souza Vanz, Samile AndreaPipolo, CarlottaDe Souza, Danival JoséPippi, RobertoDe Stasio, SimonaPiqueras, José A.De Stefani, AlbertoPirani, ElenaDe Stefano, Adriana A.Pirani, VittorioDe Stefano, RosaPiras, AlessandroDe Steur, HansPires Júnior, Osmindo RodriguesDe Vita, AlessandroPires Manso, Jose’ RamosDe Vito, ElisabettaPires, Adília O.De Vries, JurienaPires, CarlaDe Vuyst, StijnPires, JoãoDe Wilde, KatrienPirim, HarunDe Witte, LucPirttiniemi, PerttiDe Zeeuw, JaninePisani, LucianaDe Zinis, Luca Oscar RedaelliPisano, RaffaeleDe Zotti, MartaPisanu, ClaudiaDe, TanimaPisapia, RaffaellaDe’ Donato, FrancescaPisarenko, VeronikaDean, AndrewPiscia, RobertaDean, ElizabethPiscitelli, PriscoDean, FrankPiscopo, MarinaDean, MichaelPisello, AnnaDean, NathanPiskin, SenolDeAngelis, DonaldPisla, DoinaDeary, MichaelPisoni, EnricoDeath, JodiPistelli, FrancescoDeb, SubrataPitarch, AidaDebertolis, PaoloPitchford, AndrewDecaux, GuyPitt, MirandaDecroo, TomPitter, Janos G.Deehan, JamesPitts, Robert E.Deepak, JanakiPitura, KarolinaDeery, ChrisPiu, SahaDeFlorio-Barker, StephaniePiwowar, ArkadiuszDegrazia, FelipePiwowar-Sulej, KatarzynaDehghani, MiladPizarro, JoseDe-Hita-Cantalejo, ConcepciónPizzolanti, GiuseppeDeibert, PeterPizzoli, Silvia Francesca MariaDeidda, ManuelaPizzuti, ClaraDeigner, Hans-PeterPlacek, CaitlynDeim, ZoltánPlacek, MichalDeindl, ElisabethPlakas, KonstantinosDeJarnett, NatashaPlant, LeighDel Barrio, CristinaPlant, RoelDel Ben, MariaPlante, Craig J.Del Casale, AntonioPlante, Kenneth StevenDel Espino-Díaz, LuisPlaß, DietrichDel Gallo, MddalenaPlass-Christl, AngelaDel Hoyo Martínez, Carmen MaríaPlatania-phung, ChrisDel Mistro, AnnarosaPlaton, Tiniosdel Moral Arroyo, GonzaloPlayford, DeneseDel Rio, A. BelenPlaza, CarlosDel Río, F. JavierPlaza-Díaz, JulioDel Río, MarisaPleace, NicholasDel Toro, Eva M. GarcíaPleass, HenryDel Valle Villalonga, LauraPlebanczyk, KatarzynaDe-la-Cruz-Torres, BlancaPlescia, FulvioDelaire, CarolinePletsch-Borba, LauraDelalle, IvanaPliakos, KonstantinosDelCastillo-Andrés, ÓscarPlichta, MartaDelclòs-Alió, XavierPlickert, GabrieleDelellis, NailyaPliska, Benjamin T.Delfabbro, PaulPloch, JindřichDelgado Ruiz, RafaelPlouviez, MaxenceDelgado, CherylPluijm, SaskiaDelgado, JordiPlummer, DavidDelgado, PauloPluta, BeataDelgado-Gómez, DavidPluut, HelenDelgado-Lobete, LauraPlzak, JanDelgado-Losada, María LuisaPng, May EeDeligeoroglou, EfthimiosPoblete Umanzor, RodrigoDelimont, NicolePocock, NicolaDelipetrev, BlagojPoczta, JoannaDeLisi, MattPoczta-Wajda, AgnieszkaDelisle, HélènePodbielska, MagdalenaDelitala, AlessandroPoddighe, DimitriDell, BernardPodell, KennethDell, ColleenPoder, Thomas G.Dell’Isola, MarcoPodhrázská, JanaDell’olmo, ElianaPodkoscielna, BeataDell’oste, ValerioPodlasek, AnnaDella Pergola, RobertoPogacar, TjasaDellaValle, Diane M.Poggesi, AnnaDellavia, ClaudiaPoglayen, GiovanniDelmonico, LucasPogliaghi, SilviaDeLong, GaylePoinhos, RuiDelorme, VincentPoirier, Miriam C.Delussi, MariannaPojednic, RacheleDelva, FleurPojskic, HarisDelvaux, NicolasPóka, RóbertDelvecchio, ElisaPokhrel, Rudra P.Delvecchio, MaurizioPokora, IlonaDelvin, EdgardPokorný, JiříDe-Magistris, TizianaPokorska-Niewiada, KamilaDemaison, LucPoku, Rosemary A.Demant, DanielPõlajeva, TatjanaDémares, Fabien J.Polak, MaciejDeMarini, David M.Polakovicova, IvaDeMarinis, ValeriePoland, BlakeDe-Mateo-Silleras, BeatrizPolanska, KingaDeme, PragneyaPolap, DawidDemetris, AvraamPolari, JuanDemircioglu, Mehmet AkifPolatidis, NikolaosDemirhan, HaydarPoldermans, DonDemissie, Merkebe GetachewPolechoński, JacekDemlie, Molla B.Poleto, CristianoDemmrich, SarahPolimeni, John M.Demnitz, NaiaraPolinder-Bos, HarmkeDemou, EvangaliaPoliti, PierluigiDempsey, Heidi L.Politis, ConstantinusDénes, AttilaPolitis, MariaDeng, HuaPolitynska, BarbaraDeng, JayPolizzi, AlessandroDeng, JiancaiPolkowska, ZanetaDeng, JifengPollack, AnnaDeng, PengPollans, Lily BaumDeng, QihongPollard, Tessa M.Deng, QizhongPollari, FrancescoDeng, ShuguangPollesello, PieroDeng, WenPollice, AlessandraDeng, WuPollick, Frank E.Deng, XinPollini, AlessandroDeng, YihuanPołom, MarcinDeng, YongPolonsky, MichaelDeng, YunbinPolosa, RiccardoDengler-Crish, ChristinePolovina, Snezana P.Deniaud, AurélienPolzer, TobiasDening, TomPoma, AnnaDenis, FredericPomara, CristoforoDenis, KathleenPompeii, LisaDennett, AmyPompeu, JoãoDenzin, NicolaiPompili, MaurizioDepken, DianePonce-Blandón, José AntonioDePrimo, AdamPonocny, IvoDepuydt, ChristophePons, Nívea Adriana DiasDerakhshani, RezaPonsonby, Anne-LouiseDereń, KatarzynaPont, UlrichDerevensky, JeffreyPonta, LindaDering Anderson, AllyPonzini, GiuseppeDerrien, MonikaPoole, ClaudetteDerrien, MorganePoole, Jill A.Desai, Ajay S.Pooley, AlisonDesai, AmritaPoon, EricDesai, AngelPoortvliet, MarijnDesapriya, EdiriweeraPopescu, Gheorghe H.Desborough, JanePopiołek-Kalisz, JoannaDescatha, AlexisPopkin, Stephen M.Deschenes, MichaelPopoli, PaoloDeSesso, John M.Popovic, MajaDeshayes, StevenPopowczak, MarekDeshmukh, RupeshPoppe, LouiseDeshpande, PriyaPoppe, MichaelDesmond, Mary E.Porębska, AnnaDesouzart, GustavoPornaro, CristinaDetanico, DanielePorpino, GustavoDetlefsen, LenaPorreca, AlessioDetsis, VassilisPorrovecchio, AlessandroDettori, MarcoPorru, StefanoDettweiler, UlrichPortales, René LoredoDetzel, PatrickPortela Pereira, NatalieDeutsch, JonathanPortelli, MarcoDevarshi, Prasad P.Porter, AnnaDevesa, JesúsPorter, WilliamDevillers, JamesPortero De La Cruz, SilviaDevine Eller, AudreyPorter-Stransky, KirstenDevine, AlexandraPortilla-Tamarit, IreneDevine, SuePortillo López, AmeliaDevkota, SatisPosada-Baquero, RosaDevlieger, PatrickPosada-Quintero, Hugo F.Devlin, SuePosch, MarkusDeVoe, DalePoškus, Mykolas SimasDevoto, StefanoPosłuszna, ElzbietaDew, RachelPospieszna, BarbaraDeWald, TracyPossik, EliteDeWeese, RobinPostnova, SvetlanaDewey, Rebecca SusanPoston, BrachDewhurst, SusanPostuvan, VitaDeWitt, Jamie C.Potaczek, Daniel P.Dey, DebayanPotdevin, FrancoisDey, KamolPoteser, MichaelDhakal, ChandraPoto, Margherita PaolaDhariwal, RohitPotter, BethDhariwala, MiqdadPotter, Jessica L.Dharker, NachiketPotter, RachaelDhir, AmandeepPotter, WilliamDhital, RanjitaPottie, KevinDhulipala, SomayajuluPotus, FrançoisDi Baldassarre, AngelaPoudel, KritikaDi Battista, SilviaPoulain, TanjaDi Bella, EnricoPoulakis, ZeffieDi Blasio, AlbertoPouliakis, AbrahamDi Bona, GianpaoloPouliaris, ChristosDi Carlo, PaolaPoulicek, Mathieu E. A.Di Corrado, DonatellaPoulidis, Alexandros PanagiotisDi Corso, EvelinaPoulimeneas, DimitrisDi Curzio, DiegoPoulton, AlisonDi Diego, JosePoulton, Alison SallyDi Dino, BiagioPouranian, M. RezaDi Gangi, StefaniaPourchez, JérémieDi Gennaro, FrancescoPourhejazy, PouryaDi Giacomo, DinaPourová, JanaDi Giacomo, SilviaPousada, ThaisDi Giallonardo, FrancescaPovey, AndrewDi Giovanni, PamelaPovilanskas, RamūnasDi Giuseppe, DarioPowell, JamesDi Liddo, AndreaPowell, TaraDi Liegro, ItaliaPowell-Wiley, Tiffany M.Di Lodovico, FrancescaPower, DavidDi Lonardo, SaraPower, JenniferDi Lorenzo, FrancescoPower, Thomas G.Di Lorenzo, MarianaPowers, James S.Di Lorito, ClaudioPowrózek, TomaszDi Maggio, IlariaPozo Devoto, VictorioDi Martino, CatelloPozo Llorente, María TeresaDi Martino, FerdinandoPozo Sánchez, SantiagoDi Martino, GiuseppePozo-Rico, TeresaDi Mascio, PaolaPrabhu, Subbaiah MuthuDi Meglio, AntonioPraça, GibsonDi Miceli, MathieuPrada, Christopher FernandezDi Michele, LoretaPradhan, AjayDi Nicola, MarcoPradhan, PrajalDi Paolo, Carlo D.Pradhan, ShankaliDi Pasqua, Laura GiuseppinaPrado Da Fonseca, EmílioDi Profio, FedericaPragai Olswang, GillieDi Renzo, LauraPrakasarao Kovvasu, SuryaDi Spirito, FedericaPranckevičienė, AistėDi Stefano, GiovanniPranckeviciene, ErinijaDi Tecco, CristinaPranggono, BernardiDi Tella, MarialauraPranskūnienė, RasaDi Vaio, AssuntaPrashad, ShikhaDi Vincenzo, AngeloPrat, GemmaDi Vito, MauraPratap Singh, AnubhavDi Zazzo, ErikaPraticò, SalvatoreDi, GuoqingPrattichizzo, FrancescoDi, ZhenhuaPravst, IgorDiab, RamiPrchal, Josef T.Diachkova, EkaterinaPrecioso, JoseDiaconu, CameliaPreez, Surita DuDiaconu, KarinPregnolato, MariaDiakakis, MichalisPregowska, AgnieszkaDiamantis, Vasileios I.Presbitero, PatriziaDias Junior, Antonio GregorioPrescott, MelissaDias Pereira, LuisaPresentato, AlessandroDias, Albino AlvesPressyanov, DobromirDias, Cláudia SilvaPrete, Maria IreneDias, DuartePreti, MarioDias, Fernando SuparreguiPretrel, HuguesDias, Sofia B.Prezant, DavidDiawara, Moussa M.Price, GordonDíaz Agea, José LuisPrice, Matt A.Diaz Balzani, Lorenzo AlirioPrice, PatriciaDiaz Crescitelli, Matias EduardoPridmore, JasonDíaz Delgado, CarlosPridmore, Saxby A.Díaz Hernández, MatildePrieger, James E.Díaz- Noci, JavierPriego De La Cruz, Alba MaríaDíaz Redondo, Rebeca P.Prieler, MichaelDiaz Roman, AmparoPrieske, OlafDiaz, AbbeyPrieto Ayuso, AlejandroDiaz, Aimee M.Prieto, Andrés J.Díaz, AmeliaPrieto, LeonelDiaz, AnjoliiPrieto, Miguel A.Diaz, DiegoPrieto-Gutiérrez, Juan JoséDíaz-Caro, CarlosPrieto-Lloret, JesusDíaz-De Alba, MargaritaPrieto-Ursúa, MaríaDiaz-Delcastillo, MartaPrimeaux, StefanyDíaz-Fúnez, Pedro A.Printza, NikoletaDiazgranados, NancyPrinzen, FritsDiaz-Hernandez, Jose LuisPrior, Jerilynn C.Díaz-Meneses, GonzaloPrior, SarahDíaz-Milanés, DiegoPrioreschi, AlessandraDiaz-Sarachaga, Jose ManuelPripp, Are HugoDiaz-Silveira, CintiaPrist, Paula RibeiroDíaz-Simal, PedroPritikin, JoshuaDíaz-Zavala, Rolando G.Privitera, DonatellaDibaba, DanielPrivitera, GaetanoDickert, Franz L.Privitera, Michael R.Dickey, JimPriyadarshini, AnushreeDickin, ClarkPriyadarshini, MedhaDickson, Joanne M.Priyankara, Hewawasam Puwakpitiyage Dicu, TiberiusProbst, Tahira M.Didaskalou, EleniProchaska, CharikleiaDiderichsen, FinnPróchniak, PiotrDidham, RobertProchor, PiotrDidkowska, JoannaProcter, PaulaDidon, Jean-PhilippeProcter-Gray, ElizabethDiefenbach-Elstob, TanyaProdanovic, VeljkoDiego, Vincent P.Proença De Oliveira, RodrigoDieguez, TeresaProgler, JosephDiehl, Jessica AnnProia, PatriziaDiémoz, HenriPronost, GuillaumeDiener, Ed F.Protano, CarmelaDierkes, JuttaProtudjer, Jennifer L. P.Dierkes, Paul WilhelmProvenzano, DanieleDietch, JessicaProvini, FedericaDieterich-Hartwell, RebekkaProvot, ThomasDietrich, CharlesPruden, AmyDietscher, ChristinaPrudêncio, MiguelDíez Bosch, MíriamPruitt, SethDíez, JuliaPrunty, Leesa M.Díez-Bedmar, María Del ConsueloPrus, BarbaraDíez-Gómez, AdrianaPrus, PiotrDíez-Gutiérrez, Enrique-JavierPryce, RobertDifrancisco-Donoghue, JoannePryor, WesleyDiglio, AntonioPryss, RüdigerDijkstra, AriePrzednowek, Karolina H.Dijkstra, AtzePrzednowek, KrzysztofDikken, JeroenPrzybilla, JensDiko, Stephen KofiPrzybyła, KatarzynaDikshit, AbhirupPrzybylowicz, KatarzynaDillon, DenisePrzytuła, SylwiaDillon, HarveyPsarris, AlexandrosDilts, JenniferPsomopoulos, Constantinos S.Dilworth, RichardsonPsychogios, GeorgiosDiMenna, FrederickPu, BoDimitrijevic, ZoricaPu, ChristyDimitropoulos, PanagiotisPucci, LauraDimka, JessicaPucci, ResiDimopoulos, AndreasPuchalka, RadoslawDimri, ManaliPuchinger, MarkusDimyadi, Johannes Alwi WibisonoPuciato, DanielDinas, PetrosPuckett, Jae A.Ding, KaimengPueo, BasilioDing, MengPuertas Molero, PilarDing, NingPugh, BrandieDing, ShiyongPugliese, MariagabriellaDing, WeiPugnetti, CarloDing, XinruiPuig, SebastiàDing, XuhuiPuig-Barrachina, VanessaDing, YichunPujilestari, Cahya UtamieDingil, Ali EnesPujol, AïdaDinis, Maria Alzira PimentaPulakka, AnnaDinis, Maria De LurdesPulay, AlanaDinis, PedroPulgar, VictorDiNitto, Diana M.Pulidindi, Indra NeelDino, GeriPulido Fernández, ManuelDintrans, Pablo VillalobosPulido, Cristina M.Diogo, ElisetePulido, OlgaDiogo, PatriciaPulido-Moncada, MansoniaDioguardi, MarioPuljevic, ChenealDiolaiuti, FrancescaPulker, ClaireDion, KimberlyPullan, JerryDionigi, RyleePulle, Ranjeev ChrysanthDionysopoulos, DimitriosPullinen, IidaDios Sánchez, IrenePulvirenti, GiuliaDipaola, FrancaPun, MatiramDipnall, JoannaPuntillo, PinaDiquattro, StefaniaPuppo, FrancescaDiraco, GiovanniPuri, KritiDirks, KimPuri, SonamDirksen, DieterPursiainen, ChristerDirzyte, AistePuszkarewicz, AlicjaDistaso, MonicaPutrino, DavidDitta, AllahPuttilli, Matteo GirolamoDivakaruni, SrinivasPuukila, StephanieDivella, RosaPuustinen, PietriDixon, DanielPuzianowska-Kuźnicka, MonikaDixon, P. GradyPuzon, Geoffrey J.Dixon, Robyn ShirleyPycia, KarolinaDixon, SandraPyke, Robert E.Djärv, TheresePynnönen, KatjaDjerada, ZoubirPypłacz, PaulaDlouha, JanaPyrgaki, KonstantinaDludla, Phiwayinkosi V.Pyrri, IoannaDługosz, OlgaPyżalski, JacekDluholucký, SvetozárQadri, GhalibDłuski, DominikQadri, TalatDo Monte, Daniel F. M.Qanud, KhaledDo Vale, FranciscoQasim, MuhammadDo, Elizabeth K.Qasim, SalmaDo, LocQeadan, FaresDo, ThuyQi, AimingDoarn, CharlesQi, GuanqiuDobe, ChristopherQi, HangDobešová, ZdenaQi, JunyuDobrovolny, Hana M.Qi, QibinDobrowolska, BeataQi, YanDobrowolski, HubertQi, YanbinDobrzyński, MaciejQi, YiDobson, MarnieQian, Cheryl ZhenyuDobson, RuaraidhQian, Wei (Australia)Docasar, Edurne ÚbedaQian, Wei (USA)Dockrell, MartinQian, XunDoda, Sai ReddyQiao, HuanyuDodd, RachaelQin, FeiDoddapattar, PrakashQin, LuyeDodd-Reynolds, Caroline J.Qin, SanboDodge, ElizabethQin, ShulinDoerr, StefanQin, TongDoevendans, KeesQin, ZhaohaiDoherty, KathleenQin, ZhenzhenDoimo, IlariaQing, YangDoka, Kenneth J.Qiu, GuangyuDolezel, Diane M.Qiu, LingDolezel, JakubQiu, RongxuDołhańczuk-Śródka, AgnieszkaQiu, ShaopingDollahite, DavidQiu, XiuDolníková, ErikaQiu, YongDolores López Franco, MaríaQu, HaiyanDolzhenko, Anton V.Qu, MingziDomagala, AlicjaQu, NingDomaradzki, JarosławQu, ShaojianDombrowski, TobiasQuadroni, SilviaDomek, Gretchen J.Quam-Wickham, NancyDomen, AndreasQuan, MinghuiDoménech, FernandoQuan, Stuart F.Domenicali, MarcoQuan, TaihaoDomingues, EvaQuandt, SaraDomínguez Díaz, LauraQuanlei, YuDomínguez, RaulQuaranta, AlessandroDomínguez-Delgado, AntonioQuaranta, GiovanniDominguez-Maldonado, GabrielQuartu, MarinaDomínguez-Pérez, DanyQuattrocelli, MattiaDomínguez-Salas, SaraQuattropani, MariaDomínguez-Vías, GermánQuddus, NoorDominguez-Vicent, AlbertoQudrat-Ullah, HassanDominika, Jamioł-MilcQueirós, CristinaDominski, FabioQuej, Victor H.Dommerholt, JanQuek, TianDon, Aakash WelgamageQuémerais, BernadetteDonadel, GiuliaQuendler, ElisabethDonahue, PaulaQuerner, PascalDonald, Bryan J.Quest, DaleDonaldson, ShawnQuevedo, LuisaDonat, Francisco Javier SilvestreQuevedo-Blasco, RaúlDonati, Maria AnnaQuide, YannDoña-Toledo, LuisQuigley, John G.Donchin, MilkaQuiles, NorbertoDondi, AriannaQuílez-Robres, AlbertoDoneddu, AzzurraQuince, Thelma A.Donell, LorieQuinlan, JonathanDonevant, Sara BelleQuinn, Megan A.Dong, DongQuintana-Murci, ElenaDong, FaqinQuintela, Joana Alegria Ferreira DaDong, FengQuintero M., Christian G.Dong, FengpingQuinteros, Maria ElisaDong, JianjunQuintiliani, Lisa M.Dong, JingQuintiliano Scarpelli, Daiana A.Dong, QinglinQuiroga, EnedinaDong, ShangjiaQuirós-Alcalá, LesliamDong, ShengzhangQuoc Le, Trung (Tim)Dong, SijunQuock, Raymond M.Dong, WeizhenRab, AndrásDong, YajieRábago, DanielDong, YuchengRabbani, Mohammad MahbubDong, ZengchuanRabinowitz, SamuelDong, ZhichaoRabionet, Silvia E.Dongho, Ghyslaine Bruna DjeunangRabito, Felicia A.Donisi, ValeriaRabkin, Simon W.Donizzetti, Anna RosaRabuazzo, Agata MariaDonkor, EmmanuelRace, MarcoDonnelly, DavidRachek, LyudmilaDonnelly, LeeannRachel, MartaDonner, ReikRachon, DominikDono, JoanneRachwał, TomaszDonohue, WilliamRachwalski, MichałDonovan, Robert J.Rachyla, IrynaDonti, AnastasiaRacine, MélanieDonti, OlyviaRacionero-Plaza, SandraDonzelli, GabrieleRaciti, MariaDoo, MiaeRaczkowska, EwaDoody, OwenRada, ElenaDooley, ErinRadadiya, AshishDopelt, KerenRadakovic, RatkoDopico Calvo, XurxoRadanielson, AndoDora, JonasRadicetti, EmanueleĐorđević, ZoranaRadicic, DraganaDore, BettyRadin, MichaelDorea, CaetanoRadišauskas, RičardasDoria, CataldoRadko, LidiaDorian, PaulRadon, KatjaDöring, NicolaRadoslavov, GeorgiDörk, ThiloRadun, JenniDornelles, AdrianaRadwan, MichałDorney, EdwinaRadzi, HafiezalDos Anjos Pires, MariaRadzimiński, ŁukaszDos Anjos, Sarah M.Radzka, ElżbietaDos Santos Müller, JulianaRafalson, LisaDos Santos Silva, Fabrício DanielRaffegeau, TiphanieDos Santos, Fábio A.AbadeRaffenaud, AmandaDos Santos, Klinsmann CaroloRaffi, MilenaDos Santos, Luis MiguelRafieenia, RaziehDos Santos, Mário Gabriel SantiagoRagain, ChristinaDosil, MariaRagazzi, MarcoDosoky, NouraRaghavendra, AjayDossou, Paul EricRaghupathi, WullianallurDoublet, VincentRagonese, PaoloDoucoure, SouleymaneRagosta, MariaDougherty, Michael JohnRagozino, StefaniaDougherty, MichalRagusa, Angela T.Douglas, Jason A.Raha, DebadayitaDoukakis, SpyrosRahaman, KhanDoulamis, IliasRaheem, DeleDoulberis, MichaelRahi, BernaDoulgeris, CharalamposRahimi, FaridDouzi, WafaRahmadani, FirdaDow, BrionyRahmadani, Vivi GusriniDow, Margaret L.Rahman, ArifurDowgier, GiuliaRahman, AsadurDowland, Samson N.Rahman, AshiqurDowney, F. JavierRahman, AteequrDowning, AndreaRahman, AyeshaDowning, StevenRahman, AzizurDoyle, FrankRahman, HafizurDrachal, KrzysztofRahman, JuberDrachev, Sergei N.Rahman, Md AnisurDragioti, ElenaRahman, MizanoorDragomir, AliceRahman, MuhibUrDragonieri, SilvanoRahman, QuaziDrago-Serrano, Maria ElisaRahman, RahbelDrake, RossRahman, SafikurDrake, ShawnRahman, SaidurDrane, PatrickRahmani, DjamelDrápela, EmilRahmati-Abkenar, MahboubehDraper, AlizonRahme, KamilDraper, CatherineRahnama, MostafaDraus, PaulRahnavard, GholamaliDrava, GiulianaRahoi-Gilchrest, RitaDreassi, EmanuelaRai, NavneetDreborg, StenRai, Nayanjot K.Dresden, BrookeRai, Nayanjot KaurDresler, SławomirRai, VikrantDresp-Langley, BirgittaRaikes, Adam C.Drevet, GabrielleRaikhy, GauravDreyer, MarcRaimondo, DiegoDriezen, PeteRaimondo, SergioDrigas, Athanasios S.Raimundo, Armando Manuel De MendonçaDriss, TarakRainbow, Jessica G.Drobná, HelenaRainoldi, AlbertoDrott, JennyRaiola, GaetanoDrozd, RadoslawRaisamo, SusannaDrozd, ValentinaRäisänen, AnuDrozdovitch, VladimirRaišienė, Agota GiedrėDrozdowicz, JaremaRaison, BrianDrum, ScottRaj, EldonDrummer, OlafRaja, Afia ZubairDrygas, WojciechRajagopalan, AnugrahaDrywień, Małgorzata EwaRajan, BalaramanDu, ChangliangRajaram, DushhyanthDu, JiangtaoRajendiran, KarthikrajDu, JianxiaRajendran, Jagathesh ChandraDu, ShufaRajnarayanan, RajendramDu, ZhenfangRajnik, MichaelDuan, FengRajpurohit, SurendraDuan, HanchenRajtar-Zembaty, AnnaDuan, JianRaju, Muppala N. P.Duangthip, DuangpornRak, JanuszDuarte, Anderson RibeiroRakic, JelenaDuarte, José B.Ralli, MassimoDuarte, MarcosRama, LuísDubakiene, RutaRamachandiran, IyappanDubas-Jakóbczyk, KatarzynaRamachandran, RoshiniDubé, CatherineRamachandran, SujithDube, KaitanoRamada-Rodilla, José MaríaDubé, MathieuRamadas, AmuthaDube, PrabhatchandraRamakrishnan Geethakumari, PraveenDuberg, AnnaRamalho, AndréDubey, HarishchandraRamalingam, SundarDubey, JitenderRamamurthi, RamDubey, NileshkumarRamanathan, RamakrishnanDubinin, Mikhail V.Ramaswamy, Sai K.Duby, ZoeRamatchandirin, BalamuruganDucati, Jorge RicardoRambanapasi, ClintonDuchhart, IngridRameckers, Eugene A.Duchowny, Kate A.Ramilo, DavidDückers, MichelRamirez Harrington, DonnaDuckett, Jeffrey G.Ramírez Jiménez, María SalvadoraDucret, MaximeRamirez Lopez, Leonardo JuanDucza, EszterRamírez Soto, Max CarlosDuczmal-Czernikiewicz, AgataRamirez, BernardoDudek, HannaRamirez, JavierDudek, MichałRamírez, Jorge IvanDudek, TomaszRamirez, Paul MichaelDudycz, HelenaRamirez-Baena, LuciaDudziak, AgnieszkaRamirez-Castellanos, JulioDudzinski, David M.Ramirez-Graniz, Irwin AndrésDuehlmeyer, LeonieRamírez-Moreno, José MaríaDueñas, Jorge-ManuelRamírez-Santana, MurielDugan, B. DeeAnnRamírez-Vélez, RobinsonDugar, RohitRamiro-Cortijo, DavidDugdale, JamesRamis, RebecaDuggan, JackieRammah, AmalDugoua, EugenieRamoelo, AbelDuh, Feng-ChyiRamon, Peral OrtsDuijnhoven, J. VanRamon, ShulamitDuivon, MylèneRamos Campos, FranciscoDuker, Leah SteinRamos González, VictoriaDukić, WalterRamos Martinez, AntonioDuleba, SzabolcsRamos Salazar, LeslieDulebenets, Maxim A.Ramos, Célia M. Q.Dumas, OrianneRamos, Delfina Gabriela GarridoDumic, IgorRamos, DomingoDumitrache, LilianaRamos, GeorgeDumitras, DianaRamos, João António EstevesDumitriu, DanRamos, Jorge Humberto PalmeiraDumont, LaurenceRamos, JoseDumuid, DotRamos, PedroDunaway, MaryRamos, Salvador BoccalettiDunbar, Michael S.Ramos, SóniaDuncan, EarlRamos-Garcia, PabloDuncan, IshbelRamos-Jiménez, ArnulfoDuncan, MikeRamos-López, DaríoDuncan, MitchRamos-Martin, JesusDuncan, OllyRamos-Monserrat, MaríaDundes, LaurenRamos-Morcillo, Antonio JesúsDunford, CarolynRamos-Pichardo, Juan DiegoDunisławska, AleksandraRamos-Vidal, IgnacioDunning, TrishaRampedi, IsaacDunst, Carl J.Ramsauer, BabettDuong, PhuRamsay, Michael A.Duong, Tuyen VanRamsbottom, RogerDuong-Quy, SyRamsden, VivianDuplaga, MariuszRamsey, JohnDupont, JolanRan, JinjunDupont, Nana HeeRanabhat, ChhabiDuPont, RobertRancourt, Rebecca C.Duport, SophieRandall, AlanDuppati, GeetaRanderson, PeterDupre, KarineRandjelovic, PavleDupuis, KateRandle, HayleyDuquenne, Marie NoelleRandler, ChristophDuradoni, MirkoRando-Cueto, DoloresDurán Poveda, ManuelRangel, VictorDurand-Moreau, QuentinRangelov, NatalieDurand-Zaleski, IsabelleRania, NadiaDurán-Riveroll, LorenaRanieri, MariannaDuranti, GuglielmoRanjan, KishuDurazzo, AlessandraRanjan, PeeyushDurdyev, SerdarRansdell, LyndaDurkalec-Michalski, KrzysztofRanstam, JonasDurocher, JohnRantala, AndreasDurowoju, Olatunde S.Rantanen, TainaDuscher, Georg GerhardRanzato, EliaDusenge, Mirindi EricRao, FaizaDushenkov, VyacheslavRao, FujieDusso, AdrianaRao, Gundu H. R.Dutartre, PatrickRao, MuraliDutcher, DabrinaRao, Sneha B.Dutra, Andeise CerqueiraRao, VidhyaDutra, Samia V.Rapa, MattiaDutta, SanjuctaRapado-González, ÓscarDuursma, Elisabeth E.Rapisarda, FilippoDuyar, IbrahimRapisarda, VenerandoDwivedi, GarimaRapley, Bruce I.Dwyer, Rocky J.Rapone, BiagioDyduch, JustynaRaposo, AntónioDymitrow, MirekRaposo, VítorDzakpasu, MawuliRapp, PaulDzambas, TamaraRappe, ErjaDziechciarz, JózefRappelli, GiorgioDziechciarz, MartaRaquel, Aparicio-UgarrizaDziedzic, ArkadiuszRaraigh, Karen S.Dziekański, PawełRäsänen, KimmoDzielska, AnnaRascher, WolfgangDzierżanowski, TomaszRasiah, RohanDziga, DariuszRasika Dzikuć, MaciejRasmussen, MetteDzikuć, MariaRasmussen, Torben SølbeckDziurka, DorotaRasool, Sama FaizDziurkowska, EwelinaRaspa, MelissaDzobo, KevinRastegari, ElhamDzwonkowska, DominikaRastelli, EugenioEagle, Shawn R.Rastenytė, DaivaEast, KatherineRastogi, UjjwalEastman, CreswellRaszkowski, AndrzejEastman, RonaldRatajczak, KarolinaEaston, SusanRatajczak, KatarzynaEastwood, GillianRatajczak, MagdalenaEather, NarelleRatajczak, MonikaEaton, Kenneth A.Ratchford, SteveEaton, SimonRatheesh, VaishnaviEbenso, BasseyRather, IrfanEberhard, JoergRathi, NehaEbersviller, SethRathmacher, JohnEbisu, KeitaRathmann, JoachimEbrahimi Kalan, MohammadRathnayake, SarathEbrihimnejad, SaeedRathousky, JiriEcarnot, FionaRatkowsky, DavidEcelbarger, Carolyn A.Rato, Luís Pedro FerreiraEchavarria, RaquelRatsch, AngelaEcheverri, MargaritaRattanapan, CheerawitEcheverría, Carmen FernándezRatter, BeateEcke, ThorstenRauch, BernhardEckel-Mahan, Kristin L.Rauf, AbdulEckert, Katharina G.Raundhal, MaheshEckert, Mark A.Rautanen, Sanna-LeenaEckhardt, JappeRavagnan, GiampietroEckhoff, Rolf KristianRaval, Manmeet H.Economou, AthinaRaval, YashEddy, Brandon P.Ravanidis, StylianosEde, AlisonRavara, SofiaEden, SusannaRaven, MelissaEdenborough, KathrynRavin, James G.Edes, Ashley N.Ravina, MarcoEdfort, EricaRavuri, SudheerEdgerton, JasonRawassizadeh, RezaEdgeston, AnnaRawla, PrashanthEdler, DennisRay, SamriddhaEdmonds, RohanRay, SujayEdmonston, ColinRaya Félix, AntonioEdmunds, Lia R.Raya-González, JavierEdna, CabecinhaRayamajhee, VeeshanEdoh, Thierry OscarRaynes-Greenow, CamilleEdokpayi, Joshua NosaRaysoni, Amit U.Eduardo, KrugerRaz, OlgaEdwards, HelenRazmjou, HelenEdwards, Lowri C.Raźniak, PiotrEdwards, PeterRazvan, MohammadrezaEedara, Basanth BabuRazvan-Cosmin, PetcaEensaar, AguRazzaque, Mohammed S.Effertz, TobiasRe, DinoEfird, Jimmy T.Rea, CorinnaEfthimiou, GeorgeRead, DonnaEgana Gorrono, LanderReading, Stacey AllanEgawa, ShinichiReardon, Catherine A.Egbert, Jeremy R.Rebelo, Maria Augusta BessaEgberts, AngeliqueRebelo, SandraEgea Zerolo, BlancaRebelo-Gonçalves, RicardoEgerman, RobertRebessi, FilippoEggers, MariRebollo-Hernanz, MiguelEgizi, AndreaReca, Octavio LuqueEhn, MariaRecalcati, SebastianoEhret, Megan J.Recanatesi, FabioEhrhardt, MatthiasRecchia, Daniela R.Ehrlich, J. ShoshannaRechulicz, JacekEichwald, CatherineReddy, Ravi KrishnanEid, DeebRedfern, LaurenEiguren-Fernandez, ArantzazuRedina, OlgaEirín, RaúlRedman, ErinEiroa-Orosa, Francisco JoséRedondo, GiselaEisenbeck, NikolettRedondo-Castán, Juan CarlosEitaro, KodaniRedondo-Sama, GiselaEizaguirre, Josu SolabarrietaRedshaw, SarahEjidike, Ikechukwu P.Redvers, NicoleEkblad, SolvigRedwine, DavidEkblom, Örjan B.Reece, JeanetteEkblom, PaulReeck, CrystalEkenga, Christine C.Reed, Edward J.Ekkel, DinandReed, Matthew P.Ekkel, E. DinandReed, William R.Eklund Karlsson, LeenaReedman, SarahEkman, BjörnReers, StefanEkúndayò, Olúgbémiga T.Reese, GerhardEkuni, DaisukeReeson, Andrew F.El Ghoch, MarwanReeve, KathleenEl Hadi, HamzaReeves, AdamEl Kadhi, NabilRefaeli, TehilaEl Khaled, DaliaRegel-Rosocka, MagdalenaEl Khalloufi, FatimaRegmi, KrishnaEl Midaoui, AdilRegueiro, BibianaEl Muayed, MalekRehman, MichaelEl Zowalaty, Mohamed E.Rehman, SarishElahi, EhsanReibel, TracyElasra, AmiraReich, AdamEl-Awad, UsamaReichenbach, Zachary WilmerElbadry, Maha A.Reichert, MarkusElbarazi, IffatReichert, PawełElbarbary, MonaReicks, MarlaElbl, JakubReid, KennethElboj, CarmenReid, NatashaElce, AusiliaReidenberg, Daniel J.El-Dars, FaridaReiersen, JonEleftheriadis, TheodorosReif, Kathryn E.Elekes, ZsuzsannaReif, SimonElena, Bernabéu-BrotónsReifinger, MartinEleuteri, StefanoReifsnider, ElizabethElezkurtaj, SeferReigal, RafaelElf, Jessica L.Reile, RainerElf, MarieReilly, Kathleen H.Elflein, Heike MariaReiman, ArtoElfrink, Marlies E. C.Reimer, UlrichElgendy, MostafaReina Bueno, MaríaElgorriaga-Astondoa, EdurneReina, RaulElguezabal, PeruReinbach, Helene ChristineElhai, Jon D.Reindl, JudithElia, MaurizioReineholm, CathrineElias, AmanuelReinhardt, Jan DietrichElias, BrendaReis, AnabelaElias, SaidReis, ClariceElinder, Carl-GustafReis, ClaudiaElkasabi, MahmoudReis, ElizabethElkins, JeananneReis, JoanaElledge, Myles F.Reis, Lara AleluiaEllina, GeorgiaReis, VictorElliot, SusanReisz, SamanthaElliott, CharleneReiter, AbigailElliott, EliseReiter, E. MirandaEllis, ShelleyReja, Md YousufEllis, Sonja J.Rejman, MarekEllison, NickRelinque, FernandoElmer, Shandell L.Relph, NicolaElmesiry, AhmedRelvas, HelderElmore-Staton, LoriRemedios, Louisa J.Elmouki, IliasRemigante, AlessiaElosegi, ArturoRemmenga, Steven W.Elsadek, Mohamed A.Remmers, TeunElsayad, KhaledRempel, DavidEl-Sayed, Marwa M. H.Ren, BaozengEl-Sheikh Ali, HossamRen, DaicaiElstad, Jon I.Ren, HongyanElsts, AtisRen, XipeiElvik, RuneRen, YanjunElvira, NicaRen, ZhanbingEly, GretchenRen, ZhenhuaEl-Zaria, MohamedRen, ZhihongEme, PaulRenart, GemmaEmerson, Dawn M.Rendtorff, Jacob DahlEmery, Rebecca L.Rendueles, ManuelEmmanouil, ChristinaRene, EldonEmmanouil, DimitrisRennels, Jennifer L.Emmanuel, EvensRenni, Marcos JoséEmmert, HilaRenta-Davids, Ana-InésEmpler, TommasoRentería-Villalobos, MarusiaEndevelt, RonitRenwick, KerryEndo, AikoRenzi, AlessiaEndo, AkiraReoyo, Olga RoldanEndo, HidekiRequena, CarmenEndo, MakotoRequicha, JoaoEndo, Patricia TakakoRescia, AlejandroEneriz-Wiemer, MonicaResentini, FrancescaEngel, JonathanResiere, DaborEngelen, Peter JanReszka, EdytaEngelhardt, Paul E.Retamal-Salgado, JorgeEngeset, DagrunRettig, RasmusEngland, MarionRety, StephaneEngland, SarahReuter, StefanEngle-Stone, ReinaReuter-Oppermann, MelanieEnglund, TessaRevilla, Raquel GarcíaEngvall, JanRevuelta Iniesta, RaquelEnnaji, MohaRey, EzequielEnnequin, GaelRey, GrégoireEnnigkeit, FabienneRey, TapasEnnis, EdelReybrouck, MarkEnrico, Gherlone FeliceReyes Menéndez, AnaEnsenyat, AssumptaReyes-Aldasoro, Constantino CarlosEnsley, SteveReymond, DavidEnthoven, PaulReynaga-Ornelas, LuxanaEntwistle, MarcelaReynard, OlivierEnwere, MichaelReynes, BarbaraEnyioha, ChinemeReynolds, ClareEpaulard, OlivierReynolds, JamesEpelde, FranciscoReynolds, JoannaEpelde, GorkaReynolds, LoriEpping, Eric A.Reynolds, MichaelEpstein, Scott A.Rey-Reñones, CristinaEramo, StefanoRezaei, MahdiErazo, Marcia B.Rezania, ShahabaldinErb, CarlRezapour Mashhadi, Mohammad MahdiErceg, AleksandarReznik, Jacqueline E.Erickson, Mary AnnReznikov, Leah R.Eriguchi, MasahiroRezvani, KhosrowEriksson, CharliRhee, Seung-YoonEriksson, JohanRhein, MathiasErmolao, AndreaRhi, Seok-HoErmolin, MikhailRho, HojungErnesto, Cortés-CastellRhoades, MarthaEroglu, AbdulkerimRhodes, Diane McDanielEronen, JohannaRhodes, RyanErram, DineshRiad, AbanoubErreygers, GuidoRial Rebullido, TamaraErvas, FrancescaRiar, Amanjot KaurErwich, Jan Jaap H. M.Ribeiro Pinto, JoãoErwin, HeatherRibeiro, AlexandreErythropel, HannoRibeiro, AnabelaEsangbedo, Moses OlabheleRibeiro, ApoenaEscario, José-JuliánRibeiro, ArturEschweiler, Gerhard W.Ribeiro, ElsaEscobar, IsabelRibeiro, HelenaEscobar, Kurt A.Ribeiro, Jessica D.Escobar-Gómez, ElíasRibeiro, PaulaEscobedo Monge, Marlene FabiolaRibeiro, PauloEscolano-Pérez, ElenaRibeiro, Sergio SilvaEscolar-Llamazares, M. CaminoRibeiro, ThierryEscortell, RaquelRibot, MiquelEscoto Castillo, AnaRicard, Lykke MargotEscribano Cubas, SilviaRiccardo, Di GiminianiEscribano-Sotos, FranciscoRicci, AlessioEscrig-Olmedo, ElenaRicci, ClaudiaEscriva, MariaRicci, EleonoraEscudero-Gómez, Luis AlfonsoRicciardi, GualtieroEscuer-Gatius, JordiRiccio, AngeloEscuriet, RamonRiccio, GiuseppeEseadi, ChieduRiccò, MatteoEsfahani, Shiva HadiRice, Robert H.Esfandyari, SaharRice, Simon M.Esmaeli, BehzadRich, KyleEsnard, Talia RandaRich, NicholasEspa, StefaniaRichalet, Jean-PaulEspada, MárioRichard, MelissaEsparham, AnnaRichards, Dylan J.Espejo-Antúnez, LuisRichards, EmmaEspino, JaimeRichards, NgaioEspinosa-Reyes, GuillermoRichards, NoniEspinoza-Castro, BernardaRichards, SeanEspluga, JosepRichardsen, AstridEsposito De Vita, GabriellaRichardson, AliceEsposito, GianlucaRichardson, Carolyn M.Esposito, MariaRichardson, KathleenEspoz-Lazo, SebastiánRichardson, Matthew L.Esquerra-Zwiers, AnitaRichmond, JanetEssa, WiesamRichmond, PeterEsser, AndréRichmond, RobertEsser, JoachimRich-Ruiz, ManuelEstape, Estela S.Richter, ChrisEstarlich, MarisaRichter, KneginjaEstepa, RafaelRicker, BrittaEstevan, IsaacRico-González, MarkelEsteve, FrancescRider, CynthiaEsteve-Romero, JosepRidley, JosephineEstévez, EstefaníaRidley, KateEstévez, Javier GualdaRidley, SusanEsteve-Zarazaga, Maria RosaRie, Dong-HoEstravís, MiguelRiebe, DeborahEttayapuram Ramaprasad, Azhagiya Riedel, FabianEttorre, EvaristoRieder, MichaelEtxebarria, NaroaRiederer, AnneEudald, CasalsRiediker, MichaelEustace, StevenRiemma, GaetanoEva, HlaváčkováRienecke, ReneeEvans, AngelaRienmüller, AnnaEvans, ChrisRienzo, CinziaEvans, David S.Ries, FrancisEvans, Elizabeth S.Ries, Julie D.Evanson, Nathan K.Rietra, ReneEvengård, BirgittaRifkin, SusanEvens, AnneRigby, MichaelEveraert, KarelRiggs, ElishaEves, Frank F.Riggsbee, KristinEwart, JacquelineRighetti, LauraExadaktylos, AristomenisRiiser, AmundExbrayat, Jean-MarieRikkert, Marcel OldeExel, JulianaRikmann, ErgoExpósito López, JorgeRiley, Carley L.Extremera, NatalioRiley, MalcolmExum, NatalieRiley, NicholasEysel-Gosepath, KatrinRiley, William J.Eytcheson, StephanieRim, Kyung TaekEzzati Amini, RojaRimaitis, KęstutisEzzeddine, ZeinabRimal, BhagawatF. Santos, AndreiaRimando, JeremyFaal, HannahRimarova, KvetoslavaFabbian, FabioRimehaug, TormodFaber, IreneRinaldi, CarmelaFabiani, RobertoRincón, ElviraFabiano, BrunoRingseis, RobertFabio, Rosa AngelaRinninella, EmanueleFabre, ClaudineRintamaki, LanceFacal, DavidRio, EbonieFacca, ChiaraRiojas Rodriguez, HoracioFacchin, AlessioRíos-Silva, MónicaFacchinetti, FabioRiper, David VanFacchinetti, GabriellaRipollone, JohnFaccilongo, NicolaRiquelme, Alejandro GuillénFaccini, BarbaraRisch, EricFactor-Litvak, PamRisco-Castillo, VeronicaFaedda, NoemiRishworth, AndreaFaes, ChristelRisica, Patricia M.Faezipour, MisaghRiso, Eva-MariaFagan, G. HonorRissanen, RitvaFagan-Endres, MarijkeRistić-Medić, DanijelaFageda, XavierRistorini, MartinaFagerstrom, KarlRistović, IvicaFaghy, MarkRitchie, CarrieFahes, MashhadRitchie, Elspeth CameronFahlbusch, Fabian B.Ritchie, HelenaFairbank, JeremyRitchie, Lorrene D.Fairchild, GeoffreyRitter, AlexanderFaita, FrancescoRiva, GiuseppeFajardo Del Castillo, TeresaRiver, JoFajardo-Bullón, FernandoRivera De La Rosa, JavierFajfr, MiroslavRivera Del Álamo, Maria MontserratFakih, AliRivera, GilbertoFakoya, MichaelRivera, Jason D.Fala, NicolettaRivera, VictorFalandysz, JerzyRivera-Loaiza, CuauhtémocFalcon, MariaRivera-Navarro, JesúsFalcone, Pasquale MarcelloRivera-Nunez, ZorimarFalconer, Ian R.Riveros-Rosas, HectorFalfán-Valencia, RamcésRivolta, Massimo WalterFalgares, GiorgioRiza, ElenaFaling, MarijnRizk, Elias B.Falk, MartinRizkalla, NiveenFalkowska, EwaRizo-Maestre, CarlosFalla, MarikaRizos, EmmanouilFalup-Pecurariu, OanaRizun, NinaFamà, FaustoRizvi, ImranFamielec, StanisławRizvi, Syed Husain MustafaFamoso, FabioRizwan, MuhammadFan, BinRizzello, FrancescaFan, FeiRizzo, CarmenFan, HongRizzo, GianlucaFan, KeRizzo, GiovannaFan, LinlinRizzo, GiuseppeFan, XiuzhenRizzo, PiervincenzoFancello, GiovannaRizzo, RobertoFanelli, Rosa MariaRizzo, WilliamFang, ChristopherRobards, FionaFang, DiRobas, MarinaFang, Guor-ChengRobb, Nigel D.Fang, JunRobberecht, HarryFang, KegongRoberson Jr., Donald N.Fang, Mei LanRoberta, VenturellaFang, MingzhuRoberto Telles, CharlesFang, Wei-TaRoberts, AeliFang, XinRoberts, EmilyFang, XingRoberts, EvanFang, YaRoberts, Gary E.Fang, YuanhaoRoberts, James A.Fanizza, CarlaRoberts, Jennifer D.Fappi, AlanRoberts, Jessica L.Far, Sharon MalekiRoberts, KenFara, Gaetano MariaRoberts, LisaFarah, Paolo DavideRoberts, MeganFarah, RaymondRoberts, Stephen M.Farahani, AliRobertson, CarolineFarahmand, KambizRobichaud, AlainFaraji, FaridRobila, Valentina V.Faraone, ImmacolataRobillard, Pierre-YvesFarber, Harold J.Robinson, EdwardFareed, AmberRobinson, Guy M.Fareed, NadeemRobinson, JuneFaresjö, TomasRobinson, KimFarfano, Minerva VanegasRobinson, Leslie A.Fargnoli, MarioRobinson, PeterFarhat, GraceRobinson, StevenFaria, SílviaRobischon, MarcelFarina, LauraRobitaille, EricFarkas, KataRobitzsch, AlexanderFarland-Smith, DonnaRobles-Bello, María AuxiliadoraFarmaki, Aliki-EleniRobres, AlbertoFarmer, NicoleRobson, DebbieFarnese, Maria LuisaRobson, Shannon M.Farnham, AndreaRoby, Justin A.Farokhi, Moshtagh R.Roca, Jaime BonnínFaron-Górecka, AgataRocca, ElenaFarran Codina, AndreuRocca, MicheleFarrand, KathleenRoccella, MicheleFarràs-Permanyer, LaiaRoccetti, MarcoFarré, MagiRocchetti, Maria TeresaFarre, NuriaRoccuzzo, SebastianaFarrell III, John WaylandRoch, MajaFarrell, Michael K.Rocha Vaz, JoãoFarris, AlishaRocha, FernandaFarronato, GiampietroRocha, Helena BelchiorFarrow, RobRocha, JoaoFarruggia, PatriziaRocha, SuellenFasina, Folorunso OludayoRocha-Rodrigues, SílviaFasola, SalvatoreRochat, RogerFasullo, MichaelRoche Seruendo, Luis EnriqueFathalla, EissaRoche, MareeFattorini, DanieleRoche, Mary DoyleFaubert, PatrickRochfort, KeithFaulhaber, MartinRochlin, IliaFaulks, DeniseRocio-Bautista, PriscillaFaustini, AnnunziataRockerbie, DuaneFaustmann, AnnaRockloff, MatthewFauzi, IlianaRocklöv, JoacimFavalli, SilviaRoco, LisandroFavara, GiulianaRoczniewska, MartaFavas, PauloRodacki, AndreFavre-Réguillon, AlainRodak, CarolynFavretto, DonataRodakowska, EwaFazal, ZeeshanRodan, AntonioFazzo, LuciaRodby-Bousquet, ElisabetFe Rodríguez Muñoz, MªRodda, SimoneFears, RobinRodenas Motos, PauFeatherstone, JohnRoder, GiuliaFeatherstone, MarkRöder, StefanFebbraio, FerdinandoRodger, JamesFebres, GerardoRodic-Wiersma, LjiljanaFebrisiantosa, AndiRodilla, EnriqueFedele, FrancescoRodrigo Mocholi, DiegoFeder, KatyaRodrigo, LuisFederica, MasciRodrigo-Comino, JesusFederica, SancassianiRodrigo-Ilarri, JavierFederici, ArioRodrigues Domingues, MarlosFedotenkov, IgorRodrigues, AnaFedotova, Julia O.Rodrigues, AngelaFehér, AndrásRodrigues, António S.Fei, Andrew Chang-YoungRodrigues, EugénioFei, JinRodrigues, FilipeFeijó De Almeida, Giovana GorettiRodrigues, Gabriel D.Feijó, Fernando RibasRodrigues, MargaridaFeinberg, IrisRodrigues, MartaFeinman, RichardRodrigues, MatildeFeinn, Richard S.Rodrigues, PedroFekete, AlbertRodrigues, RogérioFekete, AlexanderRodrigues, SandraFekete, ChristineRodrigues-Fernandez, AlejandroFelardo, JeffRodríguez Alvarez, LleretnyFelder, Robin A.Rodríguez Costa, IsabelFeldges, TomRodríguez De Torres, Begoña ArtíñanoFeldman, BeccaRodríguez Domenech, María De Los Feldman, InnaRodríguez García, María Del CarmenFeldman, SteveRodríguez Gutiérrez, José LuisFeldmann, ReinholdRodríguez Hermosa, Juan LuisFélez Nóbrega, MireiaRodríguez Herrero, PabloFelgueiras, HelenaRodríguez Hidalgo, Antonio JesúsFeliciani, ClaudioRodríguez Jiménez, CarmenFelipe, José LuisRodriguez Larrad, AnaFelknor, Sarah A.Rodriguez Lozano, Francisco JavierFell, JamesRodriguez Martin, Jose Antonio (INIA)Felnhofer, AnnaRodríguez Martín, José Antonio (Universidad de Granada)Feng, Chun-ChiangRodriguez Meirinhos, AnaFeng, MeiliRodríguez Mercado, Juan JoséFeng, MuhuaRodriguez Perez, ManuelFeng, QichengRodríguez Sanz, DavidFeng, Sheng-WeiRodríguez Sousa, Antonio AlbertoFeng, ShilunRodríguez Villanueva, JavierFeng, Wei-shengRodriguez y Rodriguez, MartiusFeng, XinglinRodríguez, AnaFeng, YunfeiRodriguez, AnnaFeng, ZhanchunRodríguez, Carlos FreireFeng, Zhixin FrankRodriguez, CelestinoFenga, ConcettinaRodriguez, FernandoFenton, TanisRodríguez, FranciscoFeo, FrancescoRodríguez, José AntonioFeo, RebeccaRodríguez, José CarlosFeola, AlessandroRodriguez, KerriFeola, RosangelaRodriguez, LauraFerenczi, ZitaRodriguez, LuisFerguson, AlesiaRodriguez, Natalia M.Ferguson, BradleyRodríguez, OlgaFerguson, LaurenRodríguez-Almagro, DanielFeria Madueño, AdriánRodríguez-Archilla, AlbertoFeriche, BelénRodriguez-Arrastia, MiguelFerini-Strambi, LuigiRodríguez-Baena, LuisFermani, AlessandraRodríguez-Bolaños, RosibelFermino, Rogério CésarRodriguez-Bravo, BlancaFermo, PaolaRodríguez-Calero, Miguel ÁngelFernandes Rodríguez-Caravaca, GilFernandes Da Silva, SandroRodríguez-Cifuentes, FranciscoFernandes Fagundes, NathaliaRodríguez-Cohard, Juan CarlosFernandes, AntonioRodríguez-Crespo, ErnestoFernandes, Cláudia OliveiraRodríguez-Díaz, LucianoFernandes, CristinaRodríguez-Gómez, IreneFernandes, FranciscoRodríguez-Gómez, Jose ÁngelFernandes, Gisele AparecidaRodriguez-Gonzalez, AlejandroFernandes, Hélder MiguelRodríguez-Hurtado, DianaFernandes, John F. T.Rodríguez-Liébana, José AntonioFernandes, Maria JacintaRodríguez-Lozano, Francisco JavierFernandes, RúbenRodríguez-Martín, Boris C.Fernandez Calderon, FerminRodríguez-Martínez, AntonioFernandez Campoy, Juan MiguelRodríguez-Medina, JairoFernández Carnero, SamuelRodriguez-Merchan, Emerito CarlosFernández Carrasco, Francisco JavierRodriguez-Modroño, PaulaFernández Cruz, ManuelRodriguez-Moreno, Victor M.Fernández De Arroyabe, PabloRodríguez-Muñoz, Pedro ManuelFernández De Las Peñas, CésarRodríguez-Peces, Martín JesúsFernández García, DavidRodriguez-Resendiz, JuvenalFernandez Gonzalez, Jose MariaRodríguez-Roda, IgnasiFernández Gutiérrez, MartinaRodríguez-Rodríguez, FernandoFernandez Navarro, JavierRodríguez-Rojo, Inmaculada ConcepciónFernandez Salinero, SamuelRodríguez-Rosell, DavidFernández Seguín, Lourdes MaríaRodríguez-Sánchez, José-LuisFernández Vázquez, EstebanRodríguez-Seijo, AndrésFernandez, AlbertoRodríguez-Tapia, LiliaFernández, EstrellaRodríguez-Ventura, AnaFernández, Francisco BrocalRoehl, WesleyFernández, José ÁngelRoenneberg, TillFernandez, KatharineRog, JoannaFernández, María Dolores RuizRogachev, Aleksey F.Fernandez, Marina O.Rogalewicz, VladimírFernández, SoledadRogerio Pinheiro, PlácidoFernández-Antelo, InmaculadaRogers, Shane L.Férnandez-Batanero, Jose M.Rogers, W. PrattFernández-Berrocal, PabloRoghanchi, PedramFernández-Bustos, Juan GregorioRogmark, CeciliaFernández-Castillo, AntonioRognli, Erling W.Fernández-de-las-Peñas, CesarRogozea, LilianaFernández-Díaz, ElenaRogoziński, TomaszFernández-Domínguez, Juan CarlosRogula-Kopiec, PatrycjaFernandez-Ferrer, AntonioRogula-Kozłowska, WiolettaFernández-Fuertes, Andrés A.Rogus, StephanieFernández-García, José CarlosRoh, ChanghyunFernández-Gómez, ElisabetRoh, TaewooFernández-Lázaro, DiegoRoh, Young SookFernandez-Luna, AlvaroRohaim, MohammedFernández-Luqueño, FabiánRohde, CarmenFernández-Martínez, AntonioRohde, KerstinFernández-Martínez, ElenaRohit, AthiraFernández-Martínez, EliaRohr, BettyFernández-Martínez, María Del MarRohr, Linda E.Fernández-Mateos, M. PilarRohrmann, SabineFernandez-Matias, RubenRój, JustynaFernández-Medina, Isabel MaríaRojas, EricFernández-Morilla, MonicaRojas, Mariana A.Fernández-Ozcorta, EduardoRojas-Rueda, DavidFernández-Parra, AntonioRojo Tirado, Miguel ÁngelFernández-Pires, PaulaRojo, Maria AngelesFernández-Prados, Juan SebastiánRokita-Poskart, DianaFernández-Revelles, Andrés B.Rolando, SaraFernández-Rouco, NoeliaRoldán Aliaga, AinoaFernández-Salinero, SamuelRolim Neto, Modesto LeiteFernández-Tena, AnaRoll, LaraFernandez-Veiga, ManuelRolla, GiovanniFernández-Villa, TaniaRollè, LucaFernando De Rezende, LuizRollnik-Sadowska, EwaFernando, BinoshaRoma, PaoloFernando, Eustace Y.Román López, PabloFeron, FransRoman, CelianFerracini, RiccardoRoman, MichałFerracuti, StefanoRoman, MonikaFerradás Canedo, María Del MarRoman, SoniaFerragina, EmanueleRomanelli, Massimiliano M. CorsiFerrández, DanielRomán-Graván, PedroFerrante, GiulianaRomani, AndreaFerranti, EmmaRomanić, Snježana HercegFerrara, BenedettaRomaniuk, PiotrFerrara, PietroRomano Spica, VincenzoFerrari, EmanueleRomano, DanielaFerrari, FedericoRomano, LucianoFerrari, ManuelaRomano, SalvatoreFerrario, Maria FrancescaRomanoff, AnyaFerraris, ClaudiaRomanov, VictorFerraro, AlbertoRomanuke, VadimFerraz Almeida, RiselyRomaszko, JerzyFerraz, Ricardo Manuel PiresRombaut, EvyFerreira Da Silva, Leonardo AragaoRomeo, KaterineFerreira Machado, José ManuelRomer, DanFerreira, Aline AlvesRomera, EvaFerreira, Ana RitaRomerio, FabioFerreira, Andrea C. F.Romero Collado, AngelFerreira, ArthurRomero De La Cruz, Elena RuízFerreira, DéboraRomero Martínez, ÁngelFerreira, Fernanda A.Romero Martínez, Sonia J.Ferreira, Hugo Enrique JúnezRomero Morales, CarlosFerreira, JenniferRomero Rodríguez, José MaríaFerreira, JorgeRomero, DiegoFerreira, Luís PintoRomero, Karla A.Ferreira, MarceloRomero, Laura LopezFerreira, Maria EduardaRomero, Laura Ponce De LeónFerreira, Maria Manuela Pires Sanches Romero, Rosenberg J.Ferreira, MartinsRomero-Ania, AlbertoFerreira-Coimbra, JoaoRomero-Ayuso, DulceFerreira-Oliveira, Ana TeresaRomero-Bejar, José LuisFerreira-Pêgo, CíntiaRomero-Blanco, CristinaFerreira-Santos, FernandoRomero-Galisteo, RitaFerreiro, Maria De FátimaRomero-Gómez, BenjamínFerrell, AnastasiyaRomero-Gonzalez, BorjaFerrer Peña, RaúlRomero-Rodríguez, Luis MiguelFerrer, Carolina PerezRomero-Romero, SoniaFerrer, Victoria A.Romero-Saldaña, ManuelFerrer-Sargues, Francisco JoséRomeyke, TobiasFerri, Giovanni MariaRominski, SarahFerrini, FrancescoRomiti, Giulio FrancescoFerro, AnaRommel, Anna-SophieFerro, DomenicoRomo Pérez, VicenteFerro, Giancarlo AntonioRonca, FlaminiaFerronato, NavarroRonconi, LuciaFervers, BéatriceRondinone, BrunaFeser, MarkusRondon, AurélieFestila, Dana GabrielaRonga, DomenicoFettermann, DiegoRoni, MonzurulFeu, SebastianRonnebaum, JulieFeucherolles, MaureenRonquillo Lomelí, GuillermoFialkowski, WojciechRonsard, LaranceFialova, JitkaRonto, RimanteFiamoncini, JarleiRonzi, SaraFiaschi, SimoneRöösli, MartinFiates, Giovanna MedeirosRoot, MartinFibert, PhilippaRoper, CourtneyFicapal-Cusí, PilarRoper, Randall J.Ficca, GianlucaRopero-Padilla, CarmenFichna, MartaRoque, FátimaFidaldo, AntonioRoque, VitorFidani, CristianoRoques, ChristianFiddler, Joanna L.Roques, Christian-FrançoisFields, BethRoques, LionelFiessler, CorneliaRoriz, PauloFietkiewicz, Kaja J.Ros, IkerFigueiral, Maria HelenaRosa Alcázar, ÁngelFigueiredo, Amélia SimõesRosa Garcia, AlfonsoFigueiredo, DanielaRosa, Bruce A.Figueras-Aloy, JosepRosa, DeboraFigueras-Alvarez, OscarRosa, Jesús De LaFigueroa Millán, Julio V.Rosa, Maria JoseFigueroa, Johnny D.Rosa, Olga María Alegre De LaFigueroa, RogerRosabal, MaikelFigueroa-Rodríguez, Katia A.Rosales-Mayor, EdmundoFilardi, TizianaRosaly, Correa-de-AraujoFilchev, LachezarRosana Rocha, BarrosFilgueira, DulceRosário Filho, Nelson AugustoFilgueiras, RobertoRosario, BarrancoFilipiak, KrzysztofRosario, Fredrick J.Filipiak, MichałRosario, MartinFilipiak, SaraRosário, PedroFilipovic, MiodragRosario-Rosado, RosaFilippini, TommasoRosas, Jose H. AblanedoFincham, AndrewRosato, Michael G.Fine, PeterRosborg, IngegerdFinell, EerikaRose, Christopher F.Finelli, CarmineRose, JeffFink, AstridRose, JennieFinkel, Alan G.Rose, Sarah E.Finkel, Madelon L.Rosebrock, Adam P.Finkielsztein, MariuszRoseiro, LuísFinlay, KatherineRosenbaum, HowardFinneran, KevinRosenberg, ElissaFinney, GailRosenthal, MeagenFinsterer, JosefRosenthal, Sonny BenFiorentino, MariaRosenthal-von Der Pütten, AstridFioretti, AlessandraRosenvinge, Jan H.Fiori, Anna MariaRosenwasser, LannyFiori, CristianRoser, KatharinaFiorillo, LucaRosi, EleonoraFiorini, Rodolfo A.Rosic, GvozdenFiorito, FrancescoRosken, AnneFirek, WieslawRos-McDonnell, LorenzoFirmino, HoracioRosofsky, AnnaFirst, JenniferRosołek, BarbaraFirstenberg, MichaelRosquete, DanielFischbach, AndreaRoss, AllisonFischer, AndrewRoss, IanFischer, FlorianRoss, KirstinFischer, KatjaRoss, MaryFischer, Lori A.Ross, RyanFischer, MatthiasRoss, Sharon E.Fischer, Nicholas G.Ross, Victoria L.Fischer, RenéRossa Jr., CarlosFischer, Saskia M.Ros-Santaella, José LuisFischer, TatjanaRossel, Pedro O.Fischetti, FrancescoRosset, PhilippeFisher, AbigailRossheim, Matthew E.Fisher, ChristopherRossi, AlbertoFisher, JamesRossi, AlessandroFistola, RomanoRossi, AlessioFitch, MargaretRossi, AmerigoFitchett, JenniferRossi, ClaudioFitzgerald, EdwardRossi, GiuseppeFitzsimmons, SuzanneRossi, LeucioFizaine, FlorianRossi, Maria GrazziFlack, WilliamRossi, NoreenFlaschberger, EdithRossini, GabrieleFlecha, RamonRossolini, MonicaFleischhacker, SheilaRossow, Lindy M.Fleisher, Mark S.Rost, GergelyFleming, CatharineRoster, CatherineFleming, Richard K.Rostoker, GuyFleszar, MariuszRosu, HaretFletcher, IainRoszak, JoannaFletcher, JaniceRoszak, PiotrFlexeder, ClaudiaRoth, AndreasFlood, MichelleRoth, RoswithFlood-Grady, ElizabethRothe, UlrikeFlorán, BenjamínRothenberg, RichardFlorek, EwaRotherham, Ian D.Flores, EmilyRothmore, PaulFlores-Aranda, JorgeRothschild, JeffreyFlores-Fraile, JavierRoth-Walter, FranziskaFlores-Guerrero, Jose L.Rotter, IwonaFlores-Quijano, Maria EugeniaRoubertoux, PierreFlores-Ramírez, RogelioRouch, AlexanderFlores-Ruiz, DavidRouco, José Carlos DiasFloria, MarianaRougeau, KathrynFlorijn, Barend W.Roulston, DallasFlor-Montalvo, Francisco J.Rourke, BrendaFloros, GeorgiosRousseau, RonaldFloyd, MyronRout, Simon P.Floyd, SarahRovas, AlexandrosFlynn, EmileeRovito, Michael J.Foels, LeonoraRowland, SheriFogacci, FedericaRowley, GeorgiaFogarty, TimothyRoy, BidishaFogel, StuartRoy, IndraniFogler, JasonRoy, Partho SarothiFoletto, MirtoRoy, RosaFoley, EdwardRoy, Saktimayee MitraFoligni, RobertaRoy, SomeshwarFolkvord, FransRoyapoor, MohammadFolsom, LisalRoyé, DominicFölster-Holst, ReginaRoy-Engel, AstridFolta, Timothy B.Royo, FelixFomba, Khanneh WadingaRoza, ThuaneFomenko, AntonRóżańska, AnnaFominiene, Vilija BiteRóżańska-Walędziak, Anna M.Fong, KelvinRóżański, ZenonFong, Lawrence Hoc NangRozario, PapiaFonseca, AnaRozek, DavidFonseca, PedroRozerta, SokouFonseca-Alves, Carlos EduardoRozgonjuk, DmitriFonseca-Montes De Oca, Reyna María Guadalupe Rożnowski, BohdanFonseca-Pedrero, EduardoRóżowicz, AntoniFonseca-Pinto, RuiRozpara, MichałFontana, LucaRóżycki, MirosławFontana, MarioRóżyło, KrzysztofFontarigo, Juan José NietoRozza, ArianeFonte, Carla Alexandra MartinsRuan, ZengliangFonte, PedroRuas, CassioFontiela, JoaoRuban, DmitryFonzo, MarcoRubasinghege, GayanFoong, Rachel E.Rubben, AlbertFoote, Janet A.Rubin, JosephFoppa, Ivo M.Rubio, Ana SotoForbes, LauraRubio, CarmenFord, DawnRubio, SusanaFord, Timothy E.Rubio-Arias, Jacobo Á.Foreman, Anne M.Rubio-Armendariz, CarmenForeman, Stephen E.Rubio-Arraez, S.Forget, PatriceRubio-Bellido, CarlosForján Castro, RubénRubio-Valdehita, SusanaForján, RubénRubira-García, RainerForlizzi, JodiRucci, PaolaFormankova, SylvieRucco, RosariaFormenti, DamianoRuch, WillibaldFormenti, FedericoRucińska, JoannaFormenton, GianniRucki, MiroslawFormichi, PaoloRudd, RobinFormiga, Márcio De CarvalhoRudin, DeborahFormisano, PietroRudnick, AbrahamFornalski, KrzysztofRudolph, Cort W.Fornara, RobertoRudolph, HeikeFornari, FabrizioRudolph, UweFornaro, AdalgizaRudrajit, MitraForsberg, AnnaRudrappa, MohanForsell, YvonneRudroff, ThorstenForst, LindaRuducha, JennyForster, JamesonRudziński, Krzysztof J.Forsum, Elisabet A.Ruebsam, WolfgangForte, AlbertoRueda, SilviaForte, GiuseppeRueff, JoséForte, Iris MariaRuegg, MarkusForte, MaurizioRuffino, BarbaraForte, PedroRugel, EmilyFortgang, RebeccaRugg, ChristopherFortner, RosanneRuggieri, Ruggero AndrisanoFortunato, AlexandroRuggiero, CarmelindaFortunato, LeonzioRugulies, ReinerFortune, NicolaRugytė, DanguolėForwood, SuzannaRuhle, SaschaFoschino-Barbaro, Maria PiaRuipérez-Valiente, José A.Foskett, AndrewRuisoto, PabloFoster, AlexanderRuivo Alves, Ana SofiaFoster, John H.Ruiz Chico, JoséFoster, JoshRuíz Palomino, PabloFoster, Kenneth R.Ruiz, Jesús FernándezFoster, Toshana LauriaRuiz, Manuel J.Fotheringham, StewartRuiz, María José Bautista-CerroFotiadis, DimitriosRuiz, MatthewFotis, TheoRuiz-Alvarez, OsiasFotouhi, OmidRuiz-Eugenio, LauraFouche, ChristaRuiz-Fernández, María DoloresFouladkhah, AliyarRuiz-Frutos, CarlosFoulds, JamesRuiz-García, VictorFoumani, MehdiRuiz-Litago, FátimaFountas, GrigoriosRuiz-Montero, Pedro JesúsFourali, Chahid E.Ruíz-Muñoz, MaríaFournier, AngelaRuiz-Padillo, AlejandroFoust, ReganRuiz-Palmero, JulioFoust, Richard D.Ruiz-Real, Jose LuisFoutch, Brian K.Ruiz-Robledillo, NicolásFowdar, HarshaRuiz-Rudolph, PabloFowler, LaurenRukambile, ElpidiusFowler-Davis, SallyRumney, PeterFowler-Watt, KarenRunco, MarkFox, AdamRunkle, JenniferFox, CarolRuotolo, FrancescoFox, DianaRuppert, ElisabethFox, Mary AvisRuppert, KristineFox, StephenRusch, LoriFraczyk, JustynaRuser, AlexanderFrade, FátimaRush, Elaine C.Frade, João Manuel GraçaRushton, KellyFraga, Mariana AmorimRussell, AlyceFraguas-Sánchez, Ana IsabelRussell, DavidFraiz Brea, José AntonioRussell, MarionFram, MaryahRussell, MarkFrame, Leigh A.Russell, RichardFranc, SanjaRussell, SimonFranca, Eduardo F.Russell, Steve T.Francesca, CalastriniRusso, AlessioFranceschini, GiordanoRusso, BenedettaFrancesco, AlettaRusso, CarloFrancesco, SmedileRusso, FelicitaFrancetich, JadeRusso, LucaFranch-Pardo, IvánRusso, MassimilianoFranch-Parella, JordiRustico, LorenzoFrancia, GuadalupeRusu, AdrianaFrancis, KarenRusu, Alina SimonaFrancis, LeslieRusu, DianaFrancis, Leslie J.Ruszkowska-Ciastek, BarbaraFrancis, LynRutberg, StinaFrancisci, SilviaRutigliano, FloraFrancisco, AlbornozRutkauskaite, RenataFrancisco, Javier Alvaro-AfonsoRutkauskas, SauliusFranco Álvarez, EveliaRutkowski, SebastianFranco Pozzolo, AlbertoRutteman, BartFranco, Camilo AndresRuzieh, MohammedFranco, ChristianRuzsics, IstvánFranco, MediciRyabov, IgorFranco-Paredes, CarlosRyan, AnthonyFraněk, MarekRyan, GregFrange, CristinaRyan, JillianFrank, Arthur L.Ryan, MichaelFrank, LuizaRyan, MichelleFranke, NikeRyan, SusanFrankel, TylerRybak, JustynaFranken, EsmeRybak, Leonard P.Franklin, NatashaRybak-Niedziolka, KingaFranklin, PeterRybka, JakubFrankowski, MarcinRybka, Jakub DaliborFranks, SusanRybska, ElizaFransen, KoosRybtsov, StanislavFranzè, AnnamariaRychtáriková, RenataFraser, BrodieRyder, SheilaFraser, DavidRyff, Carol D.Frasquilho, DianaRyffel, BernhardFrasso, RosemaryRymanov, AlexanderFrati, PaolaRymuza, KatarzynaFratini, FilippoRyskulova, AselFrau, Diego G.Ryter, KendalFrawley, PatsieRyu, Dong-HeeFrazer, KateRyu, EunjungFrazier, AnnabelleRyu, Jae-InFredianelli, LucaRyu, Kwang SunFredricks, Karla MarieRyu, Shin KueFreedman-Doan, CarolRzadkiewicz, MartaFreeman, BonnieRząsa, KrzysztofFreeman, EmilyRześny-Cieplińska, JagienkaFreeman, LauraRzońca, EwaFreeman, Michael D.Rzońca, PatrykFreeze, GregoryRzymowska, JolantaFrei, ThomasRzymski, PiotrFreiberg, AnnaSá, Luís Octávio DeFreiberger, EllenSaad, MarceloFreire, André PimentaSaarela, MirkaFreitas De Freitas, LucasSaarela, RiittaFreitas, AlbertoSaavedra, MiguelFreitas, RaquelSabato, GaetanoFreitas, RenataSabbatinelli, JacopoFreitas, TomásSabbatucci, MichelaFrennert, SusanneSabella, Kathryn AnnFresán, UjuéSabelnikova, NataliaFrese, VanessaSabi, Stella C.Freudenthaler, Heribert HaraldSabia, MichaelFreundt, Eric C.Sabina, CivilaFrias Casas, MarioSaboga Nunes, LuisFrías-Navarro, DoloresSaboya, Patricia RecioFrič, JanSaccenti, EdoardoFricker, Jon D.Sacchi, SimonaFricker, PiaSacco, PierFrideres, JamesSaccomanno, SabinaFriedman, CliveSachlas, AthanasiosFriedman, KeithSachs, MichaelFriedman, Paula K.Sacko, RyanFriedrich, PaolaSacramento, OctávioFriedrich, RainerSacrey, Lori-Ann R.Friedrich, TheodorSádaba, CharoFries, FabianSadanaga, TsuneakiFriesen, CarolSadasivam, MohanrajFrietze, SethSaddik, BasemaFriger, MichaelSadeghian, Ramin BananFriman, HenSadler, CharlotteFrisso, GiuliaSadłowska-Wrzesińska, JoannaFristedt, SofiSadok, IlonaFritschi, LinSadowski, JerzyFroelich, WojciechŠadzevičius, RaimondasFrohn, Lise M.Sæbø, GunnarFrohnhofen, HelmutSáenz-Lopez, PedroFrolova, AnnaSaez, MarcFrolova, Tatiana S.Sáez-Padilla, JesúsFronczyk, JoannaSafavi-Naeini, PayamFrone, Michael R.Saffache, PascalFronteira, InesSaffell, JohnFrontoni, EmanueleSagbo, JonasFrosi, AlbertoSagebiel, John C.Frost, JeremySager, ManfredFrost, PeterSagone, ElisabettaFrost, RachaelSaha, AuromeetFry, AndrewSaha, ProgyaparamitaFry, AndySaha, SanjibFryc, IrenaSaha, SanjoyFrye, BjörnSaha, SubrataFu, Chih-YuanSahajpal, RitvikFu, Chiung-ShiuanSahani, RajkumarFu, JackSahay, BikashFu, JingyingSahin, AtakanFu, MinSahlem, Gregory L.Fu, QiangSahlin, UllrikaFu, QianyiSahoo, SubhransuFu, ShengSaias, JoséFu, WeijieSaidi, NeilaFu, XianqiangSaín, JulianaFu, Xiao-AnSainani, KristinFu, Yin-ChihSaint-Amour, DavidFucà, RominaSaint-Fort, RogerFuchs, Judith A.Sainz De Abajo, BeatrizFuchs, Piotr A.Sainz, CarlosFuehrer, PaulSaisho, YoshifumiFuente-Merino, IsmaelSaito, JumpeiFuentes Cabrera, ArturoSaito, KanFuentes Pumarola, ConcepcióSaito, Tais BerelliFuentes, María C.Saiz Galdós, JesúsFuentes, PatriciaSáiz Manzanares, María ConsueloFuentes-Garcia, JuanSajewicz, EugeniuszFuentes-Rivas, Rosa MaríaSak, Jarosław J.Fuereder, ThorstenSakai, KotomiFuertes-Camacho, M. TeresaSakakibara, ChieFuertes-Guiró, FernandoSakakibara, IoriFugger, GernotSakakibara, MasayukiFührer, AmandSakamoto, MaikoFuhrmann, ChristopherSakarikou, ChristinaFuji, KevinSakata, MasahiroFujibe, FumiakiSakellari, EvanthiaFujii, HidekiSakellaris, IoannisFujimoto, HiroyukiSakharova, AntoninaFujimoto, NobukazuSakkopoulos, EvangelosFujimura, ShintaroSakoda, AkihiroFujioka, KazumichiSakuma, KunihiroFujioka-Kobayashi, MasakoSakuma, ShigemitsuFujita, KatsuhideSakurai, MasaruFujita, KoheiSala, RoserFujita, ToshiyukiSałabun, WojciechFujiu, MakotoSalahuddin, MelihaFukuda, AkiraSalahuddin, MohammadFukuda, YoshiharuSalahudeen, Mohammed S.Fukuhara, YasuyukiSalami, BukolaFukumoto, RuiSalami, FiroozFulford, Richard S.Salamon, Katherine S.Fuller, A. OvetaSalanţă, Liana ClaudiaFulton, BethSalas Herranz, JorgeFulton, LawrenceSalas, José BlancoFulvio, Re CecconiSalas, Lucas A.Fung Wai-tung, AdaSalas-Guzmán, NataliaFung, Sai FuSalata, StefanoFunk, RichardSalavera, CarlosFura, BarbaraSalawu, EmmanuelFuresi, RobertoSalazar, IvanFurey, SinéadSalazar, Leslie RamosFurlan, RaffaelloSalazar-Torres, Jose De JesusFurlanetto Pacheco, Ana BeatrizSalcedo, InmaculadaFurler, JohnSaldaña, Manuel RomeroFurler, Robert L.Saldaña-Márquez, HéctorFurlong, MelissaSaldanha Matos, JoséFurman, LydiaSaldarriaga-Noreña, HugoFurniss, DavidSaldiva, Silvia Regina Dias MediciFurtado, RicardoSaleem, NayyerFurukawa, HiroshiSalehi, MaryamFurukawa, ToshiyukiSalehi, SanaFurushima, DaisukeSales Ciges, AuxiliadoraFuruta, MichikoSales, DoraFusaro, MariaSalessi, SolanaFusaro, MartinaSalguero Del Valle, AlfonsoFusco, AndreaSalguero-Caparrós, FranciscoFusco, Francesco MariaSalhi, YahiaFusco, GiulioSalido Tercero, JesúsFusco, Rachel A.Saliev, TimurFuster, DanielSalim, HengkyG. Smithard, DavidSalin, KasperGaballah, AhmedSalinas Fernández, José AntonioGabbianelli, RositaSalinas, JenniferGabbidon, KemeshaSalinas-Rodríguez, EleazarGabhainn, Saoirse NicSalinet, JoaoGabl, RomanSalisbury, JosephGablonski, Thorsten-ChristianSaliu, FrancescoGaboury, IsabelleSall, Papa MalickGabriel, AuraSallah, KankoéGabriel, BrendanSalma, JordanaGabriel, Mark C.Salman, AhmadGabriel, RonaldoSalman, MootazGabrielli, JoySalmasi, Abdul MajeedGabryelska, AgataSalmerón, IvanGabryś, TomaszSalmon, MorganGać, PawełSalokhiddinov, AbdulkhakimGacek, MariaSalomone, FedericoGacesa, RankoSalustri, AndreaGaddam, Ravinder ReddySalustri, FrancescoGaddi, Antonio VittorinoSalvado, FranciscoGade, Inger LiseSalvado, VictoriaGadomski, Anne M.Salvador-Coloma, PabloGaffney, ChristopherSalvalai, GrazianoGagat, MaciejSalvati, LucaGagliardi, CristinaSalvati, MarcoGagné, IsaacSalvia, RosannaGagnon, Joseph CalvinSalzano, FrancescoGailite, JurgitaSalzano, GiovanniGaillard, DanySalzano, GiuseppinaGainsbury, SallySamaddar, SubhajyotiGaitán-Cepeda, Luis AlbertoSamadi, Sayyed AliGaitens, JoannaSamant, YogindraGaitonde, VishwanathSamanta, SaileshGajda, MaksymilianSambataro, SergioGajdács, MárióSambri, AndreaGajewska, DanutaSambruno, AlejandroGajewski, JanSamek, LucynaGajewski, Jerzy B.Samet, JonathanGala, Rikhav P.Sami, MojganGalagher, JenniferSamim, FiroozehGalán Díaz, Juan JoséSamkange-Zeeb, FlorenceGalan, DiegoSammarco, RosaGalanis, AlexisSammells, Clare A.Galanis, Athanasios T.Samoggia, AntonellaGalan-Lopez, PabloSamorinha, CatarinaGalati, FrancescoSampaio, FranciscoGałązkowski, RobertSampaio, Luciana Maria MalosáGaldiero, Maria RosariaSampaio, Ricardo Aurelio CarvalhoGaldino, Maria José QuinaSampson, NatalieGale, JonathanSamson, SureshGaleazzi, Gian MariaSamuel, JimGalelli, LucaSamuel, Preethy S.Galeoto, GiovanniSamukawa, MinaGalesic, MorenaSamul, JoannaGalfalvy, HangaSan Juan Serrano, FuencislaGaliano-Coronil, AraceliSan Juan, Alejandro F.Galiatsatos, PanagisSan Pedro Veledo, María BelénGalindo, NuriaSan Román Mata, SilviaGallagher, DavidSanches, SandraGallagher, PaulSánchez Carreira, María Del CarmenGallandat, KarinSánchez Castillo, SheilaGallar, DavidSánchez Fernández, María DoloresGallard, HervéSanchez Fuentes, SergioGallardo Vázquez, DoloresSánchez López, FernandoGallardo, Ana MaríaSánchez Loyo, Luis MiguelGallardo, Laura O.Sánchez Martín, MicaelaGallè, FrancescaSánchez Núñez, Christian AlexisGallego, M. DoloresSánchez Rojo, AlbertoGallego-Colon, Enrique J.Sánchez Sáez, Juan AntonioGallego-Jiménez, M. GloriaSánchez Sánchez, Laura Del CarmenGallegos-Arreola, Martha PatriciaSanchez Vadeón, LeticiaGallelli, VincenzoSanchez, Anthony M. J.Gallenti, GianluigiSánchez, DanielGalletti, FrancescoSanchez, David JesseGalli, GianfrancoSanchez, EduardoGalli, JacopoSanchez, Jorge R.Galli, MassimoSánchez, Laura E. GómezGalli, MatthewSánchez, PatricioGallicchio, GermanoSanchez, TiffanyGallina, SoniaSanchez-Azanza, Victor A.Gallo Cordova, AlvaroSánchez-Caballé, AnnaGallo, SimoneSánchez-Ferrer, Maria L.Gallotta, ValerioSánchez-Garcés, María ÁngelesGalmés, SebastiàSánchez-García, Juan CarlosGalmiche, FlorenceSánchez-Gómez, María BegoñaGalosi, AndreaSanchez-Gomez, MartinGalusha, Aubrey L.Sánchez-Gómez, RubénGalvan, CristinaSánchez-González, DiegoGalván, LauraSanchez-Gordon, SandraGalvani, EmersonSánchez-Hernández, M. IsabelGálvez, AlyshiaSánchez-Herrera, Ismael S.Gálvez, Ana Maria PorcelSánchez-Jiménez, VirginiaGálvez-Martín, PatriciaSánchez-Martín, María JesúsGamache, DominickSánchez-Miguel, Pedro AntonioGamal, DoniaSánchez-Moral, SimónGamber, MichelleSánchez-Núñez, M. TrinidadGamble, AmandineSánchez-Núñez, PabloGamble, Donald S.Sanchez-Oliva, DavidGamble, Jeffrey HughSanchez-Oliver, Antonio J.Gamet-Payrastre, LaurenceSanchez-Ortiz, JorgeGámez-Guadix, ManuelSánchez-Pay, AlejandroGamian-Wilk, MalgorzataSanchez-Perez, Maria Del MarGammon, CatherineSánchez-Rico, MarinaGamo, JavierSanchez-Rodas, DanielGamon, WojciechSánchez-Rodríguez, JoséGan, GuojunSánchez-Sánchez, BeatrizGan, LydiaSánchez-Sánchez, EduardoGan, PeihengSánchez-Sánchez, Laura C.Gan, VincentSánchez-SanSegundo, MiriamGAN, Wee HoeSánchez-Zafra, MaríaGandre, CoralieSanchis-Soler, GemaGanesan, Sukirth M.Sanders, ChrisGanini, CarloSanders, JamesGantner, MagdalenaSanders, SheilaGantz, Marie G.Sanders, TarenGao, CongSandholz, SimoneGao, JinSandle, TimGao, Jing-FengSando, EiichiroGao, JinlongSándor, JánosGao, LuSandoval-Herazo, Luis CarlosGao, QiSandoval-Palomares, José De JesúsGao, Sherry ShiqianSandy, Jonathan R.Gao, Shiqian SherrySandy-Hodgetts, KylieGao, TianSanfilippo, FilippoGao, XiaolongSanftenberg, LindaGao, XingSang, NeilGao, YanSang, ShengboGao, YangSangineto, MorisGao, YingSanguigni, ValerioGao, YuSani, AnaGao, ZanSanjeevaiah, AravindGao, ZhengSankararaman, SenthilkumarGarad, RhondaSanmartín, RicardoGaragiola, UmbertoSanmiquel Pera, LluisGaraicoa-Pazmino, CarlosSannella, AlessandraGaravello, MauroSannino, FilomenaGaray, UrtzaŠánová, PetraGarbayo, InésSansone, PasqualeGarbowska, MonikaSant’Ovaia, HelenaGarcez Sardo, Pedro MiguelSanta Cruz, IsabelGarcía Ael, CristinaSanta, FernandoGarcía Buades, María EstherSantabarbara, JavierGarcía Casarejos, María NievesSantacroce, LuigiGarcía De Alcaraz Serrano, AntonioSantalla, AlfredoGarcia De Avila, Marla AndréiaSantamaria, JoanGarcia De Lima, ElaineSantamaría-Peláez, MirianGarcía Del Castillo, AlvaroSantamera, AntonioGarcía Gómez, SalekySantana, AndrésGarcía Iglesias, DanielSantana, JefersonGarcia Iriarte, EdurneSantana-Mancilla, Pedro C.García Martín, Juan FranciscoSantana-Rodriguez, José JuanGarcía Medina, NagoreSantaniello, AntonioGarcía Navarro, Esperanza BegoñaSantarelli, AndreaGarcía Pelagio, Karla PaolaSantarsiero, GiuseppeGarcía Ramírez, ManuelSantasusagna Riu, AlbertGarcía Sánchez, Jesús NicasioSantella, Anthony J.Garcia- Vargas, MarielaSantesteban Echarri, OlgaGarcía Vázquez, Fernanda InézSanti, KristiGarcía Vidal, José AntonioSantiago Junior, Joel FerreiraGarcía, AlejandroSantiago, PauloGarcia, AmberSantilio, AngelaGarcía, CarmeloSantillan Garcia, AzucenaGarcia, EdeniseSantillán, Oscar S.García, Elena GervillaSantinha, GonçaloGarcia, Emilio GutierrezSantini, AntonelloGarcia, ErikaSantiso, Cristian FerreiroGarcia, Gabriel James M.Santoro, FrancescoGarcia, Jose Daniel JimenezSantoro, MattiaGarcia, Jose ManuelSantoro, Paolo EmilioGarcía, Juan C.Santos Júnior, Aníbal De FreitasGarcía, Marta Evelia AparicioSantos Silva, DoraGarcía, OscarSantos, AlexisGarcía, Patricia J.Santos, António DuarteGarcía, Raquel SebioSantos, Catarina C.Garcia, RitaSantos, ConceiçãoGarcía, TrinidadSantos, Débora Martins DosGarcia, ValerieSantos, Eva M.Garcia, VictorSantos, FernandoGarcía, Xavier GironésSantos, Juan M.García, YolandaSantos, Juliana De Melo Batista DosGarcia-Algar, OscarSantos, Luis y GangesGarcia-Bailo, BibianaSantos, Maria José De OliveiraGarcía-Cantó, EliseoSantos, Maria PaulaGarcía-Carrión, RocíoSantos, Mário J. D. S.García-Ceberino, Juan M.Santos, Max Mauro DiasGarcia-Constantino, MatiasSantos, PauloGarcia-de-la-Hera, ManoliSantos, RicardoGarcia-Descalzo, LauraSantos-Beneit, GloriaGarcía-Díaz, PilarSantos-García, GustavoGarcia-Fernandez, BertaSantos-Guzmán, JesúsGarcía-Fernández, Francisco PedroSantos-Olmo, Ana BelénGarcía-Fernández, JerónimoSantos-Reyes, JaimeGarcía-Galbis, Manuel ReigSantric Milicevic, MilenaGarcía-García, AgustínSantulli, GaetanoGarcia-Garcia, GuillermoSañudo, BorjaGarcía-García, OscarSanyal, UdishnuGarcía-Gasca, Silvia AlejandraSanyang, EdrisaGarcía-Gaytán, VíctorSanz, AlejandroGarcía-Germán, SolSanz-Calcedo, Justo GarciaGarcía-Gil, DiegoSanz-Cervera, PilarGarcía-Goñi, ManuelSanz-Nogués, ClaraGarcía-González, ÁngelaSanz-Paris, AlejandroGarcía-González, JessicaSanz-Rivas, DavidGarcía-González, LuisSapa, AgnieszkaGarcia-Gonzalez, Marialuz ArantzazuSapezinskiene, LaimaGarcía-González, VíctorSapkota, MuktaGarcía-Hermoso, AntonioSaponaro, FedericaGarcía-Herrero, SusanaSapountzaki, KalliopiGarcía-Iglesias, Juan JesúsSapp, Ryan M.García-Izquierdo, Antonio LeónSar, Bibhuti K.García-Jaén, MiguelSara, AgnaforsGarcía-Machado, Juan J.Sara, TomekGarcía-Malinis, Ana JuliaSarabia-Cobo, Carmen MaríaGarcía-Marín, RamónSarache, William A.García-Martínez, InmaculadaSaracini, ChiaraGarcía-Monge, AlfonsoSaraiva Valente, Ana MargaridaGarcía-Moreno, Francisco M.Saraiva, Erlandson FerreiraGarcía-Muñoz Rodrigo, FerminSaraiva, LindaGarcía-Ordás, María TeresaSaraiva, MiguelGarcía-Pérez, JavierSarapultsev, AlexeyGarcía-Pola, María JoséSarapultseva, MariaGarcía-Romero, JeronimoSarapura, SilviaGarcía-Sánchez, EstherSarasija, ShaarikaGarcía-Santos, DavidSaravanakumar, KandasamyGarcía-Segovia, PurificaciónSarche, MichelleGarcía-Soidán, Jose L.Sardella, AlbertoGarcia-Tobar, JavierSardi, AlbertoGarcía-Unanue, JorgeSardone, RodolfoGarcia-Villanueva, JorgeSarecka-Hujar, BeataGarcovich, DanieleSarfraz, MuddassarGardi, ConcettaSargentis, George-FivosGardner, LaurenSargisson, RebeccaGardner, Paul D.Sari, MiriamGaree, KhanSariaslan, AmirGareth, NyeSarid, OrlyGarfield, JoshuaSarkar, AmritaGarfield, SaraSarkar, MohamadiGarg, BhavukSarkar, Mrinal K.Garg, HarishSarkarai Nadar, VenkadeshGarg, HinaSarker, MarjanaGarg, KawishSarker, Md Nazirul IslamGaridkhuu, AriuntuulSarmati, LoredanaGarn, Joshua V.Sarmento, CaioGarnacho Castaño, Manuel V.Sarmento, HugoGarneli, VarvaraSarra, AnnalinaGarner, Bianca L.Sarría, IñigoGarnham, BridgetSarria-Santamera, AntonioGarnier, Yoann M.Sartin, EmmaGarrels, VeerleSartini, MarinaGarrett, Mario D.Sartore, GiovanniGarrido Cumbrera, MarcoSarullo, Filippo M.Garrido Velarde, JacintoSárváry, AttilaGarrido, Nuno DomingosSasagawa, MasaGarriga, RosaSasai, HiroyukiGarrote-Díaz, J. M.Sasaki, DaisukeGars, JohanSasaki, KensukeGarssen, BertSasamoto, NaokoGartenmann, StefanieSase, SunetraGartin, MeredithSasegbon, AyodeleGärtner, AnneSass, JenniferGartner, Lawrence M.Sasso, Ferdinando CarloGarus-Pakowska, AnnaŠatalić, ZvonimirGaruti, GiancarloSatchidanand, NikhilGarver, JenniferSatheesh, SathyanesonGary, FayeSathish, ThirunavukkarasuGarzillo, Elpidio MariaSatitsuksanoa, PattrapornGarzon, EduardoSato, EiichiGarzon, XimenaSato, HayatoGasana, JanvierSato, KeisakuGasco, GabrielSato, ShuzoGascon, SantiagoSato, TakuichiGash, HughSato, Tatiana De OliveiraGąsior, Jakub S.Satoh, MasahikoGąsior-Perczak, DanutaSatoh, YuhiGaskin, SharynSatorres, EncarnacionGaspar De Matos, MargaridaSattler, MatteoGaspari, JacopoSaturnino Lupi, MarcoGasparini, MauroSaubade, MathieuGasparro, RobertaSauceda, JohnGastaldi, LauraSauder, KatherineGaston, SymielleSauer, Pieter JjGates, WillSaul, DominikGato, EricSaulnier, RonGatti, Maria PaolaSaunders, Colleen JayneGatz, MatthiasSaunders, Daniel G.Gatzemeier, JenniferSaura Lacárcel, José RamónGau, Li-ShiueSaura, José RamónGaudin, ValérieSaurman, EmilyGauer, JuliaSaus, J. ArthurGaur, PramodSavassi Figueiredo, LucasGautam, MukeshSavolahti, MikkoGautam, SnehaSavolainen, IinaGautheron, JérémieSavtchenko, Andrey K.Gauthier, AnnieSawada, ShojiroGavala-González, JuanSawczuk, MarekGavini, MadhaviSawicka-Powierza, JolantaGavrilidis, Athanasios-AlexandruSawicki, WłodzimierzGavurová, BeátaSawrikar, PoojaGawel, DamianSaxe-Custack, AmyGawel, KingaSaxena, AksharGawlik-Dziki, UrszulaSaxena, SaurabhGawryszewska, Beata JoannaSaxena, VikasGazdag, GáborSayans, Mario PerezGazdecki, MichałSaydah, Sharon H.Gazerani, ParisaSayres, DavidGazzano, AngeloSaz, Sergio VillanuevaGazzaroli, DilettaSboev, AlexanderGazzola, PatriziaScacco, SalvatoreGC, Raj K.Scafetta, NicolaGe, CuiScallan, MartinaGe, ErjiaScambler, GrahamGe, Hong-HuaScandurra, AnnaGe, JiaojuScandurra, CristianoGe, WeiScarano, AntonioGe, WenzhenScardina, Giuseppe AlessandroGe, XinleiScarneo-Miller, SamanthaGe, YingchunScarpellini, EmidioGea-Caballero, VicenteScarselli, MarcoGebbie, KristineSchaarschmidt, SaraGebel, MichaelSchablon, AnjaGeberemariam, Thewodros K.Schade, Gunnar W.Gebska, MonikaSchade, HannahGee, Heon YungSchaefer, Sebastian D.Geetha, ThangiahSchaeffer, ElisaGefeller, OlafSchairer, CynthiaGehrke, Sérgio AlexandreSchalinskie, KevinGeisler, StephanSchank, ChristophGelado, RobertoScharf, ThomasGellert, George A.Scharoun Benson, SaraGelsomino, AntonioSchebesch-Ruf, WolfgangGelsomino, SandroScheck, AdrienneGemar, MasonScheeren, EduardoGenc, ElifScheffler, ChristianeGenco, Caroline AttardoScheid, Jennifer L.Genda, YujiScheidsteger, ThomasGendek-Kubiak, HannaScheinker, DavidGénéreux, MélissaSchelhas, JohnGeng, RunzheSchellhorn, Herb E.Gennari, Maria LuisaSchempp, Christoph M.Genovese, CristinaSchenk, AlexanderGenovese, FedericaSchenk, LindaGentil, PauloSchenkel, Lindsay S.Gentile, AmbraScherer, LexieGentile, EleonoraScherer, MarciaGeores, MarthaScherman, AshleyGeorg, RaphaelaSchermer, Julie AitkenGeorge, KoumantakisScherrer, JeffGeorge, StaceySchey, CarinaGeorgiadi, AnastasiaSchiappacasse, PaulinaGeorgian, BadicuSchiattarella, AntonioGeorgiev, TsvetoslavSchiavano, Giuditta FiorellaGeorgieva, SimonaSchiavo, SimoneGeraghty, PatrickSchiefermeier-Mach, NataliaGerasimova-Meigal, Liudmila I.Schieferstein, EvaGerbino, GiovanniSchierano, GianmarioGerbino, Maria GraziaSchierhout, GillGerdenitsch, CorneliaSchill, Anita L.Gerdin, GöranSchillo, BarbaraGerdol, RenatoSchils, TrudieGerhold, KerstinSchiltenwolf, MarcusGerkowicz, AgnieszkaSchindler, Charles W.Germana, AntoninoSchindler, MirjamGermano, AndresaSchippa, LeonardoGeronimus, ArlineSchlüer, Anna-BarbaraGervasi, MarcoSchlumbrecht, MatthewGesesew, Hailay AbrhaSchlundt, JoergenGettel, CameronSchlüter, NadineGeukes, CorneliaSchmalwieser, Alois W.Ghadieh, Hilda E.Schmeltz, Michael T.Ghafouri-Azar, MonaSchmerer, Hans-JörgGhag, GauravSchmid, ManuelGhandour, RajabSchmidl, DoreenGhani, ArfanSchmidt, AndrewGharehgozli, OrkidehSchmidt, Elizabeth K.Ghassib, IyaSchmidt, MarkGhaziri, Mazen ElSchmidt, ThomasGhigliotti, GiorgioSchmidt-Westhausen, Andrea-MariaGhilain, NicolasSchmitz, FlorianGhilli, MatteoSchmitz, GerdGhimire, Keshar ManiSchmoch, UlrichGhimire, Pragya SharmaSchmoeckel, JulianGhimire, UttamSchmutte, TimothyGhisi, Nédia De CastilhosSchnaubelt, SebastianGhita, MihaelaSchneeweis, AdinaGhobadi, MohsenSchneider, BarbaraGhomian, TaherSchneider, DorotheeGhoreishi, Seyede FatemehSchneider, FriedemannGhosal, SambuddhaSchneider, MargieGhosh, AbhijitSchneider, PetraGhosh, ArunavaSchneider-Kamp, AnnaGhosh, MonojSchneider-Muntau, BarbaraGhosh, RajeshwarySchnell, IzhakGhosh, SampatSchneller, Liane M.Ghosh, SayanSchnettler, BertaGhosh, SubhomoySchoenberg, FredericGhosh, SumitSchoene, MatthewGiaccone, ValerioSchoepp-Cothenet, BarbaraGiacomelli, AndreaSchöffl, VolkerGiacomelli, LucaSchofield, CathyGiacomello, EmilianaSchofield, PatriciaGiacometti, AndreaSchokkaert, ErikGiacomini, RenatoSchöllhorn, Wolfgang I.Giacomozzi, ClaudiaSchols, JosGiamberardino, Maria AdeleScholten, HannekeGiancotti, MonicaScholtmeijer, KarinGianfagna, FrancescoSchomburg, LutzGianfredi, VincenzaSchoppek, WolfgangGiangrande, AdrianaSchor, AyeletGiannantoni, AntonellaSchotthoefer, Anna M.Giannattasio, CristinaSchoua-Glusberg, AlisuGiannella, LucaSchouten, RonaldGiannì, Aldo BrunoSchreiber, KarenGianni, Maria LorellaSchreiber, MichaelGiannoni, MargheritaSchreinemachers, DinaGianola, SilviaSchreuders, MichaelGiantsis, IoannisSchröder-Butterfill, ElisabethGianturco, LuigiSchroeder, KristaGiardino, John RickSchubert, Anna-LenaGiatropoulos, Athanassios K.Schueftan Hochstetter, AlejandraGibbons, JeffSchuengel, CarloGibbons, JosephSchuette, NorbertGibney, NoelSchulte, AndreasGibson, Ann L.Schulte, KimGibson, HazelSchulte, PaulGibson, RachelSchultz, RosalieGibson, RosieSchulz, AmyGibson-Miller, JillySchulz, BurkhardGibuła-Tarłowska, EwaSchulz, ChristopherGichenje, HeleneSchulze, Peter S. C.Giddon, Donald B.Schulz-Weidner, NellyGierczak, TomaszSchumacher, ConnieGierczyk, MarcinSchumacher, JensGierlak, PiotrSchumann, BarbaraGiersch, GabrielleSchumm, Walter R.Gieryńska, MałgorzataSchutte, StaceyGiessing, LauraSchütz, Luís FernandoGigante, Giovanni E.Schuurman, Henk-JanGiger, Jean - ChristopheSchwab, CharlesGigovic, LjubomirSchwab, Daniel B.Gijón-Nogueron, GabrielSchwab, Stephen D.Gijs, MarliesSchwamberger, BenGíl Ares, JavierSchwandt, Hilary M.Gil Madrona, PedroSchwartz, BrianGil, Alfonso J.Schwartz, GaryGil, ArtyomSchwartzkopf-Phifer, KKateGil, DavidSchwarz, ElenaGil, Jorge MartínezSchwarz, RolfGil, Mathew ThomasSchwarze, MichaelGilbert, AlanSchwebel, DavidGilbert, ClareSchwierzeck, VeraGil-Calvo, MarinaSchwindenhammer, SandraGilchrist, Philippe T.Sciascia, SavinoGilden, RobynSciaudone, ElizabethGilewski, Paweł GrzegorzŚcibor, MonikaGil-Gimeno, JavierScifo, LidiaGilkey, DavidSciomer, SusannaGill, HarsharnScior, KatrinaGill, SamScocco, PaolaGill, SimoneScofield, R. PaulGill, Stephen D.Scopa, ChiaraGill, TiffanyScorziello, AntonellaGil-Lacruz, MartaScotney, RebekahGillespie, AvrumScott, JoAnna M.Gillespie, JudyScott, KelliGillies, RobynScotti, MarcusGillis, DorisScraba, Paula J.Gil-Lopez, Alfonso J.Scully, Sean MichaelGil-Martínez, AlfonsoScurati, RaffaeleGilmour, StuartScuri, StefaniaGilroy, RoseScuteri, AngeloGil-Salmerón, AlejandroSdino, LeopoldoGimenez, DanielaSdona, EmmanouelaGiménez, Jesus VicenteSeabi, JosephGimeno-Miguel, AntonioSeabra, FilipaGinevičienė, ValentinaSeabra, Larissa Mont’Alverne JucáGingras, VeroniqueSeabra, Paulo Rosário CarvalhoGinja, SamuelSeals, Brenda F.Ginley, Meredith K.Sealy, JoshuaGiontella, AliceSears, Clara G.Giordano, RaffaeleSebastiani, GiorgiaGiorgi Rossi, PaoloSecanilla Campo, Esther MariaGiorgi, EmanueleSeco-Calvo, JesusGiouri, KaterinaSecondi, LucaGiovagnoli, RaffaelaSecondo, LynnGiovanardi, GuidoSederevičiūtė-Pačiauskienė, ŽivilėGirard, BeverlySedghizadeh, ParishGirardi, DamianoSediqi, Mohammad MasihGirardi, PaoloSediqzadah, SaadiaGiraudeau, NicolasSeear, Kimberley H.Girault, FrédéricSeed, SheilaGiri, HemantSeeherunvong, TossapornGirolami, FlaviaSeeman, Mary V.GIROUD, MauriceSeewaldt, Victoria L.Giske, RuneSefat, FarshidGislason, MayaSegal, NicolasGitirana, MarceloSegal, UmaGitlow, LynnSegal-Engelchin, DoritGiudetti, AnnaSegarra, Ana BelénGiudice, AmerigoSeghers, JanGiudice, Giuseppe LoSegura Robles, AdrianGiudicelli, Bruno B.Segura-Garcia, JaumeGiudici, Kelly VirecoulonSegura-Ortí, EvaGiuffrè, MauroSegura-Robles, AdriánGiuliani, AngelicaSehgal, RahulGiuliani, FrancescaSeibert, Franz JosefGiulio, NittariŠeibokaitė, LauraGiummarra, MelitaSeidel, David H.Giungato, PasqualeSeidl, MaximilianGius, Mark PaulSeidler, AndreasGiuseppe, Dario DiSeiler, RogerGiuseppe, VarvaraSeiner, Brienne N.Giustetto, AmbraSeingier, GeorgesGiusti, RaffaeleSeitz, ChristopherGjelstrup Bredahl, Thomas ViskumSeixas, AdéritoGkillas, KonstantinosSekendiz, BetulGkiouleka, AnnaSekh, Arif AhmedGkisakis, VasileiosSekine, YoshikaGkrilias, PanagiotisSekulic, DamirGłąbska, DominikaSelfridge, MarionGlade, Michael J.Selland, CoreyGladysheva, Inna P.Sellars, MauraGladysz, BartlomiejSellers, KristinGlasgow, TrevinSellitto, Miguel AfonsoGlass Lewis, MarquisetteSelvakumar, AriamalarGlass, SteveSelvaraju, VaithinathanGlässel, AndreaSelvaratnam, ThineshGlatt, RubenSelvey, LindaGlaus, JenniferSelyanchyn, RomanGlavee-Geo, RichardSembajwe, GraceGleeson, NigelSemmens, ErinGlenn, SheilaSemu, Linda L.Glickman, EllenSemwal, MonikaGlicksman, AllenSenathirajah, YaliniGliniak, MaciejSenbonmatsu, TakaakiGlinkowski, WojciechSenchina, DavidGlińska-Neweś, AldonaSenefeld, JonathonGliozzi, MicaelaSenes, GiulioGlockner, KarlSenesi, PamelaGlogauer, MichaelSenetra, AdamGloghini, AnnunziataSenft, NicoleGloria, Alberta M.Sengoelge, MathildeGlowniak, AndrzejSengoku, ShintaroGlushakov, AlexanderSengupta, ArjunGłuszak, MichałSengupta, BidishaGlynn, Peter J.Sengupta, JaydeepGmurek, MartaSenhadji-Navarro, RaoufGnat, SebastianSenin-Calderon, CristinaGnoni, Maria GraziaSennehed, Charlotte PostGnudi, SaverioSennes, Luiz UbirajaraGobba, Fabrizio MariaSenoner, ThomasGobbi, EricaSentana, IreneGobbo, StefanoSentandreu-Mañó, TrinidadGobo, João Paulo AssisSentell, TetineGocheva-Ilieva, Snezhana GeorgievaSeo, Dae YunGochyyev, PermanSeo, HeewonGodderis, LodeSeo, SongwonGodin, JeffreySeo, YongsukGodina, RaduSeok, HyunGodleski, JohnSeol, JaehoonGodlewska, JoannaSepasgozar, SamadGodlewska-Żyłkiewicz, BeataSepelevs, IgorGodoy-Cumillaf, AndrésSeposo, Xerxes TesoroGodoy-Izquierdo, DéboraSequeira, CarlosGoeckenjan, MarenSequeira, João SilvaGoeden, HelenSequí-Canet, José MiguelGoehring, TobiasSera, FrancescoGoeijenbier, MarcoSerafini, GianlucaGoel, KanikaȘerban, OvidiuGoel, RahulSerbim, Andreivna KharenineGoethals, PeterSerbu, RazvanGoffredo, MichelaSéré, GeoffroyGogina, MayyaSergunin, AlexanderGoguikian Ratcliff, BettySerov, PavelGogulamudi, Venkateswara ReddySerpa, SandroGoh, Tan LengSerpanos, YulaGoig Martínez, Rosa MaríaSerrà, AlbertGoins, JustinSerra, EmiliaGois, Pedro FrancaSerra, LídiaGokita, TonyaSerra, Maria PinaGola, DamianSerra, PamelaGola, MarcoSerra, SergioGolabi, MohammadSerra-Majem, LluisGolas, ArturSerrancolí, GilGolcher, FelixSerranheira, FlorentinoGoldberg, DavidSerrano Lara, José JavierGoldmann, DanielSerrano Rosa, Miguel AngelGoldstein, Adam O.Serrano, JoãoGoldstein, NealSerrano, LuisGoldstein, OlzanSerrano-Muñoz, DiegoGoldstein, Rise B.Serrano-Pertierra, EstherGoldstein, SusanSerra-Paya, NoemiGoldthorpe, JoannaServais, VéroniqueGoleman, MałgorzataServidio, RoccoGolestaneh, LadanServidio, Rocco CarmineGolla, VijayServín-Garcidueñas, Luis EduardoGollop, MeganSessa, AurelioGollub, ElizabethSessa, FrancescoGolmohamadi, AmirSestraș, PaulGolmohamadi, HessamSeth, SohanGolomazou, EleniSethares, KristenGolston, Levi M.Sethi, GautamGolubkina, Nadezhda A.Sethi, Jaskaran SinghGomes Da Silva, DiogoSeto, ShigekiGomes, Cid André Fidelis De PaulaSettembre Blundo, DavideGomes, Daniela LopesSetzer, William N.Gomes, José OrlandoSeubert, Christoph N.Gomes, OrlandoSever, TinaGomes, Pedro GilbertoSeverini, GiacomoGomes, Thayse NatachaSeverino, PaoloGómez Barreto, Isabel MaríaSevil, JavierGómez Carrasco, Cosme JesúsSevilla-Morán, BeatrizGómez Ciriano, Emilio JoséSevil-Serrano, JavierGomez Escalada, MargaritaSewe, Maquines OdhiamboGómez Galán, JoséSeydel, KarlGómez Piqueras, PedroSeyedi, MirHojjatGomez Rodriguez, AlmaSeyedi, MohammadRezaGómez Salgado, JuanSeyednasrollah, BijanGomez, CamiloSeymour, KateGómez, Carmen VizosoSeymour, ValentineGómez, M. Pilar MunueraSezenna, ElenaGómez, MarioSezgin, EmreGómez, Mervyn MárquezSgarzani, RossellaGómez, MontserratSgolastra, FabrizioGómez-Abajo, PabloSgourou, EvaGómez-Armesto, AntíaSha, ZongyaoGómez-Baya, DiegoShabnam, MominGómez-Berrocal, CarmenShach-Pinsly, DalitGómez-Carmona, Carlos DavidShadgan, BabakGómez-Corona, CarlosShadrina, ElenaGomez-Crisostomo, RocioShafiei Shiva, JavadGómez-de-Terreros-Guardiola, MontserratShafiq, SarfrazGómez-Espinosa, AlfonsoShafique, MuhammadGómez-Galán, JoséShafray, EkaterinaGómez-Galán, MartaShah, Adnan RaheelGómez-López, ManuelShah, Birju A.Gómez-López, MercedesShah, GulzarGómez-Mármol, AlbertoShah, Mohammad Aminur RahmanGómez-Martín, BeatrizShah, Rachana D.Gomez-Ortiz, OlgaShah, SarwarGomez-Pastrana, DavidShah, ShaliniGomez-Pujol, LluisShah, Syed Asad AliGómez-Quintero, Juan DavidShah, Syyed Adnan RaheelGómez-Ruano, Miguel ÁngelShah, Vrutangkumar V.Gómez-Salgado, JuanShahab, SinaGomez-Silva, BenitoShahar, DanitGómez-Soberón, Jose ManuelShahariar, ShayebGómez-Tafalla, AnaShaheen, SusanGómez-Trigueros, Isabel MaríaShahian Jahromi, BabakGómez-Urquiza, Jose L.Shahid, ImranGommes, ReneShahid, RizwanGomula, AleksandraShahid, ShaouliGonçalves Barros, RosanaShahmoradi, MahdiGonçalves, BrunoShaikh, ZaffarGonçalves, Carlos Eduardo BarroShaker, Mohammed R.Gonçalves, Daniela CaetanoShakeshaft, CharolGonçalves, JorgeShakya, Kabindra ManGonçalves, Ricardo RebeloShalaby, Mohammed NaderGonçalves, Rui AbrunhosaShalaby, RehamGonçalves, VítorShalfawi, Shaher A. I.Goncalves, Widjane Sheila FerreiraShamionov, Rail M.Goncharuk, AnatoliyShamputa, Isdore CholaGong, AduShan, BerginGong, BingShan, HongmingGong, Cathy HongeShan, LingpengGong, JackShan, MingGong, Shi-weiShan, Xiaojun (Gene)Gong, WenjieShandilya, Kaushik K.Gong, YichengShandro, JanisGong, YunhuaShang, QingsenGoniewicz, KrzysztofShangguan, SiyiGoniewicz, MariuszShangguan, ZihengGonjo, TomohiroShankar, EswarGonzales, Mitzi M.Shankar, KripaGonzalez Calatayud, VictorShankland, RebeccaGonzález De La Roz, AlbaShanmugam, BharanidharanGonzález García, MarianoShanmugam, Shankar GanapathiGonzález Hernández, JuanShannon, HarrisGonzález Laredo, Rubén F.Shao, ChaofengGonzález Ramos, Ana M.Shao, TianjieGonzález Valeiro, MiguelShao, YongzhaoGonzález Valero, GabrielShapira, StavGonzález, CarinaShapiro, RonGonzález, DanielSharafutdinov, IrshadGonzález, David AlonsoSharareh, NasserGonzalez, GabrielSharifan, HamidrezaGonzález, JerónimoSharkey, MartinGonzalez, Raquel LuengoSharma, Adhikarimayum LakhikumarGonzalez, RogelioSharma, AjayGonzález, RubénSharma, AkshayGonzález, SoniaSharma, AnamikaGonzález-Aleu, FernandoSharma, ArvindGonzalez-Alonso, FernandoSharma, DileepGonzález-Aragón Pineda, Álvaro EdgarSharma, GeetanjaliGonzález-Badillo, Juan JoséSharma, HarmandeepGónzalez-Bandala, DanielSharma, Isha (Canada)Gonzalez-Barcala, Francisco-JavierSharma, Isha (USA)Gonzalez-Brown, Veronica M.Sharma, KavitaGonzalez-Caballero, Juan LuisSharma, ManojGonzález-Cabrera, JoaquínSharma, Priyanka (Spain)González-Calvo, GustavoSharma, Priyanka (USA)Gonzalez-Carrasco, MonicaSharma, RahulGonzález-Castro, Jose LuisSharma, RajanGonzález-Chordá, Victor M.Sharma, RishiGonzález-de Paz, LuisSharma, VikasGonzález-Díaz, CristinaSharmin, SoniaGonzález-Gálvez, NoeliaSharon, DeniseGonzález-García, AlbertoSharp, CatherineGonzález-García, HiginioSharp, MaryGonzalez-Garcia, Jose AngelSharwood, LisaGonzález-García, Rómulo JacoboShattuck, Eric ChristopherGonzález-Gómez, KeilaShaw, HeatherGonzalez-Jurado, Jose AntonioShaw, IanGonzález-López, José RafaelShaw, LisaGonzalez-Martin, CristinaShaw, PeteGonzalez-Medina, GloriaShaw, RajibGonzalez-Mendez, RosauraShaw, William S.González-Mesa, CarmenShe, JiangfengGonzález-Navarro, PilarShea, ChristineGonzalez-Ochoa, GuadalupeShea, Jennifer M.González-Pérez, Daniel M.Shea, Patrick J.González-Ramírez, Mónica TeresaShearer, Aiden EliotGonzález-Rivera, Juan AníbalShearston, Jenni A.Gonzalez-Rodriguez, AlexandreShebl, NadaGonzalez-Rubio, JesusSheehan, Mary C.González-Víllora, SixtoSheehan, Patrick J.González-Zamar, Mariana-DanielaSheehy, Lisa MaryGonzalo-Encabo, PaolaSheen, David A.Goodall, NoahSheen, FlorenceGoodchild, BarrySheffer, Christine E.Goodla, LavanyaShehata, NaderGoodman, Alan G.Shei, Ren-JayGoodman, Allen C.Sheikh, ZubedaGoodman, CliffordSheikhattari, PayamGoodman, Fallon RachaelShelake, Rahul MahadevGoodman, Lauren F.Shelite, Thomas RobertGoodman, PatShelley-Tremblay, JohnGoodrich, ErinShemmell, JonathanGoodworth, AdamShen, ChongyangGoolaup, SandhiyaShen, GuipingGoon, DanielShen, GuofangGopalakrishnan, VarshaShen, JunGopalan, Rajendran C.Shen, KaiGopisetti, Leela Sai PraveenShen, Kao-YiGoraj, WeronikaShen, LimingGordijn, MarijkeShen, LiningGordillo León, FernandoShen, Ming-YiGordillo, AldoShen, RuiqingGordish-Dressman, HeatherShen, ShiGordon, AdamShen, XiaoyunGordon, BarbaraShen, YangGordon, Eliza L.Shen, You-ChengGoretti, EnzoShen, YudongGorgey, Ashraf S.Shen, ZhengGorgoglione, AngelaSheng, PengfeiGori, AntonellaSheng, YanwenGori, DavideShenoi, Asha N.Gorini Da Veiga, AnaShenoy, AkhilGorini, FrancescaShepherd, AndrewGöritz, AnjaShepherd, Jennifer H.Görlich, DennisSheppard, Lorraine A.Gorman, RichardSheppard, PaulaGorman, ShelleySherchan, SamendraGörner, KarolSheridan, Robert P.Górnicka, MagdalenaSherk, AdamGórnicki, KrzysztofSherman, PaulGórnik-Durose, Małgorzata E.Shertzer, Howard G.Gorres, Josef H.Shestopalov, Alexander M.Górska-Warsewicz, HannaShete, VarshaGórski, MarcinSheu, Guang YihGosav, Evelina MariaShi, AiqinGoschin, ZiziShi, HaifeiGospodarek, JaninaShi, Han-pingGoswami, AnweshaShi, Hon-YiGoswami, NanduShi, JieGoto, TakaharuShi, JunjieGotta, VerenaShi, KaifangGötz, ThomasShi, LuGoua, MarieShi, Shi-MengGouandjika-Vasilache, IonelaShi, TiemaoGoudarzi, HoumanShi, VictorGoudas, MariosShi, YijunGoulas, AntonisShiao, Young-JiGoulet, DenisShiau, StephanieGoulet, EricShibanuma, AkiraGouveia, Élvio Rúbio QuintalShibata, NaokiGouveia, SoniaShibata, SeijiGovoni, MarcoShidal, ChrisGowda, Siddabasave B.Shield, JennyGowran, RosemaryShigemura, JunGozalo, Guillermo ReyShigetomi, YosukeGoździewska-Harłajczuk, KarolinaShih, David Ching-FangGozzoli, CaterinaShih, Dong-HerGrabarek, Beniamin OskarShih, Meng Ru (Lily)Grabarek, Joanna B.Shih, PattiGrabner, Daniel S.Shih, Tzu-ChingGrabovac, IgorShim, Jae-MahnGrabska, EwaShim, Joon W.Grabski, MeryemShimabukuro, YosioGraça, AmandioShimazu, AkihitoGraça, Flávia AparecidaShimazu, HarukiGrace Chang, KaowenShimba, ShigekiGraci, ValentinaShimek, JoannaGraco, MarnieShimizu, MitsuruGraczyk, DariuszShimomura, TakumiGradalski, TomaszShimura, AkiyoshiGradišek, AntonShin, Dong-chunGradissimo, AnaShin, DongheeGrady, AliceShin, Hyeong-MooGrady, Sue C.Shin, Hyoung ChulGradziuk, PiotrShin, HyungdeokGraetzer, SimoneShin, Hyun-SangGraf, LukasShin, Jae-lkGraff, MartinShin, Jong CheolGrafova, IrinaShin, SooimGragg, Sara E.Shin, Sun MiGraham, JamesShin, Won-SopGraham, JeffreyShin, Young JooGraham, MeganShin, YoungJuGraham, Tammy J.Shinan-Altman, ShiriGraham, TiffanyShiner, Christine T.Grajdian, MariaShingu, YasushigeGramatica, AndreaShinmura, FumitoGramatica, PaolaShintel, HadasGranata, FrancescoShiose, KeisukeGranatstein, DavidShipman, KateGrand, AnnShir, YoramGrande, FrancescoShirai, JyunsukeGrande, RossellaShirin, SoniaGrande-de-Prado, MarioShivanna, VinayGranero-Gallegos, AntonioShiwaku, HirokiGranero-Molina, JoseShmarakov, IgorGranic, AntonetaShoaf, KimGrant, AirdreShoda, MakotoGrant, Gerald T.Shohet, IgalGrant, WilliamShomura, MasakoGrao-Cruces, AlbertoShon, Ho SunGrappasonni, IolandaShook, AllanGrapsa, EiriniShopova, DobromiraGrare, MarionShort, LeonieGras, L. (Lapo) MughiniShou, YongyiGrassia, VincenzoShowell, ChrisGrassman, JeanShrestha, ManiGrasso, Domenico LeonardoShrestha, NamitaGråstén, ArtoShrestha, PranitaGrattan, Lynn M.Shrestha, Prateek M.Gratz, KlausShridhar, SurekhaGravelle, FrançoisShriver, LenkaGraveto, João Manuel Garcia Do Shu, FeiGravili, GinevraShubair, TamerGrawish, MohammedShuhan, HeGray, BenShukla, ArvindGray, CorieShukla, SiddharthGray, DeborahShuPing, WanGray, Janet M.Shuval, KeremGray, KathleenShuvo, Faysal KabirGray, Peter B.Sial, Muhammad SafdarGray, SelenaSibanda, MelusiGrayling, Michael J.Sibson, David RossGray-Miceli, DeannaSicard, PierreGrazer, JacobSicari, RosaGrazieschi, GianlucaSichero, LauraGrazioli, ElisaSicking, Dean L.Grebenisan, GavrilSidali, Katia LauraGreblikaite, JolitaSiddharth, SumitGrechi, DanieleSiddiqui, MuhammadGreco, Gian MariaSidik, FridaGreco, GianpieroSidiropoulos, AntonisGreco, LuigiSidiropoulos, KonstantinosGreco, Pablo JuanSidorczuk-Pietraszko, EdytaGrecu, EugeniaSidorkiewicz, IwonaGreen, JaniceSiebenmann, ChristophGreen, Jeffrey D.Sieber, FritzGreen, KenSiebers, RobGreen, KristenSiebielec, SylwiaGreen, MichaelSiedlecki, KarenGreen, OhadSiedlik, Jacob A.Green, RogerSiegrist, JohannesGreenberg, JeffSiemianowski, OskarGreenberg, JonathanSiemienska, RenataGreene, Catherine M.Sieni, ElisabettaGreene, Geoffrey W.Sierra, Javier OrtuñoGreene, JessicaSierra, Juan CarlosGreenfield, ThomasSierra, LeonardoGreenhalgh, PaulSierra-Campos, ErickGreenleaf, ChristySiessere, SelmaGreenman, JohnSignorelli, CarloGreenwald, RobySignorino, GuidoGreer, Frank R.Sigurvinsdottir, Rannveig S.Grego-Planer, DorotaSihombing, Sabrina OktoriaGregor, MilosSikder, Sujit KumarGregorio, JoaoSik-Lányi, CecíliaGregório, JoãoSikorska, DariaGregoris, ElenaSikorska-Zimny, Kalina MajaGregory, JohnSikorski, PiotrGregoski, Mathew J.Sikorsky, MarekGreig, AlisonSila, SaraGreim, HelmutSila-Nowicka, KatarzynaGreinert, AndrzejSilberberg, MinaGremigni, PaolaŠileikienė, RimaGrennan, TroySiles-Gonzalez, JoseGrešner, PeterŠilha, DavidGressier, FlorenceSilibello, CamilloGreviskes, LindseyŠiljeg, SilvijaGrewal, Raji PaulSilke, BernardGrieco, Julie A.Sillerio Quintana, ManuelGrieger, Jessica A.Sillero, AmaliaGriffin, P. BionSiltz, Lauren A.Griffin, PhoebeSilva Filho, EdsonGriffiths, DavidSilva, AbigailGrigorenko, Elena L.Silva, Adriana RibeiroGrigorescu, Elena-DanielaSilva, Ana FilipaGrigoriev, AndreiSilva, Anabela G.Grigorieva, ElenaSilva, BrunoGrigoryev, DmitrySilva, Carla MatosGrigsby-Toussaint, DianaSilva, CatarinaGrijalvo, SantiagoSilva, Danilo Rodrigues Pereira DaGrijota, FranciscoSilva, Diego Marconi Pinheiro FerreiraGrim, KatarinaSilva, Elisabete P.Grimaccia, ElenaSilva, EmilianaGrimaldi-Puyana, MoisesSilva, FátimaGrimholt, Tine K.Silva, Filipe MonteiroGrimm, Wolf-DieterSilva, Gabriela VenturaGrinberg, KerenSilva, GiovaniGrinberg, MariannaSilva, JacquelineGrincheva, NataliaSilva, JoséGrinevsky, Sergey O.Silva, Katia ReginaGrinholc, MariuszSilva, Kelly SamaraGroenendaal, MariSilva, Luiz Eduardo Virgilio DaGroffik, DorotaSilva, ManuelaGromadzinska, JolantaSilva, MarianneGromek, PawelSilva, Marta Angélica IossiGromisz, WilhelmSilva, MihiriGromkowska-Melosik, AgnieszkaSilva, PaulaGronewold, JanineSilva, PedroGrongstad, AnitaSilva, Roberto NascimentoGrosicki, GregSilva, RuiGross, BarbaraSilva, SamuelGross, MartaSilva, SusanaGrossman, Jennifer M.Silva-Afonso, ArmandoGrosso, GiuseppeSilvaggi, FabiolaGrove, Erik L.Silván-Ferrero, PradoGrowe, RoslinSilver, TobinGrubić Kezele, TanjaSilverstein, MadisonGruijters, Rob J.Silvestre-Rangil, JavierGrumi, SerenaSilvestri, DanieleGrüngreiff, KurtSilvia, PaulGrunig, GabrieleSim, Chang SunGrunnet, Louise GrothSim, In OkGrunst, MelissaSim, Seung-GyuGruszczyński, MarekSimães, ClaraGruszecka, AgnieszkaSimakov, SergeyGrygorowicz, MonikaSimenko, JozefGryko, KarolSimeon, James C.Grządziel, JarosławSimim, Mário Antônio De MouraGrzybowska, EwaSiminica, MarianGrzybowska, MagdalenaSimirgiotis, MarioGrzybowska, Magdalena EmiliaSimirgiotis, Mario J.Grzybowski, Jose Mario VicensiSimmaco, MaurizioGrzybowski, MirosławSimmenroth, AnneGrzywacz, AgataSimms, VictoriaGu, AlunSimões, João Filipe Fernandes LindoGu, FuSimões, Margarida PiresGu, JinbaoSimões, Marta FilipaGu, LinxiaSimões, NádiaGu, MinkyungSimões, PaulaGu, XuesongSimon, BarbaraGu, YaodongSimon, JorgeGu, YingliSimón, Miguel A.Guagnano, Maria TeresaSimon, Thomas R.Gualdi-Russo, EmanuelaSimonds, VanessaGualtieri, GiovanniSimone, GiuseppeGualtieri, MaurizioSimone, VillaGuan, QingyuSimonelli-Muñoz, Agustín JavierGuan, VivienneŠimoník, OndřejGuardone, LisaSimón-López, Leticia CarmenGuarini, AnnalisaSimons, GregoryGuarino, FrancescoSimopoulou, MaraGuariso, GiorgioSimović, SlobodanGuarnieri, GabriellaSimper, TrevorGuastaferro, Wendy P.Simpson, Matthew J. R.Guazzini, AndreaSimpson, Paul L.Gubar, ElenaSimpson, SteveGubhaju, LinaSims, JaneGubin, DenisSin, JonGuccione, JuliusSinanan, JolynnaGudiña, EduardoSinatra, MariaGuedes De Pinho, LaraSinclair, AmandaGuedes, Dartagnan PintoSinclair, CraigGuedes-Alonso, RaycoSinclair, MichelleGüeita Rodríguez, JavierSinden, KathrynGuell, CorneliaSinelnikov, MikhailGuenel, PascalSinelnikov, Mikhail YegorovichGuenther, JohnSinex, Donal G.Guerra, ManuelaSing, Swee LeongGuerra, PauloSingam Guerra-Balic, MyriamSinger, JosefGuerra-Martín, Maria DoloresSingh, AjitGuerrero Barona, EloisaSingh, AkanshaGuerriero, LuigiSingh, AmardeepGuers, John J.Singh, Amit K.Guest, MarcSingh, AnandGuevara, JenniferSingh, Brajendra KumarGugushvili, AlexiSingh, ChanpreetGugushvili, DimitriSingh, FizaGuharoy, RoySingh, GatikrushnaGuiard, BrunoSingh, InderpalGuicciardi, MarcoSingh, KamalendraGuida, AgostinoSingh, NadiaGuida, MicheleSingh, Puspendra P.Guidetti, GloriaSingh, RameshGuidetti, LauraSingh, ReemaGuidi, Caterina FrancescaSingh, RekhaGuidi, PatriziaSingh, SandeepGuido, FrancescoSingh, SarbjitGuido, Giuseppe PieroSingh, ShaktiGuigue, CatherineSingh, SushantGuijarro, FranciscoSingh, VinitaGuijo, ValerianaSingh, Vipul K.Guilherme, LuizaSingla, BhupeshGuillaume, PhilippeSingleton, ChelseaGuillén, Francisco JavierSinha, NiharikaGuillien, AliciaSinha, SudarsonGuillotin, BrunoSinharoy, ArindamGuimarães, Claudinei De SouzaSiniscalchi, Sabato MarcoGuimarães, Guilherme VeigaSiniscalco, DarioGuimarães, Jorge A.Sinski, JenniferGuimarães, Rafael AlvesSiobhan, WeareGuinat, ClaireSiopis, GeorgeGuirado, DamiánSioris, ChristopherGuirao, BegoñaSioui, MiguelGuirguis, AmiraSipiläinen, TimoGujski, MariuszSipos, KatalinGulasekaran, RajaguruSipos, PéterGulati, KapilSiqueira, CarlosGülçin, DeryaSiqueira, Carlos EduardoGüldiken, NurdanSiqueira, Hugo ValadaresGuligowska, AgnieszkaSiraj, Amir S.Gulis, GabrielSirbu, DanielaGüllich, ArneSireci, FedericoGulliver, AmeliaSirico, FeliceGullo, ParideSiristatidis, CharalamposGullón, PedroSiriwardena, MohanGulluscio, CarmelaSiriwardhana, NalinGumà, JordiSirodoev, IgorGumulya, MonicaSischka, PhilippGun, RichardSisti, AndreaGunasekaran, KulothunganSisti, DavideGunatillake, TiliniSisto, RobertaGundamaraju, RohitSit, Cindy Hui-PingGunier, BobSiu, FionaGunn, JohnSivak, LedaGuo, ChaoranSivakumar, SushamaGuo, Chih-HungSivaraj, Kishor KumarGuo, Chun XiangSivelle, VianneyGuo, FengbiaoSivrikova, NadezhdaGuo, JingchuanSixsmith, JudithGuo, JingshuSiziba, ​LindaGuo, Jong-LongSjödin, FredrikGuo, JunSkaathun, BrittGuo, LiangSkakon, JanneGuo, MingchunSkalickova, SylvieGuo, ShuaijunSkalioti, ChrysanthiGuo, WeiminSkalska-Kamińska, AgnieszkaGuo, WenjingSkarzynski, Piotr HenrykGuo, XuetaoSkelton, KaraGuo, YanSkenderi, Katerina P.Guo, YingqiSkenderidis, ProdromosGuo, YuntaoŠkerjanc, AlenkaGuozhang, ZhongSki, ChantalGuppy, FergusSkiba, MartaGupta, AdyyaSkjelvåg, Arne OddvarGupta, BhawnaSkoczylas, ŁukaszGupta, CharlotteSkogstad, MaritGupta, KuldeepSkolmowska, DominikaGupta, MohitSkordilis, EmmanouilGupta, NeeruŠkorput, PeroGupta, NehalSkotnicka-Zasadzień, BożenaGupta, NilakshSkouteris, HelenGupta, PrachiSkov, MarianneGupta, ShivamSkowron, AgnieszkaGupta, Sunil KumarSkrinjar, IvanaGupta, VibhutiSkrobot, WojciechGupta, VikasSkrzypski, MarekGupta, YashSkullerud, JonivarGuran, SerpilSkuratovskaia, DariaGuriec, NathalieSkurka, ChristoferGurko, TatianaSkvortsov, DmitryGurková, ElenaSkwarzec, BogdanGurnham, DavidSlack, MarionGurovich, Alvaro N.Slack-Smith, LindaGursoy, MerviŚlaski, SławomirGusarov, Artyom (Artёm)Șlencu, Bogdan GabrielGusi, NarcisSlesarenko, Inga V.Guski, RainerSlezák, RadovanGussoni, MaristellaŚliwerski, AndrzejGustafson, Christopher R.Śliwicka, EwaGustafsson, HenrikŚliwińska-Mossoń, MariolaGustav, StalhammarŚliwińska-Wilczewska, SylwiaGustavsson, JohannaSloan, KennethGutaj, PawełSloan, Robert AlanGuth, MartaSloey, TaylorGuthrie, RobertSlominski, AndrzejGutiérrez López, AlfonsoSłomka, ArturGutiérrez Ortega, MónicaSlottje, DanGutiérrez Puertas, LorenaSlouka, DavidGutiérrez Serpa, AdriánSłowikowska-Hilczer, JolantaGutiérrez, Ángel J.Ślusarska, BarbaraGutiérrez, DaniaSly, TimothyGutiérrez, EdgarSmaglo, Brandon G.Gutierrez, Juan Manuel ParrillaSmailienė, DaliaGutierrez, MonicaŠmajs, DavidGutiérrez-Arzaluz, MirellaSmalec, AgnieszkaGutiérrez-García, CarlosSmall, Roy S.Gutiérrez-Pérez, JoséSmalls, BrittanyGutiérrez-Uribe, JanetSmartt, Chelsea T.Gutman, GloriaSmereka, JacekGuttmacher, SallyŚmielak, BeataGuyon-Harris, KatherineŚmiglak-Krajewska, MagdalenaGuzek, DominikaSmiley, AbbasGuzik, MaciejSmiley, SarahGuzik, RobertSmirnov, AndrewGuzmán Ruiz, RocíoSmirnov, IlyaGuzmán, EduardoSmit, Albertus J.Guzman, FannySmit, Jasper V.Guzzi, GianpaoloSmit, Johannes HendrikusGwiazdowska, DanielaSmit, WarrenGwynne, KylieSmith, Albert F.Gyan, CharlesSmith, Anthony JohnGyant, LaVerneSmith, Ariana L.Gyawali, PradipSmith, Beth A.Györkös, ChristinaSmith, Brian S.H Shirvani, ArashSmith, Bryan K.Ha, EunyoungSmith, CharlotteHa, Min-SeongSmith, ClaireHa, SandieSmith, CourtneyHa, SeongwookSmith, CurtHa, YoonheeSmith, DonaldHaak, HarmSmith, Dylan M.Haarbauer-Krupa, JulietSmith, Genee S.Haarmann-Stemmann, ThomasSmith, JaredHaas, Emily J.Smith, KimothyHaase, HajoSmith, Lauren M.Habachi, MagdiSmith, Leonard B.Habanova, MartaSmith, MichaelHabuda-Stanic, MirnaSmith, Paul F.Hącia, EwaSmith, PeterHackenberg, StephanSmith, RobertHackett, DanielSmith, Sinead M.Hackman, ChristineSmith, Stephanie A.Haddad, Assed N.Smith, ThomasHaddock, Christopher KeithSmith, TracyHadigal, SusheelaSmith, Webb A.Hadioui, MadjidSmith, WilliamHadjadj, SamySmitham, PeterHadjar, AndreasSmith-Han, KelbyHadjisolomou, EkateriniSmoczyński, LechHadkhale, KishorSmol, MarzenaHadžić, NevenSmoląg, KlaudiaHafeez, MuhammadSmoluk-Sikorska, JoannaHafizi, SinaSmorti, MartinaHafner, ChristianSmreczak, BożenaHagedorn, LindaSmułek, WojciechHagège, AgnèsSmyer, MichaelHagen, BrianaSmyth, NinaHagendorff, AndreasSmyth, StuartHagenfeld, DanielSnarska, MałgorzataHager, DebbieSnawder, JohnHaghshenas, Sami ShaffieeSnell, ChristopherHagihara, KeisukeSnell, Cudore L.HAGIWARA, GoichiSnelling, WilliamHagiwara, KatsuroSnowden, Robert J.Hagner-Derengowska, MagdalenaSnyder, Christopher S.Hahad, OmarSnyder, Orman L.Hahn, AndreasSnyder, RuthHahn, AxelSo, Billy C. L.Hahne, JensSo, Wi-YoungHailikari, TelleSoares Teles, ArielHain, DebraSoares, Antonio GarciaHajek, AndréSoares, FabrízzioHajjar, Emily R.Soares, Fernanda CunhaHåkansson, Anders C.Soares, Jorge PintoHåkansson, KristerSobieska, MagdalenaHakeem, Faisal F.Sobolewski, EricHala, DavidSobotta, ŁukaszHalabi, CarmenSobrinho, Paulo De Souza CostaHalabi, Manal AlSobrino Sánchez, RaquelHaldar, SumantoSobrino-Garcia, ItziarHaldeman, LaurenSöderberg, PatrikHale, LisaSoderlund, GoranHaley, RobertSöderqvist, FredrikHalik, UmitSodhi, ManMohanHall Barbosa, Carlos RobertoSoeberg, MatthewHall, AnetteSoendergaard, MetteHall, Charles B.Sofaer, ShoshannaHall, Colin MichaelSofo, SeiduHall, Emily J.Sogorb Sánchez, Miguel ÁngelHall, Eric E.Sohel Mahmud, S. M.Hall, Jeffrey W.Sohye, LeeHall, Richard W.Soiza, RoyHall, SophieSojka, MariuszHall, SusanSokal-Gutierrez, KarenHall, Toby M.Sokang, Yasinta AstinHall, WayneSokolovskaya, Irina EduardovnaHalla, JamieSolache-Ríos, Marcos JoséHallam, Jeffrey S.Solaimanzadeh, IsaacHallerman, EricSolangi, Yasir AhmedHallewell Haslwanter, Jean D.Solanke, IyiolaHalley, SamuelSolanki, AshishHalliburton, BrendanSolanki, SumeetHalliday, EmmaSolano, CarmenHallingberg, BrittSolano, FranciscoHalski, TomaszSolano, Pedro RuizHaluza, DanielaSolar, OrielleHalvatsiotis, Panagiotis G.Solarino, BiagioHalvorsen, PriyaSolari-Twadell, P. AnnHama, AkiraSola-Rodríguez, SergioHama, SarkawtSoldan, MarosHamadi, Hanadi Y.Soldatenko, Sergei A.Hamamura, JunpeiSoldatos, Gerasimos T.Hamann, MartineSolenberg, AllenHamaoka, TakafumiSolimando, Antonio G.Hamarsheh, OmarSolimini, Angelo G.Hamasaki, HidetakaSolimini, RenataHamasaki, ShunsukeSolina, VittorioHambraeus, JohanSolinas, CinziaHamdia, KhaderSolingapuram Sai, Kiran KumarHamed, Moussa BoumadanSolís García Del Pozo, JuliánHamedi, Hamid RezaSolís, Luis Fernando MagañaHamerlynck, ErikSolis, Rafael R.Hamilton, LuzaanSolis-Soto, María TeresaHamm, NicholasSolmi, FrancescaHammad, KarenSolobutina, Marina M.Hammarlund, RebeccaSołoducho-Pelc, Letycja M.Hammond, WilliamSolo-Gabriele, HelenaHampel, DanielaSolomon, Nancy PearlHamra, GhassanSolovieva, YuliaHamrefors, ViktorSolovyev, NikolayHamułka, JadwigaSołowczuk, AlicjaHan, BingSołowiej, PiotrHan, GuanghuaSoltanifar, MohsenHan, HeesupSoltysik, BartłomiejHan, HongyunSołtysik-Piorunkiewicz, AnnaHan, Jeong-WonSolymosi, RekaHan, KihyeSommaruga, MarinellaHan, KyungManSomos-Valenzuela, Marcelo A.Han, LeiSomot, StephanieHan, LijianSomoza, BeatrizHan, MinhoSon, Chong-HwanHan, SeungYongSon, JongsangHan, Won-SikSon, KeunBaDaHan, WoojaeSon, YeonjungHan, XiaoSon, Youn-SukHan, Yong-HeSone, HidekoHan, Young-JiSone, ToshimasaHan, ZheSones, Amerian D.Han, ZhigangSong, ChaoHan, ZhiyongSong, ChunhuaHanaeus, JörgenSong, HakjoonHanaka, AgnieszkaSong, HemiinHanas, JaySong, In GyuHandcock, Phil J.Song, Jae WookHandel, MinaSong, JiajiaHandschin, Martin GrossmannSong, JungbinHaneke, EckartSong, PingHanes, DouglasSong, RuiHanisch, MarcelSong, Tai-JinHanke, Alexander A.Song, WenlongHanke, MarcSong, YaoHanke, WojciechSong, YeonsuHanke, WojciekSong, YongzeHanna, AlanSongsasen, NucharinHannah, OhSoni, AnitaHanne, HoffmannSoni, ChetnaHannon, AdrianSonne, James W.Hannonen, RiittaSonowal, HimangshuHano, Mary ClareSontrop, JessicaHanoune, BenjaminSoopramanien, AnbaHanowski, Richard JosephSophocleous, FrosoHansen, BrookeSorahan, TomHansen, CamillaSorbye, Sveinung WergelandHansen, David P.Sørebø, ØysteinHansen, KarolinaSorensen, CarlHansen, Keith A.Sorensen, JohnHansen, MargaretSorensen, KristineHansen, Tia G. B.Soria Garcia, Juan MiguelHanson, Nicholas J.Soriano Ayala, EncarnaciónHansson, Eva EkvallSoriano, Jose A.Hanstock, TanyaSormunen, JormaHantrakun, ViriyaSornay-Rendu, ElisabethHanuš, MartinSörnmo, LeifHanuszkiewicz, JustynaSornpaisarn, BunditHanyuda, AkikoSorokin, Mikhail Yu.Hanzl, MałgorzataSorra, JoannaHao, YixueSorrenti, LuanaHaque, AmlanSorrenti, VincenzoHarada, Kouji H.Sorrentino, AnnaHarada, YukinoriSorzabal-Bellido, IoritzHarada-Shiba, MarikoSosa Gomez, DanielHaralur, Satheesh B.Sospedra, IsabelHarbison, Justin E.Sotelo Monge, Marco AntonioHarbron, JanettaSoteras, InigoHardin, Heather K.Sotgiu, GiovanniHarding, AndrewSoto Varela, RobertoHarding, CeliaSoto, EnriqueHarding, ChrisSoto-Cámara, RaúlHarding, ElizabethSoubervielle-Montalvo, CarlosHardisson De La Torre, ArturoSoukissian, TakvorHardisson, ArturoSoulage, ChristopheHardman, Roy J.Soule, EricHardy, AnnSoundy, AndrewHardy, Lisa J.Sousa Vieira, María EstrellaHarel-Shalev, AyeletSousa, AlessandraHarenberg, Job F.Sousa, Ana Margarida Luís DeHarila, VirpiSousa, AngelaHarkin, Kenneth R.Sousa, AntónioHarley, DavidSousa, ArturoHarley, JamesSousa, Caio VictorHarley, Naomi HalldenSousa, CristinaHarmelink, Matthew M.Sousa, FilipeHarmon, IanSousa, GoretiHarmsen, MathijsSousa, Joao MiguelHaro, GonzaloSousa, Maria JoséHarper, Diane M.Sousa, MónicaHarper, GordonSousa, PauloHarper, Sara A.Sousa-Sá, EduardaHarrington, JulieSouter, Nicholas J.Harrington, Kathleen F.Souza, AmauryHarris, CarlaSouza, Claudia Daniele DeHarris, Edward N.Souza, Fátima Pereira DeHarris, Jason T.Souza-Dantas, Vicente CésHarris, KatieSovová, EliškaHarris, RobinSowa, JerzyHarrison, HenriettaSowada, ChristophHart, Nicolas H.Sowden, MilesHart, PrueSówka, IzabelaHartanto, AndreeSoyinka, OluwoleHarth, VolkerSozańska, BarbaraHarting, JannekeSpaanenburg, LambertHartley, KendallSpada, AlessiaHartlova, AnettaSpadaro, SavinoHartmann, AlexanderSpagnoli, PaolaHarton, AnnaSpagnolo, JessicaHartung, JohnSpahija, Jadranka A.Hartwig, JosephineSpang, EdwardHarty, PatrickSpano, GiuseppinaHarvey, AdamSparkes, ValerieHarvey, CarolineSpeake, HelenHarvey, JoanSpearing, SamHarzer, ClaudiaSpeelman, Diana L.Hasan, Abdel Fattah R.Spégel, PeterHasan, EmadSpeiser, Phyllis W.Hasan, Mohammad MonirulSpeklé, Erwin M.Hasan, RiasatSpena, AngeloHasaniya, Nahidh W.Spencer, KirstenHasegawa, KenichiSperber, GeoffreyHashem, AbulSperfeld, MartinHashemi, ShervinSperlì, GiancarloHashemkhani Zolfani, SarfarazSperlich, BirgitHashim, KhalidSpetea, MarianaHashimoto, MakotoSpezio, Teresa SabolHashimoto, TakeshiSphiwe, MadibaHashimoto, YoshitakaSpickett, JefferyHasman, ArieSpiegel, MartinHasmeena, KathuriaSpiegler, JulianeHasni, SarfarazSpielmanns, MarcHaspula, DhanushSpier, CarlosHass, Alisa L.Spijker, JeroenHassall, MaureenŠpiláček, MichalHassan, AbeerSpiliopoulou, MyraHassan, AmanySpilki, FernandoHassan, Amr Abdulfattah HausienSpinas, EnricoHassan, IbrahimSpinato, SergioHassan, MarwaSpinazzè, AndreaHassan, Tidi MaharaniSpindler, HelleHassanain, MohammadSpinelli, LucianaHassani, Mohamed-AmineSpinner, LaurenHassell Jr., James E.Spires, MarkHassenrück, ChristianeSpirgienė, LinaHastings, GerardSpirig, ChristophHaszard, JillSpitschan, ManuelHata, AkihisaSpittle, BruceHata, Fernando TeruhikoSpitzenstetter, FlorenceHatch, ElizabethSpizzirri, Umile GianfrancoHatchett, AndrewSpohnholz, JohannesHatchett, LenaSpolaor, FabiolaHategan, Camelia DanielaŠpoljar, MariaHatfield, GillianSpöndlin, JuliaHatjimihail, AristidesSponsiello, NicolaHattink, BartSpoorenberg, ThomasHattori, ToshioSprajcer, MadelineHaubenhofer, DoritSprave, TanjaHauer, KlausSpreat, ScottHaugen, Håvard JSpringer, AndreaHaughton, JonathanSpringer, AndrewHauret, LaetitiaSpringer, RoxanneHauser, Sheketha R.Springgate, BenjaminHauser, Stephan J.Springs, StacyHauser, W. AllenSprovieri, MarioHausien, AmrSprunger, JoelHautière, NicolasSpychalska-Wojtkiewicz, MonikaHawe, PennySpyratou, EllasHawes, SloaneSquillacioti, GiuliaHawi, ZiarihŚrednicka-Tober, DominikaHawila, Abed Al WaheedSrinivas, SharanHawke, Thomas J.Srinivasagan, RamkumarHawkins, DavidSrinivasan, BharaniHawkins, FedericoSrivastava, AkritiHawkins, MarquisSrivastava, David ShivaHawlader, Mohammad Delwer HossainSrivastava, PranayHaworth, Kristina M.Sriwastva, Mukesh KumarHay, IanSserwadda, MartinHayakawa, KazuichiSt Clair, MichelleHayashi, KatsumaSt Hill, CatherineHaycock, BruceSt Laurent, ChristineHayden, JohnStabauer, MartinHayer, TobiasStacherzak, AgnieszkaHayes, SandiStachowska, EwaHayes, TraciStaderini, EdoardoHayford, Barbara L.Stafford, AmandaHayman, Laura L.Stafford, FrankHaynes, AshleighStahl, Hans-ChristianHayward, AnnaStahre, MandyHayward, Richard DavidStaines, AnthonyHazarika, BhabeshStájer, AnetteHazell, PhilipStajnko, AnjaHazler, Richard J.Stakišaitis, DonatasHe, BaojieStallmann, ChristophHe, Guan-HaoStallones, LorannHe, HuijunStallsmith, BruceHe, Karen Y.Stalsberg, HelgeHe, LiStamatakis, MichaelHe, Mike Z.Stamatis, AndreasHe, QinghuaStamatis, NikolaosHe, SongjieStamovlasis, DimitriosHe, WeiStana, AlexandruHe, ZhenStanciu, Corneliu N.Heads, Angela M.Stanek, AgataHeal, MathewStanek-Tarkowska, JadwigaHealy, KristenStanforth, Philip R.Healy, RobinStanhope, JessicaHeaney, Catherine A.Staniec, IwonaHeath, GregoryStaniek, HalinaHeath, HannahStaninska-Pięta, JustynaHebel, KatarzynaStanirowski, Paweł JanHébert, JohanneStanišić, SvetlanaHeck, TamaraStanistreet, DebbiHeckman, CarolynStaniszewska, AnnaHeckmann, MatthiasStaniszewski, JakubHector, DebraStankevičienė, JelenaHector, MarkStanley, John K.Hedberg, PärStanley, Jone A.Hedges, JerrisStanojevic, Dragana M.Hedlund, JamesStanula, ArkadiuszHedman, HaydenStara, VeraHeemskerk, ChristinaStark, John D.Heemsoth, TimStarling, Anne P.Heesch, FlorianStarościak, WojciechHefele, PeterStarostka-Patyk, MartaHegazy, Mostafa A.Stasis, Antonios Ch.Hege, AdamStastny, PetrHegedűs, CsabaStaszkiewicz, PiotrHegewald, JaniceStaudacher, Jonas JaromirHeiland, StefanStaunstrup, Nicklas HeineHeilman, Renata M.Stauvermann, Peter JosefHein, NilsStavi, IlanHeinävaara, SirpaStavrinou, PinelopiHeine, FlorianStavropoulou, AretiHeinonen, MaaritStawiński, WojciechHeinonen-Tanski, HelviStay, SharonHeinrich, KatieSteardo, LucaHeinz, AndreasStechmiller, JoyceHeinz, MelindaStecko, JustynaHeishman, AaronSteed, LizHeisinger, StephanSteele, DanielHejnowicz, AdamSteele, Meredith K.Helajärvi, HarriSteels, ElizabethHeld, Mary LehmanSteels, StephanieHeld, PhilippStefan, OvidiuHelden, MaartenȘtefan, Simona CătălinaHeldner, MirjamStefana, ElenaHele, KarlStefanaki, CharikleiaHelen, FisherStefanelli, Luigi VitoHeller, DeboraStefani, LauraHellman, ThereseStefania, OrrùHellström, StenStefaniak, Aleksandr ByronHellwig, NielsStefanini, AlessandroHelm, EmmanuelStefanoff, PawelHelmy, YosraStefanou, StefanosHelou, NancyStefanowicz, JoannaHelou, SamarStefanska, AnnaHelsloot, IraStehle, MariaHemkemeyer, MichaelStehle, PeterHemmingway, AndreaSteigen, Sonja ErikssonHemmingway, AnnStein, AngelicaHemphill, Jean CroceStein, CherylHemprich, AlexanderStein, PaulaHenderson, BarronSteinberg, JaneHenderson, ClaireSteinberg, MichaelHendren, RobertSteiner, JeanHendrich, Ann L.Steinke, DouglasHendriks, EefjeSteinmetz, ChristineHendriks, TomSteinsvoll, SveinHendry, AnneSteiro, TrygveHendryx, MichaelStella, Alex BuoiteHenein, MichaelŠtemberger, VesnaHeng, Lee KhengStemmer, Paul M.Heng, YanStemplewski, RafałHenley, Shauna C.Stenz, LudwigHennegan, JulieStenzel, Ashley E.Hennessy, CatherineŠtěpánek, LadislavHennessy, Emily AldenStepanic, VisnjaHenning, FrancloStepanova, Nadezhda YuilevnaHenning, GeorgStepensky, DavidHenri, DominicStephenson, Max O.Henrickson, MarkStepien, AgnieszkaHenriques, RenatoStępień, BeataHenriques-Neto, DuarteStepien, Karolina M.Hensel, EdwardStępień, ŁukaszHensel, LukasStępień-Pyśniak, DagmaraHensvold, AaseStępniewska, MałgorzataHeo, Hyun-HeeSterczyńska, MonikaHeo, JinmooSternin, AvitalHeo, SeulkeeStetina, Birgit U.Heo, WookjaeStevan Jr., Sergio L.Hepsomali, PirilStevens, DavidHeras-Pedrosa, Carlos De LasStevens, KevinHerath, H. M. Ayala S.Stevens, RebekahHerbeć, AleksandraStevenson, BrittanyHerber, OliverStevenson, MarkHerbert, AnthonyStewart, AdamHerbert, JarosławStewart, JamesHerbuela, Von Ralph Dane M.Stewart, ThomasHerbut, PiotrStewart, Wendy A.Herckes, PierreSthel, Marcelo SilvaHerda, AshleyStiburkova, BlankaHerdeiro, Maria TeresaStidham, Andrea WarnerHerlenius, Eric A. P.Stienstra, DeborahHerman, KrzysztofStilianakis, NikolaosHermann, CarollStiller, Carl-OlavHermanussen, MichelStingeni, LucaHermassi, SouhailStinghen, Andréa Emilia MarquesHermida, MargaridaStinson, JulieHerminghaus, AnnaStjepan, SpaljHermoso-Orzáez, Manuel JesúsStobdan, TseringHernández Calzada, Martín AubertStock, ChristianeHernández Fernández, AntonioStock, Naomi L.Hernandez Gomez, Juan CarlosStockfisch, NicolHernández Paniagua, Iván YassmanyStockwell, TimothyHernández, Antonio FernándezStockwell, William R.Hernandez, IgnacioStoecker, BarbaraHernández, MartaStoecklin, DanielHernández, SusanaStoffers, JolHernández-Aguado, IldefonsoStoicanescu, DorinaHernández-Arellano, Juan LuisStojić, AndrejaHernández-Escobedo, Quetzalcoatl CruzStojmenović, MarijaHernández-Hernández, ElenaStokes, LynissaHernández-Martínez, AlbaStokmans, MiaHernández-Martínez, AntonioStone, EmilyHernández-Mosqueira, ClaudioStone, Heather NicoleHernández-Núñez, EmanuelStone, KahlerHernández-Padilla, EduardoStoneham, MelissaHernández-Padilla, Jose ManuelStonerock, Gregory L.Hernández-Paniagua, Iván YassmanySt-Onge, Marie-PierreHernández-Pérez, IvánStookey, JodiHernandez-Tejada, MelbaStoops, CraigHernández-Torruco, JoséStorcksdieck Genannt Bonsmann, StefanHernando, CarlosStorek, JosephineHerold, David M.Storm, Fabio AlexanderHerold, FabianStout, JeffreyHerold, ZoltanStraburzynski, MarcinHéroux, PaulStraccia, PatriziaHerr, RaphaelStragierowicz, JoannaHerrador-Alcaide, TeresaStrain, TessaHerrador-Colmenero, ManuelStraiton, Melanie L.Herrala, MikkoStrametz, ReinhardHerrera, ManuelStrasburg, Virgílio JoséHerrera-Melián, José AlbertoStrati, VirginiaHerrera-Peco, IvánStrauß, MarkusHerrero De La Parte, BorjaStrauss, SusanHerrero, Juan BautistaStreimikiene, DaliaHerrero, María JesúsStrelitz, JeanHerrero-Diz, PaulaStreltzer, JonHerrero-Hernández, SilviaStretesky, PaulHerrero-Montes, ManuelStricker, RaphaelHerrmann, AlisonStrielkowski, WadimHerrmann, WolfgangStringfellow, Anne M.Herruzo, CarlosStringham, JamesHerruzo, JavierStrizhitskaya, OlgaHersch, JoniStroebele-Benschop, NanetteHertelendy, AttilaStrohfus, Pamela K.Hertrich, IngoStröhle, MathiasHervada Sala, CarmeStroiney, DebraHervás-Marin, DavidStromberg, ZacharyHervert, SarahStroud, CraigHerzberg, MartinStrout, Irina IgorevnaHerzog, HaroldStruk, MichalHesketh, ThereseStrumylaite, LoretaHeslop, BenjaminStrunecka, AnnaHess, MoritzStrungaru, Stefan-AdrianHesse, MortenStruzik, ArturHesselink, Jan WillemStrzałka, DominikHessheimer, Amelia J.Strzelecki, ArturHeßling, MartinStuart, HeatherHeston, Thomas F.Stubblefield, Andrew P.Hettiarachchi, HiroshanStucchi, MartaHeurtebise, Jean YvesStudlar, DonleyHew-Butler, TamaraStummer, HaraldHewit, JenniferStupar, AleksandraHewitt, JonathanSturm, DavidHewson, DavidSturm, HeidrunHeyn, PatriciaSturmey, RogerHeyndrickx, MarcStuss, Magdalena M.Hibbard, PaulStygar, DominikaHibbert, SallySu, Chung-HweiHicar, Mark D.Su, DejunHickey, AmandaSu, LeiHickey, HuhanaSu, Shih-binHidalgo Dattwyler, Rodrigo AlejandroSu, ShiliangHidalgo, NinaSu, WeihengHidalgo, Pedro L. PancorboSu, XukunHidalgo, VictoriaSuarez Relinque, CristianHidalgo-García, CésarSuarez, Maria J.Hieber, PeterSuárez, RafaelHiggins, John P.Suarez-Barraza, Manuel F.Higgins, KellySuárez-Carballo, FernandoHiilamo, HeikkiSuárez-López, María JoséHijano, Diego R.Suari, YairHiki, KyoshiroSubbiah, RameshHikita, NaokoSubbian, SelvakumarHildebrandt, Mark L.Subedi, PraveenHildenbrand, Sibylle L.Subhi, YousifHildreth, BlakeSubiza, MikelHildreth, EasonSubramaniam, MythilyHileman, Stanley M.Subramanian, ParthasarathiHilhorst, MarcSubudhi, SonuHill, Adam O.Suchanek, JakubHill, AllenSuchocka, MarzenaHill, CameronSuchomel, Timothy J.Hill, JennieSuchorab, ZbigniewHillegass, William B.Suchý, JiříHilliard, AaronSudakov, IvanHillier, AmySudarsono, Anugrah SabdonoHilliges, RitaSudolska, AgataHills, Andrew P.Sudraba, VelgaHilscher, RainerSudzina, FrantisekHilson, GavinSuen, I-Shian (Ivan)Hilton, ClaudiaSuen, Jacky Y.Hilton, Thomas F.Sugaya, NagisaHimeno, SeiichiroSuggate, SebastianHimmerich, HubertusSugiarto, TommyHinds, JoeSugiura, MikikoHinds, RupertSugiyama, KemmyoHines, Anika L.Suh, Dong-ChurlHines, StellaSuh, JangwonHingorani, RittuSui, HongjinHinkelbein, JochenSui, XiaohongHino, KimihiroSujo-Montes, LauraHinz, LisaSukegawa, ShintaroHiragi, ShusukeSukumaran, Sunil KumarHirai, TakashiSukys, SauliusHirano, TsunahikoSulaiman S., IbrahimHiraoka, KoichiSuleiman-Martos, NoraHiraoka, SakikoSulewski, PiotrHiraumi, HarukazuSuliga, EdytaHirokawa, KumiSulis, EmilioHiroshi, UreshinoSulkowski, AdamHirsch, ChristianSułkowski, ŁukaszHirsch, Mark A.Sullivan, James A.Hirsch, OliverSullivan, JosephHirschberg, CosimaSullivan, Kimberly A.Hirschtritt, MatthewSum, Raymond Kim-WaiHirshler, YafitŠūmakaris, PauliusHirvensalo, MirjaSumanen, HillaHisako, YoshidaSumin, Aleksei N.Hiscock, RosemarySumiyoshi, MasatakaHitch, GeetaSumma, VitoHites, LisleSummers, James KevinHitlin, SteveSummers, MonicaHiwa, SatoruSumner, RebeccaHjelmen, Carl E.Sumner, WaltonHjorth, PederSun, CaijunHladish, ThomasSun, ChengwenHlusko, Leslea J.Sun, ChongkuiHnatiuc, MihaelaSun, ChuanwangHo CM, RogerSun, FangminHo, Daniel S. Y.Sun, Feng-HuaHo, EnochSun, GuanghaoHo, Jeffrey C. F.Sun, GuohuiHo, JörgSun, HaiyanHo, Ken Hok ManSun, HongleiHo, Nguyen-Nhu-YSun, HuahuaHo, Pei-ShanSun, HuapingHo, Roger C.Sun, JianHo, Thanh TamSun, JianchunHo, Xuan-HungSun, JiaoHo, Yi-HuiSun, JieHoagland, BethSun, JudyHoang, Linh N.Sun, Kai SingHoang, NamSun, Li-YunHoang, Thuong N.Sun, LvhuiHoashi, ToshihikoSun, MingjingHobbelen, HansSun, MingKuanHobbs, SteveSun, ShengzhiHobson, HannahSun, WenchaoHobusch, GerhardSun, WenfeiHobza, VladimirSun, WinnieHock, EmmaSun, XiaHodge, Allison M.Sun, XuanHodges, LynetteSun, Yan (Shandong University of Finance and Economics) Hodges, MichaelSun, Yan (University of Chinese Academy of Sciences) Hodgins, MargaretSun, YiHodgson, Douglas J.Sun, YuHodgson, IanSun, YuanxiHoeksema, BertSun, YuliangHoenig, Helen MarieSun, Yun-LuHoepner, Lori A.Sun, YuxiangHoerr, RobertSun, YvonneHof, HerbertSun, ZhanboHoff, RodneySun, ZhengwangHoffer, Edward P.Sun, ZhimingHöffken, Johanna I.Sundar, Krishna M.Hoffman, StevenSunde, UweHoffmann, Lucia VieiraSunderland, BruceHofmann, PeterSundin, EbbaHofmeyr, AndreSundström, Mare LõhmusHögberg, GöranSuñer Soler, Maria RosaHogg, JenniferSung, JidongHoinoiu, TeodoraSung, JoohyunHojan, KatarzynaSung, Joseph J. Y.Hokama, TomikoSung, MinkiHoła, BożenaSung, Wei-YingHolbrook, JackSung, Wen-WeiHolcman, KatarzynaSungthong, RungrochHolcomb, Jason P.Sunindijo, RizaHolden Bergh, IngunnSunyer, JordiHolec, JosefSuominen, Sakari B.Holecki, MichałSuperti, FabianaHolen, MariSupervía, Pablo UsánHolesovsky, JanSuppan, LaurentHoll, AdelheidSurace, AnthonyHolland, MarkSuranyi, AndreaHolland, MichaelSurawski, Nicholas C.Holle, RolfSurber, SarahHolliday, Katelyn M.Surdacka, AnnaHolliday, L. ShannonSurdacki, AndrzejHolm, JesperSurguchov, AndreiHolm, RochelleSuriano, FrancescoHolman, E. AlisonSuryadevara, VidyaniHolman, HarlandSuryavanshi, SantoshHolmboe, LarsSuryavanshi, Santosh V.Holmes, DawnSusana, Rodríguez MartínezHolmes, JohnSusarrey-Arce, ArturoHolmes, Melissa GaleaSusic, MichaelHölscher, ChristianSuso-Ribera, CarlosHolsinger, EvaSussman, StevenHolst, HelleSut-Lohmann, MagdalenaHoltorf, Anke-PeggySutoh, YoichiHolvad, TorbenSutton, AnnaHolwerda, AnjaSutton, JillHoly, RichardSuzui, MasumiHołyńska-Iwan, IgaSuzuki, AyakoHomaeigohar, ShahinSuzuki, KeisukeHomem-de-Mello, MauricioSuzuki, SeitaroHomma, TetsuyaSuzuki, TakeyasuHon, CarolSuzuki, TohruHonda, AkikoSuzuki, Yuichiro J.Honda, HirotoSuzuki-Kerr, HarunaHonda, ShunichiSuzurikawa, JunHonda, Trenton JamesSvartengren, MagnusHonda, YasushiSveinsson, ThórarinnHonda-Okubo, YoshikazuSvendsen, KristianHonea, JoySvensson, AnnHoneycutt, MichaelSvoboda, JiříHong, BoSwacha, JakubHong, Bo YoungSwail, Watson ScottHong, Cheng-YihSwamikannu, XavierHong, Chien-TaiSwamy, GaneshHong, Hyun SookSwanepoel, LibbyHong, Joo YoungSwann, BrianHong, Jun SungSwann, William L.Hong, Nien-MingSwartz, Ann M.Hong, TaoSweeney, AllisonHong, Wei-ChiangSweeney, James F.Hong, Young-SeoubSwerczek, ThomasHong, YuxiangŚwierk, DariuszHonjo, KeitaSwinarew, AndrzejHonneffer, Julia B.Sy, AngelaHonório, SamuelSybilski, Adam J.Hood, Darryl B.Sycinska-Dziarnowska, MagdalenaHood, RalphSyczewska, MalgorzataHooda, JagmohanSydora, Beate C.Hooker, Angelo B.Syed, IffathHooker, BrianSyed-Abdul, Majid MufaqamHooman, AmidSygit, KatarzynaHooper, GarySykes, SusieHoover, Anna GoodmanSymonds, PhilHoover, Donald R.Symons, MartynHoover, Elizabeth M.Sypion-Dutkowska, NataliaHopkins, Benjamin D.Syrjä, PasiHopkins, ShelleySyropoulos, ApostolosHopman, NonkeSytar, OksanaHopman, RachelSyx, DelfienHopp, Russell JamesSzabados, EszterHopwood, Christopher JamesSzabo, AttilaHoracek, TanyaSzabo, GyulaHorai, YoshiroSzabova, ZuzanaHoran, DavidSzaferski, WaldemarHoran, KristinSzafranek-Nakonieczna, AnnaHorberry, TimSzafraniec, MałgorzataHorel, ÁgotaSzajerski, PiotrHoriuchi, MasahiroSzajt, MarekHoriuchi, SatoshiSzámel, LászlóHorlyck-Romanovsky, Margrethe F.Szarek-Gwiazda, EwaHorn, HaraldSzarek-Iwaniuk, PatrycjaHornby, BelindaSzark-Eckardt, MirosławaHorne, Rosemary S. C.Szatkiewicz, Jin P.Hornik, BeataSzczepanek, JoannaHornsby, AmandaSzczepankiewicz, BarbaraHornsby, William GuySzczepankiewicz, ElżbietaHornung, SeverinSzczepańska, AgnieszkaHorobet, AlexandraSzczepański, MarekHorowitz, June AndrewsSzczerbinski, LukaszHorowitz, Robert A.Szczesniak, DorotaHorsfall, DanSzcześniak, MałgorzataHorst, AxelSzékely, AndreaHorstmann, Kai T.Szekely, GyorgyHorswill, Craig A.Szeleszczuk, ŁukaszHort, KrishnaSzeliga, JacekHortobagyi, TiborSzemik, SzymonHorton, TeresaSzeto, GraceHortu, IsmetSzewczak, KamilHorvath, AngelaSzewczyk, RomanHorváthová, PetraSzewrański, SzymonHoseini-Yazdi, HoseinSzigeti, CecíliaHoshide, Aaron K.Szilágyi, Kinga M.Hosie, AnnmarieSzilveszter, SzabolcsHosmane, NarayanSzlagatys-Sidorkiewicz, AgnieszkaHosnijeh, F. SaberiSzmagliński, JacekHosoda, MasahiroSzolnoky, GyozoHosoyamada, MakotoSzóstak, MariuszHossain, FaruqueSzóstek-Mioduchowska, AnnaHossain, SharminSzpilko, DanutaHossain, Syeda ZakiaSzpotanska-Sikorska, MonikaHossein Mardi, AliSzromek, AdamHosseinian-Far, AminSztanke, KrzysztofHosseinifarhangi, MohsenSztubecka, MałgorzataHotos, George N.Szücs, AnnaHou, FujiangSzulc, KarolinaHou, Hong-ManSzulc-Dąbrowska, LidiaHou, JackSzulczewska, BarbaraHou, RuixueSzultka-Młyńska, MałgorzataHou, Wen-HsuanSzumilewicz, AnnaHou, XiaorongSzumińska, DanutaHouck, James A.Szydło, JoannaHoudmont, JonathanSzymańska, ElżbietaHoughton, AdeleSzymańska-Pulikowska, AgataHoughton, Lesley A.Szymborski, TomaszHoulden, VikkiSzymczak-Pajor, IzabelaHoulind, Morten BaltzerSzymenderski, JanHovenga, EvelynSzymkowiak, AndrzejHoward Wilsher, StephanieSzyszkowicz, MieczysławHoward, JeffreyTaank, VikasHowden, Erin J.Tabaka, PrzemyslawHowden, ReubenTabatabaei-Jafari, HosseinHowe, BrianTaber, Christopher B.Howell, BobTabernero-Duque, María-JesúsHowes, Laurence GuyTaborda-Barata, LuisHowitz, PierreTaborri, JuriHoxha, ArtanTaccone, AntonioHoyle, AndrewTacconelli, StefaniaHrdalo, InesTachikawa, HirokazuHrdy, JiriTackett, AlaynaHribar, LawrenceTada, AkioHruska, ZuzanaTada, MitsunoriHsiao, Sheng-MouTaddesse, EphremHsieh, Chi-Ming Tadeu, PedroHsieh, Chun-MingTadić, VanjaHsieh, Hsiu-ChinTafuri, DomenicoHsieh, Hsiu-FenTagarakis, GeorgiosHsieh, Huey-HongTagele, Setu BazieHsieh, Jin-chiTaggart, TamaraHsieh, Jing-ChziTaghipour, AtourHsieh, Kuen-ChangTagle, MatiasHsieh, Nan-HungTagliavia, MarcelloHsieh, Pei-HsuanTaguchi, KenseiHsieh, Ping-HungTaherzadeh, MohammadHsieh, Tsung-ChengTai, Yen F.Hsieh, Tusty-JuanTaiar, RedhaHsieh, Yi-ChenTaiki, MoriHsu, ChinyuTaira, YasuyukiHsu, Fu-YinTaishi, NakamuraHsu, Hui-ChuanTajan, NicolasHsu, Jih-TayTajani, FrancescoHsu, Jui-TingTajik-Parvinchi, DianaHsu, Li-ChiTajiri, YujiHsu, Ping-ChingTak, Erwin C. P. M.Hsu, Wei-LingTakahashi, KojiHsu, Wei-TingTakahashi, ShoHu, BoTakaidza, IsaacHu, ChengTakaki, ManabuHu, Ching-YaoTakakura, MinoruHu, DalinTakala, JukkaHu, DanningTakamura, NoboruHu, FeiTakano, AiHu, GuangjiTakano, TakakoHu, GuoqingTakasawa, KenHu, HelenTakata, TomoakiHu, HuiTakegata, MizukiHu, JiafenTakemura, KazuhisaHu, JiaxiTakenaka, ShotaHu, JingtianTakeuchi, KenjiHu, Jin-LiTalavera -Velasco, BeatrizHu, JiweiTalavera, MartaHu, JunhuiTalbot, BenoitHu, LiangTalbot, SimonHu, LiangjunTalbott, Evelyn O.Hu, LonghuaTalei, AminHu, MaocongTalifu, DilinuerHu, MinTallaki, MouhcineHu, QijunTallarico, MarcoHu, QingTallini, MarcoHu, Shih-ChengTallman, DinaHu, TangaoTallman, Paula SkyeHu, TaoTallner, AlexanderHu, WeiTalmage, CraigHu, Wei-PingTalukder, ByomkeshHu, XiTalukder, Mohammad Radwanur R.Hu, XiaoyuTam, Hon LonHu, Xue-FengTamaddun, KaziHu, YangTamai, YuyaHu, Yi FeiTamaki, NaofumiHu, YuanTamás, Varga JánosHu, ZhengYiTamayo-Ortiz, MarcelaHua, JieTambe, ChetanHuaico, AnaTambor, MarzenaHuang, ChaoTamburrano, AndreaHuang, CharlesTambyraja, Sherine R.Huang, Chien ChungTamošaitienė, JolantaHuang, Chin-HuangTamošiunas, AbdonasHuang, Chi-PingTampa, MirceaHuang, Chu-YuTampieri, FrancescoHuang, CongTamulevicius, NaurisHuang, DanlianTamura, KosukeHuang, DaquanTan, Bee K.Huang, DinaTan, Bee KangHuang, FeiTan, Chun LiangHuang, GanlinTan, JibinHuang, GuangweiTan, JinhuaHuang, GuoheTan, LiboHuang, HaiqiuTan, NingHuang, Han HsiangTan, Paul Juinn BingHuang, HanbinTan, Rui ZhenHuang, HaoTan, Shu LingHuang, Hong-YuanTan, SongwenHuang, HuayiTan, WillieHuang, Hung-ChungTan, YalunHuang, Hung-PinTan, Yinliang (Ricky)Huang, JianTanabe, KatsuyukiHuang, JianshengTanabe, ShihoriHuang, JianyingTanaka, HiroakiHuang, JiayanTanaka, Hiroyoshi Y.Huang, JijunTanaka, HiroyukiHuang, Jor-ManTanaka, KeikoHuang, JunmingTanaka, Marcel OkamotoHuang, Kuang-TzuTanaka, MasahiroHuang, LeslieTanaka, MasaruHuang, LiTanaka, MasashiHuang, Lian-HuaTanaka, SatoshiHuang, LihongTanaka, Tim HideakiHuang, Li-TungTanda, Maria LauraHuang, MengnaTang, AnnaHuang, MinTang, Anson Chui YanHuang, Paul Li-HaoTang, ChengxiangHuang, QianTang, JijunHuang, Ren-YeongTang, JinjunHuang, Shiang-FenTang, JonathanHuang, TailinTang, JulianHuang, TaoTang, JunqingHuang, Tse-HungTang, LongtengHuang, Tzu-HaoTang, MinghuaHuang, Wei-ChunTang, NingHuang, Wendy YajunTang, ShangfengHuang, WilfredTang, ShifangHuang, Wu-JangTang, ShiyuHuang, XianyuTang, SongHuang, XianzhengTang, VictorHuang, XiaobinTang, XinHuang, XiaohuTang, YilangHuang, XiaolongTang, YuanHuang, Yen-MingTang, ZhihuaHuang, YingTang, ZongliHuang, Yi-TingTanisawa, KumpeiHuang, YuTankiewicz, MatyldaHuang, Yu-ChihTanner, GritHuang, Yu-ChuTanniru, MohanHuang, Yung-FuTannous, KathyHuang, Yung-KaiTano, MauricioHuang, YuruTanoubi, IssamHuang, Zuyi (Jacky)Tanton, RobertHubálovský, ŠtěpánTanwar, VineetaHubbard, KarenTao, ChenqiHübenthal, MaksimTao, DaHuber, Max A.Tao, FengmingHuck, OlivierTao, KaiHuda, Fauzia AkhterTao, YanHuddlestone, LisaTao, YuHudson, PennyTapia Serrano, Miguel ÁngelHue, OlivierTappia, ParamjitHuebner, HannaTappin, DavidHuerta, MiguelTara, KamHuertas-Delgado, Francisco JavierTaradaj, JakubHueso-Montoro, CesarTarango, JavierHuettig, FabianTarannum, MubinHuey, SamanthaTarantino, GiovanniHugelius, KarinTaraseviciene, ZivileHughes, Jean R.Tarazona, FranciscoHughes, Jennifer C.Tarazona-Santabalbina, Francisco JoséHughes, Matthew WilliamTardivo, StefanoHugos, Cinda L.Tardy, Anne-LaureHuh, Joo YoungTari-Keresztes, NoemiHui, AdaTariq, Usman SaeedHui, Ho SuTarkowski, MaciejHui, JaneTarlov, ElizabethHui, Shuk Yi AnnieTarp, JakobHuijsman, RobbertTarperi, CantorHuiyong, YinTarraga-Minguez, RaulHuizeling, EleanorTarrósy, IstvánHumberto, MateusTartaglia, GianlucaHumer, ElkeTartaglione, EnzoHünefeld, LenaTasca, Andrea LucaHung, Chang-HungTashima, ToshihikoHung, Fei ShuoTaskinen, SaraHung, Li-FangTassier, TroyHung, Li-SanTassitano, Rafael MirandaHung, Maria Shuk YuTatarinov, FedorHung, WeihsiTateda, MasafumiHung, Yu ChiangTatnell, RuthHunova, IvaTattoli, LuciaHunt, JodiTatullo, MarcoHunt, MelissaTaufen, AnneHunt, RosTavakoli, Javad (Australia)Hunter, DavidTavakoli, Javad (Canada)Hunter, Elizabeth G.Tavakoli, Norma P.Hunter, ErinTavares, JoãoHunter, MyraTavares, SilviaHunter, RachaelTavartkiladze, GeorgeHunter, TinaTaverna, LiviaHuntingford, JaniceTavernise, AssuntaHuntington-Moskos, LuzTawia, SusanHuo, TengfeiTawiah, Vincent KonaduHuo, YimingTaxbro, KnutHuppertz, TilmanTaylor, AlyxHuq, Md. EnamulTaylor, AndyHuq, Mona SayedalTaylor, C. BarrHuque, RumanaTaylor, CaitlinHurley, JenniferTaylor, Carlene H.Hurley, Karinna B.Taylor, GemmaHurst, Brett L.Taylor, GordonHursthouse, Andrew S.Taylor, GreigHurtado Oliva, Miguel ÁngelTaylor, Jonathan W.Hurtado Pomares, MiriamTaylor, Matthew (UK)Hurtado-Mellado, AlmudenaTaylor, Matthew (USA)Hurtig Wennlöf, AnitaTaylor, Rachael M.Hurwitz, ShepardTaylor, RachelHusain, IslamTaylor, Thomas J.Husain, Muhammad JamiTaylor, ZackHusárová, DanielaTaylor-Piliae, RuthHuseth-Zosel, Andrea L.Taysum, AlisonHuseynov, SamirTchepel, Oxana AnatolievnaHuss, EphratTe Nijenhuis, JanHuss, FredrikTeah, Heng-YiHussain, MulazimTecco, SimonaHussein, HussamTeh, Soon LiHussman, John P.Tehrani, RouzbehHuston, Daniel C.Teich, IreneHuta, VeronikaTeisseyre, AndrzejHutchinson, MarkTei-Tominaga, MakiHutchison, Robert E.Teixeira Vale, VeraHutorowicz, AndrzejTeixeira, AlfredoHutton, VickiTeixeira, Ana MariaHuttunen-Lenz, MaijaTeixeira, Danielle Cardozo FrascaHuxley, PeterTeixeira, LaetitiaHuynh, ChiTeixeira, LeandroHuynh, NeilTejada Giráldez, Miguel A.Huynh, Thanh-CanhTejedor, SantiagoHuynh, TranTejera, AliciaHwang, AiwenTejpal, ShilpaHwang, Chueh-LungTelejko, MarekHwang, Chul SueTeles, SoraiaHwang, Dae-seokTeli, Mahesh KumarHwang, Eun JeongTella, SusannaHwang, Fang-MingTellez Jurado, LuciaHwang, JaejinTéllez-Téllez, MauraHwang, JihyeTemam, SofiaHwang, JinsooTemesi, ÁgostonHwang, JooyeonTempalski, BarbaraHwang, Kwon TackTemple, Jennifer L.Hwang, Lee-ChingTemple, NormanHwang, SungjaeTemple, TraceyHwang, Won-JuTenace, ValerioHwu, Ruth S.Tenbrock, KlausHwu, Yueh-JuenTendais, IvaHybiske, KevinTeng, Ru-JengHyer, SteveTenkumo, TaichiHylander, Lars D.Tenore, GianlucaHyndman, BrendonTenorio Lopes, LuanaHyndman, KathrynTentolouris, NikolaosHyunbin, JoTeo, AndrewIacobellis, GianlucaTeo, Wei-PengIacono, DiegoTeoh, Ai NiIacoviello, DanielaTeovanović, PredragIani, CristinaTepper, Beverly J.Iannetta, DaniloTeques, PedroIano, YuzoTerada, SeishiIatrakis, GeorgiosTeragawa, HirokiIatsenko, IgorTeramoto, TadahisaIbabe Erostarbe, IzascunTerčelj, MarjetaIbáñez Alfonso, Joaquín A.Tereshchenko, SergeyIbáñez Escribano, AlexandraTeresiński, GrzegorzIbáñez, Sergio JoseTerlizzi, MichelaIbáñez-Costa, AlejandroTermorshuizen, FabianIbáñez-Forés, ValeriaTerranegra, AnnalisaIbáñez-Vera, Alfonso JavierTerrazas, Luis I.Ibarluzea, JesusTerroso, MiguelIbarra Espinosa, SergioTerry, DanielIbarra-Mejia, GabrielTerzaghi, WilliamIboi, Enahoro A.Tesfaye, Wubshet H.Iborra Cuéllar, AlejandroTesfaye, Yihenew AlemuIbrahim, Maria SalemTesler, RikiIbrahim, Mohamed BabikerTeslya, NikolayIbrahim, Sherif AbdelazizTess, Beatriz HelenaIbrahim, Sulaiman SadiTesta, AlexanderIbuka, YokoTesta, MarioIchibori, YasuhiroTesta, UgoIchihashi, MasamitsuTestoni, DanielaIchinose, ToshiakiTestoni, InesIdda, Maria LauraTetel, MarcIde, KazukiTeti, LucaIdler, Ellen L.Teubner, KatrinIdowu Ajayi, AnthonyTeufel-Shone, Nicolette I.Ieong, Hada FonghaTeufl, LukasIerapetritis, DimitrisTexeira de Siqueira Filha, NoemiaIerardi, ElenaTeymoori, AliIeva, MarcoThai, PhongIfelebuegu, Augustine OsamorThai, YvonneIgartua, Juan JoséThakur, TanveeIglesias Gallego, DamiánThalamuthu, AnbupalamIglesias López, Maria TeresaThaler, ThomasIglesias-Linares, AlejandroThalheim, TorstenIglseder, BernhardThalmann, MatthiasIgnatius, JoshuaTham, RachelIgnatowski, GrzegorzTham, Su GwanIgumnov, SergeiThandra, Krishna ChaitanyaIhasz, FerencThane, PatIhle, AndreasThangathurai, DuraiyahIimura, ShuheiThanigaimani, ShivshankarIjaz, Muhammad FazalThanikachalam, KannanIkeda, ErikaThapa, JananiIkegami, KazunoriThapa, RabaIkei, HarumiThayabaranathan, TharshanahIkerd, John E.Theilmann, FlorianIl’yasova, DoraThejll, PeterIldikó, TarTheobald, MaryanneIlesanmi-Oyelere, Bolaji LilianTheodoropoulos, ChristosIlewicz, GrzegorzTheorell, TöresIlia, StavroulaThi Thu Nguyen, ThaoIlias, IoannisThiago, Carvalho De SousaIlic, NebojsaThiamwong, LaddaIlieva, MirolybaThiel, AnsgarIliyasu, AbdullahThiele, CarstenIllescas, JavierThielmann, IsabelIlmonen, PauliinaThijsen, AmandaIlomaki, JukkaThikkurissy, SaratIlyas, MohammedThill, Jean-ClaudeIlyinskii, Petr O.Thiruvengadam, MuthuIm, HyojinThiyagarajan, Jothi SaravananIm, JongkwonThodelius, CharlottaImam, TasadduqThom, BetsyImamura, HaruhikoThoma, Colleen A.Imbery, Terence A.Thomas, Casey J.Imholt, ChristianThomas, CateImmacolata, Di NapoliThomas, CharalamposImmler, UlliThomas, ChristianImperatori, ClaudioThomas, Donald B.Improta, GiovanniThomas, EwanImre, KorneliaThomas, JacquelineInada, Haruhiko (Hal)Thomas, MargaretInam, Muhammad AliThomas, NatalieInamo, YasujiThomas, Pauline A.Inan-Eroglu, ElifThomas, RhiannaInbadas, HamiltonThomas, RogerInchingolo, FrancescoThomas, SethIncollingo Rodriguez, AngelaThomas, Stephen B.Indiana, Maria LuisaThomas, Suzanne D.Indino, CristianThomée, SaraIndirli, MaurizioThompson, Ashley E.Indraccolo, UgoThompson, BronwynInfante, PauloThompson, Elizabeth C.Inglés, CándidoThompson, JeffIngraffea, AnthonyThompson, Lisa MarieIngram, Katherine H.Thompson, Miles D.Ingram, LesleyThompson, PeterIngrassia, MarziaThompson, Rebecca R.Ingrassia, MassimoThomsen, ChristaIngusci, EmmanuelaThomson, JessicaInkinen, TommiThomson, Neil C.Inmaculada Diaz, AznarThomson, RossInnerd, AlisonThongprayoon, CharatInniss, Enos C.Thoonen, KarlijnInomata, MinehikoThorne, ClaireInoue, JunThornley, SimonInoue, KazuyaThornley, TraceyInoue, Ken-ichiroThornton, Alex ReneeInoue, RyoThornton, CatherineIntini, PaoloThornton, Jeffrey A.Introne, Joshua E.Thornton, Jim G.Inturri, RosannaThorpe, ClareInuso, GiuseppinaThorpe, DavidInzunza-González, EverardoThorsteinsson, EinarIoannidou, AlexandraThredgold, LeighIoannou, LeonidasThunus, SophieIoannou, MariaThürmann, LoreenIonel, IoanaThurston, MirandaIonescu, Constantin AurelianThurston, Sally W.Ionta, SilvioThuzar, MoeIorga, GabrielaThyer, BruceIorga, MagdalenaThygerson, StevenIorio, Angelo DiTian, ChangxuIorio, Ronald M.Tian, JinfangIorio, VincenzoTian, Kun VivianaIourov, Ivan Y.Tian, WeiIovino, PaolaTian, XuIqbal, AsifTian, YixiangIqbal, Hafiz M. N.Tian, Yong-QiangIqbal, M. AnwarTiano, JustinIrannezhad, MasoudTiberius, VictorIranzo, BegoñaTiboni, Gian MarioIranzo-Cortés, José EnriqueTicinesi, AndreaIreton-Jones, CarolTiefenbacher, JohnIrga, PeterTienari, PenttiIrimiás, AnnaTietjens, MaikeIrisawa, HiroshiTiffon, CélineIrish, Leah A.Tigas, SteliosIriuchishima, HironoTigbe, WilliamIrizar, AmaiaTigrini, AndreaIronside, MariaTiidus, PeterIrurtia, AlfredoTiisanoja, AnttiIrvin, VeronicaTill, BenediktIrvine, AnnieTilley, ElizabethIrvine, IanTilli, MartaIrvine, LeslieTimilsina, ArbindraIrwin, JayTimler, AmandaIrwin, RachelTimman, ReinierIrzyniec, TomaszTimmis, JeremyIsaacs, AnnaTimofeyev, YuriyIsaacson, MichalTinajero, CarolinaIsaifan, Rima JamalTinazzi, IlariaIsaksson, UlfȚîncu, RoxanaIseppi, LucaTindle, HilaryIsernia, SaraTing, Hsien-WeiIsezaki, TakashiTinggi, UjangI-Shiang, TzengTinney, GlendaIshida, MariTintoré, MireiaIshigami, JunichiTippett, VivienneIshigami, TomoakiTipre, MeghanIshiguro, YoshioTiseo, CarloIshihara, KengoTisseyre, BrunoIshihara, ToruTitz, AlexandraIshii, MasakazuTitze, SylviaIshikawa, KiyotakeTiwari, HarishIshikawa, TetsuoTiziana, ManciniIshimoto, YasukoTjakkes, Geerten-HasIshiyama, HiroyukiTjernström, FredrikIsidori, EmanueleTkavc, RokIsigonis, PanagiotisTlapa-Mendoza, DiegoIşildar, ArdaTluczek, AudreyIskandar, IrwanTo, Wai MingIslam, FarahTobis, SlawomirIslam, Md SaifulTochkov, KirilIslam, Md ZohorulToczylowska-Maminska, RenataIslam, Mohammad MirazulTodaka, DaisukeIslam, Mohammad SaidulTodeschini, SaraIslam, SarifulToelle, BrettIslam, SorifulTognola, GabriellaIsmael, SaifudeenTohma, KentaroIsmail, SandaTohyama, JunIsobe, TomohikoToiu, AncaIsoda, NorikazuTokarek, TomaszIsola, DanielaTokarz-Deptuła, BeataIsola, GaetanoTokatlidis, IoannisIssurin, Vladimir B.Tokonami, ShinjiIstván, HajnalToledo-Neira, CarlaItatani, TomoyaTolhurst, EdwardIto, NaokiTolkou, AthanasiaIto, SusumuTollefson, MichelleIto, TomokoTolo, SilviaItrić, KatarinaToma, MilanItsukushima, ReiToma, PierluigiIttenbach, Richard F.Toma, Radu BogdanIughetti, LorenzoTomal, MateuszIvan Ramírez, JorgeTomaselli, VeneraIvandic, IvanaTomaszek, KatarzynaIvanitskaya, LanaTomaszewska, EwaIvannikov, AlexanderTomaz, Simone A.Ivanov, Luba LouiseTomba, ElenaIvanova, BojidarkaTomczak, AndrzejIvanová, KateřinaTomczak, MaciejIvanova, Olga E.Tomczak, MichałIvanovic, Stefan S.Tomczyk, ŁukaszIvanyna, MaksymTomé Pires, CatarinaIvascu, LarisaTomé, EduardoIversen, NielsTomioka, MichiyoIves, NicoleTomita, Machiko R.Ivica, AnjaTomiyama, ShingoIvona, AntoniettaTomkin, Gerald H.Iwai-Shimada, MiyukiTomlinson, DavidIwata, KentaroTomlinson, OwenIwata, TakashiTommasino, FrancescoIwu, ChinweTomnay, Jane E.Iyer, HariTomofuji, TakaakiIzaguirre, AinhoaTomohito, SanoIzawa, Kazuhiro P.Tomsone, SigneIzenstark, DinaTon, Shan-ShinIzhak, SchnellTonacci, AlessandroIzumi, YoshihiroTondelli, SimonaIzydorczyk, BernardettaTondi, DonatellaIzydorczyk, GrzegorzTondo, MireiaIzzetti, RossanaTonello, Kelly CristinaIzzo, RiccardoTonetti, LorenzoJ. Palmer, DebraTong, RuipengJ. Pen, JoeriTong, Yat-ChingJaakkola, SamuliTong, YongshengJabba, DaladierToniolo, Antonio Q.Jabłońska, BeataTonni, GabrieleJabłońska, JoannaTonolo, GiancarloJabłońska-Trypuć, AgataToomey, AnneJablonski, NinaToovey, RachelJablonskienė, ValerijaTop, SonerJabreel, MohammedTopa, GabrielaJacevic, VesnaTopic, MartinaJachens, LizaToporski, JacekJachimowski, KaylaToppila-Salmi, SannaJacinto, Alessandro FerrariTorassa, DanielJack, AnsonTorbaghan, Mehran EskandariJackman, Joshua A.Tordeux, AntoineJackowska, MartaTorero, Jose LuisJackson Woo, Chit ShingToribio-Mateas, MiguelJackson, AlunToriello, FilippoJackson, Alun C.Torio Lopez, SusanaJackson, CathToriola, Abel L.Jackson, Dylan B.Torisawa, YusukeJackson, Kathy MerlockToriyama, MichinoriJackson, ReneeTornero-Quiño, InmaculadaJackson, Shelly L.Tornese, GianlucaJacob, Chandni MariaTorng, Pao-LingJacob, NoamTörnroos, KaisaJacobo A., Rubio-AriasToro-Ibacache, VivianaJacobs, DavidTörök, ÁronJacobs, David E.Toronto, Coleen E.Jacobs, GraemeTorrado, EgídioJacobs, StephenTorrado, PriscilaJacobsen, RamuneTorrance, Andrew B.Jacobson, Melanie H.Torregrosa, Javier FerrerJacobson, Moriah L.Torres Lacomba, MaríaJacobson, Willow S.Torres Vaamonde, José EnriqueJacques, PiazzolaTorres, Arnau MirJadavji, NafisaTorres, Francoise Venezia ContrerasJadavji, Nafisa M.Torres, Javier RodríguezJaeschke, Rafał R.Torres, João PauloJafari, Najmeh (Nancy)Torres, RosaJager, AlessandraTorres, SergioJäger, GeorgTorres, SusanJager, HenrietteTorres-Castro, RodrigoJager, Martine JTorres-Collado, LauraJager, NicolasTorres-Lagares, DanielJägerbrand, Annika K.Torres-Luque, GemaJagoda, AgnieszkaTorres-Mejía, GabrielaJagoe, CarolineTorres-Ortega, SaúlJahanbin, AminhosseinTorres-Ramírez, EduardoJahandideh, ForoughTorres-Rodríguez, MiguelJahangiri, SanazTorres-Sánchez, IreneJahanshahi, AsgharTorres-Sanchez, SoniaJahng, Kyung EunTorres-Sangiao, EvaJahnke, MathiasTorres-Valenzuela, Laura SofíaJahrami, HaithamTorrico, DamirJaimes, Javier A.Torrieri, FrancescaJain, AnshikaTorti, ValeriaJain, PinkyToru, IchisekiJaja, Ishmael FestusToruńska-Sitarz, AnnaJajtner, AdamToselli, StefaniaJakica, NebojsaTosetto, BarbaraJakiel, GrzegorzTosi, Maria ChiaraJakimovski, DejanTositti, LauraJakóbik - Kolon, AgataToth, Adam J.Jakobsson, KristinaToth, GergelyJakovljevic, MihajloTottori, NobuakiJakovljevic, Mihajlo B.Toubekis, ArgyrisJakovljevic, VladimirToubiana, MylèneJakub, KrejciToueir, NadaJakubiak, Brittany K.Toumi, MarzenaJakubowski, KarenTour, GregoryJakubus, MonikaTovilla-Zárate, Carlos AlfonsoJalal, ZahraTownley, LloydJalali, MarjanTownsend, EleanorJalali, NedaTownsend, JulieJalava, KatriToyinbo, OluyemiJalilov, Shokhrukh-MirzoToyoda, HidemiJalongo, Mary RenckToyokawa, TatsuyaJámbor, AttilaToyotome, TakahitoJames, JoeToze, MichaelJames, JoelTozer, CarlyJames, LachlanTrabace, LuigiaJames, LindaTrabold, NicoleJames, LoisTrach, RomanJames, RichardTraczyk, IwonaJames, SpencerTrafiałek, JoannaJames, ZoëTraini, ToninoJamshed, HumairaTrainor, PatrickJamshidi-Naeini, YasamanTrajkcovic, NebojsaJan, Chyan-LongTran, AlvinJan, Ihsan UllahTran, BachJan, MuhammadTran, BenJan, Sana UllahTran, Binh Q.Jancloes, MichelTran, HuyJanczewski, ColleenTranchant, CaroleJanda, KatarzynaTranfo, GiovannaJandarov, RomanTrangenstein, PamelaJandl, RobertTrauer, JamesJanecki, MarcinTravassos, BrunoJanecki, RyszardTravis, CharlesJaneczko, EmiliaTraynor, KirstenJanet, JanetTreasure, TomJang, AeleeTrecroci, AthosJang, CholsoonTreff, GunnarJang, Dae-heungTregua, MarcoJang, Hye-RyenTrejos, BernardoJang, InsilTrematerra, AmeliaJang, SiwonTremolada, MartaJang, Sou HyunTrenado, CarlosJang, SunginTrendelenburg, MartenJang, Tae-WonTrepanier, AngelaJangampalli Adi, PradeepkiranTrescastro, Eva MariaJanganan, ThamaraiTreslova, MarieJanhäll, SaraTrestini, MarcJanicki, GrzegorzTrezona, AnitaJanicki, WojciechTriantafyllidis, StavrosJanik, MiroslawTricarico, LucaJanikowska, GrażynaTricase, CaterinaJaniszewska, MariolaTrifan, AlinaJanjetovic, ZoricaTrifuoggi, MarcoJankauskiené, AugustinaTrigeassou, Jean-ClaudeJankauskienė, RasaTrigo, JesúsJankovic, MomciloTrigueros, RubénJankovic, NicoleTrihinas, DemetrisJankowiak, WilliamTrip, SimonaJankowicz-Szymańska, AgnieszkaTripathi, Kumud MalikaJankowska, AnnaTripathy, RohitJankowska, MartaTriplett, R. GilbertJankowska-Mihułowicz, MarzenaTripodi, DomenicoJankowski, MateuszTripolt, NorbertJanmaimool, PiyapongTripp, LianneJanovec, MichalTrisolino, GiovanniJanovska, DagmarTritto, AngelaJanowski, AndrzejTriunfo, StefaniaJansen, CarelTrnka, Hans-JoergJansen, Erica C.Trögl, JosefJansen, Lisa T.Troiano, GiuseppeJanssen, AnnaTroiano, Richard P.Janssen, JanineTroisi, GinaJanssens, ThomasTrojanek, RadoslawJansson, MäritTrojanowicz, BoguszJanuario, Leticia BergaminTrojanowska, MonikaJanus, EdwardTrommlerová, Sofia KarinaJanuszkiewicz-Lewandowska, DanutaTroost, JonathanJanzen, BonnieTropmann-Frick, MarinaJape, GayatriTroutman-Jordan, MeredithJaradat, NidalTrovato, Maria RosaJaramillo-Alcázar, AngelTroyer, JohnJardini, Maria Aparecida NevesTroyo Diéguez, EnriqueJarl, JohanTrude, AngelaJaroslav, VrchotaTrudel, XavierJarosova, DarjaTrue, LarissaJarosz-Krzemińska, ElżbietaTruedsson, AnnaJarrott, ShannonTruijen, StevenJärvelä, SallyTrujillo De Santiago, GrisselJasienska, GrazynaTrujillo-Torres, Juan M.Jasińska, ElżbietaTruman, EmilyJasińska, IzabelaTruman, PennyJasińska-Stroschein, MagdalenaTrumello, CarmenJassal, Mandeep SinghTruszczyńska, AleksandraJastrzębska, AgnieszkaTrybek, PaulinaJatužis, DaliusTryggestad, JeanieJavanbakht, ArashTrząskowska, MonikaJavanroodi, KavanTsagkanos, AthanasiosJavanshour, FaridTsagkaris, AristeidisJaved, ArshadTsai, Chen-ChiJaved, FawadTsai, Cheng-HungJavid, Muhammad AshrafTsai, Fu-ShengJavier Jiménez-Jiménez, FelixTsai, JamesJavier Rodríguez Hernández, HumbertoTsai, Kuang-ChungJawinski, PhilippeTsai, Kun-LingJaworowska, AgnieszkaTsai, Meng-CheJayakumar, AnjaliTsai, Meng-ShanJayanthi, SankarTsai, Ming-JuJayaraman, ChandrasekaranTsai, RebeccaJayasundara, DheeshanaTsai, Rong-KungJayatissa, RenukaTsai, ShihfengJayawardene, Wasantha P.Tsai, Wang-ChinJay-Gerin, Jean-PaulTsai, Wei-LunJean, KevinTsai, Wen-tienJeannot, EmilienTsai, Yi-JuJechow, AndreasTsakiridis, IoannisJeckell, Aaron SloneTsampras, NikolaosJedrzejczak, WiktorTsapanou, AngelikiJędrzejewski, StanisławTsatsakis, AristidisJee, Hyun SeokTsatsaris, AndreasJee, Yong-SeokTse, MimiJeerapan, ItthiponTselios, VassilisJefferis, EricTseng, Chie-ChienJefferson, CurtisTseng, Chun-ChiehJeison, DavidTseng, Ming-LangJekova, IrenaTseng, Yi-JuJelks, Na’Taki OsborneTseng, Yu-ChuanJelleyman, CharlotteTsergouli, KaterinaJelski, WojciechTsermpini, Evangelia EiriniJenkins, DanielTsiakas, IliasJennings, GeorgeTsikouras, PanagiotisJensen, ElaineTsilibary, Effie-Photini C.Jensen, Kai OliverTsilika, KyriakiJensen, KimTsilimigkas, GeorgiosJensen, OlafTsimtsiou, ZoiJensen, OlgaTsionas, MikeJensen, Per AnkerTsironis, Giorgos P.Jensen, RogerTso, For YueJenson, JenniferTsoi, HoJeon, Eui-ChanTsou, Meng-TingJeon, Jin-hunTsourdi, ElenaJeon, KwonchanTsuboi, AkitoJeong, BokTsuchida, KunihiroJeong, Byung YongTsuchida, SachioJeong, JaewookTsuda, HitoshiJeong, Keun-YeongTsujimura, SoheiJeong, SeungwonTsukahara, TamotsuJeong, Seung-WooTsukamoto, HayatoJeong, Soo JinTsukamoto, TetsuoJeong, Yoo MiTsukinoki, RumiJeremy, Teoh Yuen ChunTsur, NogaJerger, KristinTsutsumi, SeijiJerome, GeraldTsydenova, NinaJeruszka-Bielak, MartaTu, Hung-PinJerzak, Malgorzata MariaTu, Min-chengJerzy, MosiewiczTually, SelinaJesdale, WilliamTuchman, AnnaJesenak, MilosTucker, PhilipJesulola, EmmanuelTudor, Mihaela-AlexandraJesús Gustavo, Ponce GonzálezTuduri, LudovicJesus, Tiago SilvaTuells, JoséJeżewska-Zychowicz, MarzenaTufarelli, VincenzoJeż-Walkowiak, JoannaTullius Scotti, MarcusJhwueng, Dwueng-ChwuanTully, LucyJi, GuozhaoTulunoglu, IbrahimJi, JohnTuluri, FrancisJi, LexiangTulve, NicolleJi, ShaofeiTumedei, MargheritaJi, XiaopengTummino, Maria LauraJia, BaoTumosa, NinaJia, CharlesTun, Mya Myat NgweJia, FanliTünde, TarrJia, JunTunér, JanJia, LiTung, Bui ThanhJia, LihongTung, Tao-HsinJia, YanweiTünnermann, JanJia, YongTuno, NobukoJia, Yunyan AndreaTunstall, Brendan J.Jian, Bo-LinTunur, TumayJian, XingTural, ÜmitJiang, ChengTurbow, DavidJiang, Crystal L.Turiel, MaurizioJiang, FanTurjamaa, RiittaJiang, GuofengTurkkahraman, Muhammet HakanJiang, HengTurkmen, MutluJiang, HongTurnbull, PaulineJiang, HuanhuanTurner, Arlener D.Jiang, MeilanTurner, BrianJiang, QuanbaoTurner, JonathanJiang, WanlinTurner, Mason SpainJiang, WenhuiTurpin, NicolasJiang, XinyinTurpin, RodmanJiang, XiqianTurri, MaraJiang, YuanyingTurturice, Benjamin ArthurJiang, YuemingTutak, MagdalenaJiannine, LiaTutkuviene, JaninaJikuzono, TomooTutueva, AleksandraJillella, DineshTuuri, GeorgiannaJim, LukTuvdendorj, DemidmaaJiménez Ballesta, RaimundoTvedebrink, Tenna Doktor OlsenJimenez, DianaTwersky, SylviaJimenez, EmilioTwomey, MichèleJiménez, José-MaríaTyagi, MuditJiménez, Julio DíazTyburski, ErnestJiménez, LucíaTychmanowicz, AnnaJimenez, RicardoTye, MichelleJiménez, TerebelTyler, HannahJimenez-Bescos, CarlosTyler, KimberlyJiménez-Jiménez, Félix JavierTyrologou, PavlosJiménez-Marín, GloriaTyson, JulianJiménez-Morales, MònikaTytła, MalwinaJimenez-Olmedo, Jose M.Tzallas, AlexandrosJimenez-Rejano, José-JesúsTzanev, PeterJimenez-Rondan, FelixTzeng, Huey-MingJiménez-Rubio, SergioTzeng, Shiang-JongJimoh, FlorenceTzeng, Shu-LingJin, AndrewTzeremes, Panayiotis G.Jin, DiTzivian, LilianaJin, Hyun JoungTzortzi, Anna S.Jin, JianjunTzovenis, IoannisJin, Jun-O.Uba, KatrinJin, Ki NamUccheddu, StefaniaJin, LiUchida, SatoshiJin, LishengUchino, HarutoJin, LiYaUchmanowicz, IzabellaJin, XueyingUcieklak-Jeż, PaulinaJin, ZhuqiuUdagawa, JunJindrichovska, IrenaUdasin, IrisJing, RanUddin, EktearJinnouchi, HiroyukiUddin, MajbahJiricka-Pürrer, AlexandraUdoh, AritJnawali, KamalUebanso, TakashiJo, AraUeberschär, OlafJo, Young MinUeda, KeijiJoachimiak-Lechman, KatarzynaUehara, IwaoJoão, Neves-AmadoUeno, ShuichiJob, KathleenUgargol, Allen P.Jobst, MarkusUgarte, SergioJochem, CarmenUgon, AdrienJochymczyk-Woźniak, KatarzynaUhl, EberhardJoda, TimUhlenhut, FrankJoddrell, PhilUink, BepJoe Lin, ChiuhsiangUitto, AnnaJohannssen, ArneUkleja-Sokołowska, NataliaJohansson, EdvardUkpokodu, PeterJohir, MohammedUl Haque, AdnanJohn, Rosalind M.Ul-Abdin, ZainJohns, LouiseUlaganathan, SekarJohnsen, SarahUlibarri Ochoa, AinhoaJohnson, Adelaide C.Ulibarri, GerardJohnson, AlisaUllah, Md AshikJohnson, AmyUllah, ShahidJohnson, Boris GabrielUllah, SharifJohnson, Dylan M.Ullah, ZahidJohnson, GarethUlloa Cerna, AlvaroJohnson, JayUllsten, AlexandraJohnson, JerracoUlrich, SebastianJohnson, JoshuaUm, NamilJohnson, Kelly E.Umanzor, ScheryJohnson, Lance A.Umayahara, YasutakaJohnson, Mark R.Umbelino, JorgeJohnson, NicholasUmeda, MakiJohnson, OliviaUmeda, MasatakaJohnson, OwenUmeokafor, NnedinmaJohnson, PaulUmiastowska, DanutaJohnson, Teresa W.Umpaichitra, VatcharapanJohnson, William F.Unchwaniwala, NuruddinJohnson-Jennings, MichelleUnderdahl, LouiseJohnston, CarolUngaro, CarmineJohnston, JamesUngerman, OtakarJollans, LeeUnni, Elizabeth J.Jolley, DavidUnsal, OmerJona, GyorgyUnson, ChristineJonathan, ElliottUnsworth, Carolyn A.Jonek-Kowalska, IzabelaUnterrainer, JosefJones, AubreyUpendram, SreedharJones, CarysUpreti, GirishJones, CherylUpshur, Carole C.Jones, Christopher S.Ur Rehman, Muhammad AtiqJones, EsylltUrada, LianneJones, Jeffery A.Uraipong, GibJones, John K.Urata, ShuzoJones, John W.Urban, SusanneJones, Kyle BradfordUrbanek, ArkadiuszJones, LaurenceUrbaniak, BoguslawaJones, MargaretUrbaniak, MaciejJones, MarjorieUrbanik, MarekJones, Michael L.Urbanos Garrido, Rosa M.Jones, MonicaUrbini, FlavioJones, Penelope J.Urchaga, José D.Jones, RachaelUrhahne, DetlefJones, RachelUribe-Toril, JuanJones, Rodney P.Urowitz, SaraJones, RogerUrra, José MiguelJones, RupertUrrestarazu, ElenaJones, SianaUrrutia, Manuel LeonJones, ThomasUrsino, MorenoJones, TiffanyUsabiaga, CarlosJones-Diette, Julie S.Uşak, MuhammetJones-Eversley, SharonUsami, Davide ShingoJong, ChianjuUsami, SatoshiJong, StephanieUseche, Sergio A.Jonker, CaraUsman, MuhammadJonnalagadda, SreekanthUspensky, IgorJonsson, FridaUstinaviciene, RūtaJonsson, OskarUsuelli, FedericoJonvik, Kristin LundanesUsui, AkihikoJoo Nagata, JorgeUtriainen, RoniJorat, EhsanUttumchandani, Shakira KaknaniJordá, VanesaUusi-Rasi, KirstiJordaan, HenryUversky, VladimirJordakieva, GalatejaUwayezu, ErnestJordan, CatherineUysal, UtkuJordan, HannahVaarno, JenniJordan, SusanneVacante, MarcoJorgensen, Jennifer JohnsonVaccari, MentoreJørgensen, LarsVaccaro Witt, Gustavo FabiánJörns, AnneVaccaro, Joan A.Joronen, KatjaVach, KirstinJosé María, Biedma FerrerVácha, RadimJosé Tauler Riera, PedroVaezghasemi, MasoudJose, MarcoVafiadaki, ElizabethJose, Sanchez RamosVafidis, DimitrisJose, Silva-NunesVagni, MoniaJose, ThulaseeVagnoni, EmidiaJoshi, GauravVahdaninia, MariamJoshi, PrathibhaVahidi, HosseinJoshi, Pushpa RajVaičiūnienė, RūtaJoshi, SameerVairo, TomasoJoshita, SatoruVäisänen, HeiniJošić, HrvojeVäisänen, PerttiJouko, TuomistoVaismoradi, MojtabaJoyce, Daniel S.Vakhania, NodariJoyce, MalcolmValafar, HomayounJoyce, Michael A.Valantine, IrenaJoyce, StellaValasik, MatthewJozaghi, AliValdemorosos San Emeterio, María De Los Á ngeles Jozwiak, MathieuValdés, HugoJu, Man KiValdez Juárez, Luis EnriqueJu, XiangyangValdez, Alan-MiguelJu, YangValdivia Salas, Maria SonsolesJu, Yeong JunValdivia-Moral, PedroJu, YiyiVale, Carlos Alberto Garcia DoJuan, WenyenVale, JoséJuárez-Vela, RaúlValencia, FelipeJudd, BruceValencia, FranciscoJudd, JenniValente, AntonioJuhász, TamásValente, Nicola AlbertoJukić Peladić, NikolinaValente, Tânia Cristina De OliveiraJulian-Rochina, IvanValentine Okoro, OseweubaJuliá-Sanchis, RocíoValentine, GregoryJulien, Pierre Y.Valentukeviciene, MarinaJuliev, MukhiddinValenzano, AnnaJumadi, J.Valenzuela, Alfonso ValeroJun, ByungMoonValenzuela, HectorJun, ChihValenzuela, MarcelaJun, Se-RanValenzuela, Pedro L.Jun, WoochunValenzuela, RodrigoJung, Bock-YoungValera-CAlero, Juan AntonioJung, ChaurenValera-Gran, DesiréeJung, Chien-ChengValeriani, FedericaJung, ChristianeValerio, AdrianoJung, Hyo SunValero, Alberto FerrizJung, JaehunValero, VictorJung, JaeyoungValero-Romero, María JoséJung, Jin-HwaValhondo, CristinaJung, JiyeonValicherla, Guru RaghavendraJung, Se YongValickienė, Rasa PilkauskaitėJung, Se-YounValipoor, ShabbooJung, SongeeValipour, MohammadJung, SungmokValkonen, Tarmo JohannesJung, Yuh-SeogVallamsundar, SuriyaJung-Choi, KyungheeVallarino, MartineJunge, CarolineVallata, AmandineJunge, KristinVallée, AlexandreJunger, JanVallée, EmilieJunglee, Naushad AliVallely, LisaJunichi, KitanakaVallentin-Holbech, LotteJunne, StefanValles, Soraya L.Jurado Castro, José ManuelVallés-Giménez, JaimeJurado De Los Santos, PedroVallesi, ShannenJuráň, StanislavVallini, GiovanniJuranek, JudytaValls Martínez, María Del CarmenJurczak, AnnaValls-Fonayet, FrancescJureviciene, DaivaVallvé-Juanico, JúliaJurjans, KristapsValm, Alex M.Jurkovic Mlakar, SimonaValmaseda-Castellõn, EduardJurkowska, HalinaValtierra-Rodriguez, MartinJurman, GiuseppeValvani, RachnaJurzik, LarsValverde, TeresaJusienė, RomaVan ’t Hul, AlexJuszczuk, PrzemysławVan Amsterdam, Jan G. C.Juvinyà, DolorsVan Asselt, Antoinette D. I.Juzeniene, AstaVan Buiten, Charlene B.Jůzl, MiroslavVan Buul, VincentKaabar, MohammedVan Cleeff, MaartenKaali, SeyramVan Dam, PieterKaba, EvridikiVan De Heijning, Bert J. M.Kabir, AbuzarVan De Kolk, IlonaKabir, GolamVan De Ven, PeterKabir, RussellVan Delden, J. J. M.Kabisch, StefanVan Den Berg, PaulineKaczmarek, AgnieszkaVan Den Bergh, HubertKaczmarek, ChristianVan Den Boomen, MartineKaczmarek, KrzysztofVan Den Ende, JefKaczor, GrzegorzVan Den Helder, JantineKaczor-Urnanowicz, Karolina ElzbietaVan Den Heuvel, EllenKaczyńska, KatarzynaVan Den Honert, Robin C.Kaczynski, AndrewVan den Noort, MauritsKadam, UmeshVan Den Noortgate, WimKádár, BálintVan Der Bent, M. LeontienKaden, ReneVan Der Laarse, Willem J.Kadotani, HiroshiVan Der Maas, Mark R.Kaesmann, LukasVan Der Molen, HenkKafel, AlinaVan Der Nest, Magriet A.Kafetsios, KonstantinosVan Der Pas, SuzanKaffashi, SaraVan Der Schueren, BartKagawa, MasaharuVan Der Tol, ArjanKageyama, HakutoVan Der Vaart, LeoniKagot, VictorVan Der Velde, Nathalie V.Kahkonen, OutiVan Dijk, Meine PieterKahl, Jonathan D. W.Van Duinen, Alex J.Kahler, DavidVan Eijk, Anna M.Kahn, Geoffrey D.Van Etten, EddieKahn, Linda G.Van Gasselt, StephanKahsay, ZnabuVan Gerwen, MaaikeKahu, JaanusVan Gestel, RafKai Heinrich, KaiVan Grieken, AmyKai, Michiakivan Hoof, JoostKaifie, AndreaVan Hoye, AurélieKain, VasundharaVan Kempen, LuukKaissi, Ali AlVan Lankveld, WimKai-wai, Willy HuangVan Leeuwen, David A.Kajamies, AnuVan Muiswinkel, Willem B.Kajdy, AnnaVan Overmeire, RoelKajiwara, YusukeVan Praag, LoreKakinoki, YasuakiVan Rijn-Van Gelderen, LoesKaklamanou, DaphneVan Rooy, LouisKakumanu, Madhavi L.Van Santen, VickyKal, ElmarVan Selms, Maurits K. A.Kalabokas, PavlosVan Son, NguyenKalafat, ErkanVan Trijffel, EmielKalalahti, MiraVan Wagoner, Nicholas J.Kalantidou, EleniVan Wee, BertKalasekar, SharanyaVan Winkle, LonKalbarczyk, RobertVan Zalk, NejraKale, AbhijitVan Zanten, MalouKalenik, MarekVAN ZWIETEN, Koos JaapKaleta, DorotaVance, David E.Kalimeri, KrystalliaVandenberg, AnnKalimeri, KyriakiVandenplas, YvanKalin, Robert MVanderloo, Leigh M.Kalinich, JohnVanderMolen, JuliaKalisz, StanisławVandermorris, AshleyKaliszewski, MironVanderpas, JeanKalita, MichałVanegas, Jairo LópezKalkstein, AdamVanHeuvelen, JaneKallas, ZeinVankova, HanaKalligeros, Stamatis SpyridonVanlaar, WardKallioniemi, ElisaVanlandingham, DanaKallu, K. WolfgangVanscheeuwijck, PatrickKalman, DouglasVänskä, SimopekkaKalnova, SvetlanaVantas, KonstantinosKalogeropoulos, Andreas P.Vant-Hull, BrianKalogiannakis, MichailVanWormer, ElizabethKalsi-Ryan, SukhvinderVaquero-Abellán, ManuelKaltsatou, AntoniaVaquero-Cristobal, RaquelKalus, AlicjaVaradinov, Maria JoséKałuża, TomaszVarando, GherardoKam, Lynn CialdellaVardasca, RicardoKamaz, MohanadVarese, EricaKamelska-Sadowska, Anna M.Varesio, CostanzaKamenidou, IreneVargas Jentzsch, PaulKamigaki, TaroVargas Terrones, MarinaKamińska, Anna MałgorzataVargas, Alessandro N.Kamińska, Joanna A.Vargas, Carolina MachucaKaminska, MartaVargas-Garcia, Elisa JoanKamiński, TomaszVargas-García, María Del CarmenKamis, ArnoldVarghese, MathewsKamocki, AndrzejVarju, CeciliaKamolz, Lars P.Varma, ParulKampas, AthanasiosVarrela, JuhaKampmann, UllaVarrica, DanielaKampouras, AsteriosVasconcellos, Klaus L. P.Kan, JinjunVasconcelos, Manuel PestanaKana, AntoninVasconcelos, MartaKanakis, IoannisVasconcelos, TeresaKanamori, MasaoVasefi, MaryamKanbayashi, ToruVashist, AseemKanczler, Janos M.Vashist, SandeepKanda, NaokoVashist, Sandeep KumarKandala, Ngianga-bakwinVasickova, JanaKandasamy, RamVasilateanu, AndreiKandeel, MahmoudVasile, MonicaKandpal, ManojVasilescu, JeniKane, EleanorVasilev, MiroslavKanekar, AmarVasiliadou, RafaelaKang, Augustine W.Vasilieva, ElenaKang, Choong-MinVasilyev, AndreyKang, Dae-KiVasnev, AndreyKang, Gu EonVásquez-Velásquez, DavidKang, Hee-TaikVassalle, CristinaKang, Hyun-KyungVassallo, RobertoKang, Jeong-HyunVassanelli, AuroraKang, Jeon-YoungVassilenko, ValentinaKang, Ju-HeeVasta, RosarioKang, JungsukVasto, SonyaKang, KyonghwaVasudevan, AbhinavKang, KyuVasudevan, SreelakshmiKang, SanghoonVatanpour, NahidKang, Seong CheolVatcheva, KristinaKang, Seong-KyuVatolkina, NataliaKang, Seung-WanVaucher, PaulKang, SunghwunVaughan, AnnaliciaKang, YoungcheolVaughan, Christopher G.Kanginakudru, SriramanaVaughan, RobertKania, AnetaVaughn, AllisonKanikowska, DominikaVavilis, DimitriosKankaanpää, EilaVavrek, RomanKanna, KurunthachalamVaz De Carvalho, CarlosKano, NaokiVaz, Irene PinaKantamaneni, KomaliVaze, Nachiket D.Kantanista, AdamVaz-Moreira, IvoneKantono, KevinVázquez Burguete, Jose LuisKantor, JiříVázquez, Carlos RepresasKanwar, AnubhavVázquez, Fernando L.Kanwar, PoojaVazquez-Alaniz, FernandoKanya, LucyVázquez-Campos, XabierKanzow, PhilippVázquez-Cano, EstebanKao, ChihwaVázquez-Castillo, JavierKao, Chun-ChiehVázquez-Cobela, RocíoKao, GraceVázquez-García, Rosa AngelesKao, Li-TingVecchia, Laura Adelaide DallaKao, Yu-HsiangVedal, SverreKapelko, MagdalenaVedøy, IngeborgKapischke, MatthiasVeedu, JaneeshKapitán, MartinVeenhoven, RuutKaplan, BekirVeerappan, ArulKaplan, BrentVeerkamp, JaapKaplan, SigalVega Gutierrez, SarathKaposztasova, DanielaVega, ClaudiaKapravelou, GaryfalliaVega-Zamora, ManuelaKapri, KulVegni, NicolettaKapritsou, MariaVeiga, Feliciano H.Kapsogeorgou, Efstathia K.Veiga, GlauciaKar, AnirbanVeiga, PaulaKar, ShantimoyVeiga-Seijo, RaquelKarabagias, Ioannis K.Veintimilla, Salvador García-AyllónKarabasevic, DarjanVel, Bharath KumarKarabetsos, EfthymiosVelander, FredrikKarabiyik, TugbaVelarde-Garcia, JuanKaraduta, OlegVelasco Garrido, MarcialKaragulian, FedericoVelasco, InésKarahan, H. EnisVelasco-Hernandez, Jorge X.Karakocak, Bedia BegumVelayudhan, LathaKarakuła-Juchnowicz, HannaVelázquez Blázquez, José SebastiánKaralis, Vangelis D.Velázquez, JavierKaralitsios, SpyrosVelázquez, RamiroKaramagioli, EvikaVelázquez-Cruz, RafaelKarambelas, AlexandraVeldman, Sanne L. C.Karanikas, NektariosVeledo, María Belén San PedroKarapinar, ErdalVelez, Anne-Lise K.Karasali, HelenVelez, MarcelaKaratela, ShamshadVelez, Maria JoãoKaravoltsos, SotiriosVelickovic, DusanKaraye, Ibraheem M.Velicogna, MarcoKarayiannakis, AnastasiosVeloso, Bruno MiguelKarbowski, AdamVeludo, FilipaKärenlampi, Petri P.Venäläinen, SallaKarim, Helmet T.Vencloviene, JoneKarim, M.Venco, Elisabetta MariaKarimi, BenyaminVencúrik, TomášKarimi, MaryamVenditti, FrancescoKarimi, MohammadVenegas, PabloKaripidis, KenVeneri, Diana A.Karipidis, PhilipposVenkataraman, RajeshKarkhanis, AnushreeVenkataramanan, VidyaKarlik, John F.Venkatesan, ThiagarajanKarliński, LeszekVennik, JaneKarlsson, ErikVentura Ribeiro, RosileneKarlsson, FredrikVentura, GabrielaKarlsson, LenaVentura-Marco, ManuelKarlsson, PetraVenuleo, ClaudiaKarmakar, ParthaVeny, Miguel BennasarKarnati, SreenivasVera Reyes, IleanaKärner, TobiasVera, TeresaKarniej, PiotrVeraldi, StefanoKarolinczak, BeataVerani, MarcoKarolkiewicz, JoannaVerbeek, HildeKaroń, GrzegorzVerbeek, ThomasKaronova, TatianaVerboven, KennethKarozis, SteliosVerdinelli, IsabellaKarp, Jonathan D.Verdu, ElenaKarpavičiūtė, SimonaVergalli, SergioKarpowicz, JolantaVergara, Lizandra Garcia LupiKarppinen, Jari E.Vergara-Moragues, EsperanzaKartsonaki, ChristianaVergidis, PaschalisKarunanayake, Chandima P.Vergnano, AndreaKarwowska, MałgorzataVergnes, Jean-NoelKarwowski, WaldemarVergos, KonstantinosKasai, FumioVerhaeghe, JohanKasai, NaoyaVerhagen, HansKasaraneni, Varun K.Verheul, JasperKascakova, NataliaVerhoeven, AdrieKaserzon, SaritVerkruijsse, WimKashima, SaoriVerma, Dinesh KumarKashou, AnthonyVerma, Shiv PrakashKaskaoutis, DimitrisVermeersch, PatKaskel, Frederick J.Vermeesch, AmberKasmaee, SaraVermeir, GerritKasparova, EvaVernez, DavidKasper-Jędrzejewska, MartynaVersaci, MarioKaspi, RoyVerso, Maria GabriellaKasuga, KensakuVerticelli, AliceKatada, KazuhiroVervaeke, William C.Kataev, EvgenyVeselka, BarbaraKatamoto, ShizuoVeskoukis, Aristidis S.Kataoka, HitomiVesselinov, ElenaKataoka, TomoyaVest, Katherine E.Katayama, TakayukiVeszteg, Róbert F.Katikou, PanagiotaVetri, LuigiKatona, JozsefVetter, SvenKatrakazas, ChristosVetter, SylviaKatsaliaki, KorinaVeyhe, Anna SofíaKatsanos, Aristeidis H.Veysey, MartinKatsantonis, Ioannis G.Via, Michael A.Katsavelis, DimitriosViana, AlonçoKatsavochristou, AnastasiaViana, David LeiteKatsinelos, TaxiarchisViana, PaulaKatsoyiannis, IoannisVianello, Francesca AliceKatsui, HisayoViazis, NikosKattoor, Ajoe JohnVicario-Merino, ÁngelKatuchova, JanaViccaro, MauroKatwal, DikshaViceconte, GiulioKatz, Ben Z.Vicent Juan, MariaKatz, Ian M.Vicente João, PauloKatz, IlanVicente Ruiz, María AsunciónKatz, JosephVicente, Cristina P.Katz, PattiVicente, EvaKatz, Sidney A.Vicente, LauraKatz, Yaacov J.Vicente, TaniaKaufhold, KellyVichi, FrancescaKaufman, PamelaVici, GiorgiaKaufmann, ChristopherVickers, BradKaufmann, Walter E.Victor, Megan RhodesKaul, SubuhiVidafar, ParisaKaur, BerinderjeetVidal, DiogoKaur, HarleenVidal, JosepKaur, MandeepVidal, LucianoKaurani, LalitVidal-Infer, AntonioKaushal, VinayakVidal-Raméntol, SalvadorKaushik, MaheshVidoni, EricKavaliauskas, MykolasVidu, AnaKavaliauskas, PovilasViegas, João CarlosKavalidou, KaterinaVieira, AlexandreKavanaugh, MelindaVieira, Edgar RamosKavanaugh-Lynch, MarionVieira, JoséKavecan, IvanaVieira, MargaridaKaviani, MojtabaVieira, SelmaKavroudakis, DimitrisVieira, Taian M.Kavvadia, KaterinaViennet, ElvinaKavvadias, VictorViennot, StéphaneKawa, ArkadiuszVierboom, Yana CatherineKawabata, HideakiVieru, MarkkuKawabe, KentaroVieyra, Jorge ParedesKawaguchi, Shin-ichiVigentini, LorenzoKawai, ToshioVigliaturo, RuggeroKawakami, RyokoVignais, NicolasKawakami, TsuyoshiVignoli, MichelaKawamura, HiroakiVijayakumar, SarathKawano, KouichiroVik, TennleyKawasaki, TsubasaViktorova, LucieKawata, KeisukeVila-Candel, RafaelKawata, YukichikaVila-Chã, CarolinaKawauchi, HideyukiVilardi, GiorgioKay, SimonVila-Suárez, Maria ElenaKay, ValerieVila-Vázquez, GuadalupeKaya, Gulsum KubraVilcassim, M. J. RuzmynKayali, GhaziVílchez, PilarKayano, RyomaVilda, DovileKayano, TaishiVilela, SofiaKaye, Linda K.Vilhelmsson, AndreasKayser, BengtVilla-Bellosta, RicardoKayser, KlausVillafaina, SantosKazak, JanVillani, Maria GabriellaKazemi, MaryamVillani, RosannaKC, RajanVillanueva, VicentKe, TaoVillarejo-Carballido, BeatrizKe, YangchuanVillares, RubénKearney, JohnVillari, PaoloKeating, DennisVillarini, AnnaKebbe, MaryamVillarreal-Gómez, Luis JesúsKeboa, Mark TambeVillaseñor, AlmaKedrowicz, April A.Villaume, KarinKędzierska, EwaVillaverde, Juan JoséKeegan, ThomasVillavicencio, Vanessa PinoKeeler, JasonVillena Carpio, OswaldoKeeley, AlexanderVilleneuve, MichelleKeen, Justin MVillen-Guzman, MariaKeenan, Jeffrey A.Villotti, PatriziaKeenan, StaceyVimercati, LuigiKeeney, AnnieVimieiro, Claysson BrunoKefallonitis, StathisVincensi, BarbaraKefeni, KebedeVincent, Amy E.Kehler, Dustin ScottVincent, GraceKeida, ElizabethVincent, Jean-louisKeidar, OsnatVincent, John BertramKeler, AndreasVincenten, JoanneKelesi, MarthaVincenti, FlaminiaKellar-Guenther, YvonneVinciguerra, ManlioKeller, Amy CelesteVine, Michelle M.Keller, FriederViner, BrianKeller, HeatherVining, Kyle HolmbergKeller, JoshuaVink, MarkKeller, SarahVin-Raviv, NeomiKelly, DanielVinturache, AngelaKelly, DervlaViola, SerenaKelly, Kevin M.Violant-Holz, VerónicaKelly, KimberlyViolanti, John M.Kelly, Louise M.Vioque, IgnacioKelly, MatthewVirtanen, HeliKelly, MichaelVirtanen, JormaKemper, HanVisconti, FedericaKemper, JoyaVisentin, MicheleKemperman, AstridVisioli, GiovannaKennair, Leif Edward OttesenViskup, RichardKennedy, AaronVismara, LauraKennedy, AlisonVišnjić, AleksandarKennedy, GerardVisser, AnitaKennel, JulieVisser, BartKenry, K.Visser-Nieraeth, AnjaKent, JenniferVissink, ArjanKent, KatherineVita, RobertoKeogh-Brown, MarcusVitacca, MicheleKepalaite, AlbinaVitale, AgataKephart, Josiah L.Vitale, Jacopo A.Kephart, WesleyVitale, MariaKeptner, Karen MarieVitale, MarilenaKeramatikerman, MahdiVitale, Salvatore GiovanniKerhervé, Hugo A.Vitali, MatteoKerkhof, PeterVitetta, LuisKerman, KaganVitiello, Virginia E.Kerr, GaigeVitish-Sharma, ParveenKerry, Matthew J.Vitoratou, SiliaKersten, Jan FelixViuda-Martos, ManuelKersten, SanderVivaldi, RominaKeshet, YaelVivat, BellaKeskinen, KirsiViviani, SimonettaKesseler, MatthewVizcaya-Moreno, M. FloresKesselring, JuergVizzari, MarcoKetelhut, Sascha R.Vlachou, EugeniaKethireddy, SwatantraVlachou, StylianiKeul, AlexanderVlakveld, WillemKevany, KathleenVlasov, DmitryKevorkian, MelineVlassopoulos, AntonisKewley, ChrisVlčková, MiroslavaKeynan, IritVnouckova, LucieKhacharem, AïmenVoaklander, Donald C.Khaddaj Mallat, RayanVoellger, BenjaminKhade, Natasha B.Vogel, MatthiasKhademi-Vidra, AnikoVogelaar, BartKhader, AbdelhaleemVogl, ClausKhakimova, AidaVoglino, GianlucaKhalaj, OmidVogt, KristiinaKhaled, AhmedVoix, JérémieKhaled, YasserVolaklis, KonstantinosKhalid, HattafVolenzo, Tom ElijahKhalili, SiavashVoliani, ValerioKhamkure, SasirotVolicer, LadislavKhan, AbdulVolken, ThomasKhan, AdnanVolkov, ArtiomKhan, AsaduzzamanVolland, GerhardKhan, DibaVollmer, BrigitteKhan, HafizVollmer, MarcusKhan, JibranVollmer, RachelKhan, Khurram J.Von Der Thüsen, JanKhan, MatlubaVon Essen, ElisabethKhan, Md. ShakhaoathVon Kutzleben, MilenaKhan, Mohd ParvezVon Philipsborn, PeterKhan, Naseer AbbasVonWald, IanKhan, ShahanshahVoothaluru, RohitKhan, ShahrukhVormann, JürgenKhan, Shazada Muhammad UmairVoroney, PaulKhan, TanvirVoroshilova, AnzhelikaKhan, TehminaVos, MargreetKhan, Zeeshan AhmadVos, StevenKhanal, RajendraVoss, JoachimKhanday, Mudasir A.Vossos, HeleneKhandelwal, SoniVousoura, EleniKhanna, Aniruddh JagdishVozinaki, Anthi-EiriniKhanra, NandishVozza, IoleKharat, SuhasVrachatis, Dimitrios A.Kharin, YuriyVrana, ScottKharroubi, SamerVriesekoop, FrankKhaustov, Aleksandr P.Vu-Eickmann, PatriciaKhezr, SeyednimaVuillaume, Jean-FrancoisKhezri, AbdolrahmanVuillermoz, CecileKhlat, MyriamVuletić, GorkaKhoeurn, KimleangVuong, Quan-HoangKhokhar, BilalVuorio, AlpoKholmukhamedov, AndalebVyas, PriyankaKholod, NazarWaak, MichaelKholodov, VladimirWachter, Jan K.Kholová, IvanaWachtler, CarolineKhor, Yet HongWacławek, StanisławKhorasani, Elham SahebkarWada, ChikamuneKhorram-Manesh, AmirWada, JunKhorsandi, FarzanehWadhi, TanujKhoury, DaliaWadley, Susan SnowKhubchandani, JagdishWadowski, Patricia P.Khuder, Sadik A.Wadzinski, ThomasKhujanazarov, TemurWagenfeld, Amy E.Khullar, SachinWagenmakers, AntonKhurshid, ZohaibWagganer, Jason DanielKhushi, MatloobWagh, PriyeshKiatkawsin, KiattipoomWagley, SariqaKiausiene, IlonaWagner, AbramKicińska, AlicjaWagner, CoraKico, IrisWagner, NataliaKida, MałgorzataWagner, SabinaKidando, EmmanuelWagner, TeresaKidd, Lisa A.Wagnild, JanelleKiec, MariuszWagnsson, StefanKiel, EllenWagoner, Rietta S.Kieson, EmilyWähälä, KristiinaKievit, RogierWahbeh, AbdullahKihm, HollyWahlström, ViktoriaKijo-Kleczkowska, AgnieszkaWainwright, ElaineKikusui, TakefumiWainwright, JohnKikuyama, MasatakaWakatsuki, MasaruKilian, CarolinWakeel, AbdulKilian, KrzysztofWakefield, RonKillian, Manuela SonjaWakeman, ShawneeKillikelly, ClareWakholi, PeterKilner, TimWakjera, Eshetu JankaKim, Amy Chan HyungWalabyeki, JulieKim, BongjuWalcek, ChrisKim, BongleeWalczak, MaciejKim, ByoungsooWalczak, Radoslaw B.Kim, Byung-JikWalczak, RenataKim, Chun-BaeWalczyk, AgnieszkaKim, Dae-KwangWaldherr, KarinKim, Dal HyungWaldhoer, ThomasKim, DayoonWaldhör, ThomasKim, Do-HounWałdykowski, PiotrKim, EungdoWalecki, PiotrKim, Eun-JungWales, LornaKim, GeonwooWali, JibranKim, George R.Walia, BhavitaKim, Gi BeomWaligóra, MarcinKim, Hae WonWalker, ArleneKim, Hae-YoungWalker, Blake ByronKim, Han-NaWalker, HeatherKim, HeejungWalker, Iain (Australia)Kim, HoWalker, Iain (USA)Kim, Ho GulWalker, MarvinKim, HonghyokWalker, Meegan A.Kim, Hyeon-CheolWalker, ShawnKim, HyeongsuWalker-Gleaves, CarolineKim, Hyoun (Andrew)Walkowiak, DariuszKim, Hyoung-RyoulWall Medrano, AbrahamKim, Hyun-DuckWall, Andrew F.Kim, HyungjooWall, TonyKim, HyungKyooWallace, EmmaKim, Jae-HongWallace, JulianeKim, JarimWaller, MichaelKim, Jee HwanWallner, StefanKim, Jee HyunWall-Reinius, SandraKim, JeonghyunWalls, KelvinKim, Jeong-YeonWalls, RichardKim, Jin-BomWalsh, CorinnaKim, JinhoWalshe, CatherineKim, Jinkyung JennyWalshe, Elizabeth A.Kim, JisuWalter, Angela WangariKim, Ji-SuWalter, Lauren A.Kim, JiyunWalter, Suzy MascaroKim, Jong InWalters, DianneKim, Jong-EunWalters, MarkKim, Jong-MinWalton, AnnMarie L.Kim, Joo YoungWalton, WilliamKim, JoosupWalton, William E.Kim, Ju HeeWalton-Moss, Benita JeanneKim, Jung-HaWalubita, LubindaKim, JunghoonWalwyn, Wendy M.Kim, JungyoonWalyaro, DickKim, KijinWamai, RichardKim, Kil-SooWan, LeiKim, KisookWan, ShibiaoKim, Kyoung MinWan, ThomasKim, KyoungjaWan, Thomas T. H.Kim, MinseoWanchai, AusaneeKim, Min-seongWanda, Wilczyńska-MichalikKim, Min-SukWang, ​ZhenpingKim, Min-YoungWang, BaoKim, NamkwenWang, BingshunKim, OksooWang, CaoKim, PearlWang, ChanghongKim, Rae YuleWang, Chao (Louisiana State University)Kim, Ryong NamWang, Chao (Research associate of Salk Institute) Kim, Sang SukWang, ChaoChenKim, Sang UgWang, Cheng (China)Kim, Sang-DolWang, Cheng (USA)Kim, Sang-JoonWang, ChenghaoKim, Seong-GonWang, Chih-HaoKim, Seong-ilWang, ChuanyiKim, Seong-TaeWang, Chung-JenKim, Seung-NamWang, CuicuiKim, SiinWang, DanlingKim, SojungWang, David Q.Kim, SokWang, David Q.-H.Kim, SujinWang, Di (Germany)Kim, Sun-AeWang, Di (Macao)Kim, SunghanWang, DieKim, Sung-HeeWang, DingKim, SungroulWang, DongqingKim, SunheeWang, Fan (China)Kim, Sun-YoungWang, Fan (UK)Kim, Tae ImWang, Fu LeeKim, Tae KonWang, Fu-jenKim, Tong-SooWang, Gai-GeKim, Woo-JinWang, GangKim, Yeo HyungWang, GuanKim, Yeong JaeWang, GuoxiangKim, YeonwooWang, GuozhengKim, YeowonWang, HaiKim, Young RanWang, HaijunKim, YoungBinWang, HaikouKim, Young-JaeWang, HaiyanKim, Young-TaekWang, Han-HsiangKim, Young-zoonWang, HaoKimball, Bruce A.Wang, HaoxiangKimball, MindyWang, HaoyuKimber-Trojnar, ŻanetaWang, Harry H. X.Kimbrough, David EugeneWang, HohuiKimmerle, JoachimWang, HongKimoto, MitsuhikoWang, HongruiKinahan, AoifeWang, HongwuKinar, Nicholas J.Wang, HuiKinarivala, NiharWang, Jason J.Kind, NinaWang, Jeff C. W.Kindermann, HaraldWang, JianmingKindu, MengistieWang, Jian-WenKindy, MarkWang, JianxiongKing, ChristianWang, JianxuKing, Elizabeth J.Wang, Jie (Jane)King, Jessica L.Wang, JindongKing, Juliet L.Wang, JingKing, KylieWang, Jin-JinKingshott, BrianWang, JoannaKingsley, J. DerekWang, JunKingston, HudsonWang, Kai (China)Kinney, Gregory LoydWang, Kai (USA)Kinnunen, Ulla-MariWang, KyleKinoshita, RyoWang, LanKinsky, Suzanne M.Wang, LeiKious, BrentWang, LeyiKiper, PawelWang, Liang-JenKirby, GilesWang, LidongKirby, Russel S.Wang, LijunKirchen, TwyllaWang, LiliKirchengast, SylviaWang, LimingKirchner, StefanWang, Lin (China)Kirenga, Bruce J.Wang, Lin (France)Kirkbride, James B.Wang, Ling (China)Kirnan, JeanWang, Ling (USA)Kirpich, AlexanderWang, LizhenKirsch, ThereseWang, LuqiKirshbaum, Marilynne N.Wang, MansiKirton, Stewart B.Wang, NianxinKirves, KaisaWang, NingKiselev, JörnWang, OuKiseleva, Irina V.Wang, Pa-ChunKishcha, PavelWang, PeiKisko, TheresaWang, Peng-HuiKiss, MariettaWang, Peng-WeiKissimova-Skarbek, KatarzynaWang, PingKistler, KurtWang, PingyuKiszewski, Anthony E.Wang, PingyuanKita, DaichiWang, Po-ChingKitagawa, KaoriWang, QiKitsios, FotisWang, QiangKittel-Schneider, SarahWang, QiaoyingKitzmüller, ClaudiaWang, QinyiKizilhan, Jan IlhanWang, QunKiziltoprak, SefikaWang, RuoxiKlapdor, RüdigerWang, San-YuanKlassen, ViktorWang, ShanKlaumann, FranciniWang, ShanyongKlaus, SusanneWang, ShaobinKleczkowska, PatrycjaWang, Shao-ChengKleib, ManalWang, ShaohuaKlein, JennieWang, Shau-ChunKlein, JensWang, Sheery YunKleinsorge, ThomasWang, Sheng-FanKleka, PawełWang, ShigongKlekiel, TomaszWang, ShushengKlemeš, Jiří JaromírWang, ShyiwuKlentrou, PanagiotaWang, SongboKlibi, WalidWang, Tao (Capital Normal University)Klich, SebastianWang, Tao (Central University of Finance and Economics) Klimašauskas, AndriusWang, Teresa W.Klimczuk, AndrzejWang, TongKlimek, AndrzejWang, Tsung-JenKlimek, DariuszWang, Tzu-ChuehKlimek, MarkusWang, WeijieKlimko, RomanWang, WeiweiKlimontov, VadimWang, William Yu ChungKlimova, AlexandraWang, XanderKlímová, BlankaWang, XiKlingensmith, J. ZacharyWang, XianyuKlinger, Francesca　Wang, XiaohuaKlinger, MarianWang, Xiao-HuaKlinich, Kathleen DeSantisWang, Xiao-LiKlizienė, IrinaWang, XiaoqinKlobučar, HrvojeWang, XinKlobucar, Philip AndrewWang, XinxinKłos, MarcinWang, XishaKlosok-Bazan, IwonaWang, XuKlug, DennisWang, XuantongKlug, KatharinaWang, XueweiKluger, NicolasWang, XushengKlukowska-Rötzler, JolantaWang, Ya-hueiKlusmann, VerenaWang, Yang (Missouri University of Science and Technology) Kmush, BrittanyWang, Yang (University of Wisconsin-Mil-waukee) Knai, CecileWang, YanleiKnapp, Kenneth A.Wang, YiKnechtle, BeatWang, YiboKneen, Melanie AnneWang, YichenKnight, Erik L.Wang, Yi-HsienKnighton, ShaninaWang, YixiangKnöchelmann, AnjaWang, Yi-zhiKnoefel, FrankWang, YoujinKnoll, Eva-MariaWang, YouxinKnoop, KathrynWang, YuhongKnorr, HelenaWang, YulingKnowlton, Barbara J.Wang, YunmingKnuckles, TravisWang, ZenengKnudsen, Elise ArsenaultWang, ZhanyongKnudsen, Jakob BrandtbergWang, ZhaoningKo, Chih-HungWang, ZhaoxinKo, DamiWang, ZheKo, HansooWang, ZhenKo, Hsin-ChiWang, ZhenboKo, JesukWang, ZhenhongKo, KisungWang, ZhenjieKo, Young DaeWang, ZhiruKob, RobertWang, ZhiweiKobayashi, HiroakiWang, ZhuoyingKobayashi, HiromitsuWang, ZidongKobayashi, NobumichiWang, ZixinKobayashi, TetsuroWang, ZunyaoKobayashi, YayoiWangdi, KinleyKobbe, RobinWańkowicz, PawełKobeissy, FirasWanschel, Amarylis Claudine Bonito A.Koberová Ivančaková, RomanaWarburton, WayneKobierzycki, ChristopherWard, AmyKobler, James BradleyWard, LeighKobus, PawełWard, MarkKobus-Cisowska, JoannaWarkentin, SarahKobyłecki, RafałWarmelink, LaraKobza, JoannaWarms, Richard L.Kobza, JozefWarnat, StephanKoch, KatherineWarner, Mildred E.Koch, RolandWarnick, Jennifer L.Koch, WojciechWarren, GrahamKochetova, Tatyana ViktorovnaWarren, JimKocian, AlexanderWarren, PaigeKociu, ArbenWarrington, ClaireKock, LorenWarshawsky, Daniel N.Kocot, EwaWartalska, KatarzynaKocur-Bera, KatarzynaWarusavitarne, JanindraKoczanowicz, LeszekWąs, AdamKoczkodaj, PawełWas, Christopher A.Koda, EugeniuszWashbourne, CarlaKodali, MaheedharWashburn, David J.Kodama, HideyaWashio, YukikoKodama, KotaWąsik, JacekKodziszewska, KatarzynaWasileski, GabrielaKoebele, ElizabethWasowicz, MarcinKoechlin, HelenWąsowski, JacekKoehler, MatthewWatanabe, EiichiKoelmans, BartWatanabe, FernandaKoelmel, BernhardWatanabe, MikikoKoelmel, JeremyWatanabe, YuichiKoerber, AmyWatanabe, YuichiroKoerner, StephanieWatanabe, YutakaKofler, DorisWatari, TakahiroKoga, Patrick MariusWaterlot, ChristopheKoh, Jae ChulWaters, KristinKohlenberger, JudithWathington, DeannaKohlitz, Jeremy P.Watkins, JamesKohls, NikoWatkins, Kenton BradleyKohyama, JunWątor, KatarzynaKoike, KenzoWatrobski, JaroslawKoike, TakayukiWatso, Joseph C.Koirala, RanjanWatson, PoppyKojima, HajimeWatson, RogerKojima, ReijiWatson, Silvana Maria RussoKojima, TaroWatt, RichardKok, WouterWatt, SimonKökény, MihályWattage, PremachandraKokkalera, Stuti S.Watts, PaulKokkinakis, EmmanouilWauters, LucasKokkinos, EvgeniosWautier, Jean-LucKokkonen, TommiWawrzyniak, AgataKokot, JohannaWaxman, EleanorKokubun, KeisukeWay, KirstenKokubun, TakanoriWaye, Mary M. Y.Kolanowski, WojciechWayland, SarahKolarczyk, EwelinaWdowiak, KamilKolarik, Andrew J.Weakliem, DavidKolawole, AbimbolaWeaver, Connie M.Kolbenschlag, JonasWeaver, Frances M.Kołcz, AnnaWeaver, ScottKolena, BranislavWebb, AnnKoleňák, JiříWebb, BenjaminKolendowicz, LeszekWebb, Bryn D.Koletsi, DespinaWebb, Cameron E.Kolev, MikhailWebb, GinnyKolishetti, NageshWebb, JohnKolivakis, TheodoreWebb, KarenKollath-Cattano, ChristyWeber, EllerieKollipara, AvinashWeber, EricaKollmuss, MaximilianWeber, Griffin M.Kolo, JerryWeber, HeinrichKołodziejczak, MałgorzataWeber, JanetKołodziejczak, WłodzimierzWeber, KonradinKolota, AleksandraWeber, Silke Anna TheresaKoltermann, JanWee, HweeKoltermann, Jan JensWee, JosephineKolvereid, LarsWeeks, FaithKolwicz Jr., Stephen C.Weghorn, HansKolya, HaradhanWegman, FredKomar, NicholasWehner, CarolineKomaris, Dimitrios SokratisWei, BeiganKomatsu, DavidWei, DongKomatsu, KazuhiroWei, GangKomeili, AminWei, Hsi-HsienKomerath, Narayanan M.Wei, JingKaiKomesli, MuratWei, QingyangKomoike, YutaWei, Wei (Nanjing Normal University)Komorowski, MatthieuWei, Wei (Xi’an University of Technology)Komosinska-Vassev, KatarzynaWei, XinyangKompala, JanuszWei, XinyuanKompaniyets, LyudmylaWei, XuebinKompotis, KonstantinosWei, YangKon, MichihiroWei, YanqiaoKonarski, JanWei, YeKondo, HitoshiWei, YigangKondo, MichelleWeidhaas, JenniferKondo, YutakaWeierbach, Florence M.Kondracki, AnthonyWeigelt, OliverKonefał, MarekWeijs - Perrée, MinouKones, RichardWeilenmann, Anne-KatharinaKon-Fook Chong, AlbertWeiner, AdeleKong, De-MingWeinhaeusel, AndreasKong, JianWeinstein, JohnKong, WeiliWeisfeld, Glenn E.Kong, XiangruiWeiss, Carol D.Kong, XiangxiongWeiss, MargaretKong, ZhaoweiWeiss, PeterKönig, BrigitteWeißenfels, AnjaKonijnenberg, CarolienWeissmannová, Helena DoležalováKoninckx, Philippe R.Weitz, KeithKonishi, ShokoWeixin, ZhaiKonjufca, VjollcaWeizman, YehudaKonkle, AnneWelch, DavidKono, YasuyukiWelch, Stephen R.Konovalov, ElenaWeller, Jason L.Konradsen, HanneWeller, Penelope JuneKonstanciak, AnnaWellmer, Friedrich-W.Konstandinidou, MyrtoWells, EllenKonstantakopoulou, FotiniWells, YvonneKonstantin, MiltoWelton-Mitchell, CourtneyKonstantinides, DimitriosWen, ConghuaKonstantinidi, AikateriniWen, FengKonstantinova, MarinaWen, Huei-JhenKontogiorgis, ChristosWen, Kuo-HsunKontou, EleftheriaWen, SijinKontsevaya, IrinaWen, ZhiqiangKónya, JózsefWen, ZhiweiKoo, MalcolmWendelboe-Nelson, CharlotteKoo, YoonmoWenderfer, Scott EdwardKoorey, GlenWender-Ozegowska, EwaKoot, Jaap A. R.Wendy, WismerKoozehchian, MajidWenfeng, ZhengKopaczka, MarcinWeng, XueKopcakova, JaroslavaWeni, Zeng Y.Koposko, JanetWenos, JeanneKopp, StevenWentz, Laurel M.Koppe, Janna G.Wenzel, UweKoppelaar, HenkWerbińska-Wojciechowska, SylwiaKoppisch, DorotheaWerenowska, AgnieszkaKopustinskiene, Dalia M.Weres, AnetaKopylov, YuriWerland, StefanKorcz, AgataWermuth, Maria-CorneliaKorf, NathalieWerner, IngeKorinthenberg, RudolfWerner, MałgorzataKorivi, MallikharjunWernio, EdytaKorkontzelos, YannisWernly, BernhardKormuth, KarenWerron, TobiasKorneev, DenisWesseler, Justus H. H.Korobeynikov, GeorgiyWesseling, Jan G.Korponai, JanosWest, JanneKortenkamp, KatherineWest, JeffreyKortüm, K. MartinWest, JuliaKoryak, Yuri A.West, MichaelKorzeniewska, EwaWesterdahl, DaneKos, BarbaraWesterholt, RenéKosa, KarolinaWestermair, Anna L.Koseoglu, NazliWestermann, ClaudiaKoshiyama, MasafumiWestgård, TheresaKosinski, ErykWestmark, Cara J.Kosinski, PrzemyslawWhanhee, LeeKosovac, AnnaWheeler, Darrell P.Kostakis, IoannisWhiley, HarrietKostalova, JanaWhillier, StephneyKostaras, PanagiotisWhitaker, Mark D.Kostareva, AnnaWhitburn, LauraKostas, Stamoulis C.White, AndrewKostas-Polston, ElizabethWhite, BarbaraKostecka, MalgorzataWhite, HankKostenidou, EvangeliaWhite, JacquieKoster, BrianWhite, JanineKostrakiewicz-Gierałt, KingaWhite, KateKostrzewa, MaciejWhite, NicoleKostrzewa-Nowak, DorotaWhite, StephenKostrzewski, MariuszWhite, Sydney D.Kot, SebastianWhite, WendyKotfis, KatarzynaWhite, ZeldaKotiya, DeepakWhitehead, Amy E.Kotlowski, RomanWhitehead, DeanKotnala, SudhirWhitehead, Margaret E.Kotova, AnnaWhitelaw, GailKotowski, TomaszWhiteman, AriKotrschal, KurtWhiteside-Mansell, LeanneKotsis, KonstantinosWhite-Williams, Cynthia LKottakota, ChandrasekharWhitfield, Geoffrey P.Kotula-Balak, MałgorzataWhitley, Deborah M.Kotval-Karamchandani, ZeenatWhitmore, Kim E.Kotzalidis, Giorgio D.Whittaker, PeterKou, GangWhitting, JohnKou, HuaiyunWhooten, RachelKouchi, MakikoWiatrak, BenitaKouidrat, YoussefWichrowski, MattewKouji, HaradaWichrowski, MatthewKoukouli, SofiaWickliffe, JeffreyKoul, SahilWickramaratne, PriyaKoulinas, Georgios K.Widemann, EmilieKouokam, Joseph CalvinWidziewicz-Rzońca, KamilaKoura, FatimaWieber, FrankKourmatzis, AgisilaosWięch, PawełKourmentza, ConstantinaWięckowska, BarbaraKoustas, EvangelosWięckowski, MarekKoutelidakis, Antonios E.Wieczorek, AnetaKoutelidakis, IoannisWieczorek-Kosmala, MonikaKoutná, VeronikaWieczorek-Szymańska, AnnaKoutra, SesilWielgomas, BartoszKovacic, Melinda ButschWieliczko, BarbaraKovács, LeventeWielki, JanuszKovacs, RoxanneWiener, R. ConstanceKovács, TiborWiens, KirstenKovaleva, MariaWierzejska, ReginaKoven, Steven G.Wieser, HeikeKowal, KrzysztofWiggill, MarleneKowal, MałgorzataWiggs, MichaelKowal, PrzemysławWiist, William H.Kowalczuk, IwonaWijayatunga, NadeejaKowalczuk, KrystynaWijnveld, MichielKowald, MatthiasWilczyński, JacekKowalewska, EwelinaWildcat, MatthewKowalewski, MaciejWilding, SarahKowalik, JaroslawWildman, JosephineKowalska, Anna SylwiaWilk, AleksandraKowalska, GrażynaWilke, AndreKowalska, Jolanta E.Wilkie, CraigKowalska, MalgorzataWilkinson, AnneKowalska-Góralska, MonikaWilkinson, ChristopherKowalski, Arkadiusz M.Wilkinson, Larrell L.Kowalski, CezaryWilkinson, ShelleyKowalski, DariuszWillaing, IngridKoyack, MarcWilland, NicolaKoyama, MitsuhikoWillems, Mark Elisabeth TheodorusKoyama, YuWillers, ClarissaKozak, AgnessaWilliam, LauraKozak, AnnaWilliams, AlfredKozdru, WojciechWilliams, AlisonKozicki, MateuszWilliams, E. MarkKoziel, JacekWilliams, FaustineKozielska, BarbaraWilliams, Gustavious PaulKozlakidis, ZisisWilliams, HelénKozłowski, ArkadiuszWilliams, JaneKozlowski, KarlWilliams, LeahKozłowski, RemigiuszWilliams, MeganKozuba, JaroslawWilliams, MyiaKozyra, CyprianWilliams, PeterKraemerc, SusanneWilliams, RoderickKrafsur, GretaWilliams, SianKrägeloh, ChristianWilliams, SusanKragness, HaleyWilliams, Susan G.Kragović, MilanWilliams, VictoriaKrähenbühl, StephanWilliamson Smith, RachelKrajcsák, ZoltánWilliamson, Heather J.Krajewska-Włodarczyk, MagdalenaWilliamson, HowardKrajewski, AdamWilliford, HenryKrajewski, PiotrWilliman, JonathanKrajewski, WojciechWilling, EstherKrajnak, Kristine M.Willis, ErinKrajnović, DušankaWillis, SarahKrakowski, PrzemysławWilson, Alphus D.Kramar, UrošWilson, AnnabelleKramer, TobiasWilson, BarbaraKraska, ThorstenWilson, Christopher G.Krasuski, AdamWilson, DonnaKrata, Agnieszka AnnaWilson, JacobKratzke, CynthiaWilson, JamesKrauklis, Andrey E.Wilson, JasonKraus, PaulWilson, Leslie S.Krausfeldt, LaurenWilson, Machelle D.Kraut, AllenWilson, Mark J.Kravats, AndreaWilson, PeterKregiel, Dorota MariaWilson, Philippe B.Kreider, Consuelo M.Wilson, SamuelKreider, Richard B.Wilson, SueKreidl, PeterWilson, TomKreiß, CorneliaWilson-Evered, ElisabethKrell, Rayda K.Wilt, JoshuaKrengel, Maxine H.Wimmer, LenaKretzschmar, AndréWindschitl, PaulKrewani, AngelaWinger, QuintonKrieg, SusanWiniarczyk-Raźniak, AnnaKrishna, GokulWiniarska-Mieczan, AnnaKrishnamurthi, NarayananWinings, KathyKrishnan, SowmyaWinkler, BastianKristjansson, AlfgeirWinkler, David A.Kristjansson, ElizabethWinn, BryanKristjánsson, KristjánWinneke, GerhardKristo, AleksandraWinner, HannesKristo, IvanWinnington, RhonaKritee, KriteeWinocur, EphraimKrockow, EvaWinston, BruceKroeker, AndreaWinston, Bruce E.Krog, Norun HjertagerWinter, ChristianKröger, EdeltrautWinter, LaraineKrois, JoachimWinter, Virginia RamseyerKról, AleksanderWinwood, PeterKról, DanutaWipfli, BradKról, KarolWirth, MichaelKról, NinaWiśniewska, DominikaKróliczewska, BożenaWiśniewski, AdamKrólikowska, AleksandraWiśniewski, PawełKroll, ThiloWiśniewski, PiotrKról-Zielińska, MagdalenaWiśniowska, EwaKromydas, TheocharisWiśniowska-Szurlej, AgnieszkaKronborg Bak, CarstenWiszumirska, KarolinaKron-Rodrigues, Meline RossettoWitchel, Harry J.Krówczyńska, MałgorzataWitowski, JanKrstacic, GoranWitruk, EvelinKruchinina, Natalia EvgenievnaWittlich, MarcKrueger, AndreasWittmann, MarcKrueger, EddyWittmann, MariaKrueger, Richard B.Włodarczyk, AnetaKrug, IsabelWłodarczyk, MałgorzataKrug, RalfWłodarczyk, Paweł P.Krüger, BjörnWłodarek, DariuszKrüger, FredWöber, ChristianKruger, KarenWoche, Susanne K.Krüger, Renata L.Woessner, Mary N.Kruger, RuanWohlgemuth, NicholasKruger, Tillmann H. C.Wohlrabe, KlausKruopienė, JolitaWohner, BernhardKrupa-Kozak, UrszulaWojciechowska-Solis, JuliaKruszelnicka, OlgaWojciechowski, WallyKrysinska, KarolinaWojcik, CezaryKrysko, KristenWójcik-Gront, ElżbietaKrysta, KrzysztofWójcik-Leń, JustynaKrzych- Fałta, EdytaWojciuk, BartoszKrzych, LukaszWojewodzka-Wiewiorska, AgnieszkaKrzyściak, WirginiaWojkowska-Mach, JadwigaKrzystanek, MarekWojnarowska, MagdalenaKrzysztof, GorynskiWojnicz, WiktoriaKrzysztofik, MichałWojszel, Zyta BeataKrzywanski, JaroslawWojtal, Agata Z.Krzyżewski, RogerWojtczuk-Turek, AgnieszkaKshirsagar, Prakash G.Wojtyńska, AnnaKsieniewicz-Woźniak, EdytaWolanśki, WojciechKsinan, Albert J.Wolbring, GregorKuan, GarryWold, BenteKuan, Yu-HsiangWoldeamanuel, Yohannes W.Kubal-Czerwińska, MagdalenaWolfel, Richard L.Kubátová, JaroslavaWolff, ChristopherKubica, JadwigaWolfson, MarkKubica, PawełWolkoff, PederKubicek, ChristianWollscheid, JimKubicek-Sutherland, Jessica Z.Wolniak, RadosławKubicz, JustynaWolter, TilmanKubik-Komar, AgnieszkaWołyniec, WojciechKubilius, RaimondasWomack, CarolineKuboń, MaciejWomack, VeronicaKubota, KeiichiWon, Chang WonKubota, YasutakaWon, DoyeonKubrak, TinaWon, MinsuKuča, KamilWon, SeahwaKucharska, KarolinaWong, Arkers Kwan ChingKucharska, MałgorzataWong, Chi-kin KenchiKucharski, MariuszWong, Dennis C. C.Kuciene, RenataWong, Florence Mei FungKucukkal, TugbaWong, Ho TingKudesia, RaviWong, JuliaKudláček, MichalWong, Ka Ki DavidKudłak, BłażejWong, Ka-ChunKudlek, EdytaWong, KapoKudrna, PetrWong, Kee ChoonKudryashova, Tatiana V.Wong, Natalie W. M.Kudryavtsev, Denis S.Wong, Ngai Sze CandyKudur Jayaprakash, GururajWong, PaulinaKuehl, LinnWong, Peter D.Kuehl, SilkeWong, Te-ChihKühne, OlafWong, WaiKühnel, AnjaWonhyuk, ChoKuipers, Mirte A. G.Woo, AyoungKujawska, JustynaWoo, Dong KookKujawska, MałgorzataWoo, Hyung RokKukielka, ElizabethWoo, JeanKularatne, KanchanaWoo, MyungjeKulda, VlastimilWood, AlisonKulhánová, IvanaWood, AmandaKulicki, PiotrWood, ElizabethKuligiewicz, ArturWood, Jennifer L.Kulik, AgnieszkaWood, KevinKulik, Margarete C.Woodfield, LorayneKulikowski, KonradWoodhall-Melnik, JuliaKulkarni, AnandWoods, John A.Kulkarni, GajananWoods, SimonKulkarni, Madhavi ThombreWoodward, AniekKulkarni, RitwijWoodward, BillKulusjärvi, OutiWoodward, Owen M.Kumamaru, HirakuWoodward, RobertKumar, AdarshWoolcock, GeoffreyKumar, AdityaWoon, LukeKumar, AmitWoosnam, Kyle MauriceKumar, AnandWoroniecki, Robert P.Kumar, BinodWorsnop, Catherine Z.Kumar, GauravWoskie, SusanKumar, ManishWoźniak, LucynaKumar, NareshWoźniak, ŁukaszKumar, PankajWoznicka, MalgorzataKumar, PrakashWraith, DarrenKumar, PraveenWright, CaradeeKumar, RajWright, Caradee YaelKumar, RameshWright, ElinaKumar, RavindraWright, EricKumar, SandeepWright, HattieKumar, SanthoshWright, Jacob L.Kumar, SantoshWright, MichelleKumar, SatishWright-Berryman, JenniferKumar, ShivendraWroblewski, AngelaKumar, SujeetWrzesien, MajaKumar, V. KrishnaWrzesińska, MagdalenaKumar, VijayWu, Chang-YuKumar, VikasWu, ChaoKumara, Ajantha SisiraWu, Chieh-ChenKume, KensukeWu, Chih-DaKumhálová, JitkaWu, Chih-Da JohnKumi-Diaka, JamesWu, Chih-HangKummer, KaiWu, Chih-WenKumnig, MartinWu, Chueh-HungKump, DavidWu, Chyi-InKumrungsee, ThanutchapornWu, DienKun, ÁgotaWu, GuochunKun, BernadetteWu, I-ChinKundi, MichaelWu, Jack H. Z.Künemund, HaraldWu, Jian-HongKung, Chin-PingWu, JianqingKung, KarsonWu, JiansongKung, Woon-ManWu, JianzhangKunicki, MichałWu, Jia-RungKunkel, Suzanne R.Wu, JiaweiKunoh, TatsukiWu, JileiKunst, AntonWu, Jin-MingKunwar, BhagawatiWu, JoKuo, Hsien-WenWu, Jong-WuuKuo, Jun-YuanWu, JunKuo, Ko-LinWu, Kuen-YuhKuo, Ming-TseWu, KushengKuo, Shu-lungWu, LingtaoKuo, Ting-ChunWu, Manfred Man-FatKuo, Tsai ChiWu, MingjunKuo, YiliangWu, MoleiKupcewicz, EwaWu, NanKupsco, AllisonWu, Pei-IngKurath-Koller, StefanWu, QiaobingKurihara, ToshiyukiWu, ShengruKurir, Tina TičinovićWu, ShijunKuroda, NaotoWu, ShuKuroda, YujiroWu, ShupingKurokawa, TomohiroWu, SonglinKurowska, KrystynaWu, StephenKurowski, MarcinWu, Tzu-HuaKurppa, SirpaWu, WeiKurscheid, JohannaWu, WenchenKurumaddali, AbhinavWu, XiangKuryłowicz, AlinaWu, XuejianKusch-Brandt, SigridWu, YichaoKusejko, KatharinaWu, YizhengKushima, MikiWu, YouKuska, MartinWu, Yuhao (Connor)Kusnoto, BudiWu, YunKüstner, ThomasWu, ZezhouKusztal, MariuszWu, ZhanshengKutay, CatWu, ZhibinKutlu, LeventWu, Zhi-FuKüttel, AndreasWu, ZuchengKutzner, TobiasWuebbles, RyanKuusio, HannamariaWuensch, Karl L.Kuvačić, GoranWuescher, Leah M.Kuwabara, MasanariWujtewicz, MagdalenaKuwahara, KeisukeWunderlich, Shahla M.Kuzhiumparambil, UnnikrishnanWurl, JobstKuzik, NicholasWurm, FranzKuźniar, AgnieszkaWyczarska-Kokot, JoannaKvaal, KariWydra, DorisKwak, DongmiWyld, LyndaKwak, Hye WeonWyness, Adam J.Kwak, Min J.Wynne, KatieKwak, Sang HoWynne, OliviaKwak, Yi-SubWysokiński, MariuszKwan, Chi KinWyszkowska, JadwigaKwan, Christine ManlaiWyszkowski, MirosławKwan, JenniferWyszyńska, JustynaKwartnik-Pruc, AnitaWyver, ShirleyKwiatkowska, AnnaWziątek-Staśko, AnnaKwiatkowska, EwaXaplanteri, PanagiotaKwiecińska-Piróg, JoannaXaver Perrez, FranzKwon, Chan-YoungXia, ChunmingKwon, Choul WoongXia, ShunxiangKwon, HyungjuXia, Ting (Monash University)Kwon, JayminXia, Ting (Northern Illinois University)Kwon, OranXia, YankaiKwon, SoyangXia, YinglinKwon, SoyoungXian, ChaofanKwon, Yu-jinXiang, HandanKyan, AkiraXiang, WenKyathanahalli, ChandrashekaraXiang, YuanKyei, George B.Xiao, BoqiKyle, RichardXiao, ChunlingKyne, DeanXiao, LiKypriotakis, GeorgeXiao, QianKyriakopoulos, GrigoriosXiao, RenbinKyriazis, Nikolaos A.Xiao, Xin (China)Kyrozis, AndreasXiao, Xin (USA)Kyrtopoulos, SoteriosXiao, YeKyvelidou, AnastasiaXiao, YongKyvelou, Stella SofiaXiao, ZhennaKyzas, GeorgeXiao, ZhiMinL. Mallet, MarieXiaofeng, HuL’Engle, KellyXiaohua, ZhaoL’Hostis, AlainXiaojie, TianL’Ollivier, CoralieXiaoqiu, LiuLa Favor, JustinXie, HualongLa Foresta, FabioXie, HuiLa Frano, Michael R.Xie, JenniferLa Mantia, IgnazioXie, JieLa Riccia, LuigiXie, JingrongLa Russa, MauroXie, LipingLa Torre, GiuseppeXie, MinLa Touche, RoyXie, PingLaaksonen, MikkoXie, QianLaatikainen, Tiina E.Xie, RuiLabeeuw, LeenXie, ShengkunLabib, S. M.Xie, WankunLabouliere, ChristaXie, WenyanLabrosciano, ClementineXie, XiaopingLabrosse, RoxaneXie, YannanLabruna, GiuseppeXie, YingyingLabus, AleksandraXing, JunjiLabuta, JanXinyuan, ZhaoŁabuz, TomaszXiong, HaoLace, John W.Xiong, JingLacerda, André Eduardo BiscaiaXiong, YongzhuLacey, Krim K.Xu, Biao (Fudan University)Lach, SławomirXu, Biao (Hunan University)Lacham-Kaplan, OrlyXu, ChenLachenmeier, Dirk W.Xu, ChongLachowicz, SabinaXu, DingdeLacina, LukasXu, DongjuanLacombe, ScottXu, FengLadeira, Francisco Sergio BernardesXu, GuoquanLadouceur, MichelXu, HaiyanLadyman, SharonXu, IrisLafave, Lynne M. Z.Xu, JianLafforgue, MichelXu, JiupingLaganà, Antonio SimoneXu, JunLaganà, GiuseppinaXu, KaichenLago, Tiffany R.Xu, LiLago-Fuentes, CarlosXu, LinfengLagos, NellyXu, LingzhongLagravère, Manuel O.Xu, MoLaguzzi, FedericaXu, PengfeiLahav, YaelXu, QingLaheij, Alexa M. G. A.Xu, QinZiLahiri, TanayaXu, RongLahlali, RachidXu, RongqingLahmar, FellaXu, TaoLahondere, ChloeXu, TingtingLahti, MariXu, WanghongLai, AlexandraXu, WeitaoLai, AntoniaXu, WilliamLai, Chia-HsiangXu, XiaoheLai, Chian-HuiXu, XiaojiangLai, CynthiaXu, YangyangLai, GavinXu, YiLai, Julian C. L.Xu, Yi-JunLai, Peddy Pi-YingXu, YongmingLai, Tsung-HsuanXu, ZhenLai, YanhaoXu, Zhiwei (Queensland University of Technology) Lai, YuanXu, Zhiwei (The University of Queensland)Laibach, NatalieXue, BaowenLaine, MarkusXue, CunjinLaiosa, Michael D.Xue, HongLajevardipour, AlirezaXue, JiaLajolo, CarloYabar, HelmutLakes, TobiaYach, DerekLakhan, RamYadav, DhananjayLakhouit, AbderrahimYadav, Hemraj M.Laking, GeorgeYadav, LalitLakin-Thomas, Patricia L.Yagci Sokat, KezbanLal, RajYagil, DanaLalande, SophieYagmurlu, KaanLaliberté, ArleneYahaya, AbdulrazaqLalueza, José LuisYakut, Aykut MertLam, Holly Ching YuYalamova, RossitsaLam, OttoYam, Shing-Chung JonathanLam, Stanley Kam KiYamada, Ann MarieLam, TinaYamada, MasaakiLamano Ferreira, MaurícioYamada, SatoruLamas, LeonardoYamaguchi, Satoshi (Tokyo Medical and Dental University) Lamb, ChristopherYamaguchi, Satoshi (Tomishiro Central Hospital) Lamb, David S.Yamamiya, YukoLamba, VineetYamamoto, MiwaLambe, BarryYamamoto, NaomichiLambert, JoshuaYamamoto, TatsuyaLambert, Simon J.Yamanashi, HirotomoLambrechts, WimYamasaki, BriannaLamichhane, Dirga KumarYamato, TakehikoLamichhane, PurushottamYamatsu, KojiLamichhane, SantoshYamin, PauliusLammel, GerhardYan, ChonglingLammi-Taskula, JohannaYan, KaiLamparelli, Rubens Augusto CamargoYan, XuLampert, David J.Yan, YingweiLampkin, JackYanagisawa, NaokoLamy, ElsaYanar, BasakLamy, EvelynYanase, TohruLan, MinxuanYanda, MuraliLan, Ting-HsunYañez, Aina M.Lan, XinYáñez-Araque, BenitoLancho-Barrantes, Bárbara S.Yang, Angela Wei HongLand, HelenYang, AnniLand, Kenneth C.Yang, BinLanderos-Weisenberger, AngeliYang, Bo (Harvard University)Landi, DanieleYang, Bo (University of Central Florida)Landi, GianlucaYang, Byoung-eunLandram, Michael J.Yang, ChunLandry, Anaïs ThibaultYang, ChunjiangLandry, MatthewYang, Eun JooLandry, Michel D.Yang, FanLandsberger, SheldonYang, FeiliLandsem, IngerYang, GuanLandt, EskildYang, HaiboLandulfo, EduardoYang, HetongLane, AndrewYang, Hsiao-YuLane, Bradley W.Yang, Hsin-LingLane, GinnyYang, HuiLane, Michael TimothyYang, JialeLang, Cajetan ImmanuelYang, JiminLang, DiYang, JingyuLang, FengchaoYang, Jong-ChulLang, MichaelYang, JunLangan, JoanneYang, KaiLangat, Philip KibetYang, Kai-ChiangLange, EwaYang, KailunLangeard, AntoineYang, KunLange-Maia, BrittneyYang, Kwang IkLangerman, KristyYang, LeiLangiano, ElisaYang, LinshengLanglais, MickeyYang, Liu (Lydia)Lanigan, Jane D.Yang, Nan-PingLanigan, JulieYang, NingxiLannin, NatashaYang, Philip Q.Lantos, HannahYang, QiangLantta, TellaYang, QinghuaLantz, BjörnYang, RonghuiLantz, BrendanYang, RuiLanza, GiuseppeYang, ShenLanza, Marcel B.Yang, ShengyuLanza-León, PalomaYang, ShupengLanzarote-Fernández, María-DoloresYang, TaoLapierre, LisetteYang, TiananŁapińska, BarbaraYang, TianrenLaplante, David P.Yang, Tse-ChuanLapointe, MathieuYang, Wai YewLapré, FreekYang, Wei-hsiungLara Moreno, RaquelYang, WeixinLara Sánchez, Amador JesúsYang, WentaoLara-Bercial, SergioYang, Woo-HwiLaranjeira, CarlosYang, XiaoLaranjeiro, RicardoYang, XiaodongLarcombe, AlexanderYang, Xue (China)Lardone, AnnaYang, Xue (Hong Kong)Laredo-Aguilera, José AlbetoYang, Xue-MingLarese, FrancescaYang, XueyingLargent-Milnes, TallyYang, Yea-Huei KaoLarkin, CelineYang, YideLarkin, DerekYang, Yu-ChenLarkin, LouiseYang, ZanLarner, AndrewYang, ZhaojunLaroche, ElenaYang, ZhongpingLarose, TriciaYanine, Franco FernandoLarre, IsabelYannarelli, GustavoLarsen, DavidYannis, GeorgeLarsen, Malte NejstYao, Ching-TengLarson, PeterYao, DechaoLarson, Sharon L.Yao, JianLarsson, AgnetaYao, LimingLarue, GrégoireYao, QiangLarussa, TizianaYao, ShengLascelles, DesmondYao, XuanxiaLascio, Simona DiYao, YuChunLashley, MaryYap, Eng HwaŁaskawiec, EdytaYap, Kai ZhenLasker, JordanYap, KeongLaskowski, RadosławYarlas, AaronLaskowski, WaclawYassi, AnnaleeLasley, StephenYasuda, JunLasota, DorotaYat, YenLaspa, ChristinaYates, MatthewLassala, Arantzatzu L.Yates, Nathanael JamesLassemillante, Annie-ClaudeYatsunami, RieLasson, ElliotYau, YungLastella, MicheleYavuz, MehmetLászló, HorváthYayan, JosefLászló, MakraYazawa, MasahikoLatella, ChristopherYazawa, TakashiLatina, RobertoYazdi, MohammadLatinis, David KyleYe, HaihangLatino, Maria ElenaYe, HailongŁątka, DariuszYe, LimingLatkin, CarlYe, QingLatoch, AgnieszkaYe, RuningLatorre Cosculluela, CeciliaYe, WeibingLatorre-Román, Pedro ÁngelYe, XinLattanzi, Pier FrancoYe, YaozongLau, AndreasYe, YunyangLau, Bobo Hi PoYe, ZhouLau, ChunghoYeager, Ray A.Lau, Chung-Ho E.Yeckel, Catherine WeikartLau, Ho Yin EricYee Chow, SusanLau, Stephen Siu-YuYee, Tan PuiLau, Wai KinYeend, IngridLau, Wallis C. Y.Yeh, Chien HungLaudanska-Krzeminska, IdaYeh, Hsi-Jen JamesLaudisi, FedericaYeh, Jarmin ChristineLaukkanen, Anne-MariaYeh, Tsu-MingLaunay, RobertYehuda, ShoenfeldLaurà, RosariaYen, Ai-ChingLaurencia, GovenderYen, Cheng-FangLaurent, MichaëlYen, Chia-FengLaurenti, EnzoYengopal, VeerasamyLauretani, FulvioYen-Wen, ShenLauretto, MarceloYeo, ChangSeobLauridsen, Jørgen TrankjærYeo, SynLaurie, KarenYeom, ChunhoLaurila, HannuYeon Chu, ChenLaurin, JérômeYeon Han, SuLaurisz, NorbertYermolaev, OlegLauritano, DorinaYerushalmi, LalehLauterbach, John H.Yeter, DenizLauver, Diane R.Yettella, VineelLaux, PatrickYeung, Andy Wai KanLavado Contador, FranciscoYeung, ElaineLaval-Gilly, PhilippeYeung, Eugene Y. H.Lavalliere, MartinYeung, JerfLavandier, CatherineYeung, PollyLavdaniti, MariaYezer, AnthonyLavega-Burgués, PereYi, BowenLavender, AndrewYi, Dae YongLaverack, Glenn R.Yi, Jean C.Lavergne, Sidonie N.Yilmaz, ErolLavezzi, AnnaYim, BrianLaviano, AlessandroYin, FeijiaLavin, Martin F.Yin, JieLavkulich, Les M.Yin, JingjingLavoie, JoséeYin, MengLavrencic, LouiseYin, Nang HtayLaw, Chi-kinYin, WeiyanLaw, EvelynYin, WenqingLaw, KeyneYin, XiaojianLawley, MeredithYin, ZenongLawley, Sean D.Ying-ting, CaoLawrence, DavidYip, JoanneLawrence, KarlYitshak-Sade, MaayanLawrence, KatherineYiu, Nicole S. N.Lawrence-Bourne, JoanneYñiguez, RocíoLawrinenko, MichaelYoder, AaronLawson, AaronYodo, NitaLawson, Michael A.Yokoi, AyaLawson, Nicholas D.Yokoi, FumiakiLawson, Nicholas PeterYokomi, Raymond K.Layne, DianaYokomichi, HiroshiLazar, ZsofiaYokose, SatoshiLazard, AllisonYokota, ShinsoLazarevic, VanjaYon, Madeline Jun YuLazaro Gutierrez, RaquelYonath, Ada E.Lázaro Pérez, CristinaYong, Kar WeyLázaro, AntonioYoo, ChangsokLazaro, José CarlosYoo, Hah YoungLazarova, EmiliyaYoo, JieunLazarus, Cathy L.Yoo, KeunjeLazarus, SulemanYoo, Tae KeunLazo, AndreaYoon, CynthiaLazopulo, StanislavYoon, HaejinLazzari, MaurizioYoon, Ju YoungLazzaroni, Maria GraziaYoon, YeonghyeLe Brocque, RobyneYordanov, AngelLe Doaré, KirstyYork, ShereeLe Magueresse-Battistoni, BrigitteYoshida, MitsuyoshiLe Pendu, JacquesYoshihara, EijiLe, MaYoshikawa, HirokiLe, T. T. YenYoshimi, ItsuroLeach, JohnYoshimoto, JunichiroLeach, LloyedYoshinaga, JunLeal Costa, CesarYoshino, KoichiLeal, Ana MendesYost, Erin E.Leal, FernandoYou, Cheol-HwanLeal, Joana FerreiraYou, QingminLeal, WalterYou, Ren-InLean, DavidYoun, Jong-SangLeao, JoséYoung Kim, JooLeardini, DavideYoung, André FerreiraLeavens, Eleanor L. S.Young, BonnieLebert, FlorenceYoung, BrianLeBlanc, JosephYoung, BryanLeblanc, Natalie M.Young, IanLEBON, NicolasYoung, JeanineLecca, Luigi IsaiaYoung, Jennifer LouiseLeccis, FrancescaYoung, JessicaLecciso, FlaviaYoung, KateLecerf, Jean-michelYoung, MichaelLechevalier, SébastienYoung, Sean G.Lechner, ChristophYounis, MustafaLecka, IzabellaYousefinaghani, SamiraLeclercq, VirginieYousefzadeh, SepidehLecureux, JonathanYperman, JanLedda, AntonioYu, BingLedda, CaterinaYu, BolinLedin, TorbjörnYu, Chia-PinLeduc-Pessah, HeatherYu, ChuanhuaLedwoń, MateuszYu, FabiaoLee, AhlamYu, HaitaoLee, Bee WahYu, Hoi-Ying ElsieLee, BonggiYu, HongjunLee, Byung UkYu, Hong-RenLee, ChangguYu, HuayangLee, Charles C. C.Yu, Jane JieLee, Chia-ChengYu, JeongsooLee, Chien-TeYu, Jeong-sooLee, Ching-ChiYu, JiaLee, Chung GunYu, JungokLee, Chun-HsienYu, JunweiLee, DaesungYu, LiangjiangLee, DonghwanYu, LiliLee, DonghyunYu, NingLee, Du-HyeongYu, Ollie YiruLee, Eun JuYu, PingLee, EunheeYu, QiongLee, EunjaiYu, RanLee, Eun-YoungYu, RondyLee, GunaYu, SheilaLee, Hae KookYu, ShubinLee, HaeyoungYu, StanleyLee, HaneulYu, WenweiLee, HannaYu, WonjongLee, Harold H.Yu, XiaosongLee, HeeSoonYu, XinLee, Hsueh-TeYu, XinhuaLee, Huei-JaneYu, YanboLee, Hui JaiYu, YangLee, Hye MyungYu, Yi-HsiangLee, HyojungYu, ZhaowuLee, Hyoung SukYu, ZhijieLee, Hyun JinYuan, BaoyinLee, JaeheeYuan, BocongLee, JaehyuckYuan, HaoLee, JaehyukYuan, JingLee, JaeyoungYuan, KuoLee, JemyungYuan, LinxiLee, JenniferYuan, SiyangLee, Jeong HwanYuan, XinxuLee, JeongGonYuan, XueliangLee, Jeong-hoonYuan, Xue-Ming.Lee, JeongwooYuan, XuyinLee, JessicaYuan, ZhihongLee, Jin-YongYudes Gomez, CarolinaLee, JiwonYue, MinLee, Jong HyukYue, QiLee, Jong-TaeYue, WenzeLee, JoohyongYue, Xiao-GuangLee, JoonheeYuen, BelindaLee, JoonyoungYuen, John W.Lee, JuheonYuh, Chiou-HwaLee, Jung EunYumi, HashizumeLee, JungaYumoto, TetsuyaLee, Jung-HoonYun, JonghyunLee, Jung-WooYun, SunyeLee, JuyeunYunusa, IsmaeelLee, JuyoungYves, CombarnousLee, Keun HwaYvette, WohnLee, KiwonZaami, SimonaLee, Kun-HuaZabaloy, SantiagoLee, Kuo-JungZaborskis, ApolinarasLee, Kwan OkZaccarelli-Marino, Maria AngelaLee, KwanukZaccaroni, AnnalisaLee, KyoungaZach, SimaLee, KyungheeZacharakis, EmmanouilLee, KyuwanZacharias, JohnLee, Li-AngZacharis, Constantinos K.Lee, LizaZachurzok, AgnieszkaLee, LukasZada, RichardLee, Lukas Jyuhn-HsiarnZadarko, EmilianLee, Meng-ChihZadnik, VesnaLee, Mi KyungZádor, ErnöLee, Min YoungZadra, CinziaLee, MinsunZafar, AfnanLee, MunjaeZafar, MuhammadLee, OgcheolZaffina, SalvatoreLee, Paul H.Zafra, Jose LuísLee, Pei-ChunZagalaz Sánchez, María LuisaLee, PeterZagalaz-Anula, NoeliaLee, PetronaZagórska, AgnieszkaLee, Phyllis C.Zagubień, AdamLee, Po-FuZaharia, CarmenLee, PosenZahoor, HafizLee, PyoungsooZahoor, MuhammadLee, Sang KiZahradka, NicoleLee, Sang-SupZaia, VictorLee, SangtakZaias, NardoLee, Seul KiZaidi, SyedjavedLee, Seung-haZaikman, YulianaLee, Seung-JinZainul, ZarinLee, ShermanZaiter, AliLee, Soo YounZaitone, Sawsan A.Lee, Sung JinZaitsu, MasayoshiLee, SungminZajac, MarzenaLee, Tae KyoungZając-Lamparska, LudmiłaLee, TaesamZajusz-Zubek, ElwiraLee, Tian-ShyugZak, MarekLee, Tsu-ChuanZakłos-Szyda, MalgorzataLee, Tsung-HsienZakrocka, IzabelaLee, Un-KonZakrzewska-Półtorak, AlicjaLee, VivianZalewska, AnnaLee, WanhyungZalewski, Bartłomiej M.Lee, Wee Meng EricZalewski, TomaszLee, Weon-YoungZalmanova, TerezaLee, WonZaman, FarasatLee, WoojooZaman, Quazi M. Mahtab UzLee, Wui-ChiangZaman, WidaadLee, YaelimZamarripa, JorgeLee, Yen-HanZambon, IvanLee, Yo HanZambrana, Ivis GarciaLee, Young-SilZambrano, Rodrigo ElíasLee, Yu-ChiZambrano-Martinez, Jorge LuisLeemans, KathleenZamora-León, S. PilarLees, Rosemary SusanZamora-Polo, FranciscoLeeson, CarolineZampieri, NicolaLefkowitz, Rafael Y.Zampogna, BiagioLéger, MaximeZamyslowska-Szmytke, EwaLegg, PhilZanca, GiseleLeggat, Sandra G.Zandonadi, Renata PuppinLegge, AllanZandonai, ThomasLeggett, Sophia S.Zanin, Giulia MottaLegout, ArnaudZanini, MilkoLehmann, MariaZanoni, SimoneLehner, MałgorzataZapata-Diomedi, BelénLehnherr, DanZapico, Sara C.Lehtimäki, JenniZapletal, DavidLei, FanZapolski, TomaszLei, LipingZappalà, GiuseppeLei, TaoZappulla, DavidLei, Tze-HuanZarà, MartaLei, WeiZarabska-Bożejewicz, DariaLei, ZhimeiZaragoza Martí, AnaLeibovici, Didier G.Zaras, NikolaosLeicht, ChristofZárate-Marco, AnabelLeicht, Kevin T.Žardeckaitė-Matulaitienė, KristinaLeigh, JamesZare, HosseinLeigh, LucyZaręba, KorneliaLeimanis, MaraZarnowski, TomaszLeinonen, HenriZarobkiewicz, MichałLeira Feijóo, YagoZarogoulidis, PaulLeirós-Rodríguez, RaquelZarone, FernandoLeister, WolfgangZart, SebastianLeitão, LuisZas Varela, LuzLeitch, CarolZatwarnicka-Madura, BeataLeitch, DanielZaukuu, John-Lewis ZiniaLeite, Fábio R. M.Zavala-Díaz de la Serna, Francisco JavierLeite, NunoZavras, DimitrisLeithardt, ValderiZawadzki, JarosławLeiva Olivencia, Juan J.Zdarta, AgataLemay, GuyZdunek-Wiegolaska, JustynaLeme, Ana Carolina BarcoZe, TianLemière, CatherineZechariah, SunithaLemieux, KiTani ParkerZeeb, HajoLemke, BrunoZeebari, ZanginLemola, SakariZegan-Barańska, MałgorzataLemonde, ManonZegrean, MihaelaLemoyne, SabineZehnder, MatthiasLencucha, RaphaelZeidler-Erdely, Patti C.Lenell, Charles M.Zeigler, ZacharyLeng, ShuguangZeigler-Johnson, Charnita M.Lennon, RobertZeitoun, Jean-DavidLentner, CsabaZeka, KetiLenzer, BenediktZekovic, MilicaLenzi, JacopoZeleke, BerihunLeo, Carlo GiacomoZeleny, ReinhardLeocádio, Rodolfo Rocha VieiraZelikoff, Judith T.León Del Barco, BenitoZema, Demetrio AntonioLeón Zarceño, EvaZeman, Catherine L.Leon, Lee P.Zeman, PetrLeonard, LlewelwynZeng, Chang-JunLeonard, Rosemary JillZeng, HuipingLeone, AlessandroZeng, ShihongLeone, AntonioZeng, ShouzhenLeón-Gómez, Brenda BiaaniZeng, ShuwenLeonhard, ChristophZeng, WeihuaLeoni, LunaZeng, ZhenzhongLeón-Moreno, CelesteZenic, NatasaLeonn Guerrero, RachaelZepeda, Rossana C.León-Quismondo, JairoZeraatpisheh, MojtabaLeón-Rubio, José MaríaZerbini, GiuliaLeontopoulos, StefanosZerbo, StefaniaLepage, CecileZerihun, Mulatu FekaduLePoire, DavidZeunert, JoshuaLeppänen, Marja H.Zewdie, Ephrem TakeleLera, María JoséZghebi, Salwa S.Lerma, ClaudiaZgliczyński, Wojciech S.Lerman, ZviZha, ChenLermen, Richard ThomasZha, FushengLerner, HenrietteZhai, HuaiyuanLeroi, IracemaZhai, XuesongLerouge, FredericZhai, YujiaLeru, PolianaZhamsueva, Galina S.Lesener, TinoZhan, GuopingLeshem, RotemZhan, KunLesiuk, TeresaZhan, WenboLeslie, JulianZhang, AipingLeśniewicz, AnnaZhang, AnluLeso, VerusckaZhang, AnqiLessard, LauraZhang, BingLesser, IrisZhang, BinshengLesyk, LiliaZhang, ChaolanLeszczak, JustynaZhang, ChenLeszczyński, PiotrZhang, ChunqingLeszek, WanatZhang, ChunyongLetellier, Marie-ClaudeZhang, DahaiLethaus, BerndZhang, DianbaoLetsinger, AylandZhang, DingmeiLetvak, Susan A.Zhang, DuoLeung, AbrahamZhang, ErshuaiLeung, Alice W. Y.Zhang, FangLeung, Alice Wai YiZhang, FeiLeung, AmyZhang, FengtaiLeung, Doris Y. P.Zhang, GuohuaLeung, JanniZhang, GuoxingLeung, KatherineZhang, HaoLeung, Yu minZhang, HaoPengLevante, AnnalisaZhang, HeyeLeventhall, GeoffZhang, HongboLeverett, BetsyZhang, HongchaoLevesque, LucieZhang, Hui (China)Levey, DanielZhang, Hui (USA)Levitan, RobertZhang, HuifengLevitz, NicoleZhang, JanetLevkovich, InbarZhang, JiachenLevy, Jason K.Zhang, Jing-ZeLevy, StevenZhang, JueLew, EileenZhang, JunlingLewandowicz, JacekZhang, LeiLewandowska, MałgorzataZhang, LiLewandowska-Gwarda, KarolinaZhang, LiwuLewicki, SławomirZhang, LuLewin, EyalZhang, MengLewiński, AndrzejZhang, Ming-guangLewis, ChandaniZhang, MingluLewis, Ghislaine L.Zhang, MingzheLewis, HelenZhang, NanLewis, James D.Zhang, NiLewis, JessicaZhang, PuyangLewis, JohnZhang, QiLewis, Jordan P.Zhang, QianLewis, NehamaZhang, QiangzheLewis, SarahZhang, QichunLewis-Smith, HelenaZhang, RuLewko, JolantaZhang, RuilianLewkowski, OlegZhang, RuixiongLeyton Roman, MartaZhang, ShiqiuLeyva, Misti J.Zhang, ShuLezzi, Greta EnricaZhang, Shuang-QingLi, AihuaZhang, ShuoLi, AndongZhang, TaoLi, AoZhang, TingLi, Bing-YanZhang, TingbinLi, ChenshuangZhang, TingruLi, ChenyuZhang, Wei (China)Li, Chien-FengZhang, Wei (UK)Li, Chih-YingZhang, Wei (USA)Li, ChuanZhang, WeishiLi, ChunfengZhang, WenboLi, ChunjieZhang, WenjunLi, ChunkaiZhang, Wen-QingLi, ChunshengZhang, WentaoLi, Chu-ShiuZhang, Xiao-bingLi, DanZhang, XiaohengLi, DeyaoZhang, XiaojunLi, Dian-JengZhang, Xiaoning (China)Li, DongyingZhang, Xiaoning (UK)Li, EricZhang, XiaowenLi, FeiZhang, XiaoxiaLi, FrankZhang, XichaoLi, GuangdiZhang, XinLi, GuangdongZhang, XinxingLi, GuofaZhang, XinyanLi, GuohongZhang, XiujuanLi, GuohuaZhang, Xu DongLi, GuonengZhang, XueleiLi, GuoyuanZhang, XueyingLi, HaifengZhang, XufangLi, HaoZhang, XunLi, HongdaZhang, YakunLi, Hsing-HuiZhang, YanfeiLi, HuaZhang, YiLi, HuiquanZhang, YinfengLi, JiaZhang, YingLi, JiajiaZhang, YuLi, JialiangZhang, YunquanLi, JiannanZhang, ZhaoweiLi, JiayiZhang, ZhenhuaLi, JiayuZhang, ZheqingLi, JineZhang, ZhiguoLi, Jing (Hong Kong)Zhang, ZhiqiangLi, Jing (USA)Zhang, Zi-KeLi, JinghuaZhao, BaoyinLi, JoanZhao, ChuanfengLi, JunZhao, ChunhongLi, KaitaoZhao, DongLi, KaiyuanZhao, GengxingLi, KelinZhao, HuiLi, LiZhao, JieLi, LianboZhao, JiexiuLi, LingguangZhao, JinfengLi, LiubaiZhao, KaiLi, ManyuZhao, LinpingLi, Mei-LingZhao, Lu-TaoLi, MingchaoZhao, NingningLi, Ming-ChiehZhao, PeixinLi, MinghuiZhao, PengxiangLi, MuzhenZhao, QiranLi, PingZhao, ShengchuanLi, RaymondZhao, ShuaiLi, Ruei-NianZhao, ShuoLi, Rui (Northeastern University）Zhao, Wei (China)Li, Rui (University of Rochester）Zhao, Wei (USA)Li, ShichengZhao, XianLi, Shirley XinZhao, XiaohuaLi, Shu GengZhao, XinLi, ShunpingZhao, XingqingLi, ShuoZhao, YangLi, SijianZhao, YaolongLi, SimengZhao, YongfengLi, SiyueZhao, ZebinLi, TengfeiZhen, XingweiLi, TimmyZheng, GuomaoLi, WeiningZheng, GuozhongLi, WenkaiZheng, MinzhangLi, WenliangZheng, ShanLi, Xiang (Australia)Zheng, ShuanningLi, Xiang (USA)Zheng, XingLi, XiangnanZheng, XingshanLi, XiangpingZheng, XunLi, XiaodongZheng, YangxingLi, XiaofangZheng, YuanrunLi, XiaofengZheng, ZhenhuaLi, XiaoliZheng, ZhenzhenLi, XiaoshuZheng, Zi-ZhengLi, XinZhitova, ElenaLi, XingZhong, CuiqingLi, XingweiZhong, LexuanLI, XinhaiZhong, QiuanLi, XinjunZhong, TaiyangLi, XiujunZhong, YongpingLi, XiuyangZhou, CaihuaLi, XuanZhou, ChengchaoLi, Yang (China)Zhou, ChiLi, Yang (Sweden)Zhou, FengfengLi, Yao-ChuenZhou, HongyiLi, YigeZhou, HuanLi, YikeZhou, JianfengLi, YinZhou, JingLi, Yi-NaZhou, JunjieLi, YingZhou, JunminLi, YizeZhou, LianjieLi, YonghuaZhou, LifengLi, YongmingZhou, MingjieLi, YuanyuanZhou, QianlingLi, YumengZhou, QichaoLi, YunyaoZhou, QingxiangLi, YupengZhou, QingyuLi, Zheng (Eddie)Zhou, RuiLi, ZhihuaZhou, ShiLi, ZhixiongZhou, WusiLi, ZhonggenZhou, Xiao-hua AndrewLi, ZhongjuZhou, YanboLi, Zhongju (Hugh)Zhou, YishuLi, ZiminZhou, YuanLi, ZiqiZhou, YubinLiaaen Jensen, JanickeZhou, ZhiLiagkou, VasilikiZhou, Zhijun (China)Lian, Ie-BinZhou, Zhijun (USA)Liang, ChenZhou, ZhixiongLiang, ChunZhu, BangzhuLiang, HuajunZhu, BingLiang, JunZhu, Bo-WeiLiang, KongzhengZhu, ChangzhengLiang, LinzhouZhu, ChengjingLiang, YajunZhu, ChenglinLiang, YiruiZhu, DianchenLiang, YuerongZhu, DongshanLianos, PanagiotisZhu, GuoniuLianou, MariaZhu, HongkaiLiao, Hua-FangZhu, JunjunLiao, HuchangZhu, JunxiangLiao, Hung-ChangZhu, LinLiao, JiawenZhu, PengLiao, Jiunn-WangZhu, QingziLiao, Jyh-feiZhu, QiuguoLiao, XinqinZhu, ShaotongLiao, YungZhu, ShengLiao, ZehuanZhu, ShupengLiapi, CharisZhu, SixiLiaw, Shu-YiZhu, TianyuLiaw, Shyue-CherngZhu, TingshaoLiberda, Eric N.Zhu, WenfeiLicaj, IdlirZhu, WenjunLicen, SabinaZhu, XiaoqinLichtenauer, MichaelZhu, XiheLichtenberg, DovZhu, XinqiangLichtenstein, Mia BeckZhu, XiupingLichtveld, KimZhu, XueyongLicitra, GaetanoZhu, YifanLiczmańska-Kopcewicz, KatarzynaZhu, YongLicznar-Fajardo, PatriciaZhu, YuLidia Gimena, Domínguez PárragaZhu, YunchanLie, MabelZhu, ZhenLiébana-Presa, CristinaZhu, ZijieLieberman, Lauren JoyZhuang, Jie (Jackie)Lieberman, LisaZhuang, PingLiebl, AndreasZhuang, Shu-linLiehr, ThomasZhuang, XuliangLien, Yin-JuZhuo, WeihaiLieneck, CristianZhurakivska, KhrystynaLiesiene, JolantaZiada, HassanLiew, Mei ShanZiakopoulos, ApostolosLightfoot, Adam P.Ziarno, MałgorzataLignier, BaptisteZielińska, Monika A.Liguori, GiorgioZielinski, MarcinLih-Ming, YiinZieliński, WojciechLikoya, EmmanuelZielinski-Gutierrez, EmilyLikozar, BlažZiemba, EwaLiller, KarenZiembik, ZbigniewLillrank, PaulZiemińska-Stolarska, AleksandraLim, Chae-gilZierold, KristinaLim, GilbertZilic, ZeljkoLim, Hee SookZilioli, SamueleLim, Hong SooZima, EndreLim, JiseunZima, KrzysztofLim, Jun-SikZimeri, Anne MarieLim, Kam MingZimmerman, Meghan S.Lim, Kar HoZimmermann, AgnieszkaLim, Lum PengZimmermann, ClaudiaLim, OcktaeckZimney, KoryLim, SeunghooZimnoch, MiroslawLim, Yau YanZimon, DominikLim, Young-JunZineldin, MosadLima Rocha, Hermano AlexandreZinkler, MartinLima Rodrigues, Jhennyfer AlineZinszer, KateLima, MartaZiogas, IoannisLima, RodrigoZiouzenkova, OulianaLima, Tânia M.Zita, RaquelLimaye, Vijay S.Zitka, OndrejLimoncin, ErikaZitman-Gal, TaliLimonero, Joaquín T.Zito, MargheritaLimongi, JeanZiv, YairLin, AqiangZlatar, TomiLin, BeiyuZlatkin-Troitschanskaia, OlgaLin, Chao-NanZłotkowska, DagmaraLin, Cheng-WenZmyślony, PiotrLin, Che-WeiZografakis Sfakianakis, MichailLin, Chia-HuaZoia, StefaniaLin, Chien-yuZoli, AngeloLin, Chih-HaoZolnik, EdmundLin, Chih-MingZolnikov, Tara RavaLin, Ching TorngZolotukhin, Igor A.Lin, Chu-ChingZölzer, FriedoLin, Chung-Tung J.Zorbas, ChristinaLin, Chung-YingZorell, Carolin V.Lin, GangZorena, KatarzynaLin, Gen-MinZorrilla, VanessaLin, HanzhiZorrilla-Miras, PedroLin, HebinZosky, GrahamLin, Hsiao-HsienZotova, OlgaLin, HualiangZotti, FrancescaLin, Hui-JuZou, ChunbinLin, Jen-YangZou, CongmingLin, Jin-DingZou, HanLin, Jin-SengZou, PingLin, JixiangZou, YajieLin, JunLinZou, YuanchunLin, Lan-PingZouhal, HassaneLIN, Liang-tingŹróbek-Sokolnik, AnnaLin, LinZsákai, AnnamariaLin, Mei-ChenZschau, ToralfLin, MeimeiZsembeli, JózsefLin, Min-PeiZsidó, AndrásLin, Pei-FengZsirka, BalázsLin, Pi-I D.Zubac, DamirLin, QianZubelzu Minguez, SergioLin, Ro-TingZubiaur, MartaLin, SainanZubizarreta, MikelLin, ShaolingZubizarreta-Macho, ÁlvaroLin, Sheng-HsuanZucaro, FlorianaLin, Tang-HuangZucaro, RaffaellaLin, TaoZuccotti, MaurizioLin, Tz-FengŽučko, JuricaLin, WeiZuellig, RichardLin, Wei-ChunZuercher, JenniferLin, Wei-RongZuhl, MicahLin, Wei-YongZuidersma, MarijLin, XialuZuk, PiotrLin, XiangZukow, WaleryLin, Yan (UK)Žulec, MirnaLin, Yan (USA)Zumbo, GiuliaLin, YibinZumstein-Shaha, MayaLin, Yi-ChinZuniga, CristalLin, Yi-ChingZuniga-Teran, AdrianaLin, Yi-TingZuniga-Villaneuva, GregorioLin, Yong-JunZuo, DepengLin, YuZuo, YantaoLin, Yuan-ChienZuo, YiLin, Yu-HsuanZupunski, LjubicaLin, Yuh-YihZur, JoannaLin, ZhoumengŻuradzki, TomaszLinares, José Jesús GázquezŽuraulis, VidasLinares, Lucía I.Zurita Ortega, FélixLinares, ManuelZurita, FlorentinaLind, ErikZurqani, Hamdi A.Lindberg, Ann-SofieZurriaga, ÓscarLinder, Stephen H.Zuzolo, DanielaLindholm-Lehto, PetraZwęgliński, TomaszLindner, Arno E.Zweiker, DavidLindqvist, Anna KarinZwierko, TeresaLindroth, MalinZwierzchowska, AnnaLindsay, AngusZwingel, SusanneLindvall, KristinaZwozdziak, AnnaLing, ChenZwoździak, AnnaLing, ChrisZyśk, JanuszLing, JiaZyskowski, MichaelLing, SupingZyznarska-Dworczak, Beata

